# Ion-pairing π-electronic systems: ordered arrangement and noncovalent interactions of negatively charged porphyrins†

**DOI:** 10.1039/d1sc02260a

**Published:** 2021-06-14

**Authors:** Yoshifumi Sasano, Hiroki Tanaka, Yohei Haketa, Yoichi Kobayashi, Yukihide Ishibashi, Tatsuki Morimoto, Ryuma Sato, Yasuteru Shigeta, Nobuhiro Yasuda, Tsuyoshi Asahi, Hiromitsu Maeda

**Affiliations:** Department of Applied Chemistry, College of Life Sciences, Ritsumeikan University Kusatsu 525-8577 Japan maedahir@ph.ritsumei.ac.jp; Department of Materials Science and Biotechnology, Graduate School of Science and Engineering, Ehime University Matsuyama 790-8577 Japan; Department of Applied Chemistry, School of Engineering, Tokyo University of Technology Hachioji 192-0982 Japan; RIKEN Center for Biosystems Dynamics Research (BDR) Suita 565-0874 Japan; Center for Computational Sciences, University of Tsukuba Tsukuba 305-8577 Japan; Department of Physics, Graduate School of Pure and Applied Sciences, University of Tsukuba Tsukuba 305-8577 Japan; Diffraction and Scattering Division, Japan Synchrotron Radiation Research Institute Sayo 679-5198 Japan

## Abstract

In this study, charged π-electronic species are observed to develop stacking structures based on electrostatic and dispersion forces. ^*i*^π–^*i*^π Interaction, defined herein, functions for the stacking structures consisting of charged π-electronic species and is in contrast to conventional π–π interaction, which mainly exhibits dispersion force, for electronically neutral π-electronic species. Establishing the concept of ^*i*^π–^*i*^π interaction requires the evaluation of interionic interactions for π-electronic ion pairs. Free base (metal-free) and diamagnetic metal complexes of 5-hydroxy-10,15,20-tris(pentafluorophenyl)porphyrin were synthesized, producing π-electronic anions upon the deprotonation of the hydroxy unit. Coexisting cations in the ion pairs with porphyrin anions were introduced as the counter species of the hydroxy anion as a base for commercially available cations and as ion-exchanged species, *via* Na^+^ in the intermediate ion pairs, for synthesized π-electronic cations. Solid-state ion-pairing assemblies were constructed for the porphyrin anions in combination with aliphatic tetrabutylammonium (TBA^+^) and π-electronic 4,8,12-tripropyl-4,8,12-triazatriangulenium (TATA^+^) cations. The ordered arrangements of charged species, with the contributions of the charge-by-charge and charge-segregated modes, were observed according to the constituent charged building units. The energy decomposition analysis (EDA) of single-crystal packing structures revealed that electrostatic and dispersion forces are important factors in stabilizing the stacking of π-electronic ions. Furthermore, crystal-state absorption spectra of the ion pairs were correlated with the assembling modes. Transient absorption spectroscopy of the single crystals revealed the occurrence of photoinduced electron transfer from the π-electronic anion in the charge-segregated mode.

## Abstract 2

π-Electronic ion pairs comprising porphyrin-based π-electronic anions have exhibited characteristic assembling modes and resulting electronic properties such as solid-state absorption and photoinduced electron transfer.

## Graphical abstract

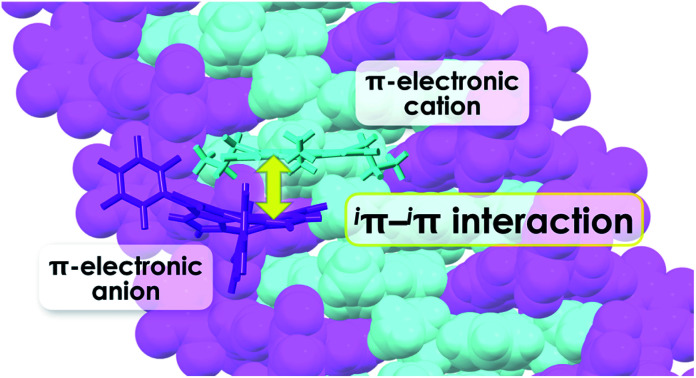

## Introduction

**Fig. 1:**
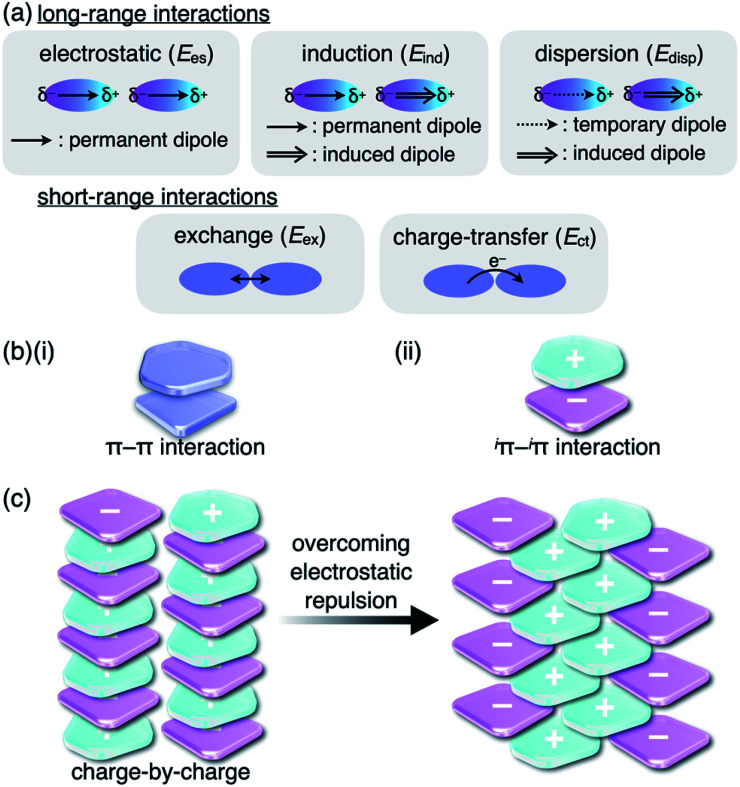
(a) Fundamental intermolecular forces, (b) representative model structures based on (i) π–π and (ii) ^*i*^π–^*i*^π interactions and (c) charge-by-charge assembly (left) and an actually observed assembly mode (right).

Noncovalent interactions are crucial for developing various assembled structures that exhibit characteristic properties.^1^ In DNA, complementary hydrogen-bonded base pairs stack *via* π–π interactions with a distance of 3.4 Å, resulting in double helical structures that carry genetic instructions.^2^ In a conceptual sense, interactions such as hydrogen-bonding and π–π interactions consist of several intermolecular forces. Fundamental intermolecular forces include electrostatic, induction and dispersion forces, which are long-range interactions based on coulombic forces (Fig. 1a).^3^ Exchange-repulsion and charge-transfer forces are short-range interactions attributed to interorbital interactions. Contributions by these intermolecular forces are dependent on the geometries and electronic structures of the constituent molecules. The total interaction energy (*E*_tot_) between molecules can be represented by the summation of the intermolecular forces (*E*_tot_ = *E*_es_ + *E*_ind_ + *E*_disp_ + *E*_ex_ + *E*_ct_). π–π Interactions, which are attractive interactions between π-electronic systems arising chiefly from dispersion (London dispersion), are essential for the assembly of π-electronic systems (Fig. 1b(i)).^4^ The contribution of dispersion forces is significant and predominant in most π–π stacking structures, although weak electrostatic forces are present even for electronically neutral π-electronic systems. The stacking orientations of π-electronic systems are controlled by minimizing electrostatic repulsion between the π-electrons and maximizing attraction between the π-electrons and positively charged σ-frameworks.^4*a*,5^ Recent advanced studies regarding π–π interactions have created opportunities for the development of various electronic materials and devices such as semiconductors.^6^

Although there are numerous reports on conventional π-electronic systems, there have been few examples of assemblies comprising charged π-electronic systems (π-electronic ions).^7^ Here, π-electronic ions are defined as π-electronic systems with a charged core unit rather than a species with an electronically neutral core bearing ionic side chains.^8,9^ Ion pairs, a combination of positively and negatively charged π-electronic systems in dispersed (monomeric) and assembling states, form various pairing modes. A detailed discussion of the properties for charged π-electronic systems requires a newly proposed conceptual interaction: ^*i*^π–^*i*^π interactions (Fig. 1b(ii)), wherein ^*i*^π represents π-electronic ions and charged π-electronic systems. The major contributive interionic forces in the ^*i*^π–^*i*^π interactions are electrostatic and dispersion forces.^10^ On the basis of the ^*i*^π–^*i*^π interactions, two distinct assembly modes of π-electronic ions can be considered for charge-by-charge assemblies, namely alternately stacking anions and cations (Fig. 1c, left), and charge-segregated assemblies, wherein identically charged species stack.^7^ These two modes are represented in the perfectly ordered arrangements of constituent π-electronic ions to easily understand what ion-pairing assemblies are. The assemblies that are obtained in forms such as crystals and liquid crystals are constructed with contributions of both the charge-by-charge and charge-segregated modes (Fig. 1c, right).

Despite numerous studies on assembling modes, the characteristics of ^*i*^π–^*i*^π interactions that exist between π-electronic ions in the solid state are not well understood. The under-exploration of ^*i*^π–^*i*^π interactions is partially attributed to the lack of appropriate ion-pairing systems. In particular, the preparation of π-electronic anions is challenging because anionic species are readily converted to other species due to their electron-rich states. Furthermore, for the development of electronic devices based on crystal engineering,^11^ it is crucial to understand the interactions within the complete crystal systems and not only those of constituent stacking ion pairs. This study focuses on the characteristic interactions operative in ion-pairing assemblies comprising π-electronic anions in combination with two different counter species: aliphatic (bulky) and π-electronic (planar) cations. The solid-state properties, including UV/vis absorption and photoinduced electron transfer correlated with the assembling modes, have also been elucidated.

## Results and discussion

### ^*i*^π–^*i*^π Interactions in the PCCp^−^–TATA^+^ ion-pairing assembly

**Fig. 2:**
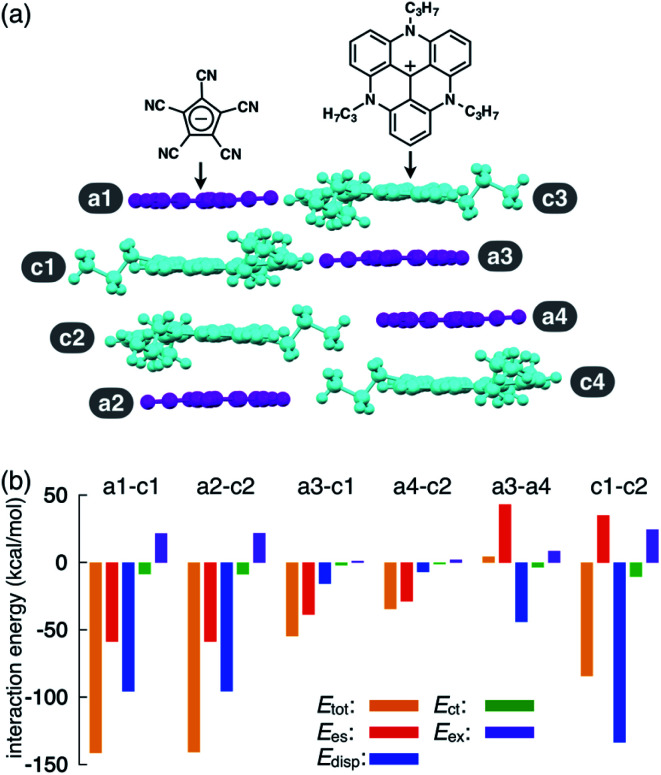
(a) Solid-state packing structure of TATA^+^–PCCp^−^ and (b) selected oppositely and identically charged ion pairs and their estimated interaction energies (kcal mol^−1^) according to EDA based on the FMO2-MP2 method using the NOSeC-V-DZP basis set with MCP (calculated packing structures for EDA were surrounded by point charges to simplify the calculation systems). Colour code in (a): magenta and cyan refer to anion and cation parts, respectively.

As an example of ion-pairing assemblies of π-electronic ion pairs, a two-by-two charge-by-charge assembly was observed in a single crystal of the ion pair of pentacyanocyclopentadienide (PCCp^−^)^12^ and 4,8,12-tripropyl-4,8,12-triazatriangulenium cation (TATA^+^)^13,14^ (Fig. 2a).^15^ In the crystal structure, the stacking distances between PCCp^−^ and TATA^+^ were 3.28 and 3.29 Å, which were within the typical π–π stacking range. The pairs of oppositely charged π-electronic ions can be observed in the stacking charge-by-charge columns as well as in the neighbouring columns. Thus, π-electronic ions compensate for their charges not only with stacking counterions but also with laterally located ions. To reveal the interaction energies between the ion pairs, energy decomposition analysis (EDA)^16^ based on the fragment molecular orbital (FMO) method (FMO2-MP2) using the NOSeC-V-DZP basis set with model core potential (MCP)^17–19^ was performed for the packing structure of PCCp^−^–TATA^+^ (Fig. 2b). It is to be noted that the calculated packing structures for EDA were surrounded by point charges to simplify the calculation systems (see Fig. S78 and Table S4† for details).^20^ The EDA calculations using the FMO yielded the resulting outputs of *E*_es_, *E*_disp_, *E*_ct_ and *E*_ex_ along with *E*_tot_. It is to be noted that the induction energy was considered to be due to the polarization effects. In FMO calculations, the fragment monomer wavefunctions were optimized with the presence of classical electrostatic potentials of other fragment monomers; hence, the converged fragment monomers were polarized. Therefore, induction energy is included in all the energy values obtained by using EDA. The *E*_tot_ values of the representative stacking ion pairs, a1–c1 and a2–c2, of PCCp^−^–TATA^+^ were −141.3 and −140.6 kcal mol^−1^, respectively, which were larger than those of laterally located ion pairs a3–c1 and a4–c2 (−54.55 and −34.36 kcal mol^−1^, respectively). The large *E*_tot_ values of stacking ion pairs were attributed to the characteristic *E*_es_ and *E*_disp_ values. In particular, the *E*_disp_ values of the stacking ion pairs were −95.54 and −95.44 kcal mol^−1^ for a1–c1 and a2–c2, respectively, which were larger than those of the lateral pairs (a3–c1 and a4–c2 for −15.53 and −6.81 kcal mol^−1^, respectively), indicating that *E*_disp_ is a crucial contributive interaction of stacking ion pairs. Favourable *E*_es_ and *E*_disp_ for stacking ion pairs indicated effective ^*i*^π–^*i*^π interactions. Interestingly, energetically disfavoured *E*_es_ values were observed for the stacking of identically charged a3–a4 and c1–c2 (43.21 and 35.01 kcal mol^−1^), whereas favoured *E*_disp_ values of −43.92 and −133.40 kcal mol^−1^ were observed for the stacking structures of PCCp^−^ and TATA^+^, respectively. π-Electronic anions with larger π-planes would show ion-pairing assemblies based on the ^*i*^π–^*i*^π interaction.

### Synthesis and characterization of porphyrin-based π-electronic ion pairs

**Fig. 3:**
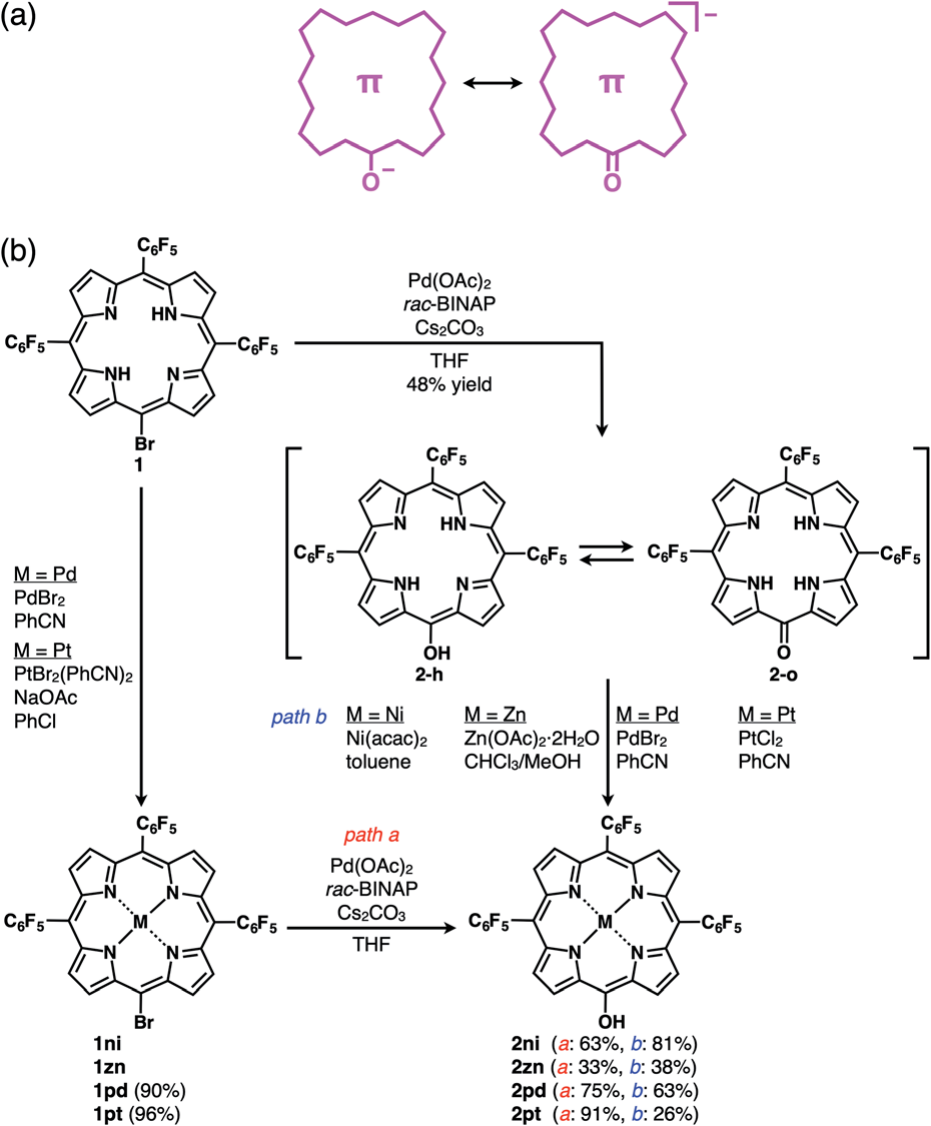
(a) Conceptual diagram for π-electronic anions obtained *via* the deprotonation of an acid unit, including the right structure representing the negative charge delocalization in the core π-system, and (b) synthetic routes for **2**, **2ni**, **2zn**, **2pd** and **2pt**. **1zn** was synthesized from 5,10,15-tris(pentafluorophenyl)porphyrin by Zn^II^ coordination and bromination,^26^ and the synthesis of **2ni** from the Ni^II^ complex of 20-bromo-5,10,15-tris(pentafluorophenyl)porphyrin (**1ni**)^23*i*^ was previously reported.^24*a*^

π-Electronic anions were prepared by the deprotonation of an acid unit introduced into π-electronic systems (Fig. 3a).^21^ The delocalization of negative charges in π-electronic systems, such as porphyrin, is essential for stabilizing the deprotonated anions. *meso*-Hydroxy-substituted porphyrins (MHPs), which are the intermediates of haem degradation^22^ and can form radical species *via* oxidation,^23^ were the candidate precursors of π-electronic anions. In our previous studies, the Ni^II^ complex of deprotonated MHP as a stable anion was reported to form solid-state charge-by-charge ion-pairing assemblies with an aliphatic tetrabutylammonium (TBA^+^) cation.^24^ Porphyrin core π-electronic systems such as π-electronic anions are suitable for ion-pairing assemblies with stacking structures and can provide insights into the ^*i*^π–^*i*^π interactions between the constituent π-electronic ions. The electronic states of porphyrin-based π-electronic anions can be tuned by varying the central metal species, which additionally affects the packing states of ion-pairing assemblies. It should be emphasized that strategies for the design and preparation of porphyrin-based π-electronic anions are considerably different from those of the charged porphyrins comprising an electronically neutral porphyrin core unit with anionic substituents^9*a*,*c*,*e*^ in the following aspects: (i) unnecessity of multiple charges, (ii) availability of *meso*-substituents for modification and (iii) utilization of genuine ^*i*^π–^*i*^π interactions.

The free base (metal-free state) and Zn^II^ complex of 20-bromo-5,10,15-tris(pentafluorophenyl)porphyrin, **1** and **1zn**, respectively, were prepared, as previously reported.^25,26^**1** was converted to Pd^II^ and Pt^II^ complexes, **1pd** and **1pt**, in 90% and 96% yields by treatment with PdBr_2_ and PtBr_2_(PhCN)_2_,^27^ respectively. Analogous to the conversion from **1ni** to **2ni**,^23*i*,24*a*^ the hydroxy group was introduced by the reaction of **1**, **1zn**, **1pd** and **1pt** with Pd(OAc)_2_, racemic 2,2′-bis(diphenylphosphino)-1,1′-binaphthyl (*rac*-BINAP) and Cs_2_CO_3_ in refluxing THF for 24 h,^23*f*^ resulting in 5-hydroxy-substituted **2**, **2zn**, **2pd** and **2pt** in 48%, 33%, 75% and 91% yields, respectively (Fig. 3b). **2** was also converted to **2ni**,^23*i*,24*a*^**2zn**, **2pd** and **2pt** in 81%, 38%, 63% and 26% yields by treatment with Ni(acac)_2_, Zn(OAc)_2_·2H_2_O, PdBr_2_ and PtCl_2_, respectively. The obtained MHPs, as the precursors of π-electronic anions, were identified by ^1^H and ^13^C NMR and MALDI-TOF-MS.

Free base **2** existed as a mixture of tautomers, hydroxyporphyrin-type **2-h** and oxophlorin-type **2-o**. The ^1^H NMR spectrum of **2** displayed signals at 9.58, 8.96 and 8.82 ppm for β-CH groups and 7.95, 7.69, 7.30 and −1.15 ppm for the NHs in DMSO-*d*_6_ at 20 °C. The signal at −1.15 ppm arose from the NH signal of **2-h**, while those at 7.95–7.30 ppm were ascribable to the NHs of **2-o**; the β-CH signals of the two tautomers coalesced. At 20 °C, the NH signals were independently observed to be in a molar ratio of 58 : 42 for **2-h** and **2-o**, becoming broader at elevated temperatures, suggesting rapid equilibrium. The ^1^H NMR spectrum of **2zn** displayed broad β-CH signals at 9.68, 8.94 and 8.84 ppm in DMSO-*d*_6_. The ^1^H NMR signals were likewise broad in DMSO-*d*_6_ at 140 °C, likely due to the presence of a small amount of radical species. On the other hand, **2pd** and **2pt** resulted in sharp β-CH signals at 9.79, 9.11, 9.04 and 9.01 ppm and at 9.73, 9.08, 9.02 and 8.99 ppm, respectively, in DMSO-*d*_6_.

The UV/vis absorption spectrum of **2** included a Soret band at 414 nm and Q bands at 515, 551, 594 and 649 nm, while that of **2zn** displayed a Soret band at 418 nm and Q bands at 548 and 597 nm as well as a low-intensity absorption band at 863 nm, possibly suggesting the presence of a small amount of radical species. The UV/vis absorption spectra of **2ni**, **2pd** and **2pt** exhibited blue-shifted bands in this order. Furthermore, the spectrum of **2ni** exhibited a Soret band at 411 nm and Q bands at 527 and 563 nm, while the spectra of **2pd** and **2pt** displayed Soret bands at 412 and 399 nm and Q bands at 523/555 and 510/542 nm, respectively. This tendency was also observed in the time-dependent (TD)-DFT-based theoretical spectra of **2ni**, **2pd** and **2pt** and their HOMO–LUMO gaps (2.774, 2.769 and 2.863 eV, respectively) at the B3LYP/6-31+G(d,p) level with LanL2DZ for Ni, Pd and Pt (Fig. S48, S50, S51, S57a, S59a and S60a†).^20^

**Fig. 4:**
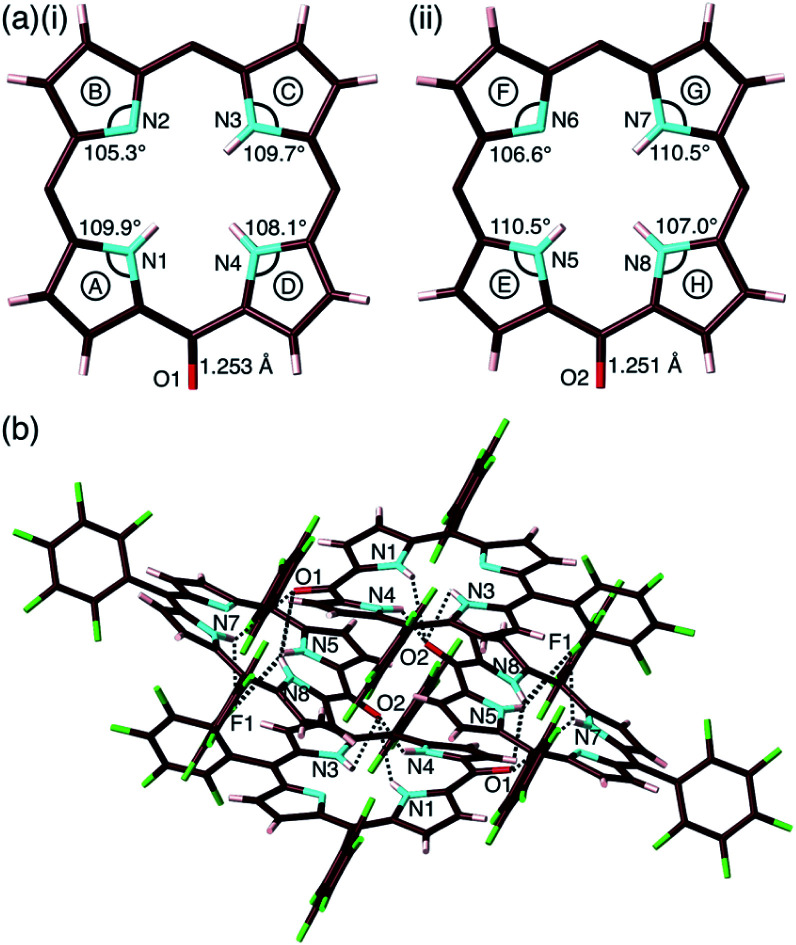
Solid-state structure of **2-o**: (a) top views with selected bond lengths and angles of (i) structure 1 and (ii) structure 2 (labels A–H represent pyrrole rings in the porphyrin framework) and (b) packing structure with hydrogen bonding. Hydrogen-bonding distances (Å): N1(–H)⋯O2 = 2.86, N3(–H)⋯O2 = 3.25, N4(–H)⋯O2 = 2.74, N5(–H)⋯O1 = 2.91, N5(–H)⋯F1 = 3.20, N7(–H)⋯O1 = 3.17, N7(–H)⋯F1 = 3.13, N8(–H)⋯O1 = 2.99 and N8(–H)⋯F1 = 2.80. Atom colour code: brown, pink, cyan, red and green refer to carbon, hydrogen, nitrogen, oxygen and fluorine, respectively. In (a), pentafluorophenyl groups are omitted for clarity.

The exact structure and solid-state assembly of **2** were elucidated as those of **2-o** by single-crystal X-ray analysis (Fig. 4),^28^ for which single crystals were prepared by the vapor diffusion of *n*-hexane into a CHCl_3_ solution of **2**. **2-o** possessed two different independent structures (structures 1 and 2), both of which showed oxophlorin-type tautomers. In structure 1, the distance between O and *meso*-C was 1.253 Å, exhibiting a C=O double-bond character, and the pyrrole C–N–C angles were 109.9°, 105.3°, 109.7° and 108.1° for A, B, C and D rings, respectively, as labelled in Fig. 4a, suggesting that the B ring contains a deprotonated nitrogen (imine), while the remaining three pyrrole rings possessed NH (amine). Structure 2 similarly indicated an O–*meso*-C distance of 1.251 Å and pyrrole C–N–C angles of 110.5°, 106.6°, 110.5° and 107.0° for E, F, G and H rings, respectively. As suggested by the small difference between 106.6° and 107.0°, the amine and imine forms could be disordered between the F and H rings. These observations along with DFT-based theoretical studies (*vide infra*) suggested the formation of oxophlorin-type **2-o** (Fig. 4a). The π–π interactions between the D rings formed tetramer structures *via* hydrogen bonding (Fig. 4b and S17†). The N(–H)⋯O distances between three NHs of structure 1 and the carbonyl oxygen of structure 2 were estimated to be 2.91, 3.16 and 2.99 Å. Interactions between three NHs of structure 2 and the carbonyl oxygen of structure 1 exhibited N(–H)⋯O distances of 2.86, 3.25 and 2.74 Å, and the N(–H)⋯F distances between the NHs of structure 2 and F atoms of structure 1 were 3.20, 3.13 and 2.80 Å.

**2** can form several tautomers, including hydroxyporphyrins (**2-h**), oxophlorins (**2-o**) and isooxophlorins possessing an sp^3^*meso*-carbon (Fig. S41†). Theoretical optimization at the B3LYP/6-31G(d,p) level (vacuum) indicated that the hydroxyporphyrin-type form is the most stable tautomer. Two oxophlorin-type NH tautomers were less stable by 1.01 and 1.89 kcal mol^−1^.^20^ The most stable structure was observed to be that of the oxophlorin-type tautomer under calculations at the PCM-B3LYP/6-31G(d,p) level in various solvents including DMSO, CH_3_CN, MeOH, acetone, CH_2_Cl_2_ and toluene (Table S3†), which is in agreement with the crystal structure (Fig. 4). Tautomer stabilities were controlled by the substituents at the *meso*-positions,^26^ as observed in 15*H*-isooxophlorins for the derivatives with 15-ethyl^26*b*^ and 15-*tert*-butyl substituents,^26*c*^ as well as in 10*H*-isooxophlorin for the 10,15,20-triphenyl-substituted derivative.^23*h*^ As observed for **2-o**, the 15-cyano-substituted derivative formed an oxophlorin-type tautomer.^26*a*^

### Deprotonation behaviour

**Fig. 5:**
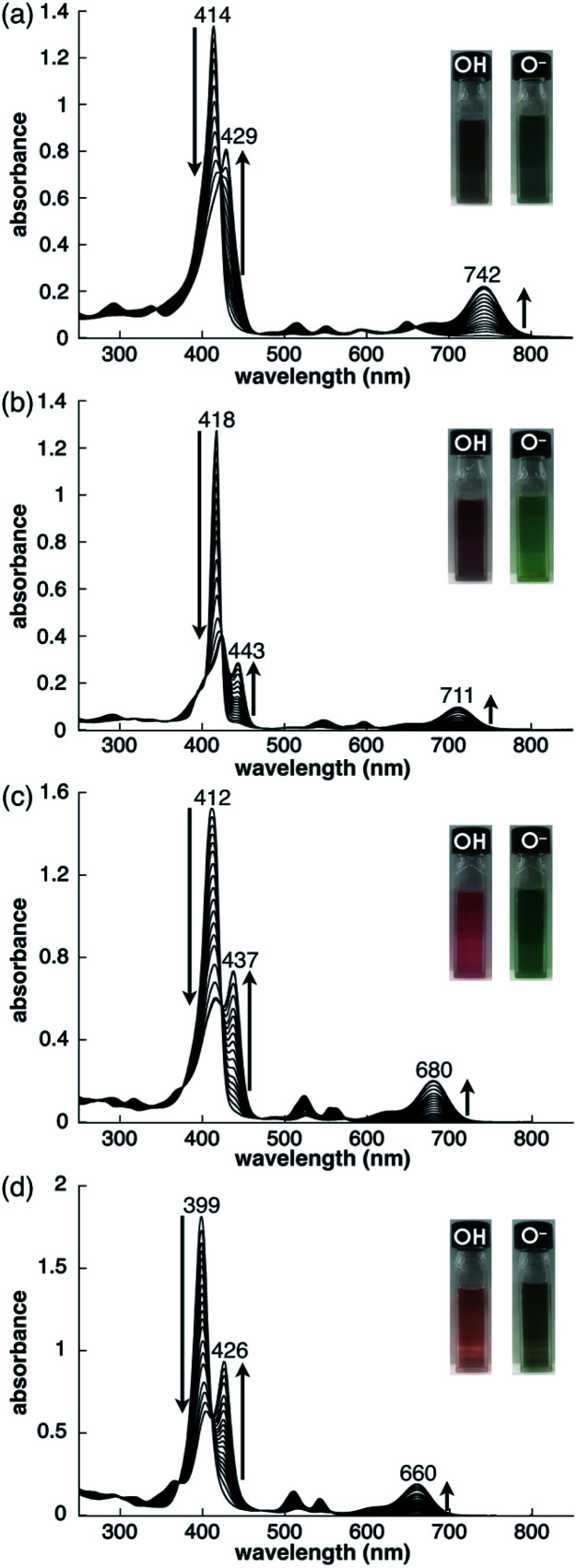
Deprotonation behaviour of (a) **2** (1.0 × 10^−5^ M), (b) **2zn** (2.4 × 10^−6^ M), with a low-intensity absorption band at 863 nm (omitted), (c) **2pd** (7.1 × 10^−6^ M) and (d) **2pt** (1.0 × 10^−5^ M) in CH_2_Cl_2_ studied by the analysis of UV/vis absorption spectral changes upon the addition of TBAOH.

As examined for **2ni**,^24*a*^ the deprotonation of **2**, **2zn**, **2pd** and **2pt** upon the addition of tetrabutylammonium hydroxide (TBAOH) in CH_2_Cl_2_ provided **2**^−^, **2zn**^−^, **2pd**^−^ and **2pt**^−^, respectively, as indicated by UV/vis absorption spectral changes (Fig. 5). The absorption maximum (*λ*_max_) of **2** at 414 nm was shifted to 429 nm for **2**^−^ with increased absorption at 742 nm. Accordingly, the solution colour of **2** changed from violet to green. The *λ*_max_ of **2zn** was shifted from 418 to 443 nm with increased absorption at 711 nm upon deprotonation, and the solution colour was changed from purple to green. Furthermore, the *λ*_max_ of **2pd** and **2pt** were shifted from 412 and 399 nm to 437 and 426 nm, respectively, with an increase in the intensity for the bands at 680 and 660 nm, respectively. For **2pd** and **2pt**, the solution colour changed from red and orange, respectively, to green. **2** exhibited the most significant spectral changes upon the addition of 1 equiv. of base. On the other hand, **2zn** required an excess amount of base for deprotonation to result in UV/vis absorption spectral changes, suggesting OH^−^ coordination to Zn^II^. Furthermore, the requirement of an excess amount of OH^−^ for the deprotonation of **2pd** and **2pt** may be due to the partial OH^−^ coordination to the metals, as observed in **2ni**.^24*a*^ The spectral changes upon deprotonation were consistent with the TD-DFT-based theoretical spectra and HOMO–LUMO gaps of their optimized structures at the B3LYP/6-31+G(d,p) level with LanL2DZ for Zn, Pd and Pt (Fig. S49–S51 and S58–S60†).^20^ The deprotonation of MHPs increased the HOMO and LUMO levels and narrowed the HOMO–LUMO gaps, resulting in longer absorption wavelengths in the anionic forms.

### Preparation of ion pairs

**Fig. 6:**
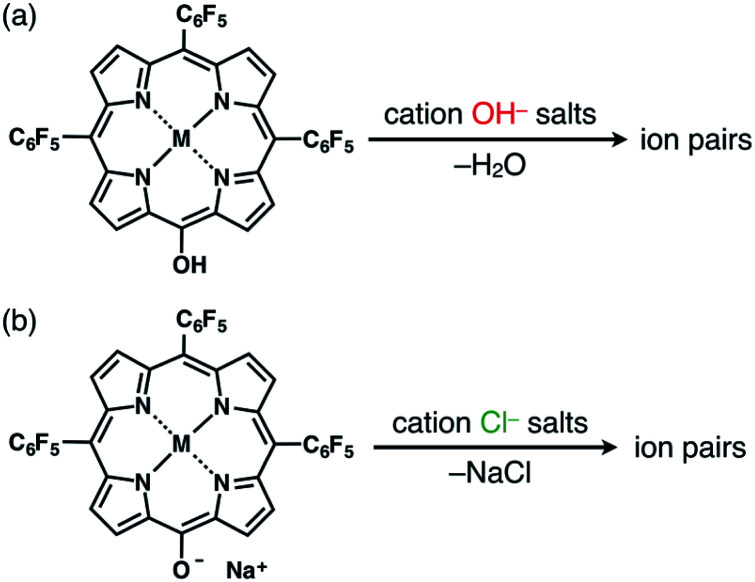
Two methods for preparing single crystals of ion pairs: (a) mixing MHPs and hydroxide salts of the desired organic cations as bases, followed by the removal of water, and (b) mixing the Na^+^ salts of porphyrin anions, prepared from the CH_2_Cl_2_ solutions of MHPs with aqueous NaOH, and Cl^−^ salts of the desired organic cations, followed by the removal of NaCl through washing with water.

The solid-state ion-pairing assemblies of π-electronic anions **2**^−^, **2ni**^−^, **2zn**^−^, **2pd**^−^ and **2pt**^−^ were fabricated in combination with cations, and their structures were revealed by single-crystal X-ray analysis. Two methods were evaluated for the preparation of ion pairs (Fig. 6): (a) mixing CH_2_Cl_2_ solutions of MHPs and 1 equiv. of hydroxide salts of desired organic cations as bases, followed by the removal of water, and (b) mixing Na^+^ salts of porphyrin anions, prepared from the CH_2_Cl_2_ solutions of MHPs with aqueous NaOH, and 1 equiv. of chloride (Cl^−^) salts of desired organic cations, followed by the removal of NaCl by washing with water.^24*b*^ In both the methods, deprotonated porphyrin anions and organic cations were soluble as ion pairs in the CH_2_Cl_2_ phase. Following CH_2_Cl_2_ evaporation, the ion pair solid-state assemblies were formed by appropriate purification techniques, such as recrystallization. The use of the hydroxide salts of organic cations in method (a) is less compared to the use of the salts of other anions, such as Cl^−^, Br^−^, OTf^−^, BF_4_^−^ and PF_6_^−^, which are readily available as organic countercation species. A variety of synthesized cations can be introduced *via* the use of their Cl^−^ salts in method (b).

### Ion pairs with bulky cations

**Fig. 7:**
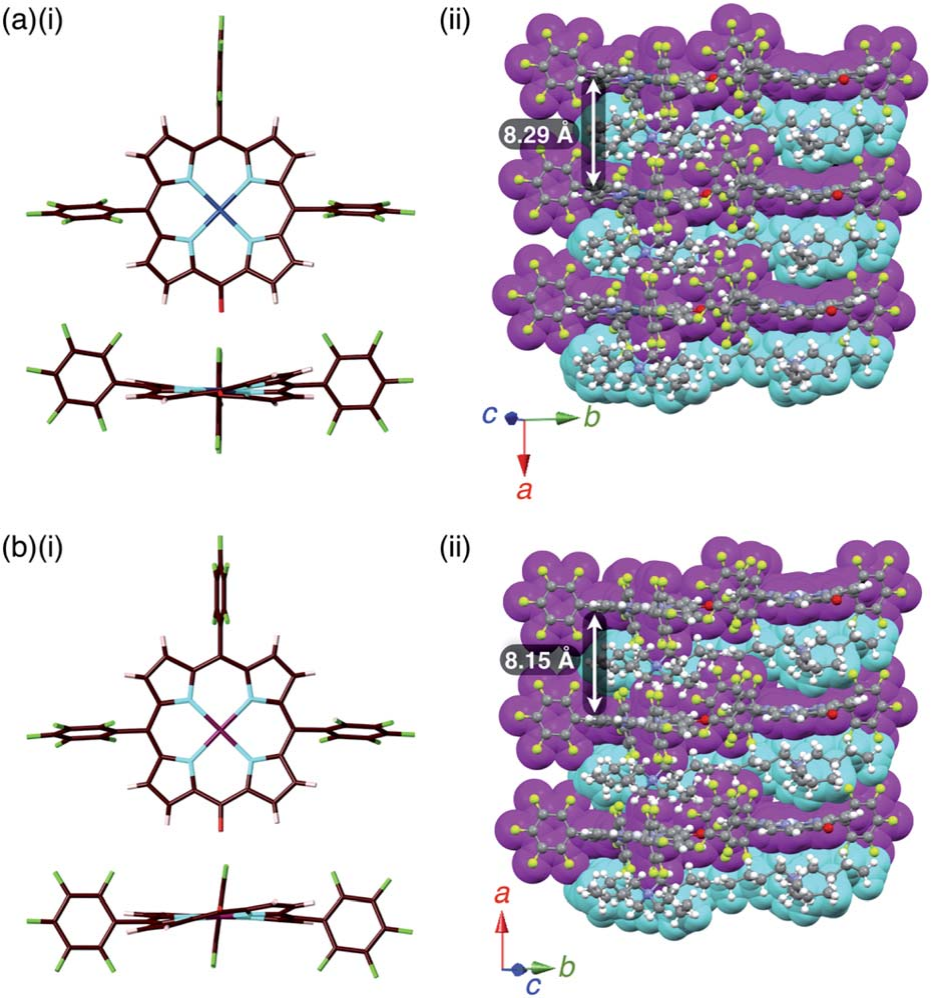
Solid-state structures of (a) **2pd**^−^–TBA^+^ and (b) **2pt**^−^–TBA^+^ as (i) top and side views of porphyrin anions and (ii) packing structures of ion pairs. Atom colour code in (i): brown, pink, cyan, red, green, blue and purple refer to carbon, hydrogen, nitrogen, oxygen, fluorine, palladium and platinum, respectively. Colour code in (ii): magenta and cyan refer to anion and cation parts, respectively.

The single crystals of **2pd**^−^–TBA^+^ and **2pt**^−^–TBA^+^ were obtained by the vapor diffusion of *n*-hexane into CH_2_Cl_2_ solutions of the 1 : 1 mixtures of the metal complex (**2pd** or **2pt**) and TBAOH (Fig. 7, S11, S12 and S22–S25†). The X-ray analyses of these crystals revealed similar charge-by-charge assembly modes. Both were chiral crystals (*P*2_1_2_1_2_1_) with cell parameters of *a* = 8.29 Å, *b* = 22.49 Å and *c* = 26.34 Å for **2pd**^−^–TBA^+^ and *a* = 8.19 Å, *b* = 22.07 Å and *c* = 25.56 Å for **2pt**^−^–TBA^+^. The Flack parameters of **2pd**^−^–TBA^+^ and **2pt**^−^–TBA^+^ were 0.18 and 0.472, respectively, suggesting that **2pd**^−^–TBA^+^ and **2pt**^−^–TBA^+^ formed crystals including one enantiomer and twin crystals, respectively. The single crystal of previously reported **2ni**^−^–TBA^+^^24*a*^ exhibited cell parameters (*Pbca*) different from those of **2pd**^−^–TBA^+^ and **2pt**^−^–TBA^+^.

The mean-plane deviations of the 24-atom planes of **2ni**^−^,^24*a*^**2pd**^−^ and **2pt**^−^ in their TBA^+^ ion pairs were 0.41, 0.28 and 0.21 Å, respectively, suggesting that the size of coordinated metals influenced the planarity of the porphyrin anions. The distances between O and *meso*-C in **2ni**^−^,^24*a*^**2pd**^−^ and **2pt**^−^ were 1.245, 1.249 and 1.235 Å, respectively, and those between O-attached *meso*-C and neighbouring α-C in **2ni**^−^,^24*a*^**2pd**^−^ and **2pt**^−^ were 1.442/1.433, 1.437/1.453 and 1.399/1.402 Å, respectively (Fig. S32†). The C=O double bond character suggested the delocalization of the anionic oxygen lone pair into the porphyrin frameworks in the order **2pt**^−^ > **2pd**^−^ > **2ni**^−^. Ion pair **2ni**^−^–TBA^+^ presented a zigzag-shaped charge-by-charge assembly,^24*a*^ while **2pd**^−^–TBA^+^ and **2pt**^−^–TBA^+^ formed more vertically aligned charge-by-charge columnar assemblies along their *a*-axis (Fig. 7a, b(ii)). The stacking distances were dependent on the planarity of anions and the locations of proximal cations. In fact, the distances between two **2ni**^−^, **2pd**^−^ and **2pt**^−^ units in their TBA^+^ ion pairs were 8.95,^24*a*^ 8.29 and 8.15 Å, respectively. The anion layer structures were formed by the intermolecular hydrogen bonding between β-CH and anionic O atoms with C(–H)⋯O distances of 3.23 and 3.29 Å for **2pd**^−^–TBA^+^ and **2pt**^−^–TBA^+^, respectively.

**Fig. 8:**
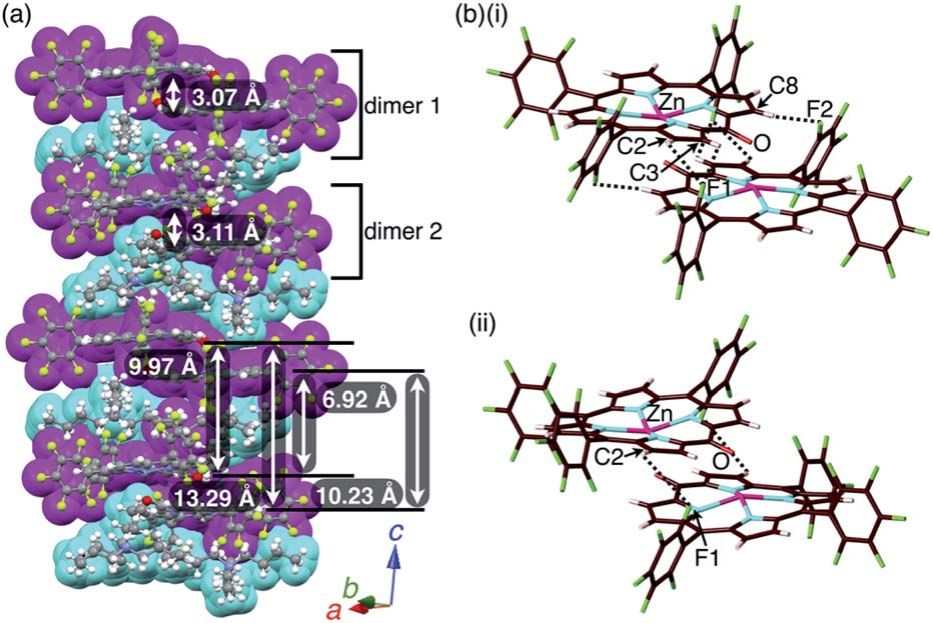
Solid-state structure of **2zn**^−^–TBA^+^ as (a) columnar structures and detailed structures of (b)(i) dimer 1 and (ii) dimer 2. Distances for (b)(i): Zn⋯O = 2.10 Å, C2(–H)⋯F1 = 3.28 Å, C3(–H)⋯F1 = 3.30 Å and C8(–H)⋯F2 = 3.30 Å; distances for (b)(ii): Zn⋯O = 2.09 Å and C2(–H)⋯F1 = 3.36 Å. Colour code in (a): magenta and cyan refer to anion and cation parts, respectively. Atom colour code in (b): brown, pink, cyan, red, green and magenta refer to carbon, hydrogen, nitrogen, oxygen, fluorine and zinc, respectively.

A single crystal of **2zn**^−^–TBA^+^ was obtained by the vapor diffusion of *n*-hexane into a CH_2_ClCH_2_Cl solution of a 1 : 1 mixture of **2zn** and TBAOH. A charge-by-charge assembly was constructed based on the dimer of **2zn**^−^, which was formed *via* the coordination of anionic O to Zn with O⋯Zn distances of 2.10 and 2.09 Å for dimers 1 and 2, respectively (Fig. 8, S10, S20 and S21†). For dimer 1, the distance between two **2zn**^−^ units was 3.07 Å. Hydrogen-bonding interactions were formed between F units and β-CH with C(–H)⋯F distances of 3.28 and 3.30 Å. For dimer 2, the distance between two **2zn**^−^ units was 3.11 Å with a C(–H)⋯F distance of 3.36 Å. Both the dimers indicated a distance of 0.404 Å for the Zn^II^ ion deviated from the plane of the four Ns. The distances between **2zn**^−^ units in dimer 1 and dimer 2 were 10.23, 6.92, 13.29 and 9.97 Å, while the dihedral angle between two **2zn**^−^ units (24-atom plane) was estimated to be 4.0° (Fig. 8a).

### Ion pairs with π-electronic cations

**Fig. 9:**
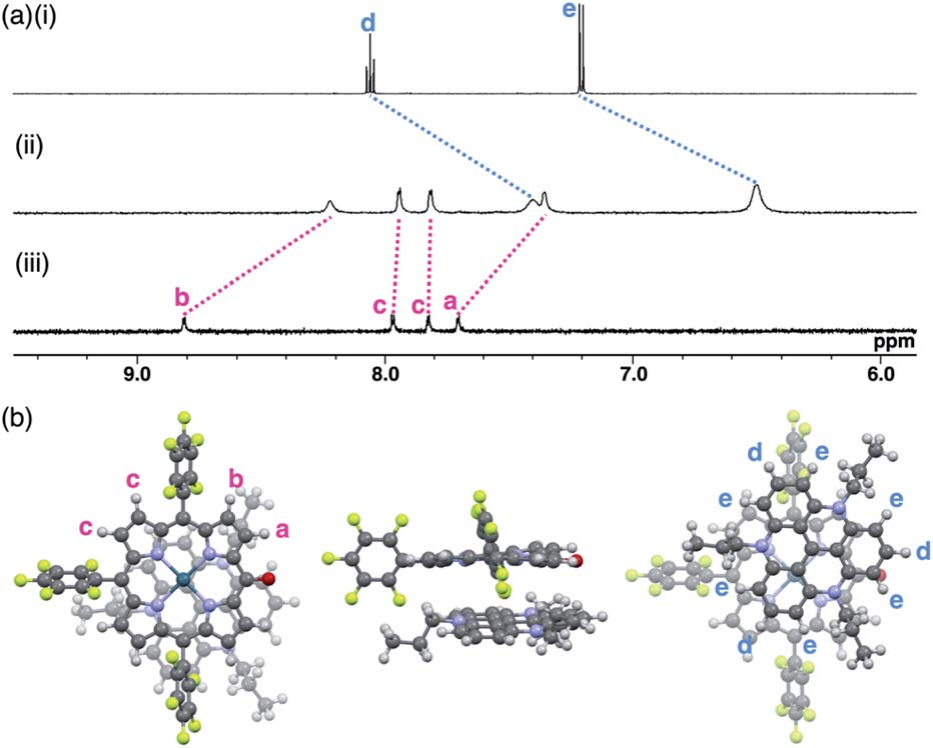
(a) ^1^H NMR spectra of (i) TATA^+^–Cl^−^, (ii) **2pd**^−^–TATA^+^ and (iii) **2pd**^−^–TBA^+^ in CD_2_Cl_2_ (1.0 × 10^−3^ M, 20 °C) and (b) optimized structure of **2pd**^−^–TATA^+^ (top, side and bottom views) at the B3LYP-GD3BJ level with the 6-31+G(d,p) basis set for C, H, N, O and F and LanL2DZ for Pd (optimized from the crystal structure of **2pd**^−^–TATA^+^).

The ion pairs of porphyrin anions with π-electronic cations, such as TATA^+^,^13,14^ were prepared *via* method (b) in Fig. 6. The **2pd**^−^–TATA^+^ ion pair exhibited characteristic ^1^H NMR signal shifts due to ion-pairing stacking in the solution state (Fig. 9 and S90†).^29^ For example, the ^1^H NMR signals of **2pd**^−^ in **2pd**^−^–TATA^+^ were observed at 8.22, 7.94, 7.82 and 7.36 ppm and those of TATA^+^ were observed at 7.41 and 6.50 ppm in CD_2_Cl_2_ at 20 °C (1.0 × 10^−3^ M). These signals were shifted upfield compared to those of **2pd**^−^–TBA^+^ and TATA^+^–Cl^−^, which were observed at 8.81, 7.97, 7.83 and 7.71 ppm as well as 8.06 and 7.21 ppm, respectively. Upon cooling to −80 °C, a **2pd**^−^ signal was shifted upfield from 8.22 to 8.16 ppm (H^b^) and three signals were shifted downfield from 7.97 and 7.82 ppm to 8.00 and 7.86 ppm (two H^c^ signals), respectively, and from 7.36 to 7.37 ppm (H^a^). Furthermore, the signals corresponding to TATA^+^ appeared at 7.68 (1H), 7.23 (1H), 6.86 (2H), 6.62 (1H), 6.28 (3H) and 6.02 (1H) ppm at −80 °C.

According to the optimized structure of **2pd**^−^–TATA^+^ at the B3LYP-GD3BJ level with the 6-31+G(d,p) basis set for C, H, N, O and F and LanL2DZ for Pd (calculated starting from the crystal structure as described below),^20^**2pd**^−^–TATA^+^ formed a stacking pair of **2pd**^−^ and TATA^+^ in the solution state (Fig. 9b and S44d†). The upfield shifts of **2pd**^−^ suggested a shielding effect from the stacking π-systems, while those of TATA^+^ signals indicated a shielding effect from stacking π-systems as well as from electron-rich anionic states. In addition, the signal shift of **2pd**^−^ upon cooling implied a strongly bound ion pair. The upfield shift of H^b^ indicated that the proton is located inside the ring current of TATA^+^, while the downfield shifts of H^c^ upon cooling suggested that their location was outside the ring current of TATA^+^. The slight downfield shift of H^a^ likely resulted from the contributions of the shielding effect from the ring current and the deshielding effect from the electron-deficient cationic state of TATA^+^. Furthermore, the split signals of TATA^+^ suggested the slow rotation of TATA^+^ at −80 °C because of the strong ion pairing (Fig. S91†).^30^ Interestingly, the UV/vis absorption spectra of the ion pairs in CH_2_Cl_2_ (1 mM) at r.t. displayed independent absorption bands for each π-electronic ion, suggesting very weak exciton coupling between π-electronic anions and cations.

The single crystals of the ion pairs, **2**^−^–TATA^+^, **2ni**^−^–TATA^+^ and **2pd**^−^–TATA^+^, were obtained by the vapor diffusion of *n*-hexane into a CH_2_Cl_2_ solution, water into an CH_3_CN solution and *n*-hexane into an EtOAc solution, respectively (Fig. S13–S15 and S26–S31†). The distances between the O and *meso*-C of **2**^−^, **2ni**^−^ and **2pd**^−^ were 1.241, 1.270 and 1.276 Å, respectively, suggesting C=O double character (Fig. S32†). The mean-plane deviation of the 24-atom plane of **2**^−^ was 0.14 Å, whereas those of **2** were 0.20 and 0.25 Å for the two independent structures in the crystal, indicating that the planarity increased with anion formation. Furthermore, the mean-plane deviations of the 24-atom planes of **2ni**^−^ and **2pd**^−^ as TATA^+^ ion pairs were 0.36 and 0.25 Å, respectively, which were smaller than those of **2ni**^−^–TBA^+^ ^24*a*^ and **2pd**^−^–TBA^+^ (Fig. S32†). These results suggested that the anion structures can be controlled by countercations.

**Fig. 10:**
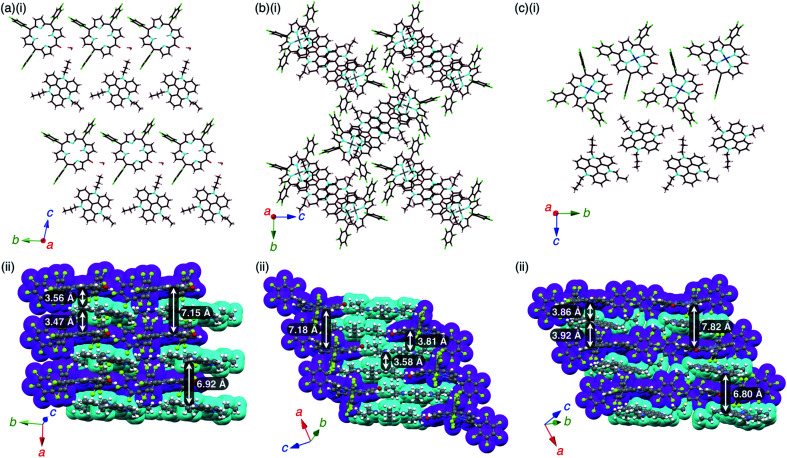
Single-crystal X-ray structures of (a) **2**^−^–TATA^+^, (b) **2ni**^−^–TATA^+^ and (c) **2pd**^−^–TATA^+^: (i) side views of layer structures and (ii) top views of stacking structures. Atom colour code in (i): brown, pink, cyan, red, green, grey and blue refer to carbon, hydrogen, nitrogen, oxygen, fluorine, nickel and palladium, respectively. Colour code in (ii): magenta and cyan refer to anion and cation parts, respectively.

**2** ^−^–TATA^+^ presented a charge-by-charge assembly mode, wherein the distances between **2**^−^ and TATA^+^ were 3.47 and 3.56 Å, implying ^*i*^π–^*i*^π interactions between the π-electronic anion and cation (Fig. 10a, S26 and S27†). The distance between two **2**^−^ units was 7.15 Å and that between two TATA^+^ units was 6.92 Å, while the dihedral angle between **2**^−^ and TATA^+^ was 12.0°. Interionic hydrogen-bonding F⋯(H–)C distances between the F atoms of C_6_F_5_ and CH of TATA^+^ were 2.95, 2.98, 3.04 and 3.15 Å. Hydrogen-bonding interactions were additionally observed between porphyrin-O and water and between porphyrin-β-CH and water, with O⋯(H–)O and O⋯(H–)C distances of 2.66 and 3.06 Å, respectively.

**2ni** ^−^–TATA^+^ exhibited both charge-by-charge and charge-segregated mode contributions, wherein anions and cations alternately stacked with partial cation stacking (Fig. 10b, S28 and S29†). The distance between two **2ni**^−^ units was 7.18 Å, while those between **2ni**^−^ and TATA^+^ and between two TATA^+^ units were 3.82 and 3.58 Å, respectively, indicating ^*i*^π–^*i*^π interactions. The dihedral angle between **2ni**^−^ and TATA^+^ was 9.07°. On the other hand, **2pd**^−^–TATA^+^ exhibited a charge-by-charge assembly mode, wherein the distances between **2pd**^−^ and TATA^+^ were 3.86 and 3.92 Å due to the weak ^*i*^π–^*i*^π interactions (Fig. 10c, S30 and S31†). The packing structures were also formed through hydrogen bonding: the intermolecular hydrogen-bonding O⋯(H–)C distance between anionic O and porphyrin-β-CH was 3.12 Å. The distance between two **2pd**^−^ units and that between two TATA^+^ units were 7.82 and 6.80 Å, respectively. The dihedral angle between **2pd**^−^ and TATA^+^ was 11.7°. These observations indicated that the packing structures of TATA^+^ ion pairs depend on the core metal ions of porphyrin anions.

### Interactions of stacking ion pairs

**Fig. 11:**
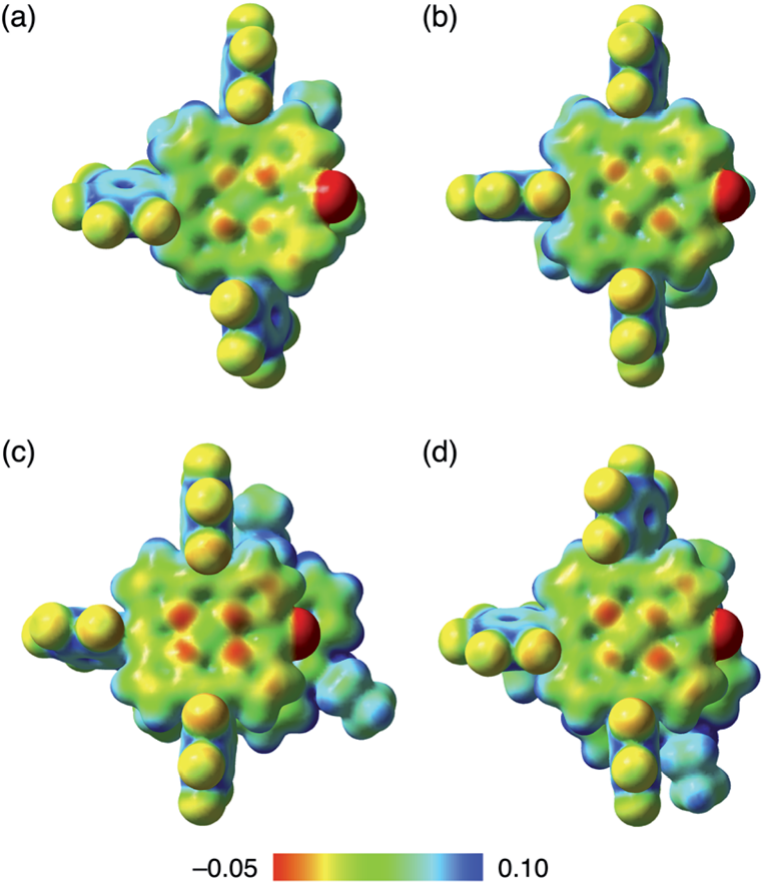
Electrostatic potential mapping of (a) **2ni**^−^–TBA^+^,^24*a*^ (b) **2pd**^−^–TBA^+^, (c) **2ni**^−^–TATA^+^ and (d) **2pd**^−^–TATA^+^ (*δ* = 0.01), calculated at the B3LYP/6-31+G(d,p) level for C, H, N, O and F with LanL2DZ for Ni and Pd, for the single-crystal X-ray structures.

Assembled ion pair behaviour in the solid state is influenced by the geometries and electronic states of constituent ions. In the packing structures of **2**^−^–TATA^+^, **2ni**^−^–TATA^+^ and **2pd**^−^–TATA^+^, the Hirshfeld surface analysis^31,32^ of porphyrin anions mapped over shape-index and curvedness properties indicated close interplanar contact and a flat region, respectively, resembling a characteristic mapping pattern for the ^*i*^π–^*i*^π stacking of π-electronic ion pairs (Fig. S37–S39†).^32*a*,*c*^ These results suggested that the packing structures of **2**^−^–TATA^+^, **2ni**^−^–TATA^+^ and **2pd**^−^–TATA^+^ were stabilized by the ^*i*^π–^*i*^π interactions. In contrast, the surfaces of porphyrin anions when paired with TBA^+^ ion pairs (**2ni**^−^–TBA^+^,^24*a*^**2zn**^−^–TBA^+^, **2pd**^−^–TBA^+^ and **2pt**^−^–TBA^+^) mapped over the shape-index properties exhibited depressions above the anionic π-planes, suggesting that the stacking structures were stabilized by the CH–π interactions (Fig. S33–S36†). Interestingly, in the electrostatic potential (ESP) diagrams at the B3LYP/6-31+G(d,p) level with LanL2DZ for Ni and Pd based on the corresponding crystal structures, the electron densities of the π-planes in **2ni**^−^ and **2pd**^−^ of TATA^+^ ion pairs were larger than those of TBA^+^ ion pairs (**2ni**^−^–TBA^+^ ^24*a*^ and **2pd**^−^–TBA^+^) (Fig. 11, S70, S72, S75 and S76†).^20^ In the X-ray structures of **2ni**^−^ and **2pd**^−^, the C–O distances of **2ni**^−^–TBA^+^ and **2pd**^−^–TBA^+^ were shorter than those of the corresponding TATA^+^ ion pairs. These results indicated that the C=O double-bond characters of **2ni**^−^ and **2pd**^−^ as TBA^+^ salts increased due to the enhanced delocalization of electrons (negative charge) of the anionic oxygen into the porphyrin framework, which occurs due to the lowering of the electron density of the anion core decreased by the positive charge of TBA^+^. Furthermore, the interionic charge delocalization between the oppositely charged species in the TATA^+^ ion pairs occurred to a lesser extent than that in the TBA^+^ ion pairs because TATA^+^ can delocalize the positive charge by itself more efficiently than TBA^+^. Therefore, the ion pairs comprising π-electronic anions and cations can effectively form assembled structures *via* electrostatic interactions.

**Fig. 12:**
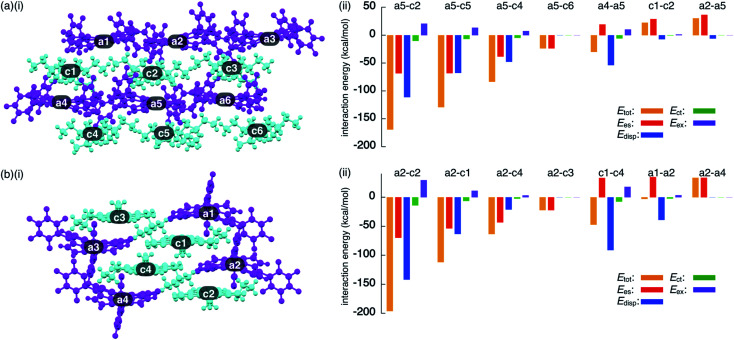
Decomposition of the total intermolecular interaction energy (*E*_tot_) of (a) **2ni**^−^–TBA^+^ and (b) **2ni**^−^–TATA^+^ for (i) single-crystal X-ray structures and (ii) selected ion pairs and their estimated interaction energies (kcal mol^−1^) according to EDA based on the FMO2-MP2 method using mixed basis sets including NOSeC-V-TZP with MCP for Ni and NOSeC-V-DZP with MCP for the other atoms (calculated packing structures for EDA were surrounded by the point charges to simplify calculation systems) (see Fig. S79 and S80† for the complete data list). Colour code in (i): magenta and cyan refer to anion and cation parts, respectively.

To investigate the contributions of interactions between π-electronic ions in the ion-pairing assemblies of single crystals, the interactions between the components of ion-pairing assemblies were estimated using EDA^16^ based on FMO2-MP2 using mixed basis sets including NOSeC-V-TZP with MCP for Ni and NOSeC-V-DZP with MCP for the other atoms (Fig. 12, S79 and S80, and Tables S5 and S6 for details†).^17–19^ In the packing structure of **2ni**^−^–TBA^+^, the *E*_tot_ values of −169.0 and −129.1 kcal mol^−1^ were observed for the charge-by-charge stacking ion pairs of a5–c2 and a5–c5, respectively, with the values of *E*_es_/*E*_disp_ being −68.34/−111.1 and −68.08/−67.66 kcal mol^−1^, respectively (Fig. 12a(ii)). Moreover, *E*_tot_ values of −83.34 and −23.77 kcal mol^−1^ were observed for a5–c4 and a5–c6 ion pairs, respectively, indicating lower attractive interaction energies than those of charge-by-charge stacking ion pairs (a5–c2 and a5–c5). Decreased *E*_disp_ values were observed for ion pairs with longer interionic distances, while *E*_es_ was relatively effective even in these cases. Identically charged ions, such as those in the c1–c2 pair, were in energetically disfavoured states and its dominant contributor is electrostatic repulsion. Furthermore, the identically charged pair a4–a5 yielded a favourable *E*_tot_ value, arising chiefly due to a contribution from dispersion forces.

The EDA calculation for the packing structure of **2ni**^−^–TATA^+^ revealed characteristic energy contributions (Fig. 12b). *E*_tot_ of the oppositely charged stacking ion pair a2–c2 was −196.2 kcal mol^−1^, whose absolute value was larger than that of the ion pair located at the lateral positions (−63.42 kcal mol^−1^ for a2–c4). The main contributions to the attractive forces in **2ni**^−^–TATA^+^ are *E*_es_ and *E*_disp_, with values of −69.72 and −141.9 kcal mol^−1^, respectively, for the a2–c2 ion pair. The *E*_es_ values were comparable to the values observed in the charge-by-charge stacking ion pairs of **2ni**^−^–TBA^+^. On the other hand, the characteristic large negative *E*_disp_ values can be attributed to the overlap of the π-planes. The *E*_disp_ values of a2–c1 (−63.30 kcal mol^−1^) and a2–c4 (−21.14 kcal mol^−1^) are lower in absolute values than that of a2–c2 due to the diminished or absent overlapping of core π-planes. Similarly, the stacking of TATA^+^ in **2ni**^−^–TATA^+^ (c1–c4) is stabilized chiefly by the contribution of dispersion forces. These results strongly support that dispersion forces are important for controlling the locations of identically charged species. Favoured *E*_disp_ values for the stacking structures of TATA^+^ are also observed in PCCp^−^–TATA^+^. On the basis of the favourable contribution by dispersion forces in the stacking of π-planes, the *E*_tot_ values of charge-by-charge stacking ion pairs in the ion-pairing assembly of **2ni**^−^–TATA^+^ are larger than those of **2ni**^−^–TBA^+^. Furthermore, the *E*_tot_ value of **2ni**^−^–TATA^+^ is larger than that of PCCp^−^–TATA^+^, particularly for *E*_disp_, suggesting that larger π-electronic ions exhibit more effective ^*i*^π–^*i*^π interactions. These results clearly demonstrate that effective electrostatic and dispersion forces as attractive forces are characteristic in the ^*i*^π–^*i*^π stacking charge-by-charge structures comprising π-electronic ions.^33^

For evaluating the results of EDA calculations without considering the effect of packing structures, monomeric ion pairs obtained from the crystal structures and their optimized structures were examined. Ion-pair monomers in the crystal state exhibited energy contributions analogous to those of the packing structures (Table S7†). The importance of electrostatic forces in ^*i*^π–^*i*^π interactions was clearly supported by the analysis of the model structure as an electronically neutral pair based on **2ni**^−^–TATA^+^ in the crystal form (Fig. S81†). The EDA calculation of the electronically neutral pair revealed decreased electrostatic forces due to the absence of charges (Table S9†). Meanwhile, the electronically neutral pair exhibited an *E*_disp_ value similar to that of the ion pair **2ni**^−^–TATA^+^, suggesting a distinction between the π–π and ^i^π–^i^π interactions and higher total interaction energies for the latter. On the other hand, optimized monomer ion pairs that were based on the crystal structures exhibited larger *E*_tot_ because of a closer ion-pairing arrangement than that in the crystal structures (Table S8†). It is to be noted that the EDA calculations of packing states and monomer ion pairs indicated that the *E*_es_ of **2ni**^−^–TATA^+^ is comparable with that of **2ni**^−^–TBA^+^, although ESP calculations of ion pairs suggested significant interionic charge delocalization in **2ni**^−^–TBA^+^. The enhanced dispersion forces of **2ni**^−^–TATA^+^ compared to that of **2ni**^−^–TBA^+^ can be attributed to the effective delocalization of negative and positive charges in **2ni**^−^ and TATA^+^, respectively. Therefore, the critical intermolecular forces for the ^*i*^π–^*i*^π interactions are electrostatic and dispersion forces.

### Solid-state absorption and photoinduced electron transfer

The charge-by-charge and charge-segregated modes in the crystal states of ion-pairing assemblies can modulate the electronic properties based on the arrangement of the π-electronic systems, as evidenced from the UV/vis absorption spectra. The UV/vis absorption spectra of the assemblies can provide valuable information on the arrangement of the π-electronic ions based on exciton coupling. It is noteworthy that a preceding study revealed the existence of weak exciton coupling between stacking oppositely charged species owing to the significantly different MO levels of the cations and anions. This was also corroborated by the faint spectral changes.^24*b*^ This property is in contrast to those of the stacking electronically neutral π-electronic systems. Thus, the crystal-state absorption spectra of the ion pairs were acquired by optical microscopy for spectroscopically examining the assembling modes of the ion-pairing assemblies.

**Fig. 13:**
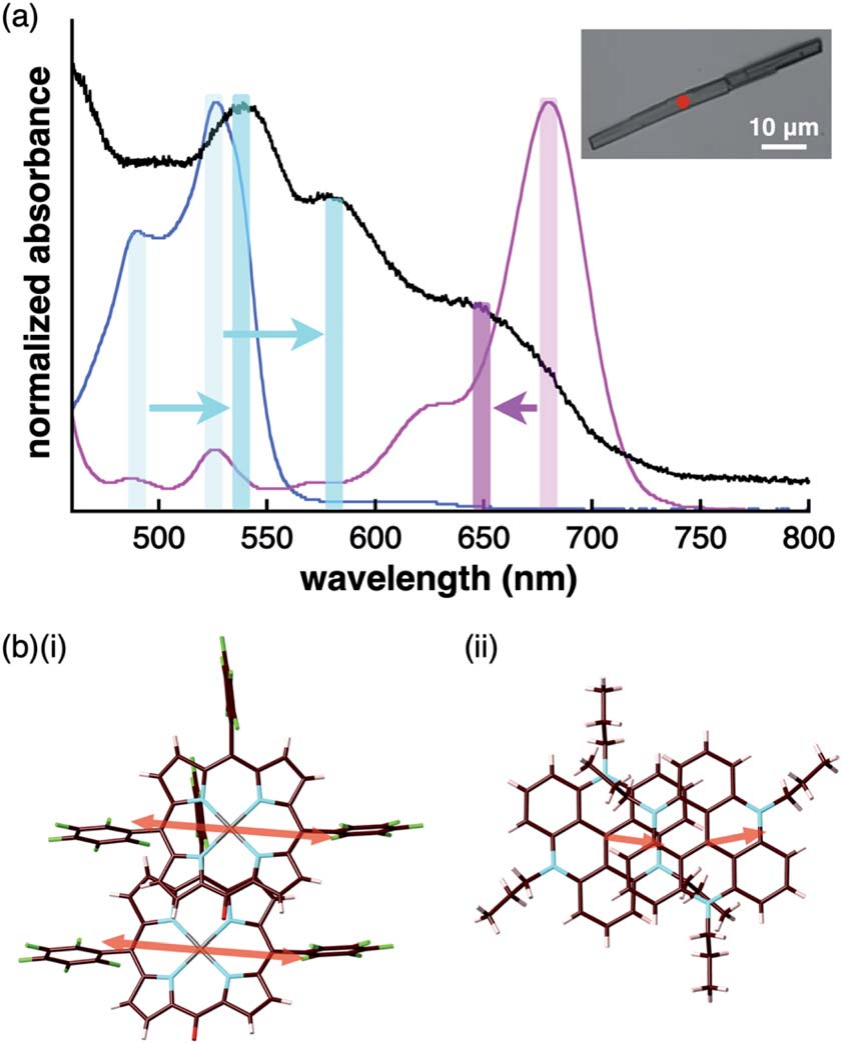
(a) UV/vis absorption spectrum of the single crystal of **2ni**^−^–TATA^+^ (black) along with those of the solution states (1 × 10^−5^ M in CH_2_Cl_2_) of **2ni**^−^–TBA^+^ (magenta) and TATA^+^–Cl^−^ (cyan) (inset: optical microscopy image of a single crystal of **2ni**^−^–TATA^+^ with the red spot indicating the position where UV/vis absorption measurement was conducted) and (b) arrangement of transition dipole moments in the dimers of (i) **2ni**^−^ and (ii) TATA^+^ in the crystal state.

The UV/vis absorption spectrum of the single crystal of **2ni**^−^–TATA^+^, which included the contribution of the charge-segregated assembly, showed significantly broad bands with *λ*_max_ at 539 and 580 nm and a shoulder at 650 nm (Fig. 13a, S95b and S96b†). These bands in the solid state were considerably distinct from the UV/vis absorption bands of **2ni**^−^ and TATA^+^ in solution. The shoulder at 650 nm was blue-shifted with respect to the band at 680 nm, which corresponded to monomeric **2ni**^−^ (1 × 10^−5^ M **2ni**^−^–TBA^+^ in CH_2_Cl_2_). In contrast, the bands at 539 and 580 nm were red-shifted with respect to those at 490 and 527 nm, which corresponded to monomeric TATA^+^ (1 × 10^−5^ M TATA^+^–Cl^−^ in CH_2_Cl_2_). Therefore, the shift in the broad absorption bands of solid-state **2ni**^−^–TATA^+^ with respect to those of the monomeric states can be considered to originate from the exciton coupling of the proximally located identically charged π-electronic systems (Fig. 10b).^34^ The arrangement of the charged π-electronic systems is more clearly discussed on the basis of their transition dipole moments, which were estimated by the TD-DFT calculations at the B3LYP/6-31+G(d,p) level (Fig. S83†). The **2ni**^−^ units, with the side-by-side-arrangement in solid-state **2ni**^−^–TATA^+^, showed parallel transition dipole moments, resulting in a blue-shifted absorption band (Fig. 13b). On the other hand, TATA^+^ formed slipped-stacking dimeric structures with head-to-tail-arranged transition dipole moments, resulting in red-shifted absorption bands. In contrast to **2ni**^−^–TATA^+^, the crystal-state absorption spectrum of **2pd**^−^–TATA^+^ showed bands corresponding to the sum of the bands of monomeric **2pd**^−^ and TATA^+^ (Fig. S95d and S96d†) owing to their arrangement in the charge-by-charge assembly (Fig. 10c). Similarly, the absorption bands of the charge-by-charge assemblies of **2ni**^−^–TBA^+^ ^24*a*^ and **2pd**^−^–TBA^+^ (Fig. 7a) were correlated with the arrangement of monomeric **2ni**^−^ and **2pd**^−^, respectively (Fig. S95a, c and S96a, c†). The excitonic interactions between identically charged species were less effective in the charge-by-charge assemblies due to the long distances. The arrangement of π-electronic ions through ^i^π–^i^π interactions enabled the modulation of the bulk-state electronic properties. It should be emphasized that the correlation between the modes of the solid-state assemblies and their absorption spectra was established in the constructed ion-pairing assemblies. To the best of our knowledge, this feature for assemblies comprising π-electronic cations and anions has not been demonstrated thus far.

**Fig. 14:**
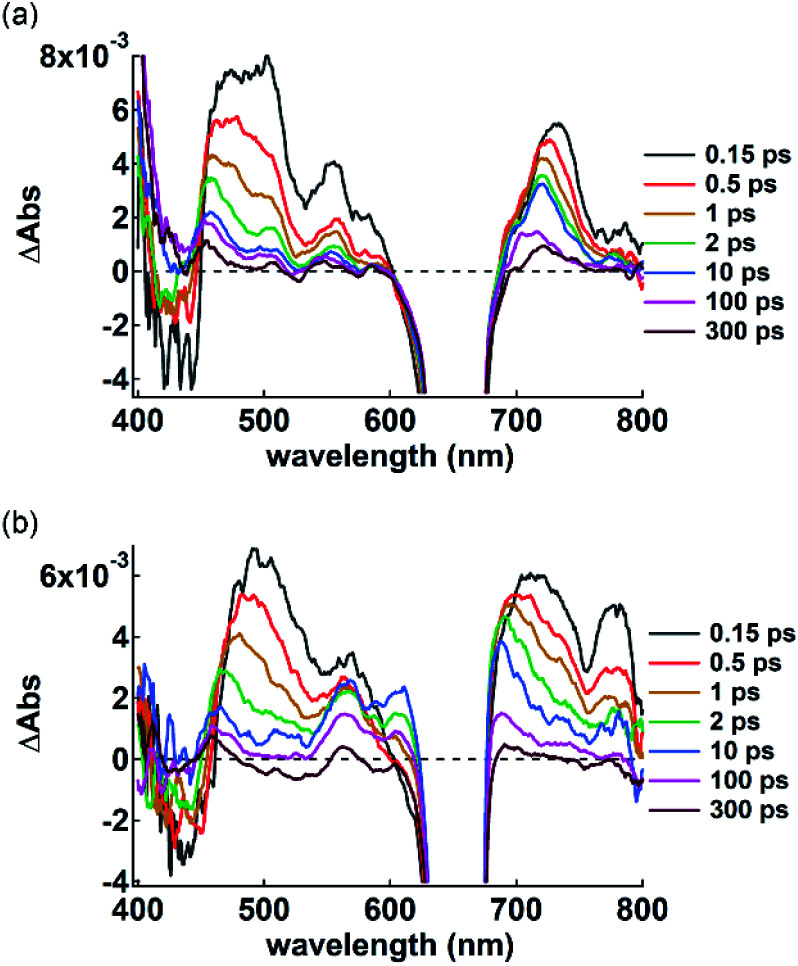
Transient absorption spectra of single crystals of (a) **2ni**^−^–TBA^+^ and (b) **2ni**^−^–TATA^+^ excited at 650 nm (<100 μJ cm^−2^) with delay time spanning from 0.15 ps to 300 ps. A negative signal ranging from 625 to 675 nm contains the scattering of the excitation beam in addition to the ground-state bleach signal.

π-Electronic anions and cations in an ordered arrangement can behave as electron donors and acceptors, respectively, thus inducing effective electron transfer in their ion-pairing assemblies.^35^ To investigate the electron transfer process, visible transient absorption spectroscopy of the single crystals of **2ni**^−^–TBA^+^ and **2ni**^−^–TATA^+^ under a microscope was performed using a 650 nm excitation pulse. A pulse of this wavelength can selectively excite **2ni**^−^ because its countercations do not absorb at 650 nm. The obtained transient absorption spectra were globally analyzed using three- or four-state sequential decay kinetic models convolved with a Gaussian pulse using the Glotaran program.^36^ The evolution-associated difference absorption spectra (EADS) thus obtained present the transient absorption spectra resolved into each component of the kinetic models (Fig. S97–S102†). Time evolution of the transient absorption spectra of **2ni**^−^–TBA^+^ (Fig. 14a) showed several sharp absorption bands at ∼475, 555 and 731 nm instantaneously after excitation. The intense negative signals in the range of 625–675 nm originate from the scattering of the excitation pulse, although the ground-state bleach signal is also superposed in this region. The peak at 731 nm was slightly shifted to shorter wavelengths and the broad absorption band at ∼475 nm was gradually resolved into two peaks at 456 and 505 nm with a lifetime of 760 fs. After the slight shifts in the bands, all the signals gradually decayed with lifetimes of 60 and 680 ps. The spectral shape and time evolution were similar to those in CH_2_Cl_2_, although the decay of the excited state in solution is much faster than those in the crystal, *i.e.*, 700 fs and 8.6 ps. The accelerated excited state dynamics of **2ni**^−^–TBA^+^ in solution relative to that in crystal is most probably due to the restricted nonradiative relaxation in the crystal. Therefore, the transient absorption spectrum of the single crystal of **2ni**^−^–TBA^+^ indicates that **2ni**^−^ is electronically well isolated due to the charge-by-charge assembly with the aliphatic cation.

The single crystal of **2ni**^−^–TATA^+^ shows an additional transient absorption band at 780 nm immediately after the excitation (Fig. 14b, 0.15 ps), in addition to the transient absorption bands which are similar to those of **2ni**^−^–TBA^+^ and are slightly broadened probably due to the π-stacking of **2ni**^−^. It is noteworthy that the simultaneous two-photon absorption can be neglected, because the excitation occurs at an exposure of less than 100 μJ cm^−2^, indicating that some electron transfer occurs through the excitation of **2ni**^−^. Moreover, spectroelectrochemical measurements in CH_3_CN suggest the oxidation of **2ni**^−^; the Ni radicals resulted in a similar absorption band at ∼790 nm (Fig. S103–S106†),^37^ while no absorption bands were observed upon the reduction of **2ni**^−^ and oxidation of TATA^+^. This strongly suggests that the ultrafast electron transfer occurs within the instrumental response function in the single crystal of **2ni**^−^–TATA^+^. This solid-state electron transfer process in π-electronic ion-pairing assemblies has not been achieved thus far.

## Conclusions

In this study, a variety of MHP metal complexes were synthesized and deprotonated to form π-electronic anions. Various ion-pairing assemblies with bulky and π-electronic cations were developed according to the introduced metal ions in the core. Ni^II^, Pd^II^ and Pt^II^ complexes exhibited charge-by-charge assembling modes with a bulky cation, while the Zn^II^ complex formed anion dimers through coordination bonds. It is to be noted that the mixtures of porphyrin anion Na^+^ salts and π-electronic cation (TATA^+^) Cl^−^ salts provided various ion pairs through ion exchange. For the obtained TATA^+^ ion pairs, free-base and Pd^II^ complex anions exhibited charge-by-charge assembling modes, while the assembly of Ni^II^ complex anions showed the contributions of charge-by-charge and charge-segregated assembling modes. The detailed analysis of interaction energies in the ion-pairing packing structures revealed that the stacking pairs of π-electronic ions exhibited ^*i*^π–^*i*^π interactions, resulting predominantly from synergistically operating electrostatic and dispersion forces. Furthermore, the correlations between the solid-state ion-pairing assembling modes based on the ^*i*^π–^*i*^π interactions and their absorption spectra were clearly observed. Electron transfer from the π-electronic anion was also observed in the crystal with contribution of the charge-segregated assembly. As various substituents can be introduced into the porphyrin frameworks, further design and synthesis of π-electronic anions for dimension-controlled assemblies, such as supramolecular gels and liquid crystals, are currently underway.

## Data availability

The datasets supporting this article have been uploaded as parts of the supplementary material.

## Author contributions

H. M. designed and conducted the project. Y. S. (Sasano), H. T. and Y. H. carried out the synthesis, characterization and property examinations. Y. K., Y. I. and T. A. evaluated the solid-state absorption and transient absorption spectra. T. M. evaluated the solution-state redox properties. R. S. and Y. S. (Shigeta) conducted the theoretical calculations. N. Y. analyzed the single-crystal X-ray structures.

## Conflicts of interest

There are no conflicts of interest to declare.

## Supplementary Material

SC-012-D1SC02260A-s001

SC-012-D1SC02260A-s002
