# Supplementary material for: Ion-pairing π-electronic systems: ordered arrangement and noncovalent interactions of negatively charged porphyrins
Source: Chem Sci. 2021 Jun 14;12(28):9645–57. doi: 10.1039/d1sc02260a (PMC8293984; doi:10.1039/d1sc02260a)
Supplement: SC-012-D1SC02260A-s001 [file SC-012-D1SC02260A-s001.pdf]

Electronic Supplementary Material (ESI) for Chemical Science.
This journal is © The Royal Society of Chemistry 2021

Electronic Supplementary Information

Ion-pairing π-electronic systems: ordered arrangement and noncovalent interactions of negatively charged porphyrins

Yoshifumi Sasano, Hiroki Tanaka, Yohei Haketa, Yoichi Kobayashi, Yukihide Ishibashi, Tatsuki Morimoto, Ryuma Sato, Yasuteru Shigeta, Nobuhiro Yasuda, Tsuyoshi Asahi and Hiromitsu Maeda*

Department of Applied Chemistry, College of Life Sciences, Ritsumeikan University, Kusatsu 525–8577, Japan, E-mail: maedahir@ph.ritsumei.ac.jp, Department of Materials Science and Biotechnology, Graduate School of Science and Engineering, Ehime University, Matsuyama 790–8577, Japan, Department of Applied Chemistry, School of Engineering, Tokyo University of Technology, Hachioji 192–0982, Japan, RIKEN Center for Biosystems Dynamics Research (BDR), Suita 565–0874, Japan, Center for Computational Sciences, University of Tsukuba, Tsukuba 305–8577, Japan, Department of Physics, Graduate School of Pure and Applied Sciences, University of Tsukuba, Tsukuba 305–8577, Japan and Diffraction and Scattering Division, Japan Synchrotron Radiation Research Institute, Sayo 679–5198, Japan

Table of Contents

1. Synthetic procedures and spectroscopic data S2
Fig. S1–7 ^{1}H and ^{13}C NMR spectra. S5
2. Deprotonation behavior S12
Fig. S8 UV/vis absorption spectral changes upon deprotonation. S12
3. X-ray crystallographic data S13
Fig. S9–15 Ortep drawings of single-crystal X-ray structures. S14
Fig. S16–31 Packing diagrams of single-crystal X-ray structures. S17
Fig. S32 Details in the crystal structures of porphyrin anions. S26
Fig. S33–40 Hirshfeld analysis of single-crystal X-ray structures. S27
4. Theoretical studies S32
Fig. S41–45 Optimized structures and ESP mappings. S32
Fig. S46–65 Molecular orbitals and theoretical UV/vis absorption spectra. S38
Fig. S66–68 Theoretical calculation for NICS. S50
Fig. S69 Theoretical calculation for ACID. S51
Fig. S70–77 ESP mappings based on crystal structures. S52
Fig. S78–80 Single-crystal X-ray structures for EDA analyses. S55
Fig. S81 Electronically neutral species for EDA analysis. S62
Fig. S82–86 Theoretical calculations for transition dipole moments. S63
Cartesian coordination of optimized structures S67
5. Solution-state behaviors of ion pairs S94
Fig. S87–92 ^{1}H NMR spectra of ion pairs. S94
Fig. S93,94 UV/vis absorption spectra of ion pairs. S99
6. Solid-state properties of ion pairs S100
Fig. S95,96 Solid-state absorption spectra. S101
Fig. S97–102 Transient absorption spectra and decay profiles. S103
Fig. S103,104 Cyclic voltammograms. S106
Fig. S105,106 Spectroelectrochemical analysis. S107

1. Synthetic procedures and spectroscopic data

General procedures. Starting materials were purchased from FUJIFILM Wako Pure Chemical Corp., Nacalai Tesque Inc., Tokyo Chemical Industry Co., Ltd., and Sigma-Aldrich Co. and were used without further purification unless otherwise stated. NMR spectra used in the characterization of products were recorded on a JEOL ECA-600 600 MHz spectrometer. All NMR spectra were referenced to solvent. UV-visible absorption spectra were recorded on a Hitachi U-3500 spectrometer. Matrix-assisted laser desorption ionization time-of-flight mass spectrometries (MALDI-TOF-MS) were recorded on a Shimadzu Axima-CFRplus. TLC analyses were carried out on aluminum sheets coated with silica gel 60 (Merck 5554). Column chromatography was performed on Wakogel C-300 and Merck silica gel 60 and 60H.

Pd^{II} complex of 20-bromo-5,10,15-tris(pentafluorophenyl)porphyrin, 1pd. To a benzonitrile solution (1.6 mL) of 20-bromo-5,10,15-tris(pentafluorophenyl)porphyrin **1**[S1] (7.13 mg, 8.03 μmol) was added $PdBr_2$ (9.08 mg, 40.2 μmol). The reaction mixture was heated to 140 °C and was stirred under air for 2 h. After benzonitrile was evaporated, the residue was chromatographed over flash silica gel column (CH_2Cl_2/*n*-hexane = 1:2) and was recrystallized from CH_2Cl_2/MeOH to afford **1pd** (7.20 mg, 7.26 μmol, 90%) as a red solid. R_f = 0.49 (CH_2Cl_2/*n*-hexane = 1:2). ^{1}H NMR (600 MHz, $CDCl_3$, 20 °C): δ (ppm) 9.79 (d, J = 4.8 Hz, 2H, β-CH), 8.84 (d, J = 4.8 Hz, 2H, β-CH), 8.81 (d, J = 4.8 Hz, 2H, β-CH), 8.80 (d, J = 5.4 Hz, 2H, β-CH). ^{13}C NMR (151 MHz, $CDCl_3$, 20 °C): δ (ppm) 146.30 (d, $J_{13C-19F}$ = 242 Hz), 142.34, 142.20 (d, $J_{13C-19F}$ = 261 Hz), 142.14, 141.97, 141.43, 137.53 (d, $J_{13C-19F}$ = 254 Hz), 134.47, 131.34, 131.01, 130.78, 115.14, 107.91, 105.43, 104.55. UV/vis (CH_2Cl_2, λ_{max}[nm] (ε, 10^5 $M^{-1}cm^{-1}$)): 412 (1.4), 524 (0.14), 556 (0.053). MALDI-TOF-MS: *m/z* (% intensity): 989.7 (100). Calcd for $C_{38}H_8BrF_{15}N_4OPd$ ($[M]^+$): 989.87.

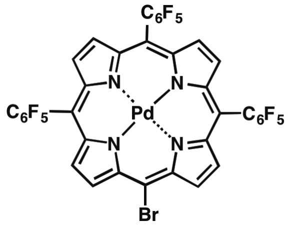

Pt^{II} complex of 20-bromo-5,10,15-tris(pentafluorophenyl)porphyrin, 1pt. To a chlorobenzene solution (1.0 mL) of **1**[S1] (15.0 mg, 16.9 μmol) were added $PtBr_2(PhCN)_2$ (47.4 mg, 0.0845 mmol) and NaOAc (13.9 mg, 0.169 mmol). The reaction mixture was heated to reflux and was stirred under air for 24 h. After chlorobenzene was evaporated, the residue was chromatographed over flash silica gel column (10% EtOAc/*n*-hexane) and was recrystallized from CH_2Cl_2/MeOH to afford **1pt** (17.6 mg, 0.0163 mmol, 96%) as an orange solid. R_f = 0.44 (10% EtOAc/*n*-hexane). ^{1}H NMR (600 MHz, $CDCl_3$, 20 °C): δ (ppm) 9.73 (d, J = 5.4 Hz, 2H, β-CH), 8.79 (d, J = 5.4 Hz, 2H, β-CH), 8.75 (s, 4H, β-CH). ^{13}C NMR (151 MHz, $CDCl_3$, 20 °C): δ (ppm) 146.23 (d, $J_{13C-19F}$ = 249 Hz), 142.26 (d, $J_{13C-19F}$ = 258 Hz), 141.90, 141.63, 141.48, 140.86, 137.57 (d, $J_{13C-19F}$ = 254 Hz), 134.30, 131.14, 130.74, 130.51, 114.71, 108.74, 106.28, 105.34. UV/vis (CH_2Cl_2, λ_{max}[nm] (ε, 10^5 $M^{-1}cm^{-1}$)): 397 (2.1), 511 (0.17), 543 (0.13). MALDI-TOF-MS: *m/z* (% intensity): 1078.8 (100). Calcd for $C_{38}H_8BrF_{15}N_4Pt$ ($[M]^+$): 1078.93.

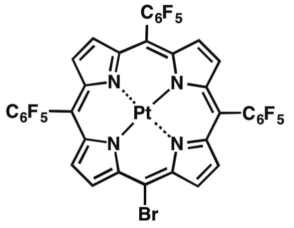

5-Hydroxy-10,15,20-tris(pentafluorophenyl)porphyrin, 2. According to the literature procedure for the related reactions,[S2] to a THF solution (19 mL) of **1**[S1] (90.0 mg, 0.101 mmol) were added $Pd(OAc)_2$ (2.30 mg, 10.2 μmol), racemic 2,2′-bis(diphenylphosphino)-1,1′-binaphthyl (*rac*-BINAP) (5.60 mg, 8.99 μmol), and Cs_2CO_3 (230 mg, 0.707 mmol). The reaction mixture was heated to reflux and was stirred under N_2 for 24 h, and then it was separated with CH_2Cl_2 and water. The organic phase was washed with brine, was dried over Na_2SO_4, and was evaporated to dryness. The residue was chromatographed over flash silica gel column (CH_2Cl_2) and was recrystallized from CH_2Cl_2/*n*-hexane to afford **2** as a mixture with a hydroxyporphyrin-type tautomer **2-h** and an oxophlorin-type tautomer **2-o** (10,15,20-tris(pentafluorophenyl)oxophlorin) (39.2 mg, 0.0475 mmol, 48%) as a green solid. **2** was also obtained from **2zn** by treatment with TFA in CH_2Cl_2. R_f = 0.11 (CH_2Cl_2). ^{1}H NMR (600 MHz, DMSO-d_6, 20 °C; this compound was obtained as a mixture of **2-h** and **2-o** in the ratio of 58:42): **2-h**: δ (ppm) 9.58 (br, 2H, β-CH), 8.96 (br, 2H, β-CH), 8.82 (br, 4H, β-CH), –1.15 (br, 2H, NH); **2-o**: δ (ppm) 9.58 (br, 2H, β-CH), 8.96 (br, 2H, β-CH), 8.82 (br, 4H, β-CH), 7.95 (br, 1H, NH), 7.69 (br, 1H, NH), 7.30 (br, 1H, NH). ^{13}C NMR (151 MHz, DMSO-d_6, 20 °C): δ (ppm): 145.91 (d, $J_{13C-19F}$ = 200 Hz), 141.64 (d, $J_{13C-19F}$ = 253 Hz), 137.42 (d, $J_{13C-19F}$ = 252 Hz) (the signals of C_6F_5 units are overlapped and the other signals broadened and were missing probably due to tautomerization). UV/vis (CH_2Cl_2, λ_{max}[nm] (ε, 10^5 $M^{-1}cm^{-1}$)): 414 (1.3), 515 (0.064), 551 (0.050), 594 (0.036), 649 (0.069). MALDI-TOF-MS: *m/z* (% intensity): 823.1 (100). Calcd for $C_{38}H_{10}F_{15}N_4O$ ($[M-H]^-$): 823.06. This compound was further characterized by single-crystal X-ray analysis as a neutral form and also an anionic form as an ion pair with triazatriangulenium (TATA) cation.[S3,4]

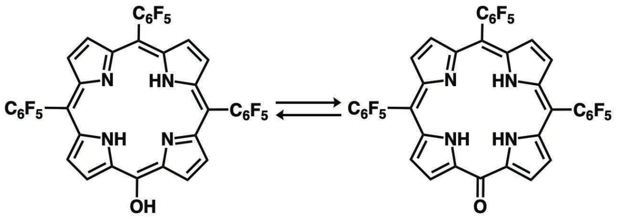

2h 2o

Ni^{II} complex of 5-hydroxy-10,15,20-tris(pentafluorophenyl)porphyrin, 2ni. **2ni** was obtained from Ni^{II} complex of 20-bromo-5,10,15-tris(pentafluorophenyl)porphyrin **1ni** as previously reported.[S5] As another procedure, to a toluene solution (1.5 mL) of **2** (5.00 mg, 6.10 μmol) was added Ni(acac)$_2$ (4.7 mg, 18.3 μmol). The reaction mixture was heated to reflux and was stirred under air for 13 h. After toluene was evaporated, the residue was chromatographed over flash silica gel column (CH_2Cl_2) and was recrystallized from CH_2Cl_2/*n*-hexane to afford **2ni** (4.5 mg, 5.1 μmol, 81%) as a red solid. Spectroscopic data were reported previously.[S5] This compound was further characterized by single-crystal X-ray analysis as an anionic form as an ion pair with TATA cation.[S3,4]

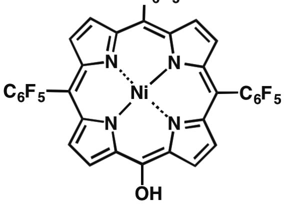

Zn^{II} complex of 5-hydroxy-10,15,20-tris(pentafluorophenyl)porphyrin, 2zn. To a THF solution (9.7 mL) of Zn^{II} complex of 20-bromo-5,10,15-tris(pentafluorophenyl)porphyrin **1zn**[S6] (40.0 mg, 42.0 μmol) were added Pd(OAc)$_2$ (1.70 mg, 7.57 μmol), *rac*-BINAP (5.56 mg, 8.39 μmol), and Cs_2CO_3 (97.0 mg, 0.298 mmol). The reaction mixture was heated to reflux and was stirred under N_2 for 24 h, and then it was separated with CH_2Cl_2 and water. The organic phase was washed with brine, was dried over Na_2SO_4, and was evaporated to dryness. The residue was chromatographed over flash silica gel column (CH_2Cl_2) and was recrystallized from CH_2Cl_2/*n*-hexane to afford **2zn** (12.3 mg, 0.0139 mmol, 33%) as a purple solid. R_f = 0.33 (CH_2Cl_2). Another procedure: To a $CHCl_3$/MeOH solution (0.5/0.1 mL) of **2** (2.96 mg, 3.59 μmol) was added Zn(OAc)$_2$·2H_2O (11.1 mg, 60.8 μmol). The reaction mixture was heated to reflux and was stirred under air for 24 h. After $CHCl_3$/MeOH was evaporated, the residue was chromatographed over flash silica gel column (CH_2Cl_2) and was recrystallized from CH_2Cl_2/*n*-hexane to afford **2zn** (1.22 mg, 1.37 μmol, 38%) as a purple solid. ^1H NMR (600 MHz, DMSO-d_6, 20 °C): δ(ppm) 12.41 (br, 1H, OH), 9.68 (br, 2H, β-CH), 8.94 (br, 2H, β-CH), 8.84 (br, 4H, β-CH). ^{13}C NMR (151 MHz, DMSO-d_6, 20 °C): δ(ppm) broad signal was observed probably due to the presence of radical species. UV/vis (CH_2Cl_2, λ_{max}[nm] (ε, 10^5 $M^{-1}cm^{-1}$)): 418 (5.3), 548 (0.17), 597 (0.14). MALDI-TOF-MS: *m/z* (% intensity): 884.9 (100). Calcd for $C_{38}H_8F_{15}N_4OZn$ ([M – H]$^-$): 884.98. This compound was further characterized by single-crystal X-ray analysis as an anionic form as an ion pair with tetrabutylammonium (TBA) cation.

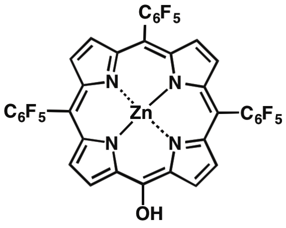

Pd^{II} complex of 5-hydroxy-10,15,20-tris(pentafluorophenyl)porphyrin, 2pd. To a THF solution (15 mL) of **1pd** (83.2 mg, 83.8 μmol) were added Pd(OAc)$_2$ (1.88 mg, 8.38 mmol), *rac*-BINAP (11.1 mg, 16.7 μmol), and Cs_2CO_3 (191 mg, 0.587 mmol). The reaction mixture was heated to reflux and was stirred under N_2 for 24 h, and then it was separated with CH_2Cl_2 and water. The organic phase was washed with brine, was dried over Na_2SO_4, and was evaporated to dryness. The residue was chromatographed over flash silica gel column (1:1 CH_2Cl_2/*n*-hexane) and was recrystallized from CH_2Cl_2/*n*-hexane to afford **2pd** (58.4 mg, 0.0629 mmol, 75%) as a red solid. Another procedure: To a benzonitrile solution (1 mL) of **2** (3.19 mg, 3.89 μmol) was added $PdBr_2$ (17.0 mg, 47.9 μmol). The reaction mixture was heated to 150 °C and was stirred under air for 2 h. After benzonitrile was evaporated, the residue was chromatographed over flash silica gel column (CH_2Cl_2) and was recrystallized from EtOAc/*n*-hexane to afford **2pd** (2.26 mg, 2.43 μmol, 63%) as a red solid. R_f = 0.29 (CH_2Cl_2). ^1H NMR (600 MHz, DMSO-d_6, 20 °C): δ(ppm) 9.79 (br, 2H, β-CH), 9.11 (br, 2H, β-CH), 9.04 (br, 2H, β-CH), 9.01 (br, 2H, β-CH). ^{13}C NMR (151 MHz, DMSO-d_6, 20 °C): δ (ppm) 145.96 (d, $J_{13C-19F}$ = 241 Hz), 143.08, 141.76 (d, $J_{13C-19F}$ = 244 Hz), 139.91, 139.37, 137.51 (d, $J_{13C-19F}$ = 246 Hz), 135.54, 132.69, 131.02, 128.38, 128.24, 114.53, 104.67, 99.89 (one signal of the porphyrin ring is missing due to overlapping, and the signals of C_6F_5 units are also overlapped). UV/vis (CH_2Cl_2, λ_{max}[nm] (ε, 10^5 $M^{-1}cm^{-1}$)): 412 (2.1), 523 (0.13), 555 (0.071). MALDI-TOF-MS: *m/z* (% intensity): 927.0 (100). Calcd for $C_{38}H_8F_{15}N_4OPd$ ([M – H]$^-$): 926.95. This compound was further characterized by single-crystal X-ray analysis as a neutral form and also an anionic form as ion pairs with TBA and TATA[S3,4] cations.

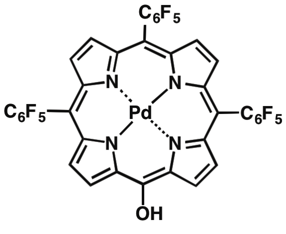

Pt^{II} complex of 5-hydroxy-10,15,20-tris(pentafluorophenyl)porphyrin, 2pt. To a THF solution (9.2 mL) of

1pt (55.5 mg, 51.4 μmol) were added $Pd(OAc)_2$ (1.15 mg, 5.14 μmol), *rac*-BINAP (6.81 mg, 12.8 μmol), and Cs_2CO_3 (117 mg, 0.360 mmol). The reaction mixture was heated to reflux and was stirred under N_2 for 24 h, and then it was separated with CH_2Cl_2 and water. The organic phase was washed with brine, was dried over Na_2SO_4, and was evaporated to dryness. The residue was chromatographed over flash silica gel column (1:1 CH_2Cl_2/*n*-hexane) and was recrystallized from CH_2Cl_2/*n*-hexane to afford **2pt** (47.6 mg, 0.0468 μmol, 91%) as an orange solid. Another procedure: To a benzonitrile solution (1 mL) of **2** (5.00 mg, 4.63 μmol) was added $PtCl_2$ (8.10 mg, 23.2 μmol). The reaction mixture was heated to 150 °C and was stirred under air for 4 days. After benzonitrile was evaporated, the residue was chromatographed over flash silica gel column (CH_2Cl_2) and was recrystallized from CH_2Cl_2/*n*-hexane to afford **2pt** (1.59 mg, 1.57 μmol, 26%) as a red solid. R_f = 0.31 (CH_2Cl_2). ^{1}H NMR (600 MHz, DMSO-d_6, 20 °C): δ (ppm) 9.73 (d, J = 4.8 Hz, 2H, β-CH), 9.08 (d, J = 4.8 Hz, 2H, β-CH), 9.02 (d, J = 4.8 Hz, 2H, β-CH), 8.99 (d, J = 4.8 Hz, 2H, β-CH). ^{13}C NMR (151 MHz, DMSO-d_6, 20 °C): δ (ppm) 145.91 (d, $J_{13C-19F}$ = 243 Hz), 144.93, 142.43, 141.84 (d, $J_{13C-19F}$ = 249 Hz), 138.93, 138.71, 137.56 (d, $J_{13C-19F}$ = 250 Hz), 135.20, 132.62, 130.59, 128.11, 128.09, 114.14, 105.60, 100.57. UV/vis (CH_2Cl_2, λ_{max}[nm] (ε, 10^5 $M^{-1}cm^{-1}$)): 399 (1.8), 510 (0.15), 542 (0.10). MALDI-TOF-MS: *m/z* (% intensity): 1017.2 (100). Calcd for $C_{38}H_9F_{15}N_4OPt$ ($[M]^-$): 1017.02. This compound was further characterized by single-crystal X-ray analysis as an anionic form as an ion pair with TBA cation.

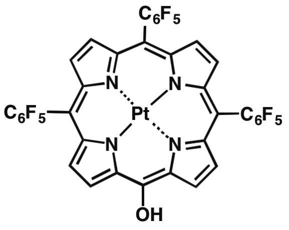

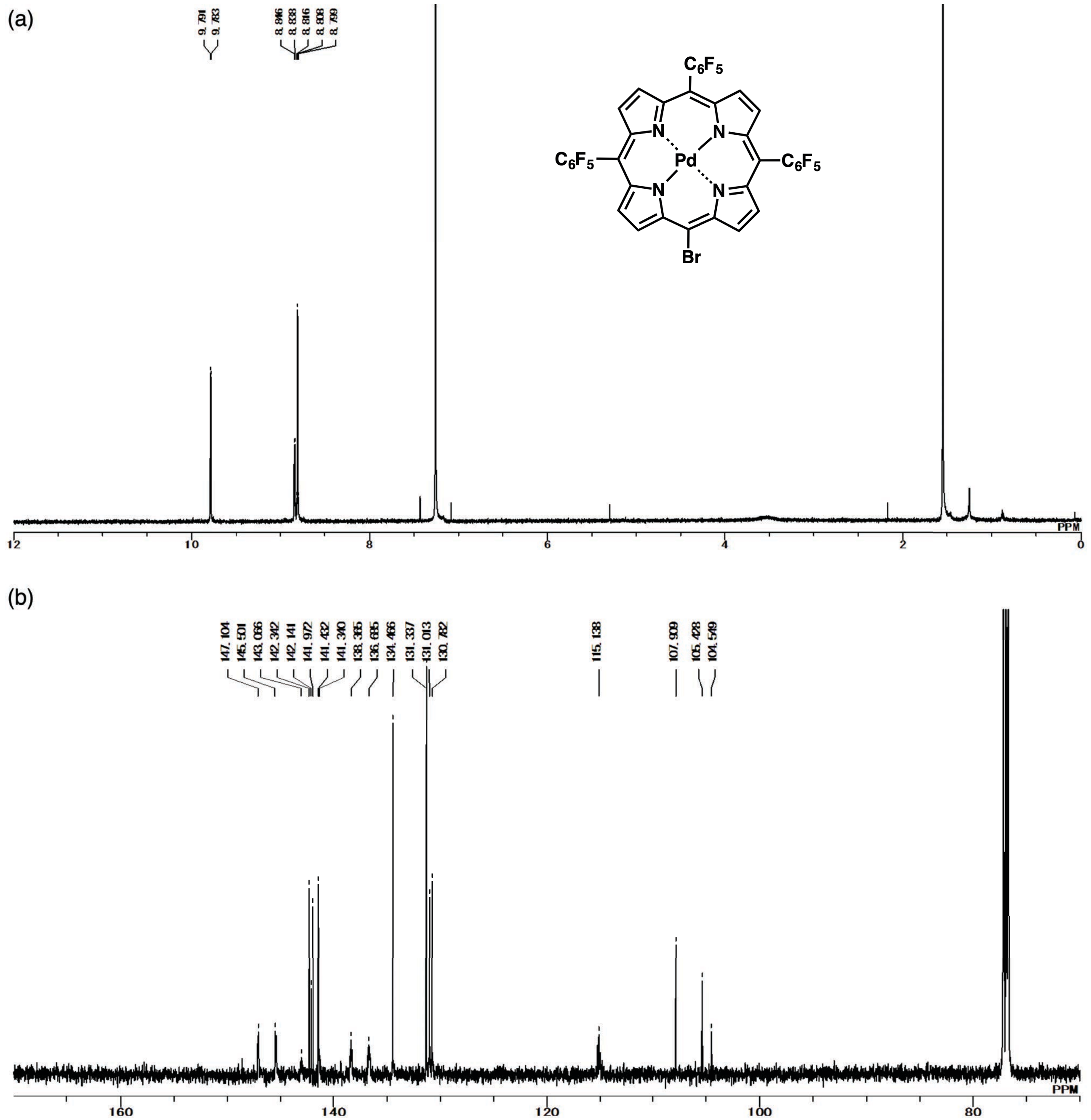

Fig. S1 (a) ^{1}H NMR and (b) ^{13}C NMR spectra of **1pd** in $CDCl_3$ at 20 °C.

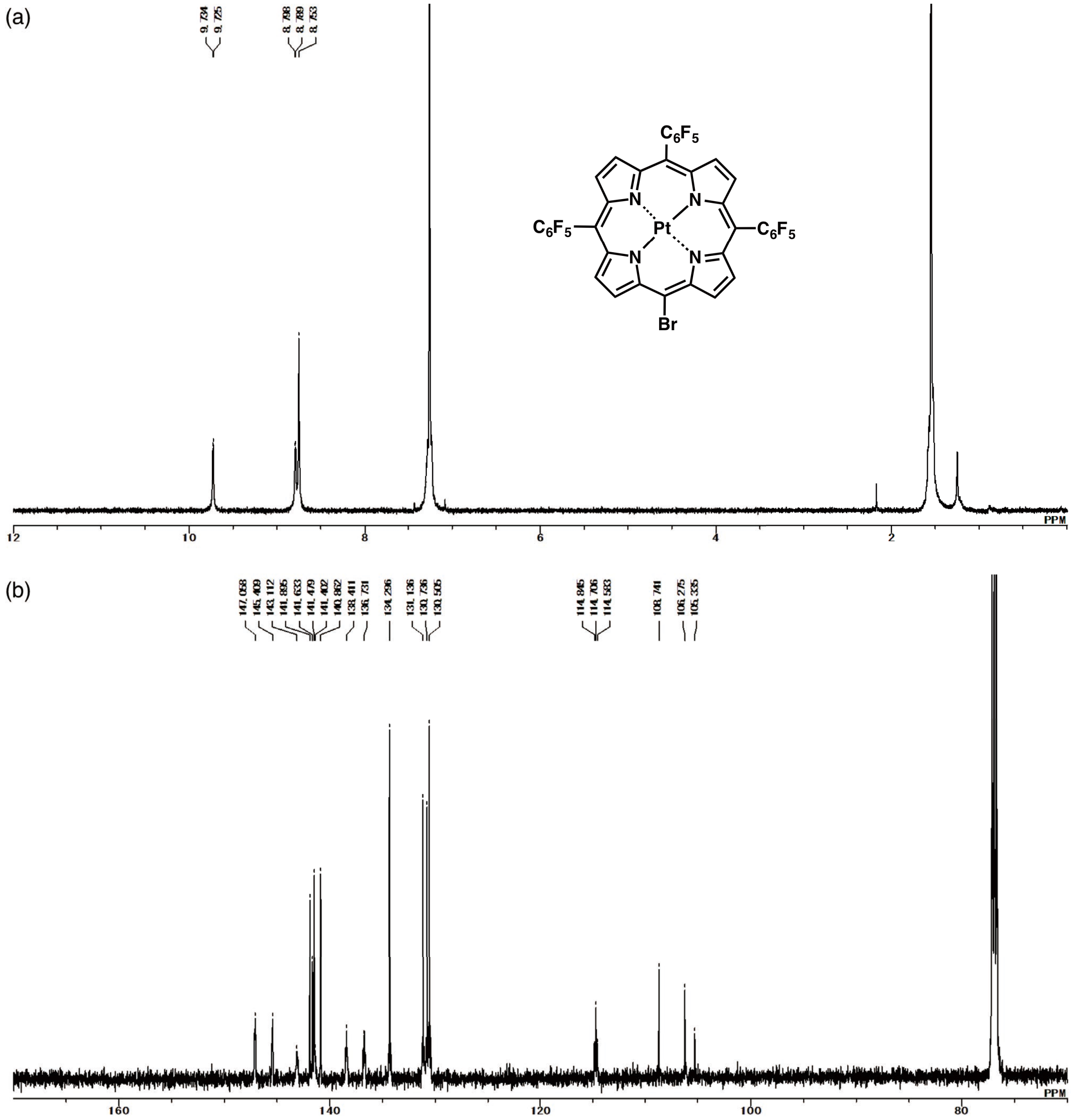

Fig. S2 (a) ^{1}H NMR and (b) ^{13}C NMR spectra of **1pt** in $CDCl_3$ at 20 °C.

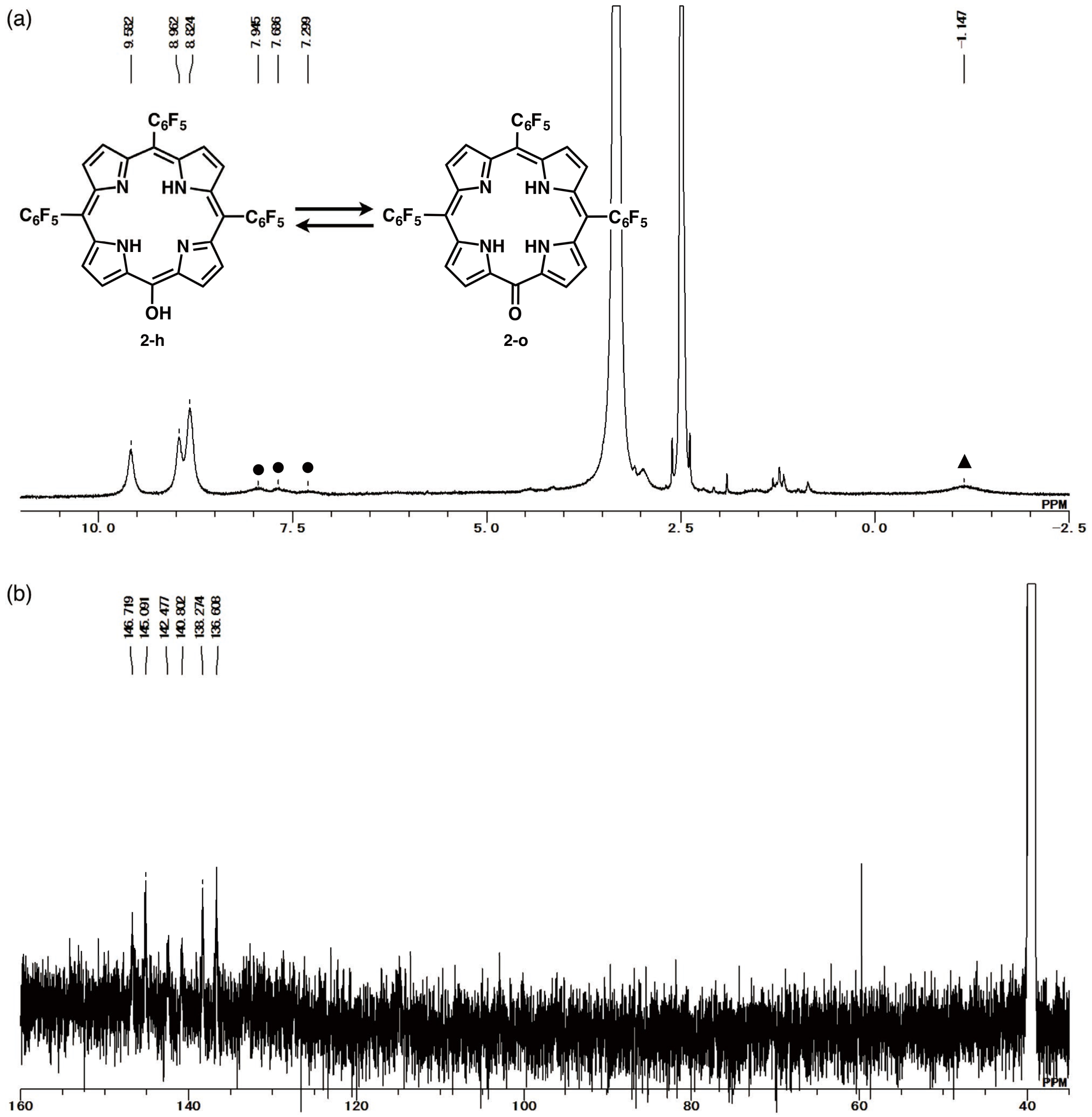

Fig. S3 (a) ^{1}H NMR and (b) ^{13}C NMR spectra of **2** in DMSO-d_6 at 20 °C. Signals labeled with triangle and circles are ascribable to hydroxyporphyrin-type tautomer **2-h** and oxophlorin-type tautomer **2-o**, respectively, in an equilibrium.

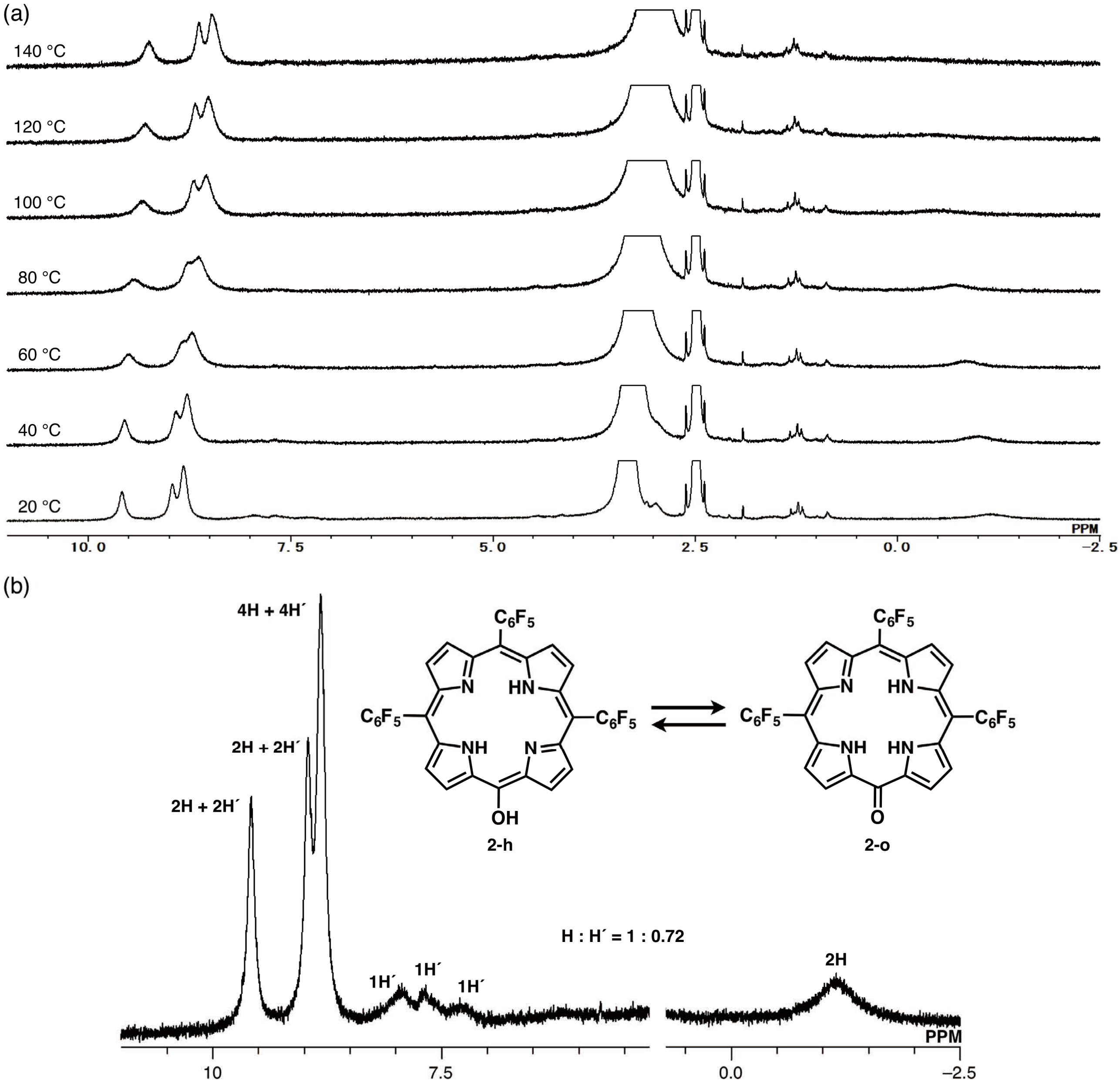

Fig. S4 (a) VT ^{1}H NMR spectral changes of **2** (2.0×10^{-3} M) in DMSO-d_6 at 20 to 140 °C and (b) enlarged view at 20 °C. The NH signals at −1.15 and 7.95–7.30 ppm at 20 °C could be assigned for **2-h** and **2-o**, respectively, whereas β-H signals of two tautomers were coalesced. The NH signals were separated with the ratio of 58:42 for **2-h** and **2-o** at 20 °C, and became broader at higher temperature.

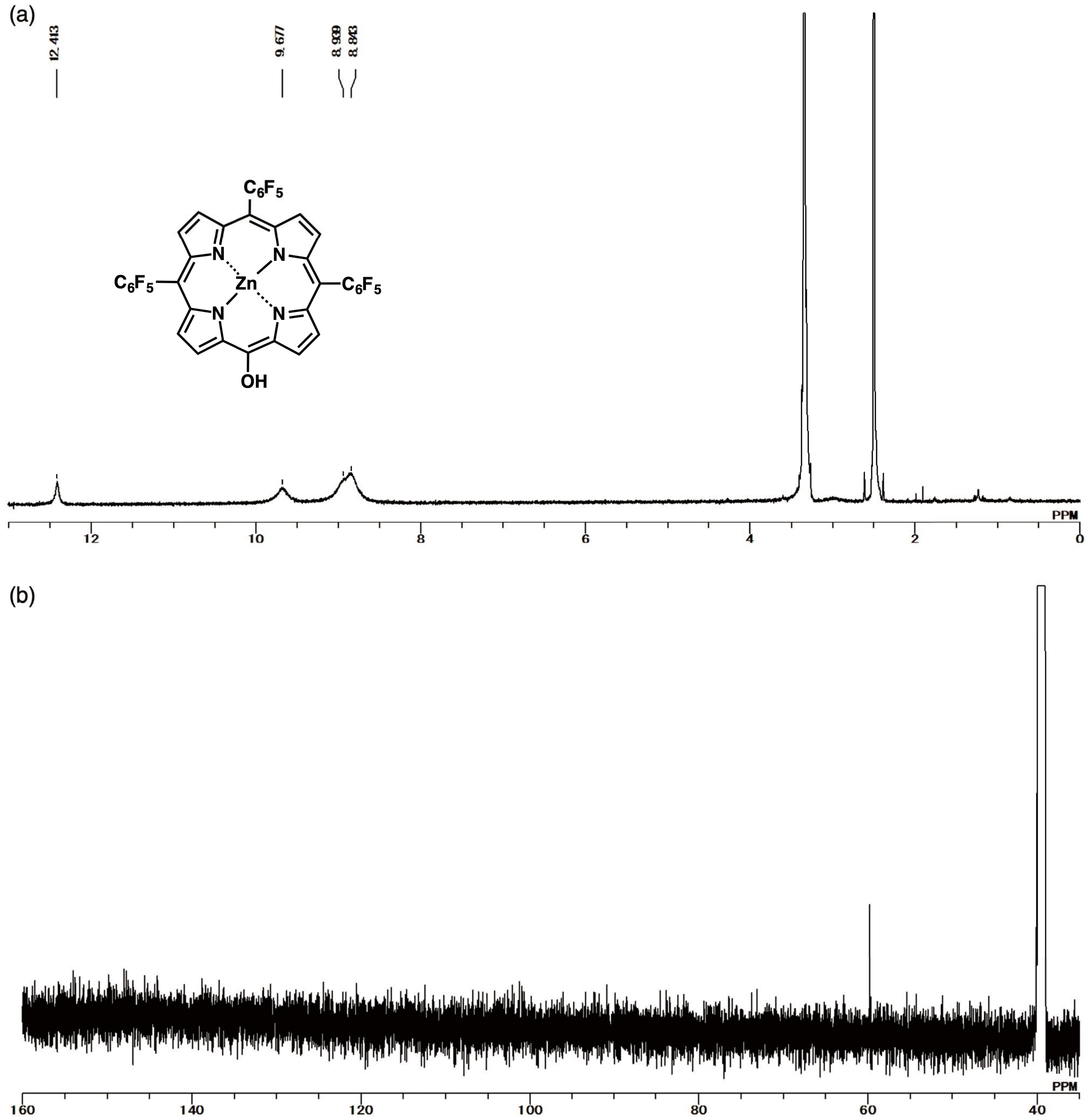

Fig. S5 (a) ^{1}H NMR and (b) ^{13}C NMR spectra of **2zn** in DMSO-d_6 at 20 °C. The ^{1}H NMR signals were broad in DMSO-d_6 at 140 °C, probably due to the presence of a small amount of radical species.

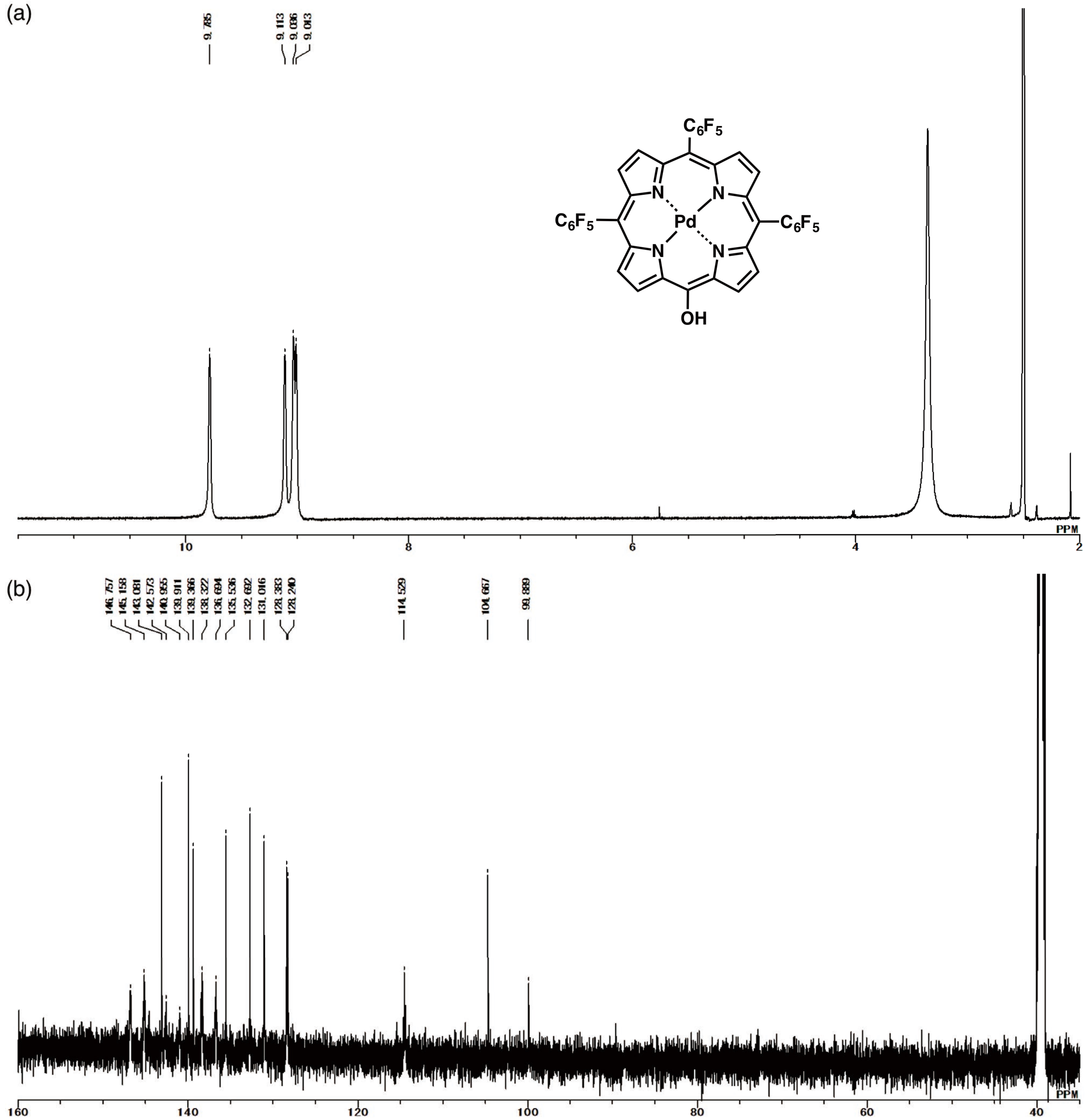

Fig. S6 (a) ^{1}H NMR and (b) ^{13}C NMR spectra of **2pd** in DMSO-d_6 at 20 °C.

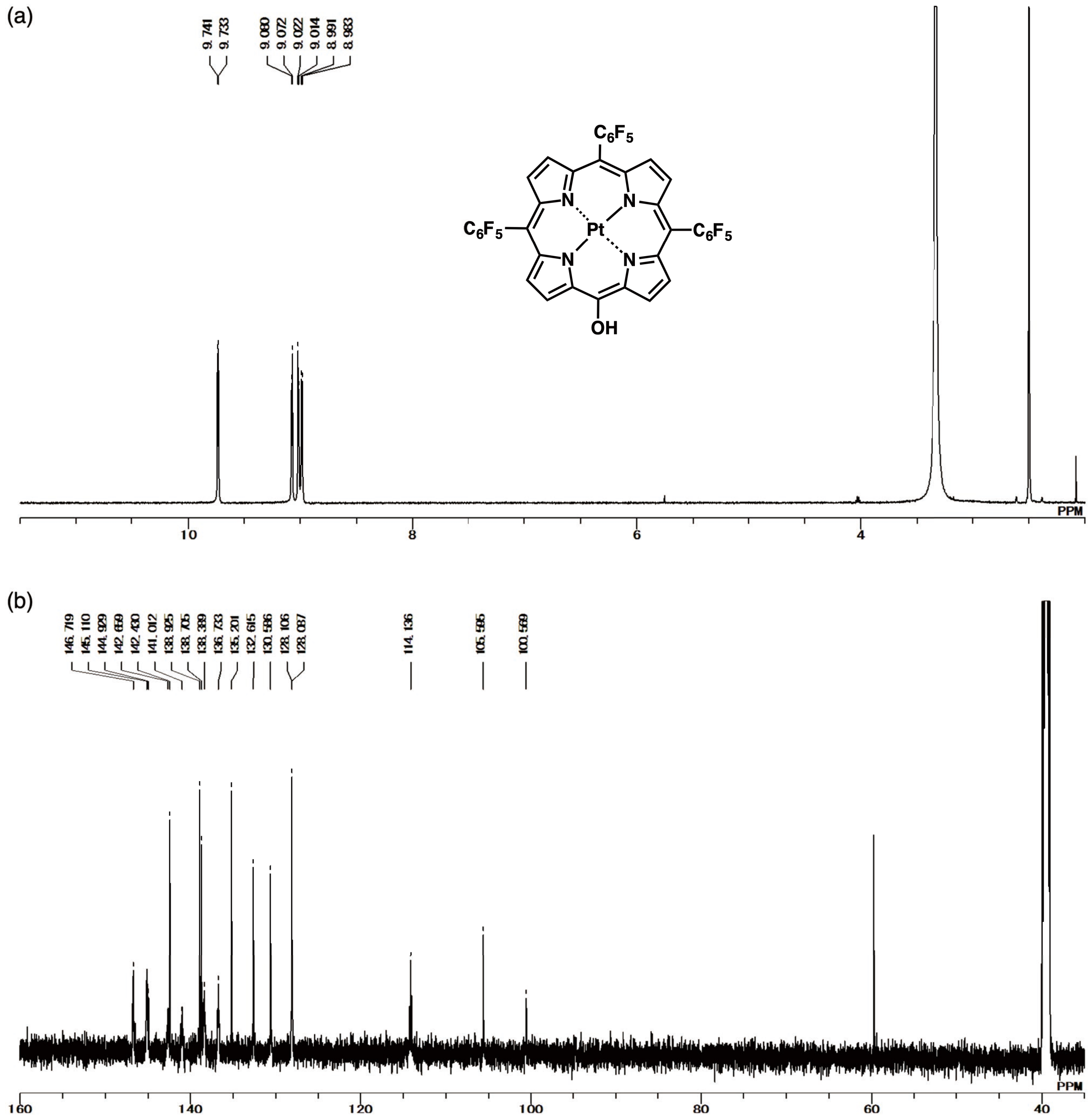

Fig. S7 (a) ^{1}H NMR and (b) ^{13}C NMR spectra of **2pt** in DMSO-d_6 at 20 °C.

2. Deprotonation behaviors

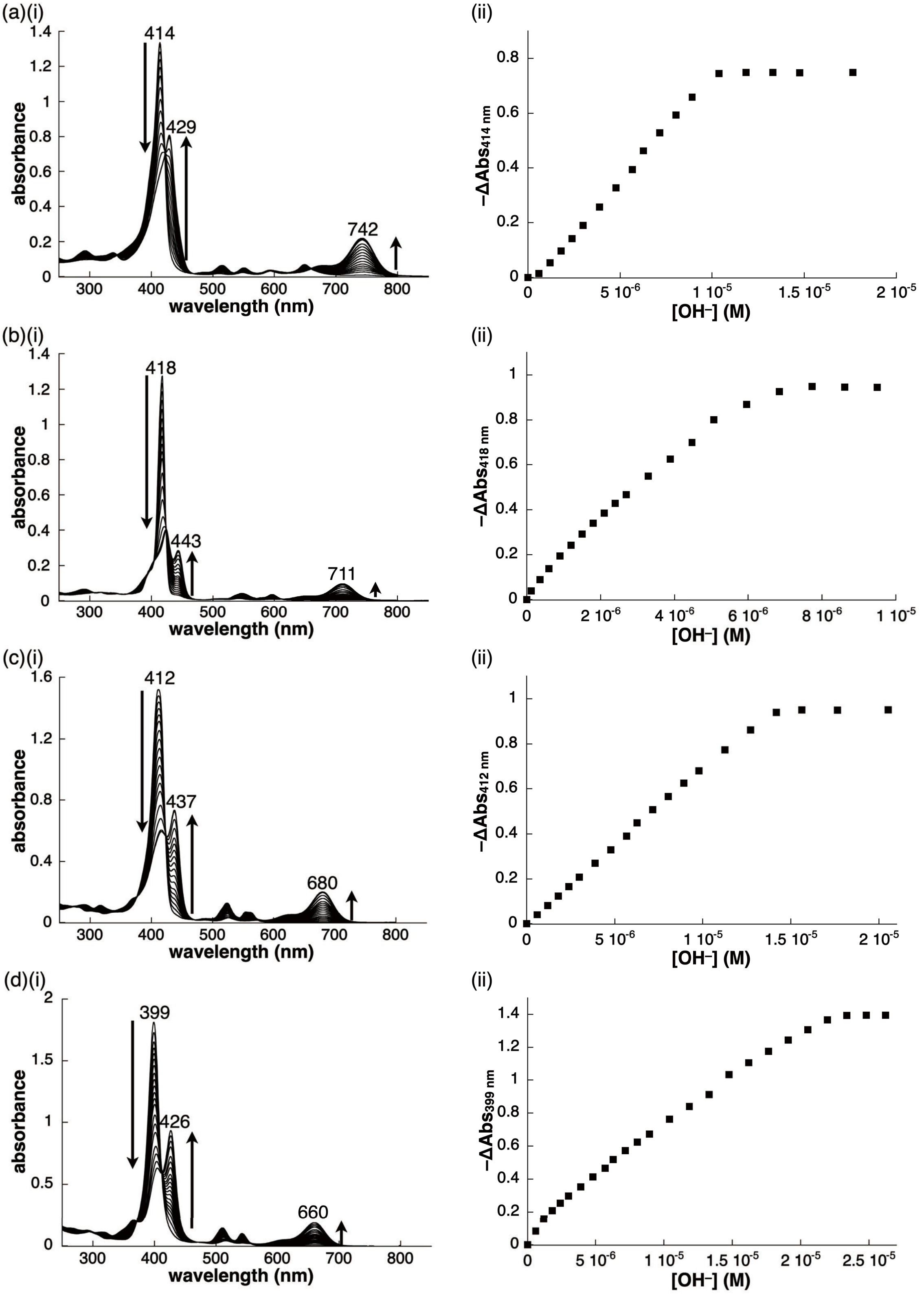

Fig. S8 Deprotonation behaviors of (a) **2** (1.0×10^{-5} M), (b) **2zn** (2.4×10^{-6} M), (c) **2pd** (7.1×10^{-6} M), and (d) **2pt** (1.0×10^{-5} M) in CH_2Cl_2 upon the addition of TBAOH: (i) UV/vis absorption spectral changes and (ii) corresponding titration plots. **2** showed the maximum spectral changes by adding 1 equiv of the base. On the other hand, **2zn** required an excess amount of the base for the deprotonation to exert UV/vis absorption spectral changes, suggesting the OH^- coordination to Zn^{II}. Furthermore, the requirement of an excess amount of OH^- for the deprotonation of **2pd** and **2pt** may be derived from the partial OH^- coordination to metal as seen in **2ni**.[S5b]

3. X-ray crystallographic data

Method for single-crystal X-ray analysis. Crystallographic data are summarized in Table S1. A single crystal of **2-o** was obtained by vapor diffusion of *n*-hexane into a $CHCl_3$ solution of **2**. The data crystal was a green prism of approximate dimensions 0.040 mm × 0.040 mm × 0.020 mm. A single crystal of **2zn**$^-$-TBA$^+$ was obtained by vapor diffusion of *n*-hexane into a CH_2ClCH_2Cl solution of the 1:1 mixture of **2zn** and TBAOH. The data crystal was a green prism of approximate dimensions 0.040 mm × 0.040 mm × 0.001 mm. A single crystal of **2pd**$^-$-TBA$^+$ was obtained by vapor diffusion of *n*-hexane into a CH_2Cl_2 solution of the 1:1 mixture of **2pd** and TBAOH. The data crystal was a green prism of approximate dimensions 0.100 mm × 0.015 mm × 0.010 mm. A single crystal of **2pt**$^-$-TBA$^+$ was obtained by vapor diffusion of *n*-hexane into a CH_2Cl_2 solution of the 1:1 mixture of **2pt** and TBAOH. The data crystal was a green prism of approximate dimensions 0.080 mm × 0.010 mm × 0.010 mm. A single crystal of **2**$^-$-TATA$^+$ was obtained by vapor diffusion of *n*-hexane into a CH_2Cl_2 solution of the 1:1 mixture of **2**$^-$ as a Na^+ salt, which was prepared by treating a CH_2Cl_2 solution of **2** with NaOH aq, and TATA$^+$-Cl^-[S3,4] upon washing with ion-exchanged water several times to remove NaCl. The data crystal was a red prism of approximate dimensions 0.010 mm × 0.010 mm × 0.010 mm. A single crystal of **2ni**$^-$-TATA$^+$ was obtained by vapor diffusion of H_2O into an CH_3CN solution of the 1:1 mixture of **2ni**$^-$ as a Na^+ salt, which was prepared by treating a CH_2Cl_2 solution of **2ni** with NaOH aq, and TATA$^+$-Cl^-[S3,4] upon washing with ion-exchanged water several times to remove NaCl. The data crystal was a red prism of approximate dimensions 0.060 mm × 0.010 mm × 0.010 mm. A single crystal of **2pd**$^-$-TATA$^+$ was obtained by vapor diffusion of *n*-hexane into an EtOAc solution of the 1:1 mixture of **2pd**$^-$ as a Na^+ salt, which was prepared by treating a CH_2Cl_2 solution of **2pd** with NaOH aq, and TATA$^+$-Cl^-[S3,4] upon washing with ion-exchanged water several times to remove NaCl. The data crystal was a yellow prism of approximate dimensions 0.120 mm × 0.005 mm × 0.005 mm. Data were collected at 90 K on a Rigaku Saturn 724 diffractometer with Si (111) monochromated synchrotron radiation (λ = 0.7829 Å for **2-o**, **2zn**$^-$-TBA$^+$, **2pt**$^-$-TBA$^+$, and **2**$^-$-TATA$^+$ and 0.78192 Å for **2pd**$^-$-TBA$^+$, **2ni**$^-$-TATA$^+$, and **2pd**$^-$-TATA$^+$) at BL40XU (SPring-8).[S7] All the structures were solved by dual-space method. The structures were refined by a full-matrix least-squares method by using a SHELXL 2014[S8] (Yadokari-XG).[S9] In each structure, the non-hydrogen atoms were refined anisotropically. CCDC 1906673–1906679 contain the supplementary crystallographic data for this paper. These data are provided free of charge from the Cambridge Crystallographic Data Centre via www.ccdc.cam.ac.uk/data_request/cif.

Table S1 Crystallographic details.

	**2-o**	**2zn**$^-$-TBA$^+$	**2pd**$^-$-TBA$^+$	**2pt**$^-$-TBA$^+$	**2**$^-$-TATA$^+$	**2ni**$^-$-TATA$^+$	**2pd**$^-$-TATA$^+$
formula	$C_{38}H_{11}F_{15}N_4O\cdot 0.23CHCl_3\cdot 0.13C_6H_{14}$	$C_{38}H_8F_{15}N_4OZn\cdot C_{16}H_{36}N\cdot 0.42C_6H_{14}$	$C_{38}H_8F_{15}N_4OPd\cdot C_{16}H_{36}N$	$C_{38}H_8F_{15}N_4OPt\cdot C_{16}H_{36}N$	$C_{38}H_{10}F_{15}N_4O\cdot C_{28}H_{30}N_3\cdot H_2O$	$C_{38}H_8F_{15}N_4ONi\cdot C_{28}H_{30}N_3\cdot CH_3CN$	$C_{38}H_8F_{15}N_4OPd\cdot C_{28}H_{30}N_3$
fw	862.51	1200.33	1170.34	1259.03	1250.06	1329.80	1336.43
crystal size, mm	0.040 × 0.040 × 0.001	0.040 × 0.040 × 0.020	0.100 × 0.015 × 0.010	0.080 × 0.010 × 0.010	0.010 × 0.010 × 0.010	0.060 × 0.010 × 0.010	0.120 × 0.005 × 0.005
crystal system	triclinic	triclinic	orthorhombic	orthorhombic	triclinic	monoclinic	monoclinic
space group	$P\bar{1}$ (no. 2)	$P\bar{1}$ (no. 2)	$P2_12_12_1$ (no. 19)	$P2_12_12_1$ (no. 19)	$P\bar{1}$ (no. 2)	$P2_1/c$ (no. 14)	$P2_1/c$ (no. 14)
a, Å	11.734(2)	15.303(3)	8.2881(3)	8.1911(16)	7.389(2)	8.4930(4)	8.8987(2)
b, Å	15.683(3)	16.985(3)	22.4879(6)	22.065(4)	13.345(4)	28.6109(13)	18.5720(5)
c, Å	18.431(3)	20.280(4)	26.3395(9)	25.559(5)	26.551(8)	22.8426(9)	32.3929(8)
α, °	84.631(3)	92.310(2)	90	90	103.059(4)	90	90
β, °	80.158(3)	94.162(2)	90	90	93.168(4)	96.787(3)	90.213(2)
γ, °	81.477(3)	101.368(2)	90	90	93.947(4)	90	90
V, Å^3	3297.0(11)	5146.2(17)	4909.2(3)	4619.5(16)	2537.6(14)	5511.7(4)	5353.4(2)
ρ_{calcd}, gcm^{-3}	1.738	1.549	1.583	1.810	1.633	1.603	1.658
Z	4	4	4	4	2	4	4
T, K	90(2)	90(2)	90(2)	90(2)	90(2)	90(2)	90(2)
μ, mm^{-1}	0.277	0.815	0.611	4.001	0.173	0.589	0.576
no. of reflns	27892	44052	27310	25047	20578	29443	22814
no. of unique reflns	11841	18560	8719	7915	9091	9787	9642
variables	1112	1425	691	529	814	840	814
λ, Å	0.7829	0.7829	0.78192	0.7829	0.7829	0.78192	0.78192
R_1 ($I > 2\sigma(I)$)	0.0699	0.0853	0.0641	0.0636	0.0629	0.0956	0.0878
wR_2 ($I > 2\sigma(I)$)	0.1810	0.2113	0.1539	0.1351	0.1427	0.1942	0.2039
GOF	1.136	1.196	1.101	1.127	1.055	1.060	0.966

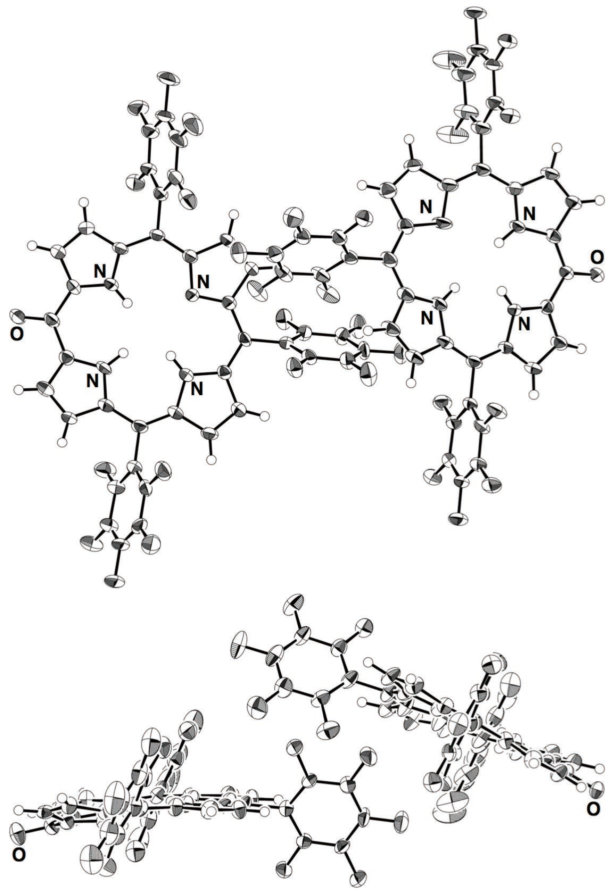

Fig. S9 Ortep drawings of single-crystal X-ray structure (top and side views) of **2-o**. Thermal ellipsoids are scaled to the 50% probability level.

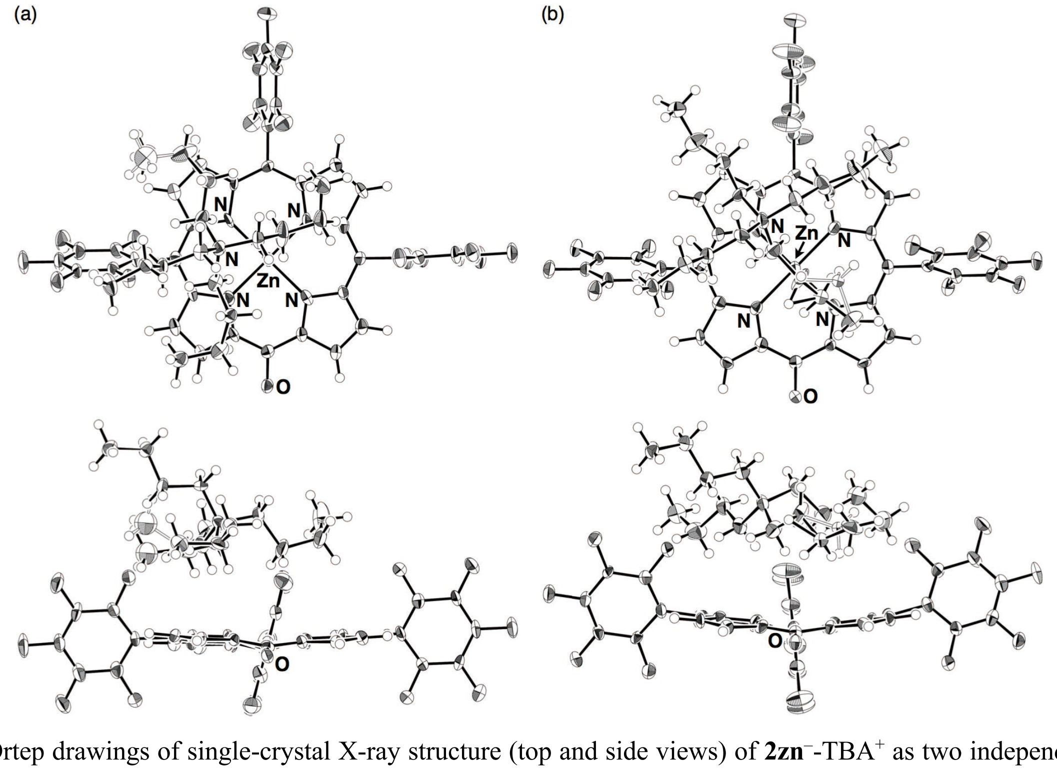

Fig. S10 Ortep drawings of single-crystal X-ray structure (top and side views) of **2zn**$^-$-TBA$^+$ as two independent structures ((a) and (b)). Thermal ellipsoids are scaled to the 50% probability level. TBA$^+$ has disordered structures in the ratios of 57 (black bond) : 43 (white bond) and 60 (black bond) : 40 (white bond) for (a) and (b), respectively.

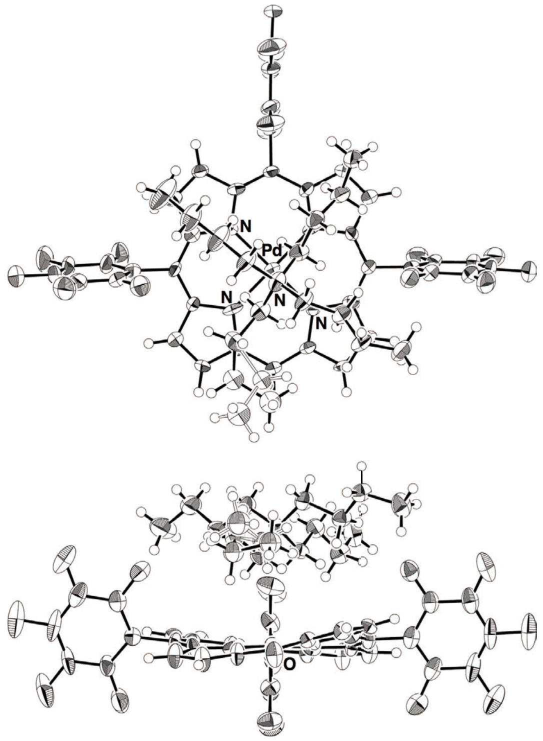

Fig. S11 Ortep drawings of single-crystal X-ray structure (top and side views) of **2pd**$^{-}$-TBA^{+}. Thermal ellipsoids are scaled to the 50% probability level. A butyl group of TBA^{+} has disordered structures in the ratio of 61 (black bond) : 39 (white bond). Flack parameter (0.178(16)) suggested the crystal including one enantiomer.

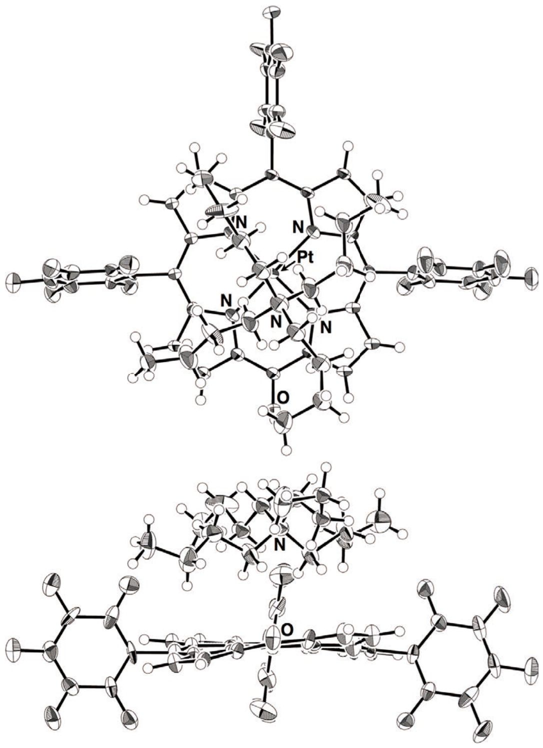

Fig. S12 Ortep drawings of single-crystal X-ray structure (top and side views) of **2pt**$^{-}$-TBA^{+}. Thermal ellipsoids are scaled to the 50% probability level. Flack parameter (0.475(6)) suggested the racemic twin crystals.

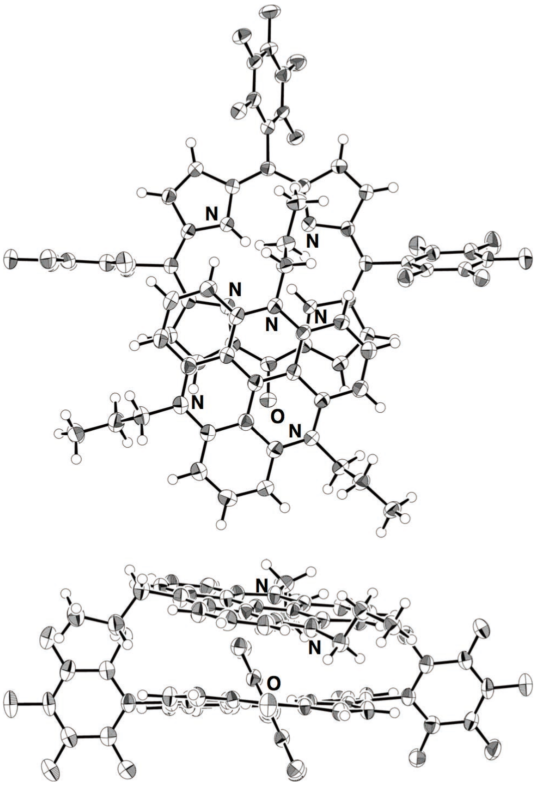

Fig. S13 Ortep drawings of single-crystal X-ray structure (top and side views) of **2**⁻-TATA^{+}. Thermal ellipsoids are scaled to the 50% probability level.

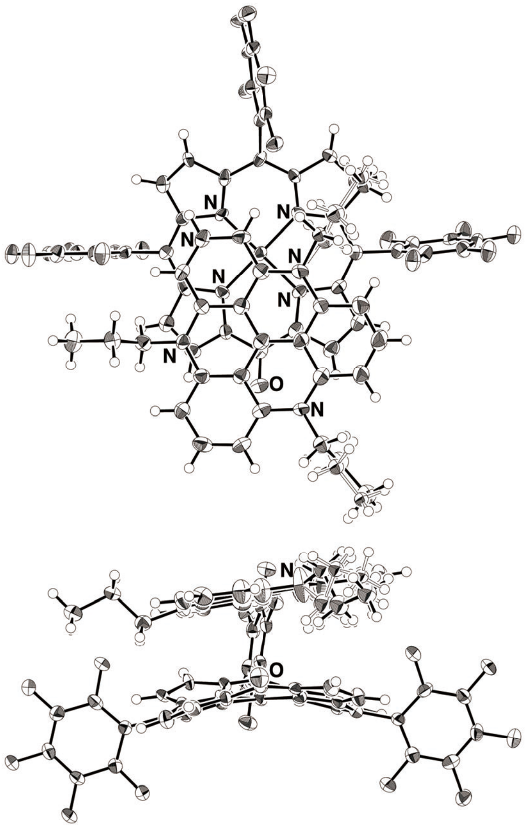

Fig. S14 Ortep drawings of single-crystal X-ray structure (top and side views) of **2ni**⁻-TATA^{+}. Thermal ellipsoids are scaled to the 50% probability level. A propyl group of TATA^{+} has disordered structures in the ratio of 63 (black bond) : 37 (white bond).

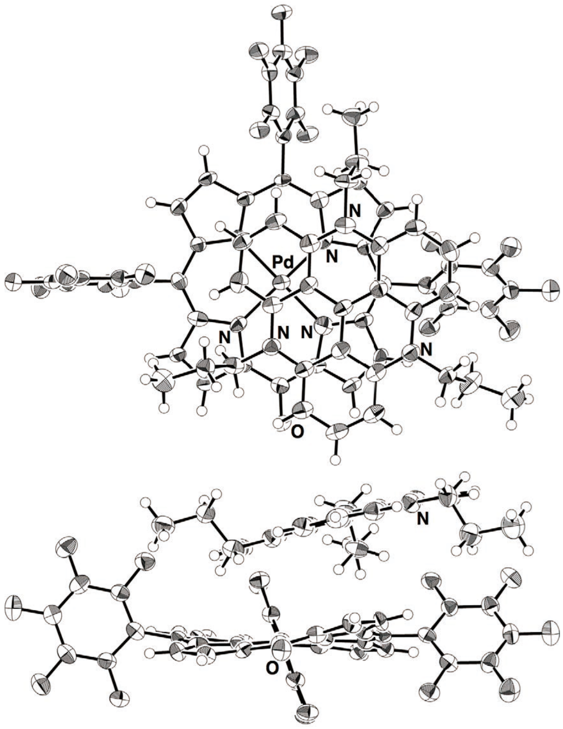

Fig. S15 Ortep drawings of single-crystal X-ray structure (top and side views) of **2pd**$^-$-TATA$^+$. Thermal ellipsoids are scaled to the 50% probability level.

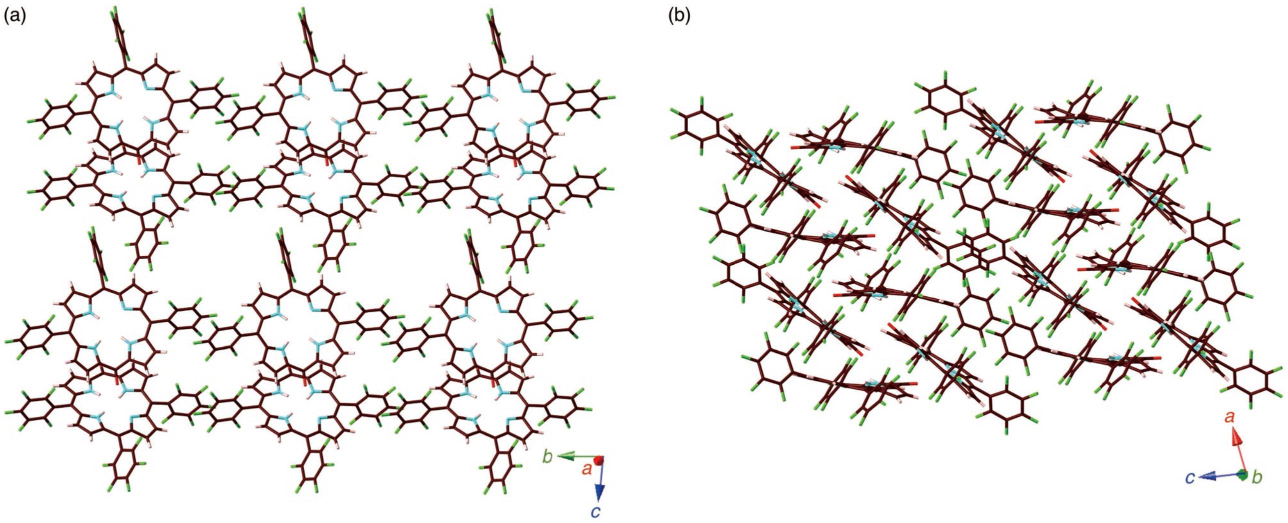

Fig. S16 Packing diagrams (stacking assemblies) of **2-o** as (a) top and (b) side views. Atom color code: brown, pink, cyan, red, and green refer to carbon, hydrogen, nitrogen, oxygen, and fluorine, respectively.

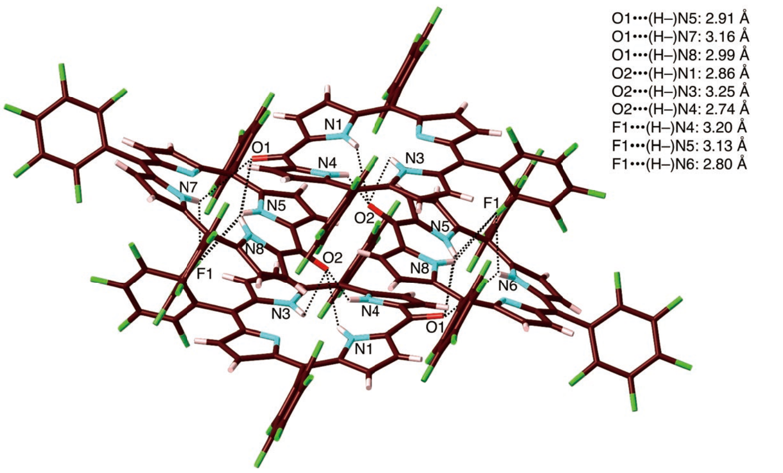

Fig. S17 Packing diagram of **2-o**. Intermolecular hydrogen-bonding O···(H–)N distances between O atom and inner NH were 2.91, 3.16, 2.99, 2.86, 3.25, and 2.74 Å. Hydrogen-bonding interactions were also observed between F atom of C_6F_5 group and inner NH with F···(H–)N distances of 3.20, 3.13, and 2.80 Å. Atom color code: brown, pink, cyan, red, and green refer to carbon, hydrogen, nitrogen, oxygen, and fluorine, respectively.

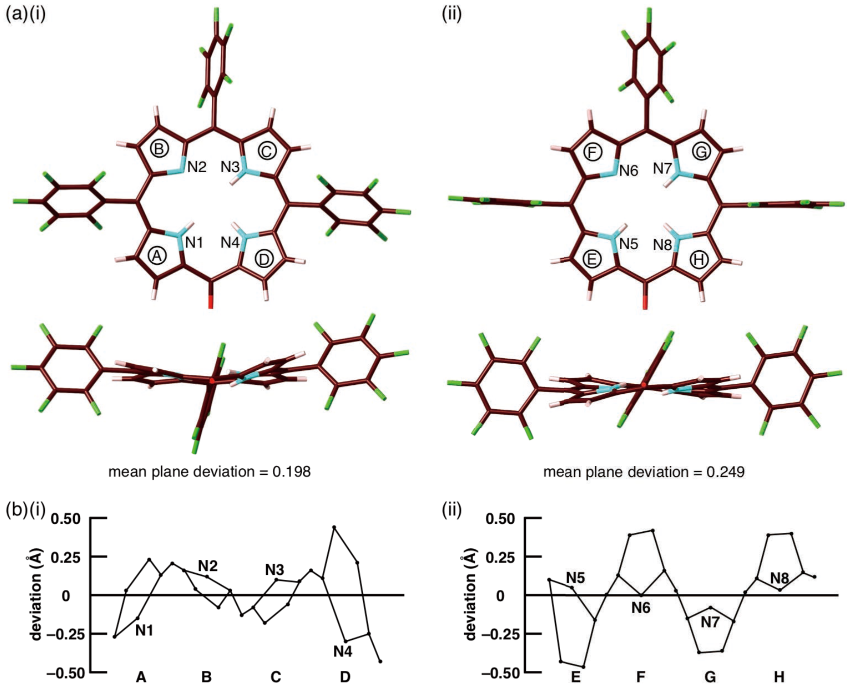

Fig. S18 (a) Top and side views of crystal structures of **2-o** as two independent structures ((i) and (ii)) with mean-plane deviations (Å) of porphyrin 24-atom planes and (b) linear displays of the deviations (Å) from the plane of 24 atoms for two independent structures. Atom color code: brown, pink, cyan, red, and green refer to carbon, hydrogen, nitrogen, oxygen, and fluorine, respectively.

Table S2 Dihedral angles (°) between A, B, C, and D rings (planes of five atoms of pyrrole units) and those between E, F, G, and H rings (planes of five atoms of pyrrole units) of **2-o** (the structure in Fig. S18a(ii)).

	B	C	D
A	14.2	14.8	32.0
B	–	0.9	19.6
C	–	–	19.5

	F	G	H
E	17.5	21.8	17.4
F	–	13.9	22.2
G	–	–	13.7

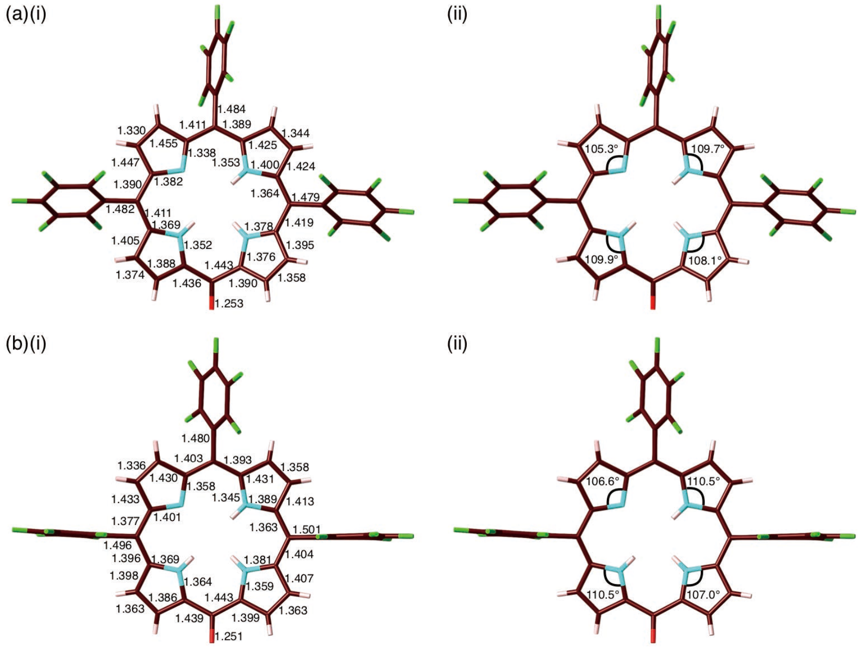

Fig. S19 Two independent structures ((a) and (b)) of **2-o** with (i) bond lengths (Å) and (ii) C–N–C angles. The C–N–C angles of the structure in (a) were 109.9°, 105.3°, 109.7°, and 108.1°, and the distance between O atom and *meso*-C atom was 1.253 Å. The C–N–C angles of the structure in (b) were 110.5°, 106.6°, 110.5°, and 107.0°, and the distance between O atom and *meso*-C atom was 1.251 Å. These angles and distances suggested the oxophlorin-type structures. Atom color code: brown, pink, cyan, red, and green refer to carbon, hydrogen, nitrogen, oxygen, and fluorine, respectively.

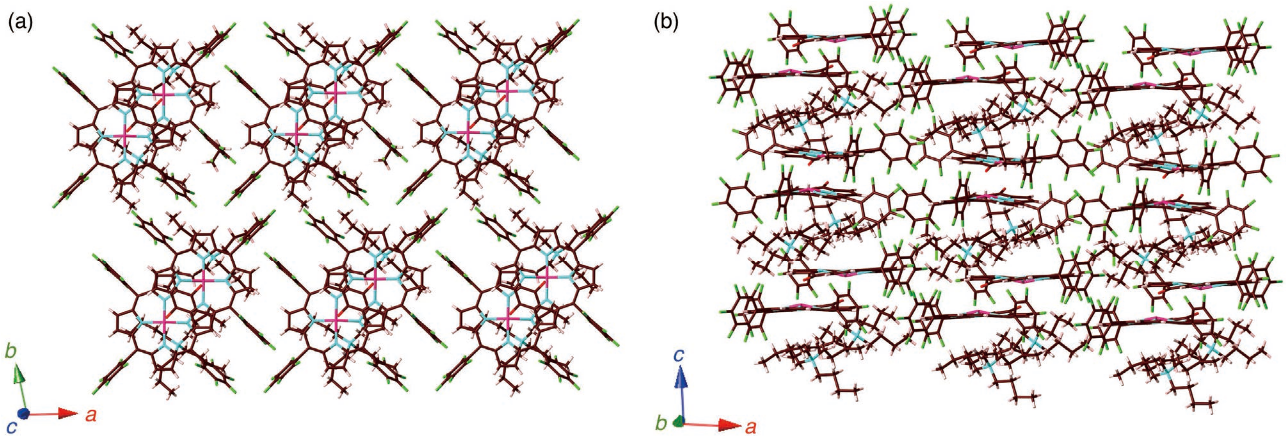

Fig. S20 Packing diagrams (stacking assemblies) of **2zn**$^{-}$-TBA^{+} as (a) top and (b) side views, which show the charge-by-charge assembly mode, wherein **2zn**$^{-}$ formed the dimer through coordination bond between Zn atom and anionic O atom. Atom color code: brown, pink, cyan, red, green, and magenta refer to carbon, hydrogen, nitrogen, oxygen, fluorine, and zinc, respectively.

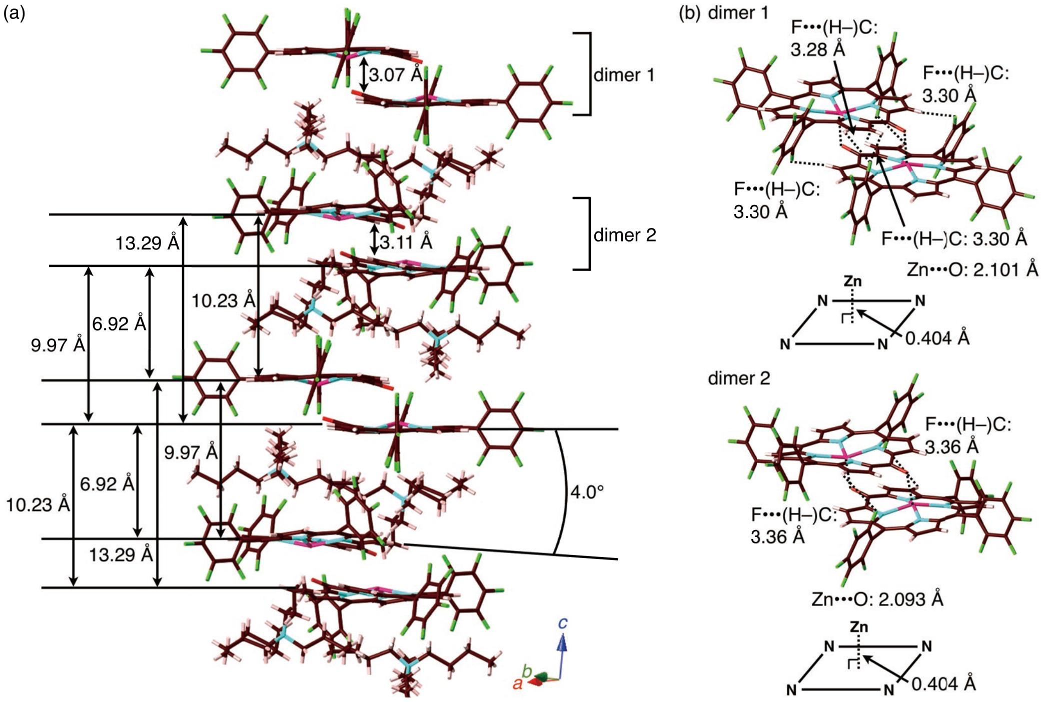

Fig. S21 Packing diagrams of **2zn**$^{-}$-TBA^{+} as (a) side view of charge-by-charge column and (b) dimers of laterally located anions. The distances between two **2zn**$^{-}$ (24-atom plane) were 10.23, 6.92, 13.29, and 9.97 Å. The dihedral angle between two **2zn**$^{-}$ (24-atom plane) was 4.0°. The **2zn**$^{-}$ dimers (dimer 1 and dimer 2) were formed through coordination bond between Zn atom and anionic O atom, wherein the distance between Zn and O were 2.101 and 2.093 Å for dimer 1 and dimer 2, respectively. The distance between two **2zn**$^{-}$ (11-atom plane of dipyrrin unit) were 3.07 and 3.11 Å for dimer 1 and dimer 2, respectively. Dimer 1 showed intermolecular hydrogen bonding between F of C_6F_5 group and porphyrin-β-CH with F···(H–)C distances of 3.28 and 3.30 Å. Dimer 2 also showed intermolecular hydrogen bonding with an F···(H–)C distance of 3.36 Å. Both dimer 1 and dimer 2 showed the distance of 0.404 Å between Zn atom and plane of four N atoms. Atom color code: brown, pink, cyan, red, green, and magenta refer to carbon, hydrogen, nitrogen, oxygen, fluorine, and zinc, respectively.

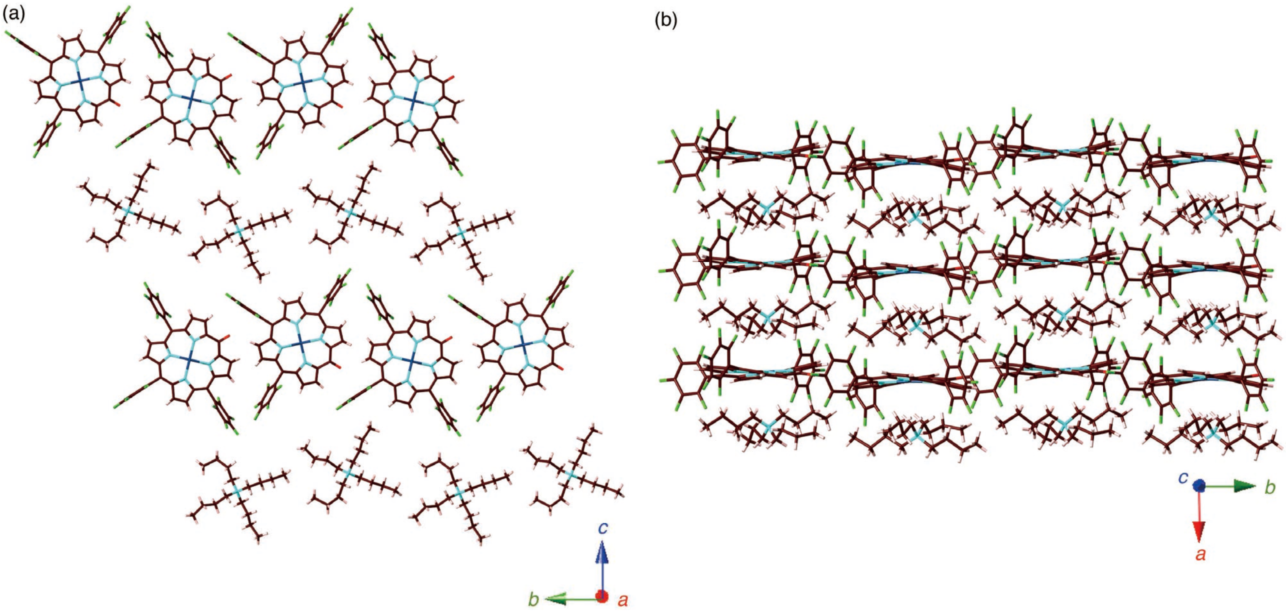

Fig. S22 Packing diagrams (stacking assemblies) of **2pd**$^{-}$-TBA^{+} as (a) top and (b) side views, which show the charge-by-charge assembly mode. Atom color code: brown, pink, cyan, red, green, and blue refer to carbon, hydrogen, nitrogen, oxygen, fluorine, and palladium, respectively.

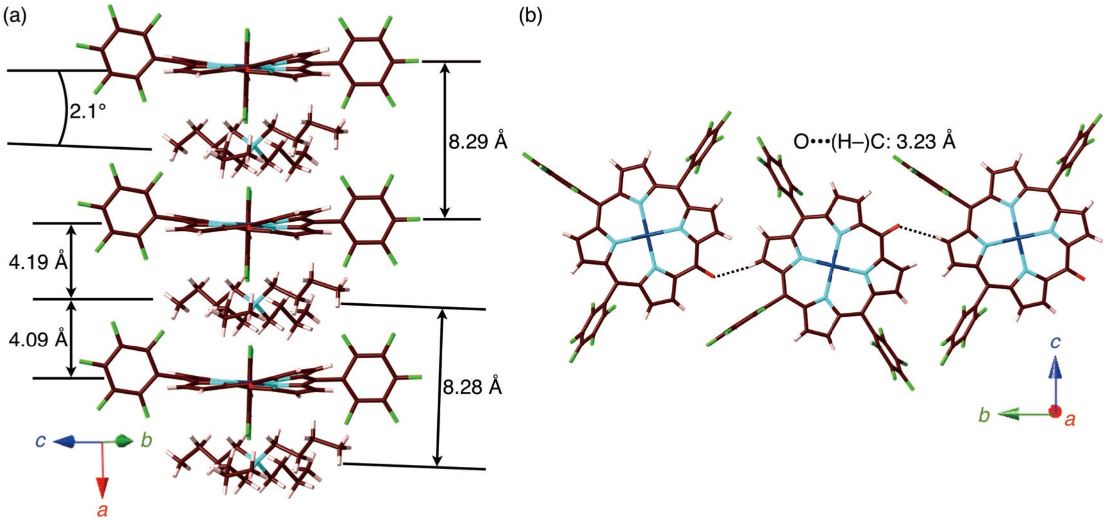

Fig. S23 Packing diagrams of **2pd**$^{-}$-TBA^{+} as (a) side view of charge-by-charge column and (b) top view of laterally located anions. The distance between two **2pd**$^{-}$ (24-atom plane) was 8.29 Å and that between two TBA^{+} (17-atom plane) was 8.28 Å. The dihedral angle between **2pd**$^{-}$ (24-atom plane) and TBA^{+} (17-atom plane) was 2.1°. The distances between **2pd**$^{-}$ (24-atom plane) and TBA^{+} (17-atom plane) were 4.09 and 4.19 Å. Intermolecular hydrogen-bonding O···(H–)C distance between anionic O atom and porphyrin-β-CH was 3.23 Å. The plane of TBA^{+} was defined by one N atom and 16 C atoms. Atom color code: brown, pink, cyan, red, green, and blue refer to carbon, hydrogen, nitrogen, oxygen, fluorine, and palladium, respectively.

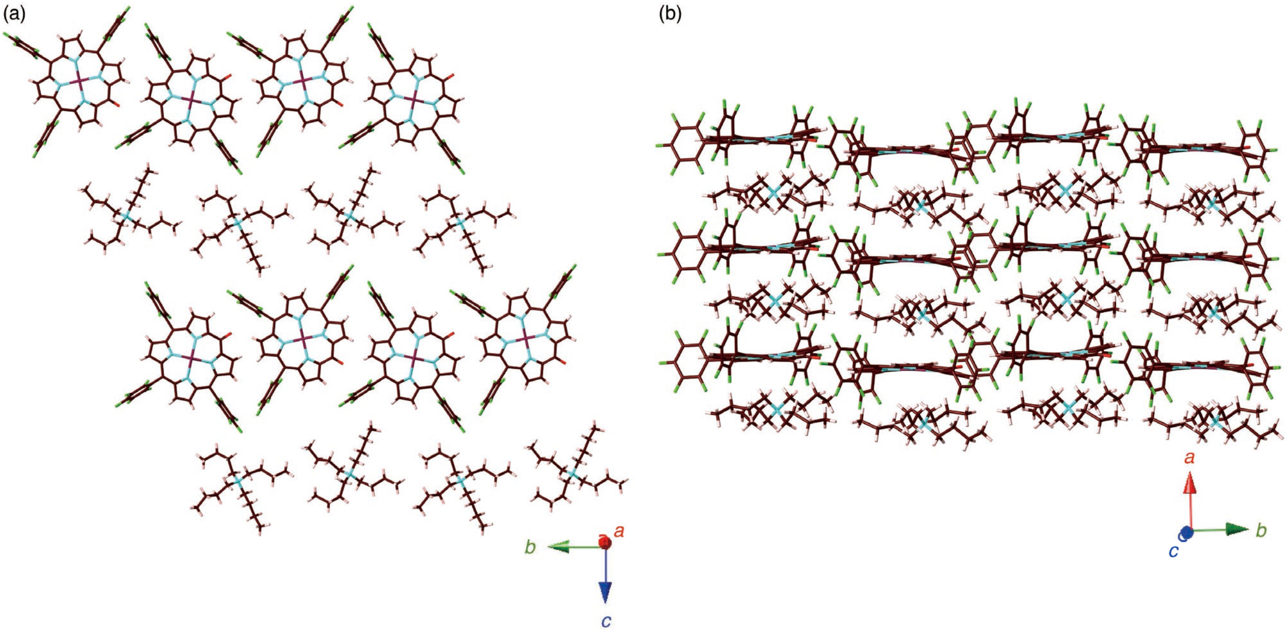

Fig. S24 Packing diagrams (stacking assemblies) of **2pt**$^{-}$-TBA^{+} as (a) top and (b) side views, which show the charge-by-charge assembly mode. Atom color code: brown, pink, cyan, red, green, and purple refer to carbon, hydrogen, nitrogen, oxygen, fluorine, and platinum, respectively.

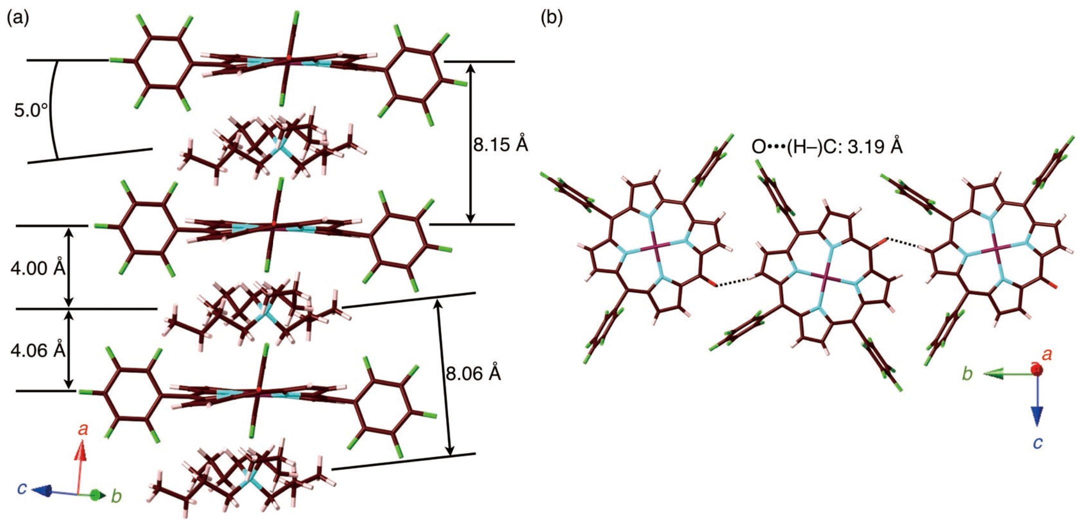

Fig. S25 Packing diagrams of **2pt**$^{-}$-TBA^{+} as (a) side view of charge-by-charge column and (b) top view of laterally located anions. The distance between two **2pt**$^{-}$ (24-atom plane) was 8.15 Å and that between two TBA^{+} (17-atom plane) was 8.06 Å. The dihedral angle between **2pt**$^{-}$ (24-atom plane) and TBA^{+} (17-atom plane) was 5.0°. The distances between **2pt**$^{-}$ (24-atom plane) and TBA^{+} (17-atom plane) were 4.00 and 4.06 Å. Intermolecular hydrogen-bonding O···(H–)C distance between anionic O atom and porphyrin-β-CH was 3.12 Å. The plane of TBA^{+} was defined by one N atom and 16 C atoms. Atom color code: brown, pink, cyan, red, green, and purple refer to carbon, hydrogen, nitrogen, oxygen, fluorine, and platinum, respectively.

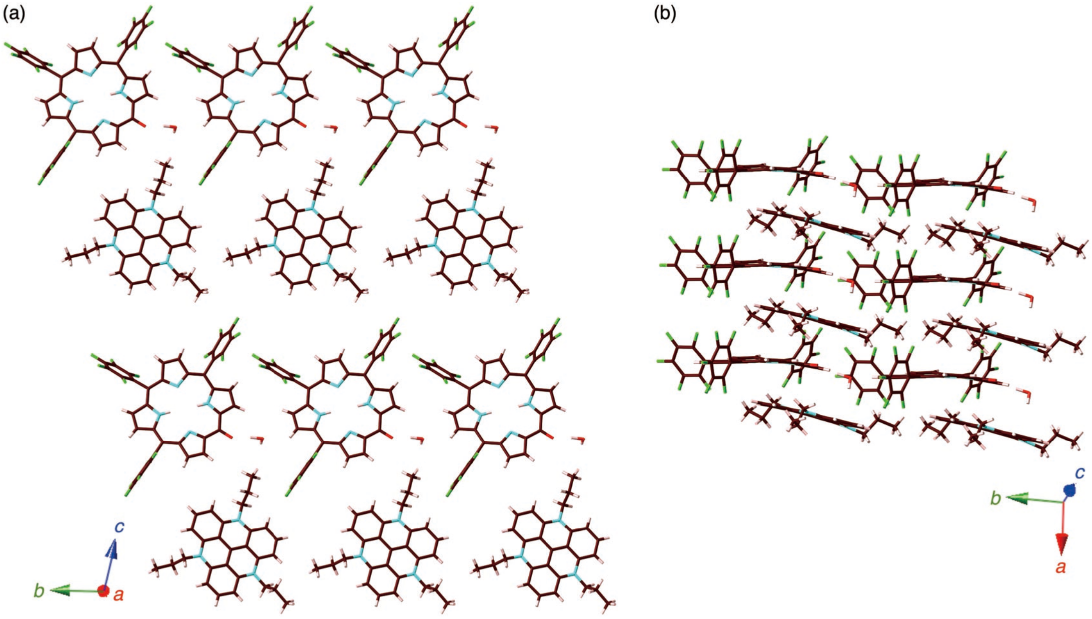

Fig. S26 Packing diagrams (stacking assemblies) of **2**$^{-}$-TATA^{+} as (a) top and (b) side views, which show the charge-by-charge assembly mode. Atom color code: brown, pink, cyan, red, and green refer to carbon, hydrogen, nitrogen, oxygen, and fluorine, respectively.

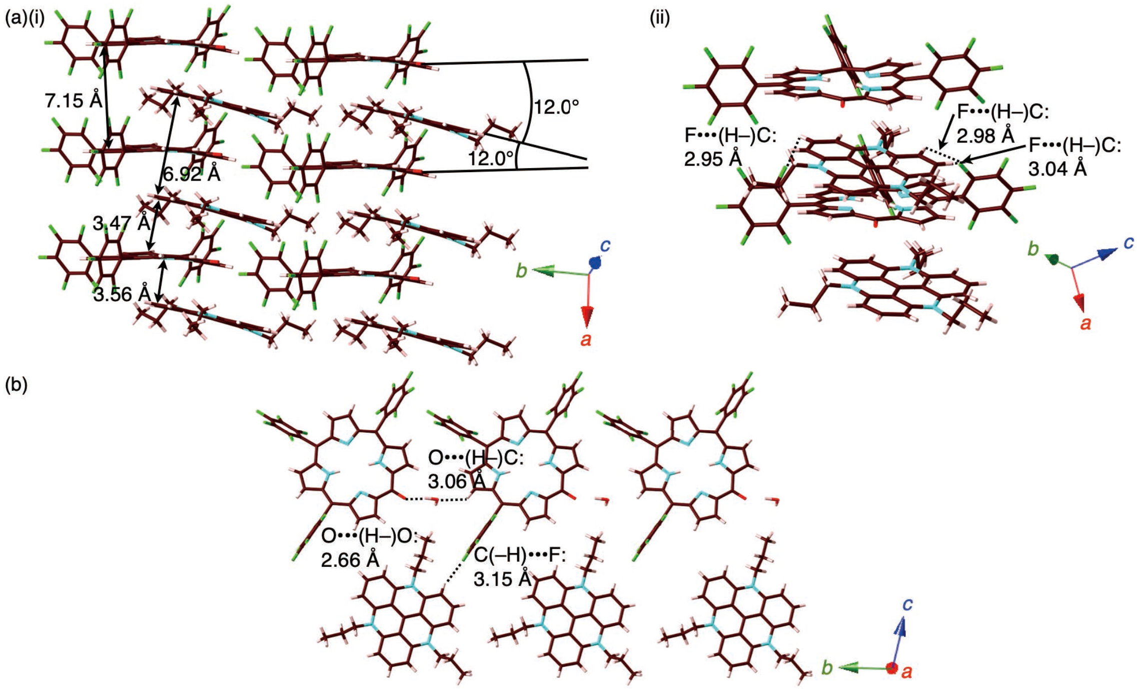

Fig. S27 Packing diagrams of $\mathbf{2}^-$-TATA$^+$ showing (a) side and (b) top views. The distance between two $\mathbf{2}^-$ (24-atom plane) was 7.15 Å and that between two TATA$^+$ (22-atom plane) was 6.92 Å. The dihedral angle between $\mathbf{2}^-$ (24-atom plane) and TATA$^+$ (22-atom plane) was 12.0°. Packing structure was formed through ${}^i\pi$–${}^i\pi$ interactions and hydrogen bonding. The distances between $\mathbf{2}^-$ (11-atom plane of dipyrrin unit) and TATA$^+$ (14-atom plane of acridine unit) were 3.47 and 3.56 Å. Furthermore, intermolecular hydrogen-bonding F···(H–)C distances between F of C_6F_5 group and CH of TATA$^+$ were 2.95, 2.98, 3.04, and 3.15 Å. Hydrogen-bonding interactions were also observed between porphyrin-O and water and between porphyrin-β-CH and water with an O···(H–)O distance of 2.66 Å and a C(–H)···O distance of 3.06 Å, respectively. Atom color code: brown, pink, cyan, red, and green refer to carbon, hydrogen, nitrogen, oxygen, and fluorine, respectively.

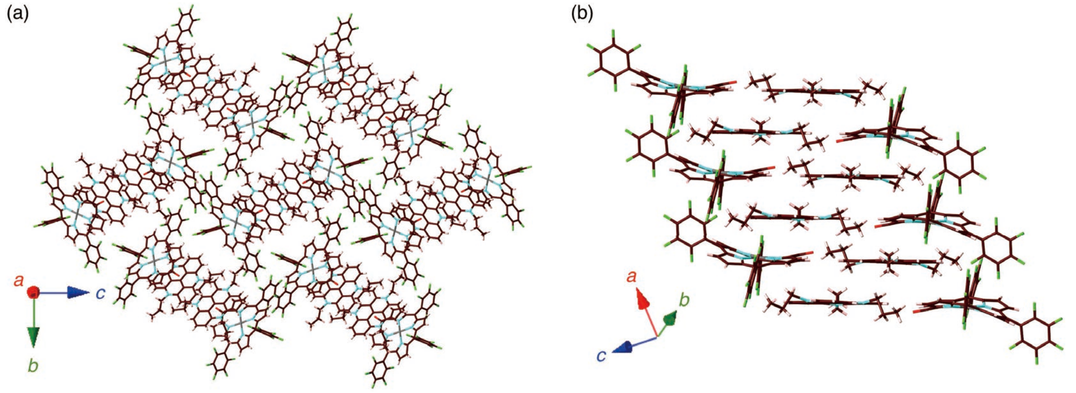

Fig. S28 Packing diagrams (stacking assemblies) of $\mathbf{2ni}^-$-TATA$^+$ as (a) top and (b) side views, which show the contribution of both charge-by-charge and charge-segregated modes. Atom color code: brown, pink, cyan, red, green, and gray refer to carbon, hydrogen, nitrogen, oxygen, fluorine, and nickel, respectively.

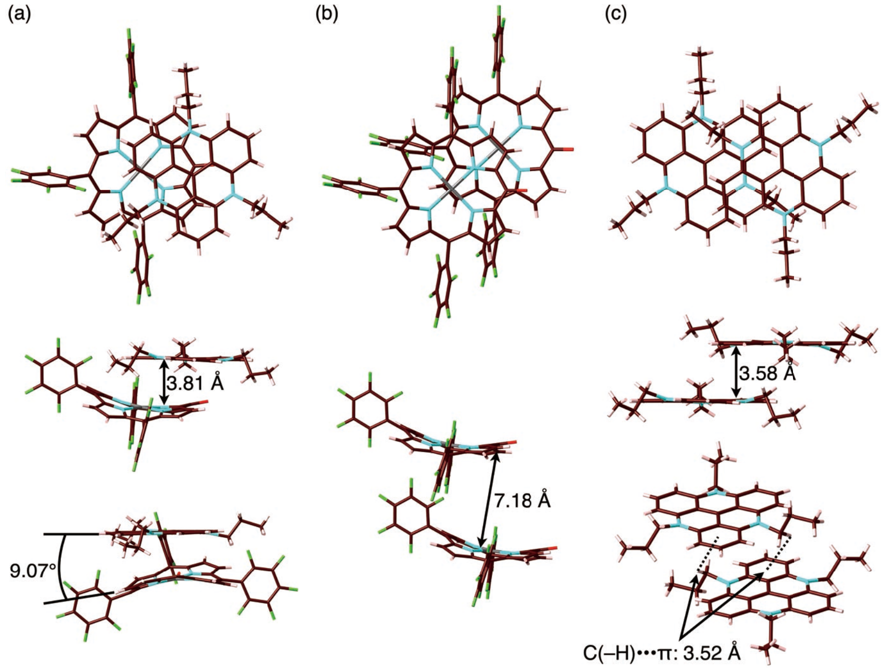

Fig. S29 Packing diagrams of **2ni**$^{-}$-TATA^{+} showing top and side views of (a) ion pair, (b) pair of anions, and (c) pair of cations. The distance and dihedral angle between **2ni**$^{-}$ (24-atom plane) and TATA^{+} (22-atom plane) were 3.81 Å and 9.07°, respectively. The distance between two **2ni**$^{-}$ (24-atom plane) was 7.18 Å and that between two TATA^{+} (22-atom plane) was 3.58 Å. Furthermore, the C–H···π distance between π-plane and alkyl units of TATA^{+} was 3.52 Å. Atom color code: brown, pink, cyan, red, green, and gray refer to carbon, hydrogen, nitrogen, oxygen, fluorine, and nickel, respectively.

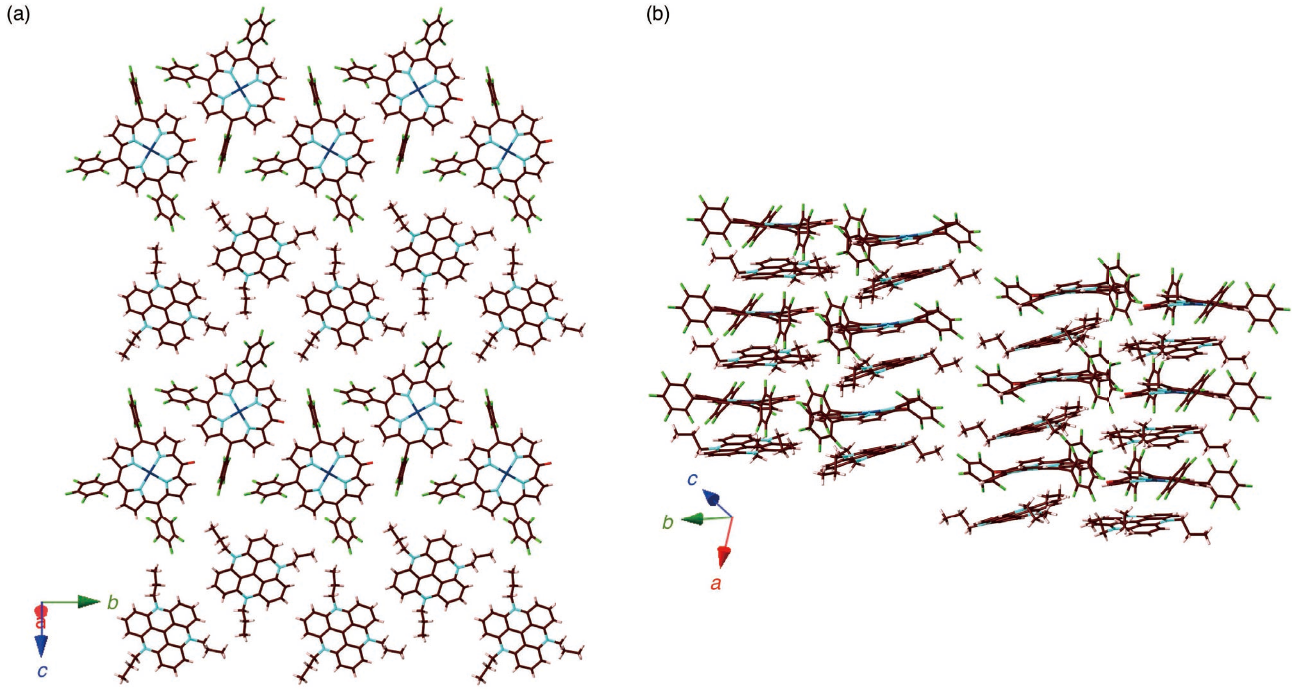

Fig. S30 Packing diagrams (stacking assemblies) of **2pd**$^{-}$-TATA^{+} as (a) top and (b) side views, which show the charge-by-charge assembly mode. Atom color code: brown, pink, cyan, red, green, and blue refer to carbon, hydrogen, nitrogen, oxygen, fluorine, and palladium, respectively.

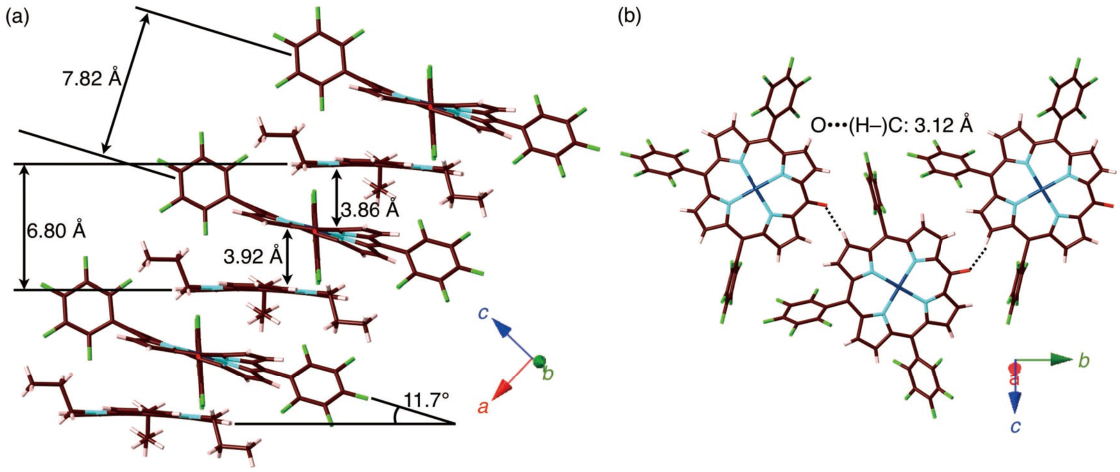

Fig. S31 Packing diagrams of **2pd**$^{-}$-TATA^{+} as (a) side view of charge-by-charge column and (b) top view of laterally located anions. The distance between two **2pd**$^{-}$ (24-atom plane) was 7.82 Å and that between two TATA^{+} (22-atom plane) was 6.80 Å. The dihedral angle between **2pd**$^{-}$ (24-atom plane) and TATA^{+} (22-atom plane) was 11.7°. Packing structure was formed through $^{i}\pi$–$^{i}\pi$ interactions and hydrogen bonding. The distances between **2pd**$^{-}$ (11-atom plane of dipyrrin unit) and TATA^{+} (22-atom plane) were 3.86 and 3.92 Å. Furthermore, intermolecular hydrogen-bonding O···(H–)C distance between anionic O atom and porphyrin-β-CH was 3.12 Å. Atom color code: brown, pink, cyan, red, green, and blue refer to carbon, hydrogen, nitrogen, oxygen, fluorine, and palladium, respectively.

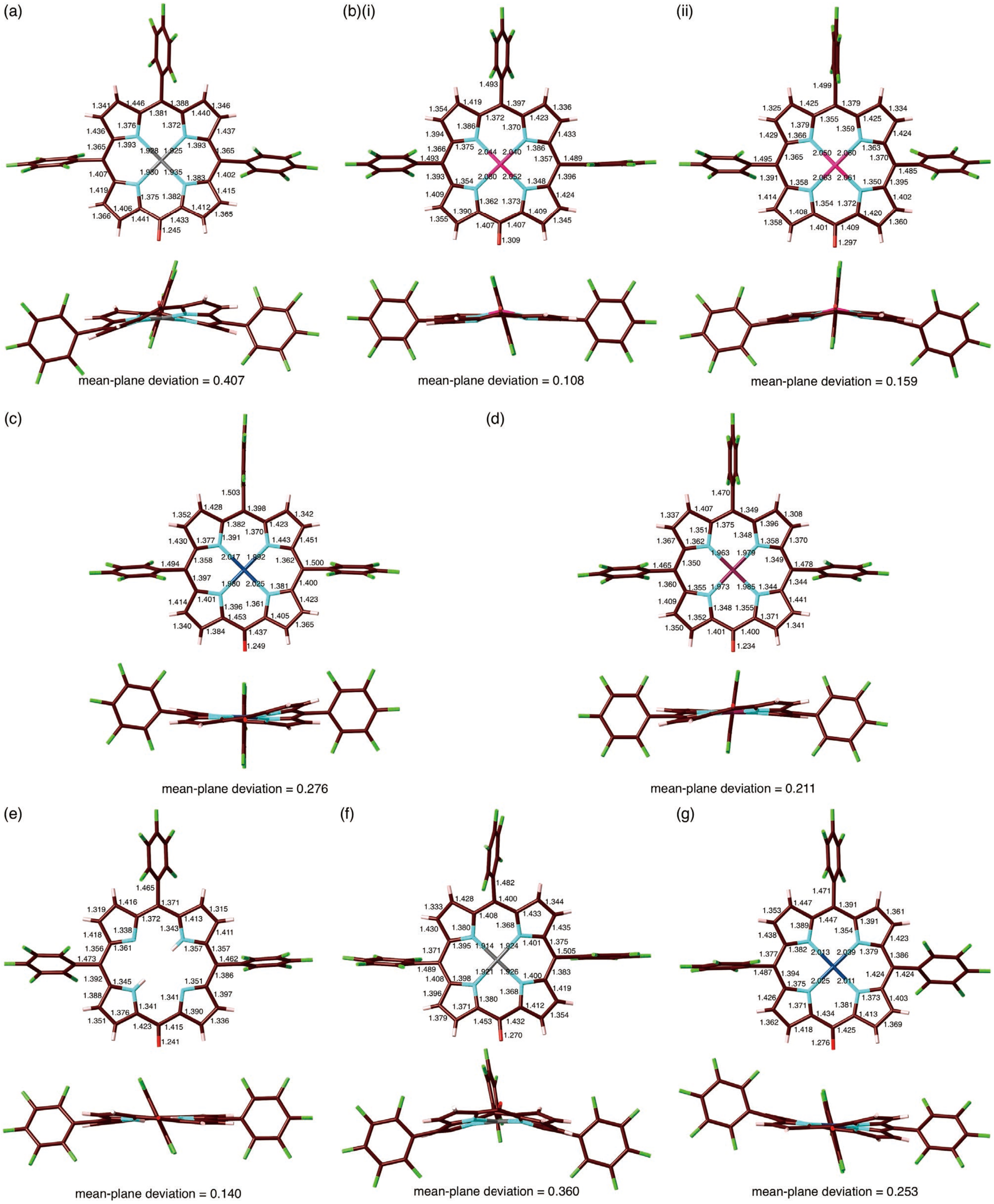

Fig. S32 Top and side views of crystal structures of porphyrin anions in (a) **2ni**$^{-}$-TBA^{+},[S5b] (b) **2zn**$^{-}$-TBA^{+} (two independent structures ((i) and (ii))), (c) **2pd**$^{-}$-TBA^{+}, (d) **2pt**$^{-}$-TBA^{+}, (e) **2**$^{-}$-TATA^{+}, (f) **2ni**$^{-}$-TATA^{+}, and (g) **2pd**$^{-}$-TATA^{+} with bond lengths (Å) and mean-plane deviations (Å) of porphyrin 24-atom planes. Atom color code: brown, pink, cyan, red, green, gray, magenta, blue, and purple refer to carbon, hydrogen, nitrogen, oxygen, fluorine, nickel, zinc, palladium, and platinum, respectively.

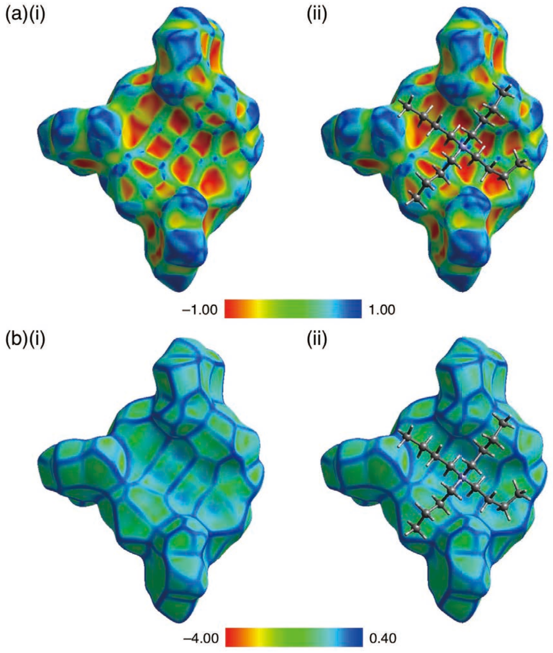

Fig. S33 Hirshfeld surface[S10] of **2ni**$^{-}$ in the crystal structure of **2ni**$^{-}$-TBA^{+}[S5b] mapped over (a) shape-index property and (b) curvedness property: (i) only surface and (ii) surface with a ball-and-stick model of the neighboring TBA^{+}. Shape index is a qualitative measure of shape and is sensitive to subtle changes in surface shape, particularly in a flat region by differing by sign represent complementary bumps (blue) and hollows (red), whereas curvedness is a function of the root-mean-square curvature of the surface, and maps of curvedness typically show large regions of green (relatively flat) separated by dark blue edges (large positive curvature). The surface of **2ni**$^{-}$ mapped over shape-index property exhibited depressions above the π-planes, suggesting that the stacking structures were stabilized by CH–π interaction. Atom color code: gray, white, and blue refer to carbon, hydrogen, and nitrogen, respectively.

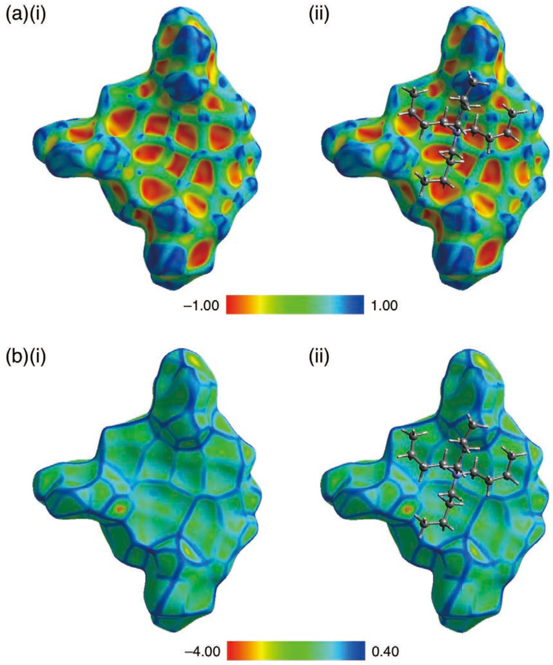

Fig. S34 Hirshfeld surface[S10] of representative **2zn**$^{-}$ in the crystal structure of **2zn**$^{-}$-TBA^{+} (Fig. S20,21) mapped over (a) shape-index property and (b) curvedness property: (i) only surface and (ii) surface with a ball-and-stick model of the neighboring TBA^{+}. Shape index is a qualitative measure of shape and is sensitive to subtle changes in surface shape, particularly in a flat region by differing by sign represent complementary bumps (blue) and hollows (red), whereas curvedness is a function of the root-mean-square curvature of the surface, and maps of curvedness typically show large regions of green (relatively flat) separated by dark blue edges (large positive curvature). The surface of **2zn**$^{-}$ mapped over shape-index property exhibited depressions above the π-planes, suggesting that the stacking structures were stabilized by CH–π interaction. Atom color code: gray, white, and blue refer to carbon, hydrogen, and nitrogen, respectively.

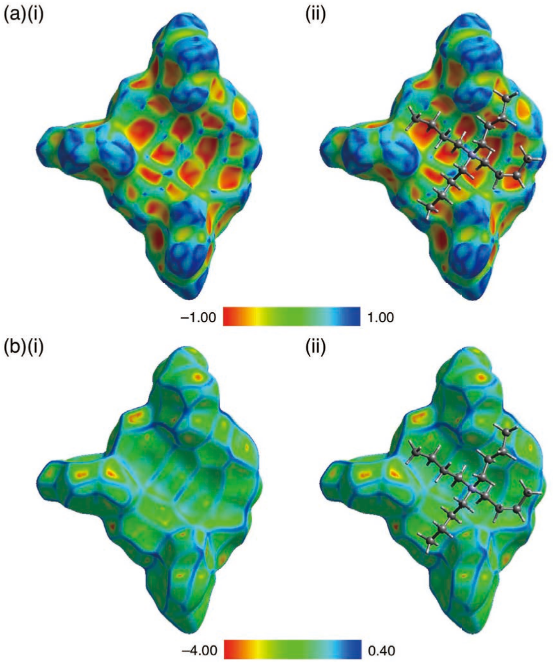

Fig. S35 Hirshfeld surface[S10] of **2pd**$^-$ in the crystal structure of **2pd**$^-$-TBA$^+$ (Fig. S22,23) mapped over (a) shape-index property and (b) curvedness property: (i) only surface and (ii) surface with a ball-and-stick model of the neighboring TBA$^+$. Shape index is a qualitative measure of shape and is sensitive to subtle changes in surface shape, particularly in a flat region by differing by sign represent complementary bumps (blue) and hollows (red), whereas curvedness is a function of the root-mean-square curvature of the surface, and maps of curvedness typically show large regions of green (relatively flat) separated by dark blue edges (large positive curvature). The surface of **2pd**$^-$ mapped over shape-index property exhibited depressions above the π-planes, suggesting that the stacking structures were stabilized by CH–π interaction. Atom color code: gray, white, and blue refer to carbon, hydrogen, and nitrogen, respectively.

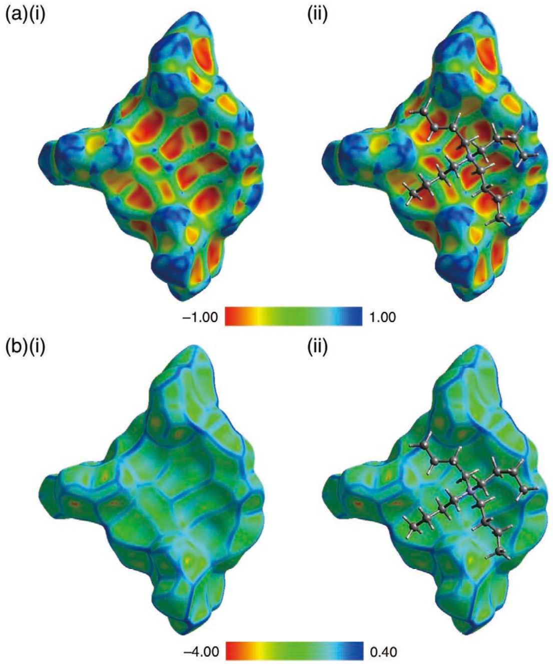

Fig. S36 Hirshfeld surface[S10] of **2pt**$^-$ in the crystal structure of **2pt**$^-$-TBA$^+$ (Fig. S24,25) mapped over (a) shape-index property and (b) curvedness property: (i) only surface and (ii) surface with a ball-and-stick model of the neighboring TBA$^+$. Shape index is a qualitative measure of shape and is sensitive to subtle changes in surface shape, particularly in a flat region by differing by sign represent complementary bumps (blue) and hollows (red), whereas curvedness is a function of the root-mean-square curvature of the surface, and maps of curvedness typically show large regions of green (relatively flat) separated by dark blue edges (large positive curvature). The surface of **2pt**$^-$ mapped over shape-index property exhibited depressions above the π-planes, suggesting that the stacking structures were stabilized by CH–π interaction. Atom color code: gray, white, and blue refer to carbon, hydrogen, and nitrogen, respectively.

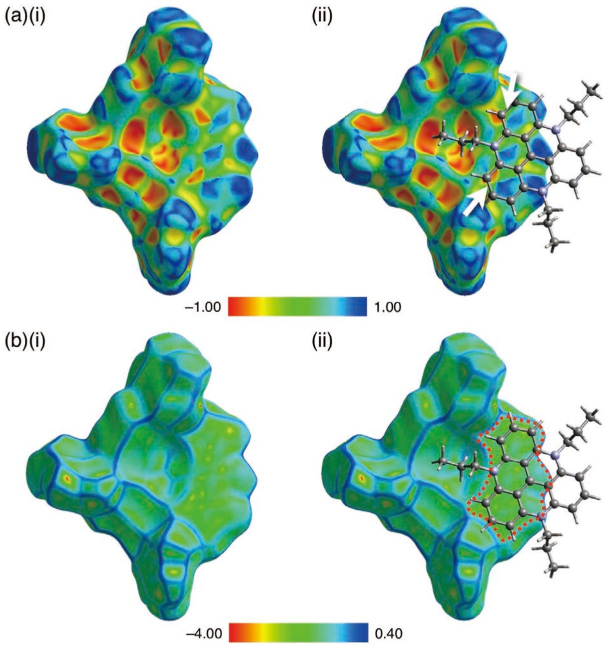

Fig. S37 Hirshfeld surface[S10] of **2**$^-$ in the crystal structure of **2**$^-$-TATA$^+$ (Fig. S26,27) mapped over (a) shape-index property and (b) curvedness property: (i) only surface and (ii) surface with a ball-and-stick model of the neighboring TATA$^+$. Shape index is a qualitative measure of shape and is sensitive to subtle changes in surface shape, particularly in a flat region by differing by sign represent complementary bumps (blue) and hollows (red), whereas curvedness is a function of the root-mean-square curvature of the surface, and maps of curvedness typically show large regions of green (relatively flat) separated by dark blue edges (large positive curvature). The surfaces of **2**$^-$ showed the red and blue triangles arranged in bow-tie shapes (indicated by white arrows in (a)) on the shape-index surface and flat region (indicated by red dashed area in (b)) on the curvedness surface, indicating the characteristic mapping pattern for $^i\pi$–$^i\pi$ stacking of **2**$^-$ and the core part of TATA$^+$.[S11] Atom color code: gray, white, and blue refer to carbon, hydrogen, and nitrogen, respectively.

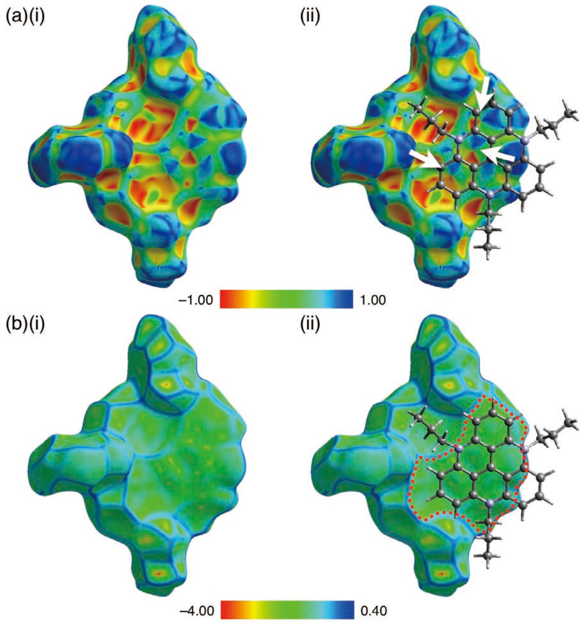

Fig. S38 Hirshfeld surface[S10] of **2ni**$^-$ in the crystal structure of **2ni**$^-$-TATA$^+$ (Fig. S28,29) mapped over (a) shape-index property and (b) curvedness property: (i) only surface and (ii) surface with a ball-and-stick model of the neighboring TATA$^+$. Shape index is a qualitative measure of shape and is sensitive to subtle changes in surface shape, particularly in a flat region by differing by sign represent complementary bumps (blue) and hollows (red), whereas curvedness is a function of the root-mean-square curvature of the surface, and maps of curvedness typically show large regions of green (relatively flat) separated by dark blue edges (large positive curvature). The surfaces of **2ni**$^-$ showed the red and blue triangles arranged in bow-tie shapes (indicated by white arrows in (a)) on the shape-index surface and flat region (indicated by red dashed area in (b)) on the curvedness surface, indicating the characteristic mapping pattern for $^i\pi$–$^i\pi$ stacking of **2ni**$^-$ and the core part of TATA$^+$.[S11] Atom color code: gray, white, and blue refer to carbon, hydrogen, and nitrogen, respectively.

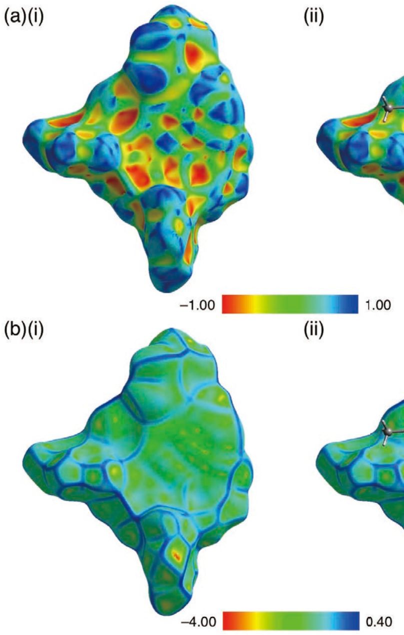

Fig. S39 Hirshfeld surface[S10] of **2pd**$^{-}$ in the crystal structure of **2pd**$^{-}$-TATA^{+} (Fig. S30,31) mapped over (a) shape-index property and (b) curvedness property: (i) only surface and (ii) surface with a ball-and-stick model of the neighboring TATA^{+}. Shape index is a qualitative measure of shape and is sensitive to subtle changes in surface shape, particularly in a flat region by differing by sign represent complementary bumps (blue) and hollows (red), whereas curvedness is a function of the root-mean-square curvature of the surface, and maps of curvedness typically show large regions of green (relatively flat) separated by dark blue edges (large positive curvature). The surfaces of **2pd**$^{-}$ showed the red and blue triangles arranged in bow-tie shapes (indicated by white arrows in (a)) on the shape-index surface and flat region (indicated by red dashed area in (b)) on the curvedness surface, indicating the characteristic mapping pattern for $^{i}\pi$–$^{i}\pi$ stacking of **2pd**$^{-}$ and the core part of TATA^{+}.[S11] Atom color code: gray, white, and blue refer to carbon, hydrogen, and nitrogen, respectively.

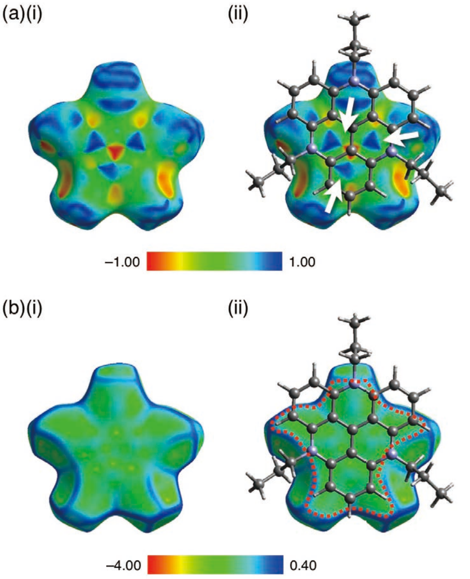

Fig. S40 Hirshfeld surface[S10] of PCCp^{-} in the crystal structure of TATA^{+}-PCCp^{-}[S12] mapped over (a) shape-index property and (b) curvedness property: (i) only surface and (ii) surface with a ball-and-stick model of the neighboring TATA^{+}. Shape index is a qualitative measure of shape and is sensitive to subtle changes in surface shape, particularly in a flat region by differing by sign represent complementary bumps (blue) and hollows (red), whereas curvedness is a function of the root-mean-square curvature of the surface, and maps of curvedness typically show large regions of green (relatively flat) separated by dark blue edges (large positive curvature). The surfaces of PCCp^{-} showed the red and blue triangles arranged in bow-tie shapes (indicated by white arrows in (a)) on the shape-index surface and flat region (indicated by red dashed area in (b)) on the curvedness surface, indicating the characteristic mapping pattern for $^{i}\pi$–$^{i}\pi$ stacking of PCCp^{-} and the core part of TATA^{+}.[S11] Atom color code: gray, white, and blue refer to carbon, hydrogen, and nitrogen, respectively.

4. Theoretical studies

DFT calculations. DFT calculations for the geometrical optimizations were carried out by using the *Gaussian 09* program.[S13]

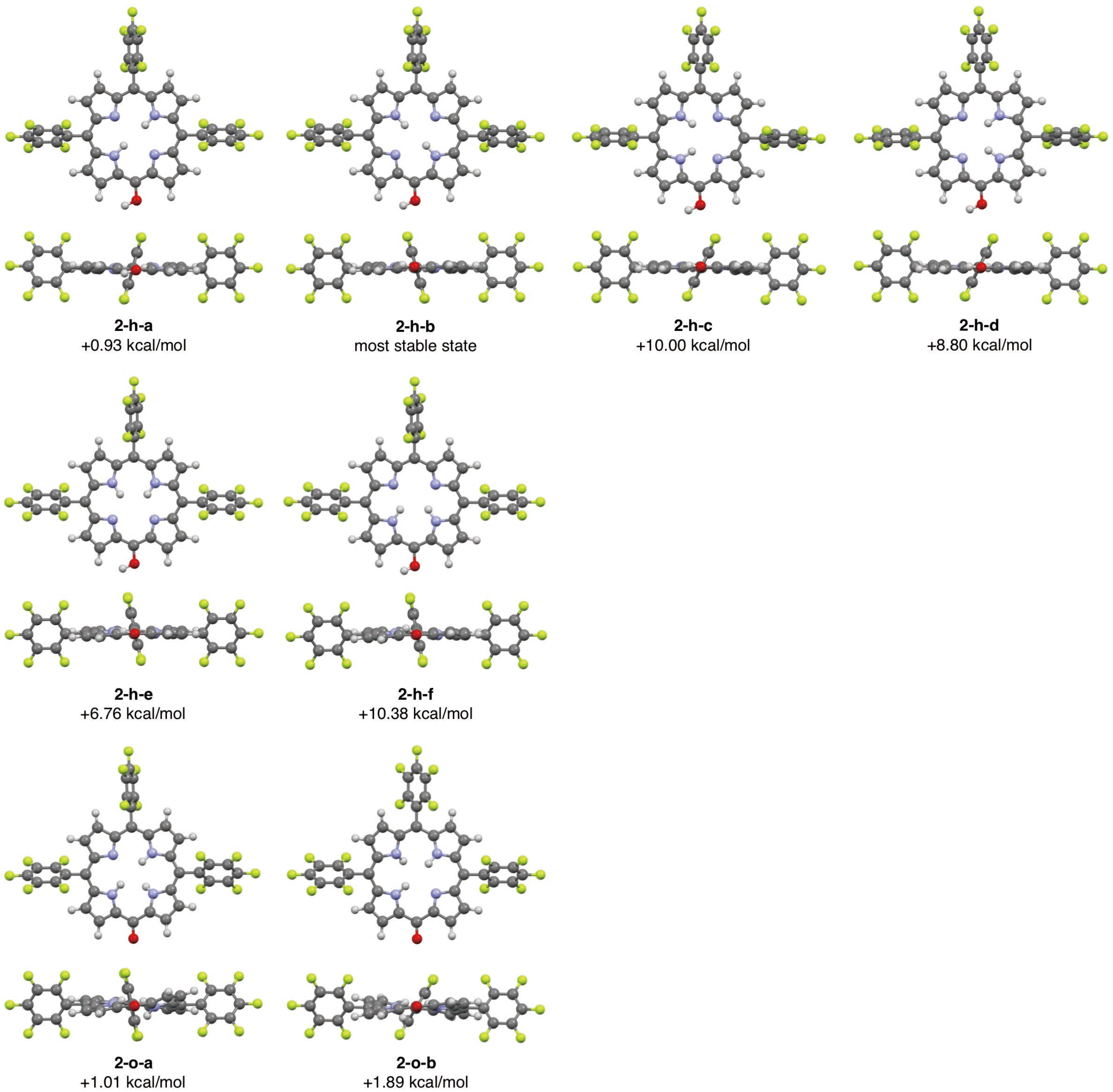

Fig. S41 Optimized structures of **2** as hydroxyporphyrin-type **2-h**, oxophlorin-type **2-o**, and isooxophlorin-type **2-i** at B3LYP/6-31G(d,p) level. **2-h**, **2-o**, and **2-i** have OH in an inner-2H form, C=O in an inner-3H form, and C=O in a *meso*-CH form, respectively. Hydroxyporphyrin-type tautomer **2-h-b** was the most stable among all the tautomers.

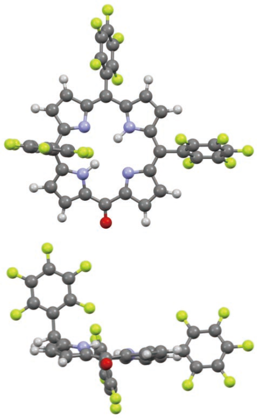

2-i-a
+10.33 kcal/mol

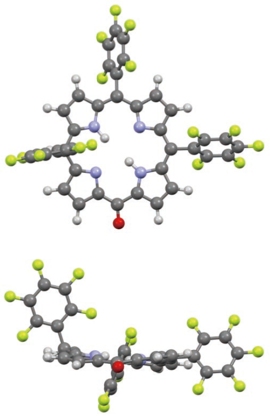

2-i-b
+26.47 kcal/mol

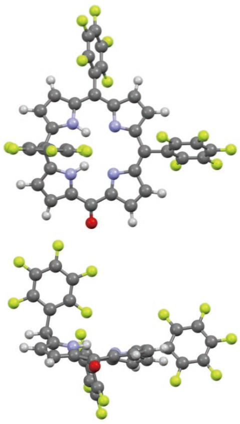

2-i-c
+17.89 kcal/mol

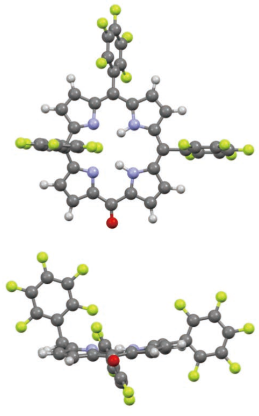

2-i-d
+32.80 kcal/mol

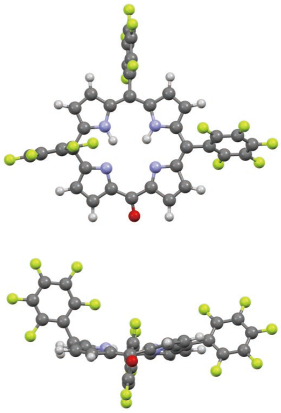

2-i-e
+35.29 kcal/mol

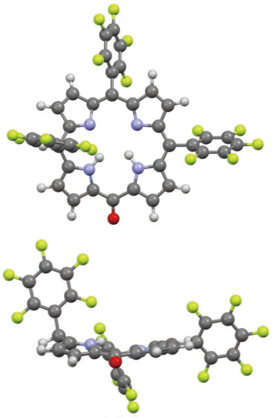

2-i-f
+18.51 kcal/mol

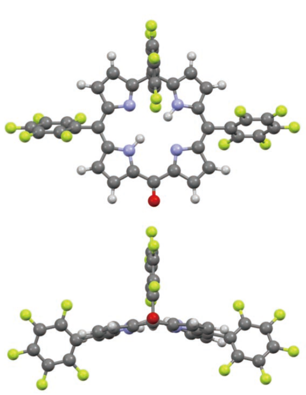

2-i-g
+8.35 kcal/mol

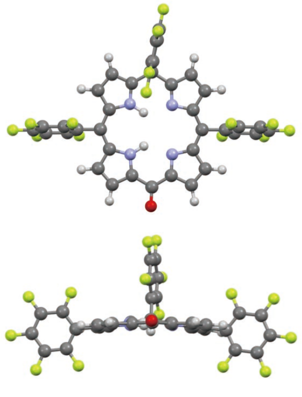

2-i-h
+24.91 kcal/mol

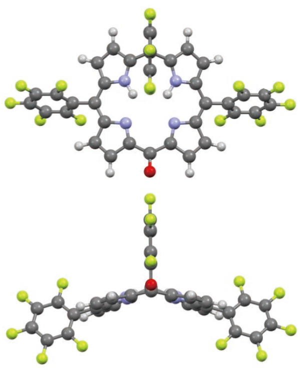

2-i-i
+14.41 kcal/mol

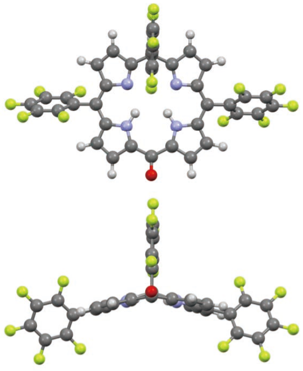

2-i-j
+13.69 kcal/mol

Fig. S41 (Continued)

Table S3 Summarized energy differences (kcal/mol) of hydroxyporphyrin-type and oxophlorin-type tautomers (Fig. S41) of **2** optimized in vacuum at B3LYP/6-31G(d,p) level and in DMSO, CH_3CN, CH_3OH, acetone, CH_2Cl_2, toluene, and *n*-heptane at PCM-B3LYP/6-31G(d,p), showing small solvent effects. The energy differences among **2-h-a**,**b** and **2-o-a** (differences between hydroxyporphyrin-type and oxophlorin-type tautomers) were smaller in nonpolar solvents and those were larger in polar solvents. On the other hand, the energy differences between **2-o-a**,**b** (difference between two oxophlorin-type tautomers) were smaller in polar solvents and those were larger in nonpolar solvents.

	vacuum	DMSO	CH_3CN	CH_3OH	acetone	CH_2Cl_2	toluene	*n*-heptane
**2-h-a**	0.93	1.41	1.40	1.39	1.37	1.26	0.68	0.77
**2-h-b**	0	1.09	1.07	1.06	1.02	0.84	0.04	0
**2-h-c**	10.00	9.30	9.31	9.31	9.34	9.40	9.36	9.49
**2-h-d**	8.80	8.85	8.85	8.85	8.85	8.82	8.47	8.52
**2-h-e**	6.76	7.28	7.27	7.27	7.25	7.16	6.61	6.62
**2-h-f**	10.38	10.26	10.26	10.26	10.26	10.24	9.94	10.02
**2-o-a**	1.01	0	0	0	0	0	0	0.19
**2-o-b**	1.89	0.17	0.19	0.19	0.23	0.36	0.69	0.94

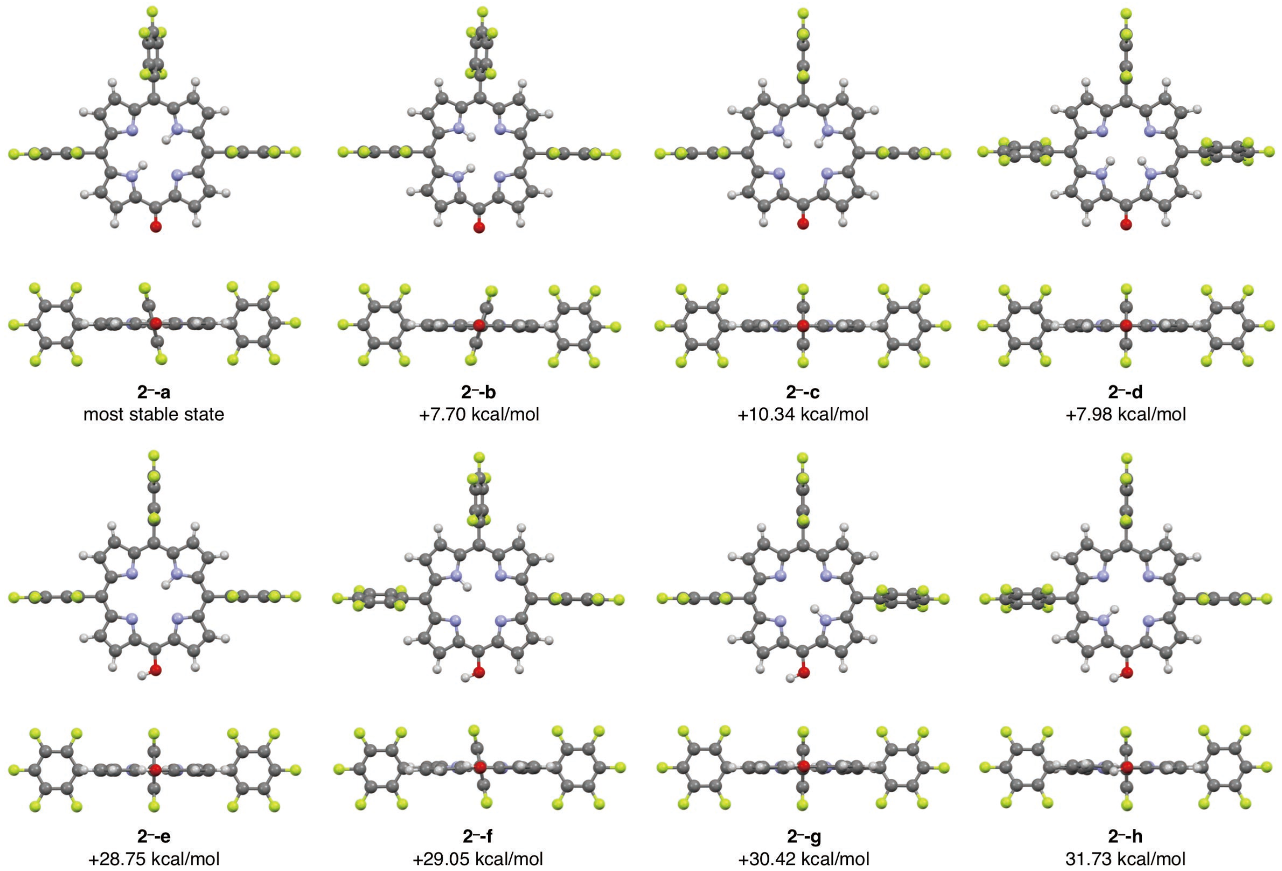

Fig. S42 Optimized structures (eight representative tautomers) of **2**$^-$ at B3LYP/6-31+G(d,p) level.

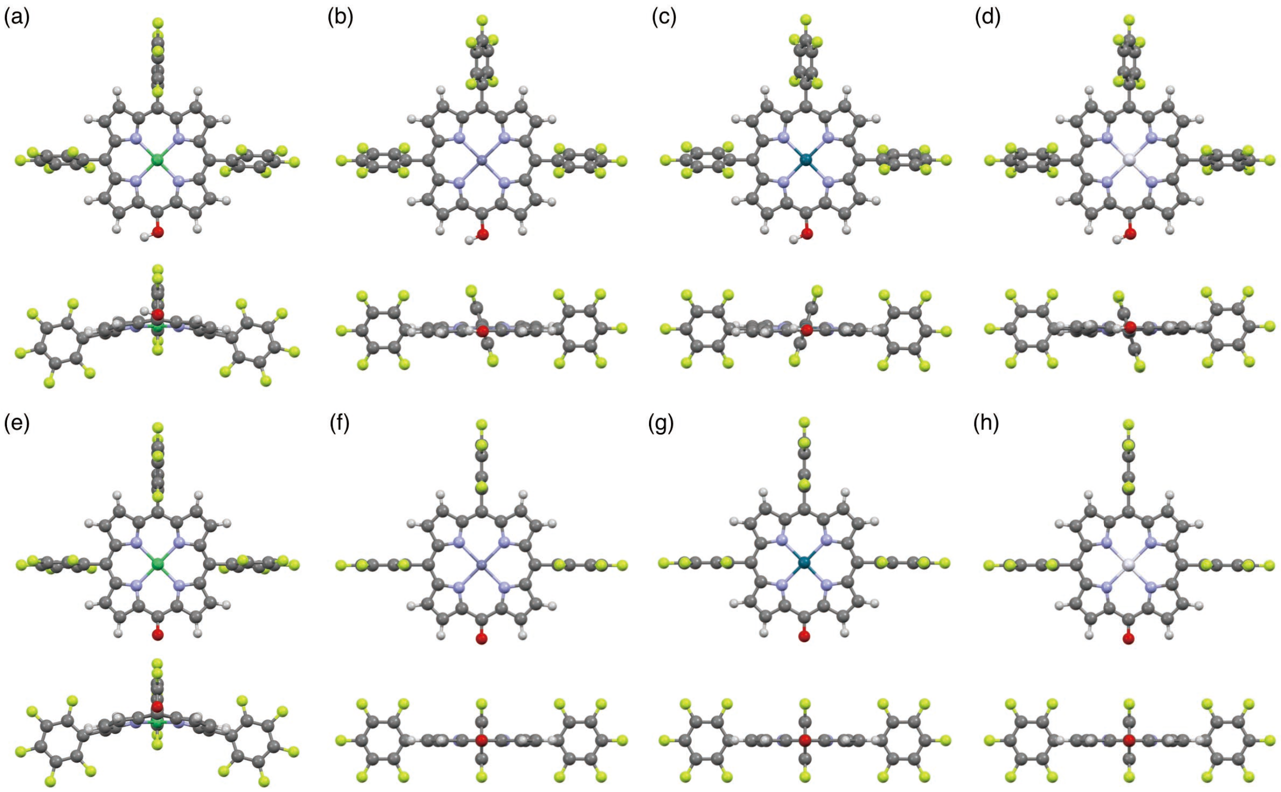

Fig. S43 Optimized structures of (a) **2ni**, (b) **2zn**, (c) **2pd**, and (d) **2pt** at B3LYP/6-31G(d,p) for C, H, N, O, and F and B3LYP/LanL2DZ for Ni, Zn, Pd, and Pt and (e) **2ni**$^{-}$, (f) **2zn**$^{-}$, (g) **2pd**$^{-}$, and (h) **2pt**$^{-}$ at B3LYP/6-31+G(d,p) for C, H, N, O, and F and B3LYP/LanL2DZ for Ni, Zn, Pd, and Pt.

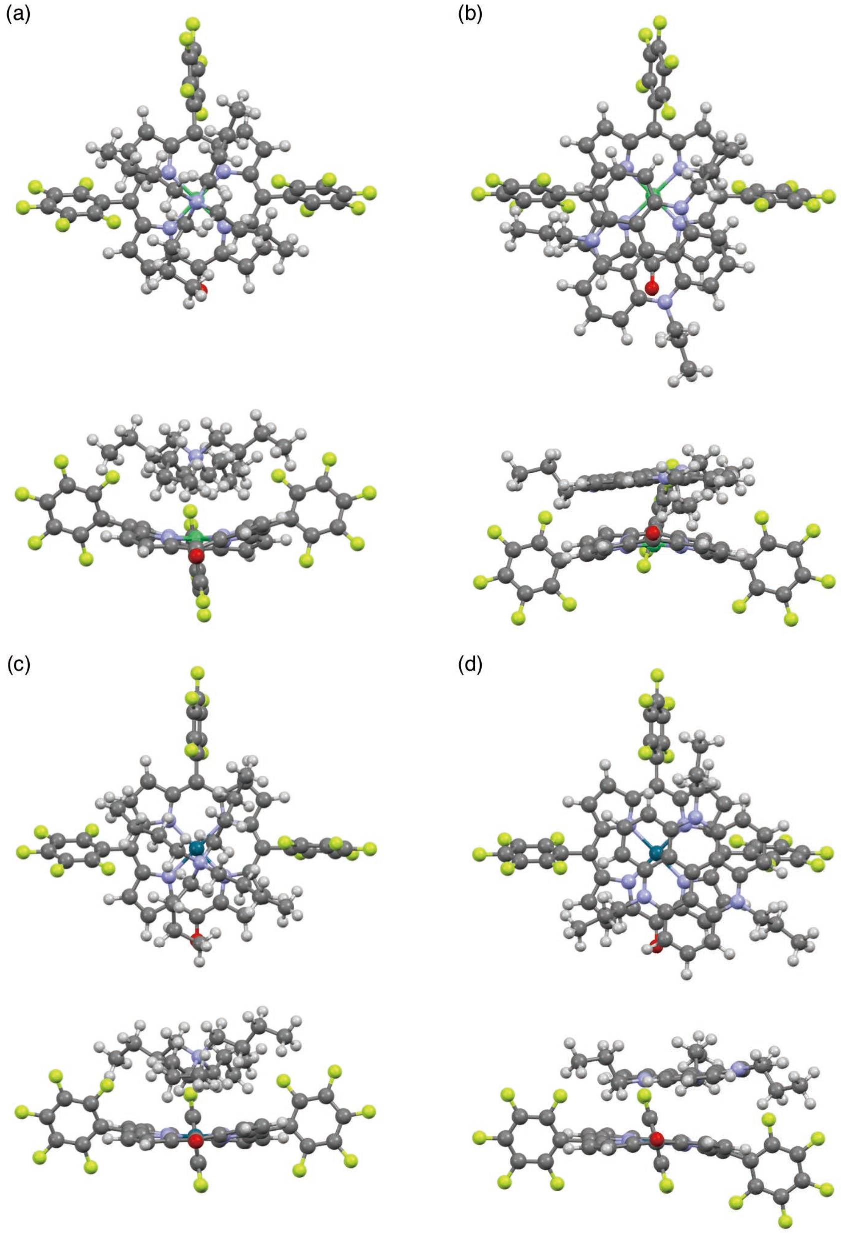

Fig. S44 Optimized structures of (a) **2ni**⁻-TBA⁺, (b) **2ni**⁻-TATA⁺, (c) **2pd**⁻-TBA⁺, and (d) **2pd**⁻-TATA⁺, based on the crystal structures, at B3LYP-GD3BJ/6-31+G(d,p) for C, H, N, O, and F and B3LYP-GD3BJ/LanL2DZ for Ni and Pd.

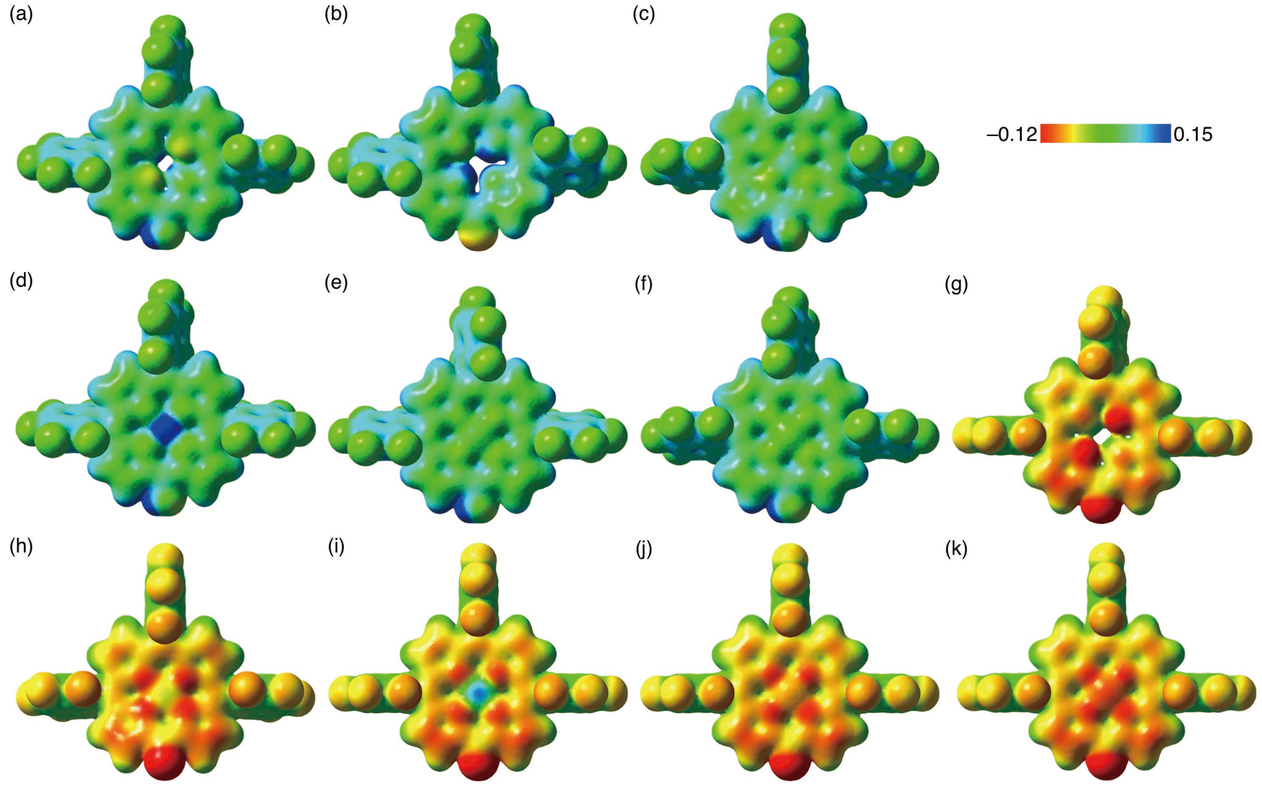

Fig. S45 Electrostatic potential (ESP) mapping (δ = 0.01) of (a) **2-h-b**, (b) **2-o-a**, (c) **2ni**, (d) **2zn**, (e) **2pd**, (f) **2pt**, (g) **2⁻-a** (h) **2ni⁻**, (i) **2zn⁻**, (j) **2pd⁻**, and (k) **2pt⁻** at B3LYP/6-31+G(d,p) for C, H, N, O, and F and B3LYP/LanL2DZ for Ni, Zn, Pd, and Pt. The ESP mappings of the π-electronic anions indicated the delocalization of negative charge of the anionic oxygen into porphyrin frameworks. Furthermore, even with the introduction of three C_6F_5 groups as electron-withdrawing substituents, the porphyrin cores of the π-electronic anions have the negative charge comparable to the core of the tetraphenylporphyrin derivative (free base) bearing sulfonate units at the two *meso*-phenyl 4-positions.

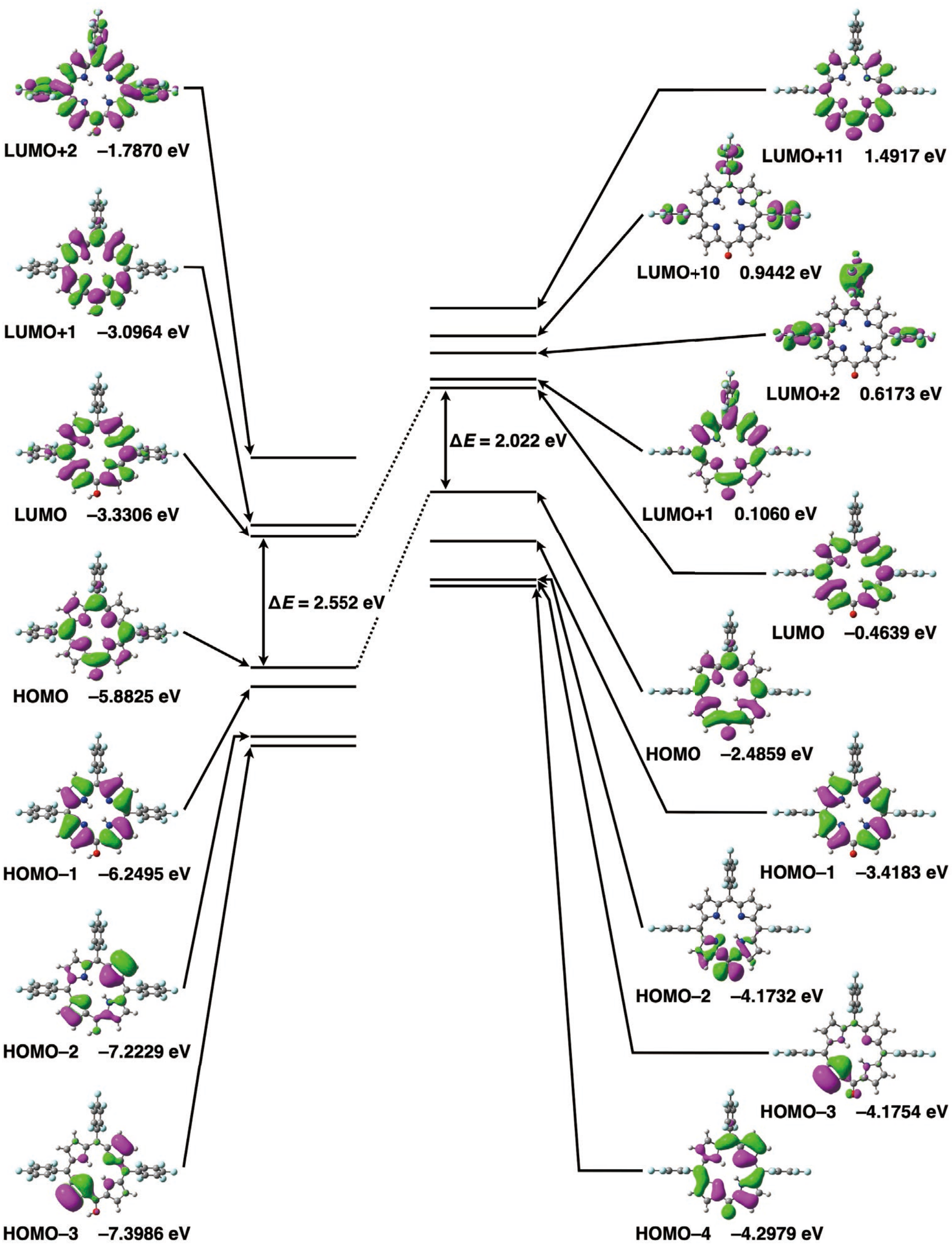

Fig. S46 Molecular orbitals (HOMO/LUMO) of **2-h-b** (left) and **2⁻-a** (right) estimated at B3LYP/6-31+G(d,p).

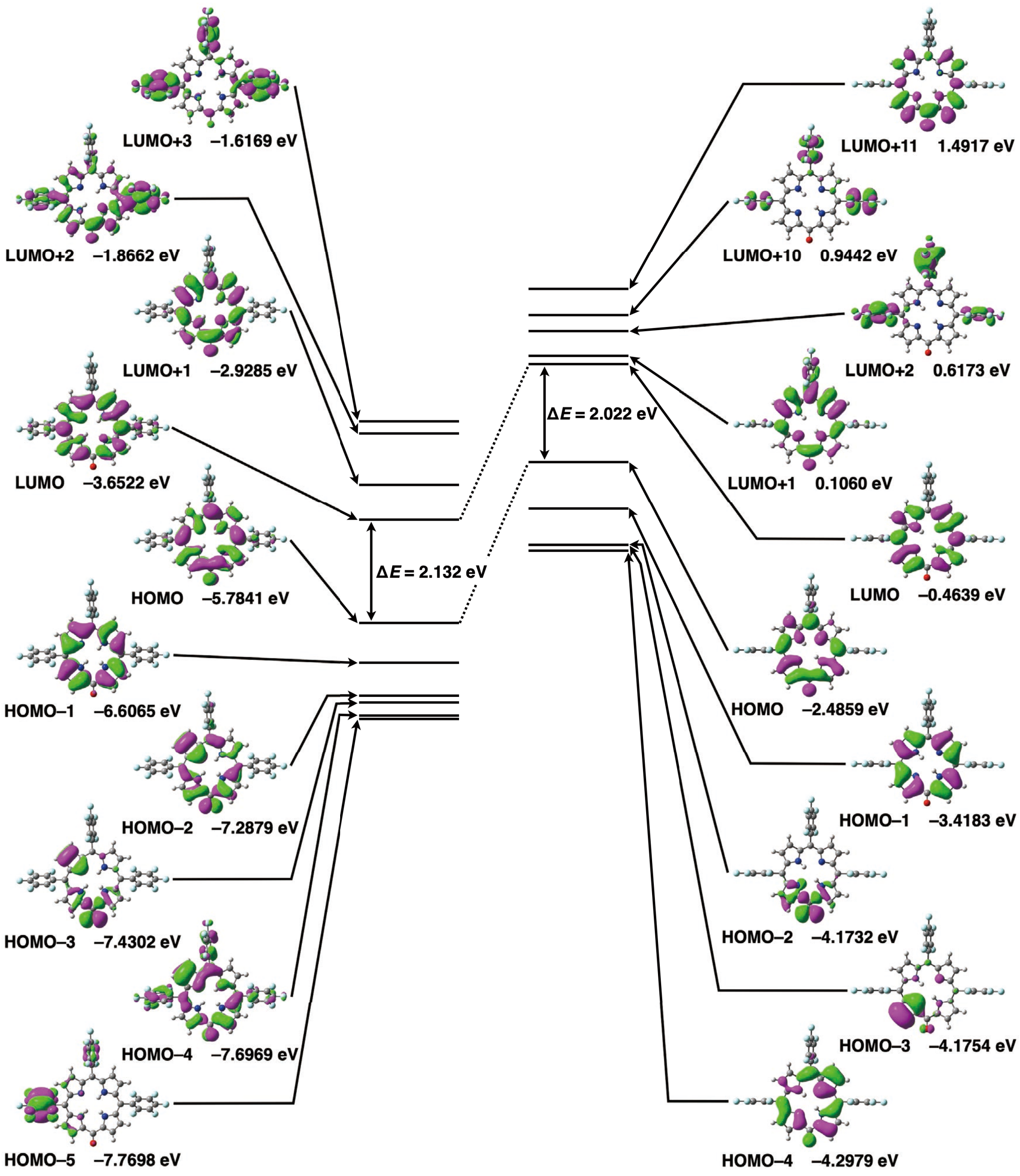

Fig. S47 Molecular orbitals (HOMO/LUMO) of **2-o-a** (left) and **2⁻-a** (right) (Fig. S46 right) estimated at B3LYP/6-31+G(d,p).

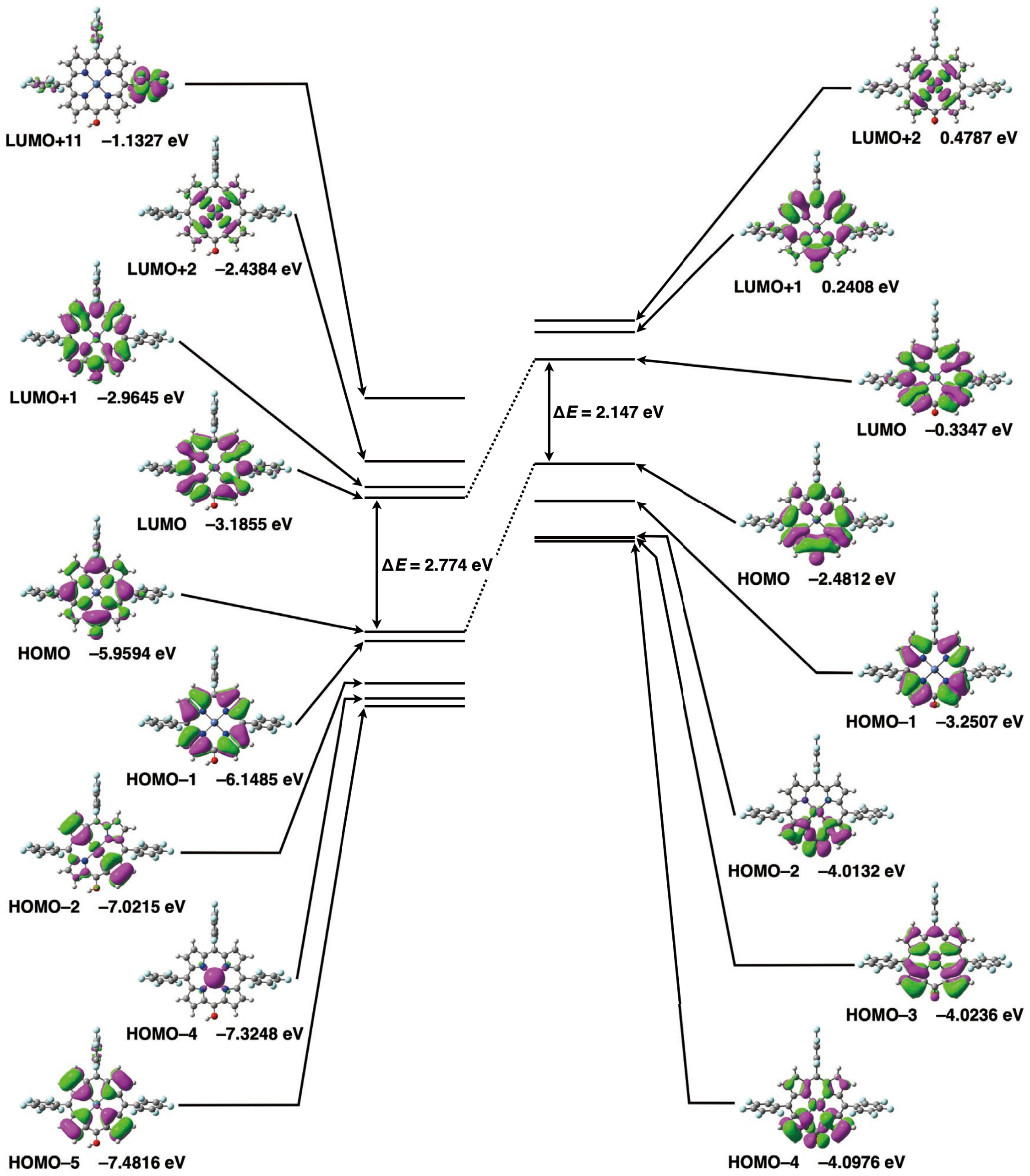

Fig. S48 Molecular orbitals (HOMO/LUMO) of **2ni** (left) and **2ni**$^{-}$ (right) estimated at B3LYP/6-31+G(d,p) for C, H, N, O, and F and B3LYP/LanL2DZ for Ni.

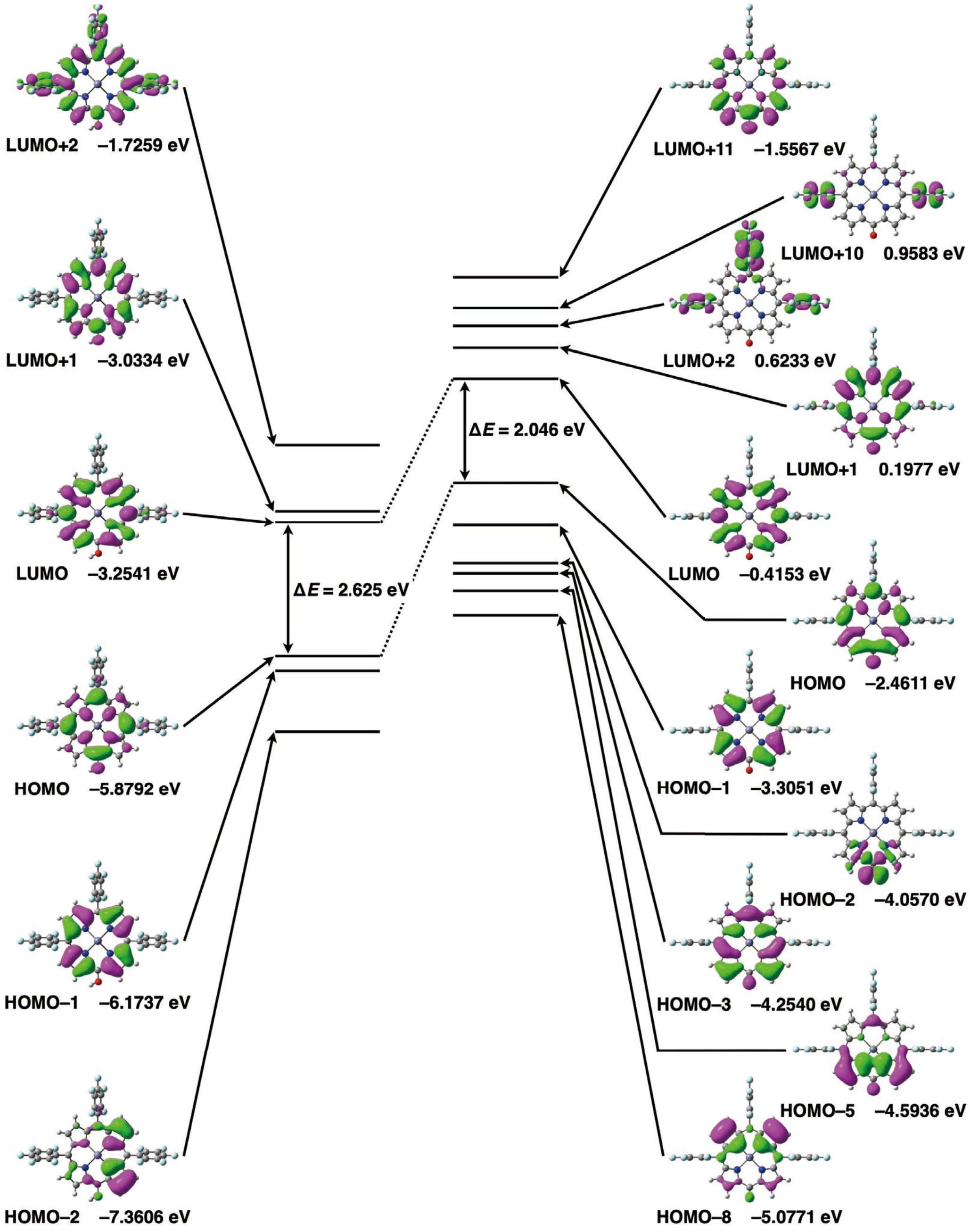

Fig. S49 Molecular orbitals (HOMO/LUMO) of **2zn** (left) and **2zn**$^{-}$ (right) estimated at B3LYP/6-31+G(d,p) for C, H, N, O, and F and B3LYP/LanL2DZ for Zn.

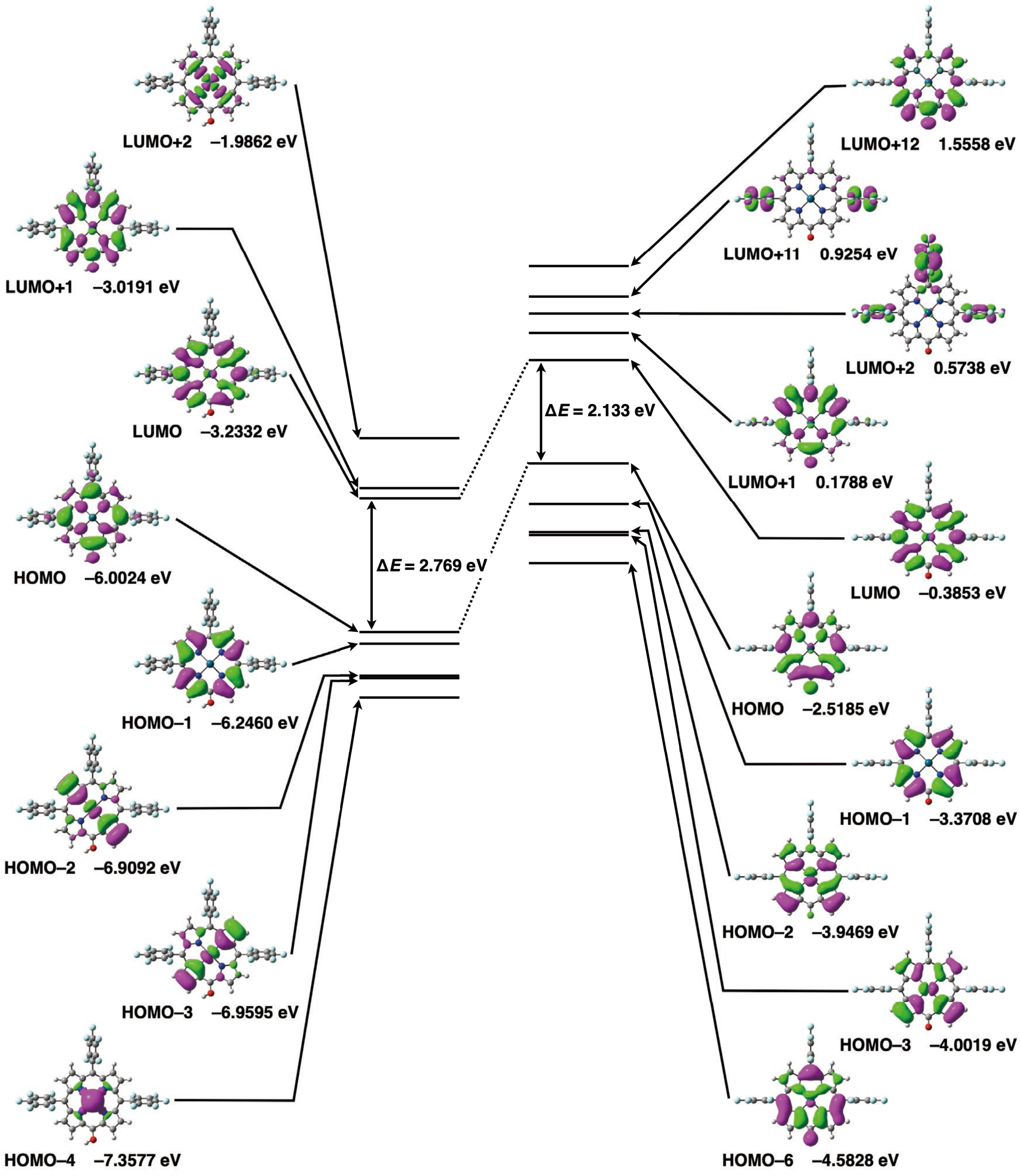

Fig. S50 Molecular orbitals (HOMO/LUMO) of **2pd** (left) and **2pd**$^{-}$ (right) estimated at B3LYP/6-31+G(d,p) for C, H, N, O, and F and B3LYP/LanL2DZ for Pd.

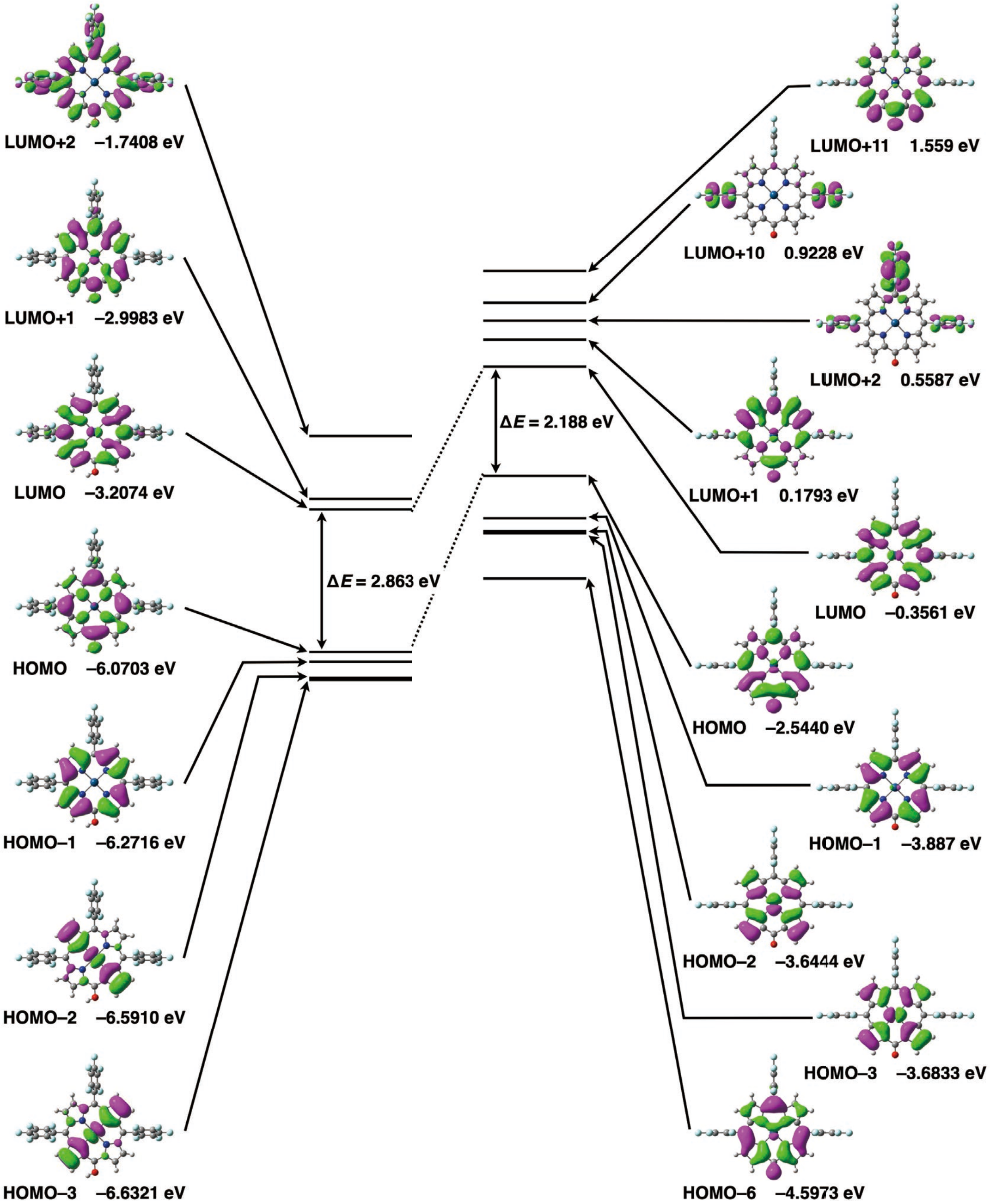

Fig. S51 Molecular orbitals (HOMO/LUMO) of **2pt** (left) and **2pt**$^{-}$ (right) estimated at B3LYP/6-31+G(d,p) for C, H, N, O, and F and B3LYP/LanL2DZ for Pt.

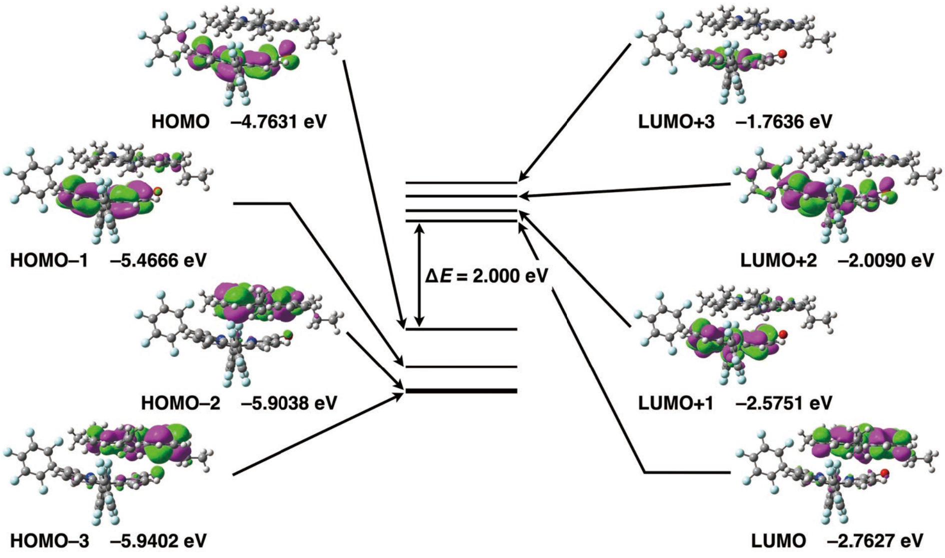

Fig. S52 Molecular orbital (HOMO/LUMO) of **2ni**$^{-}$-TATA^{+} estimated at B3LYP/6-31+G(d,p) for C, H, N, O, and F and B3LYP/LanL2DZ for Ni.

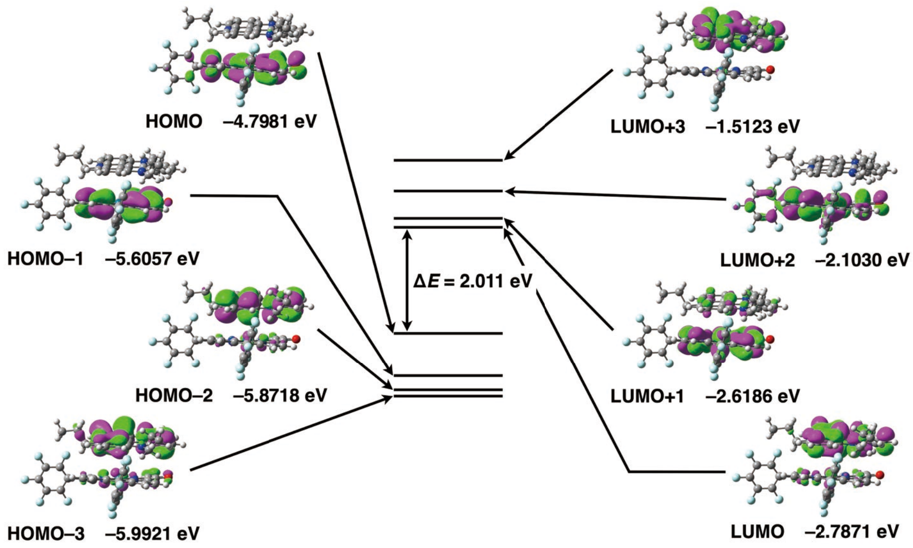

Fig. S53 Molecular orbital (HOMO/LUMO) of **2pd**$^{-}$-TATA^{+} estimated at B3LYP/6-31+G(d,p) for C, H, N, O, and F and B3LYP/LanL2DZ for Pd.

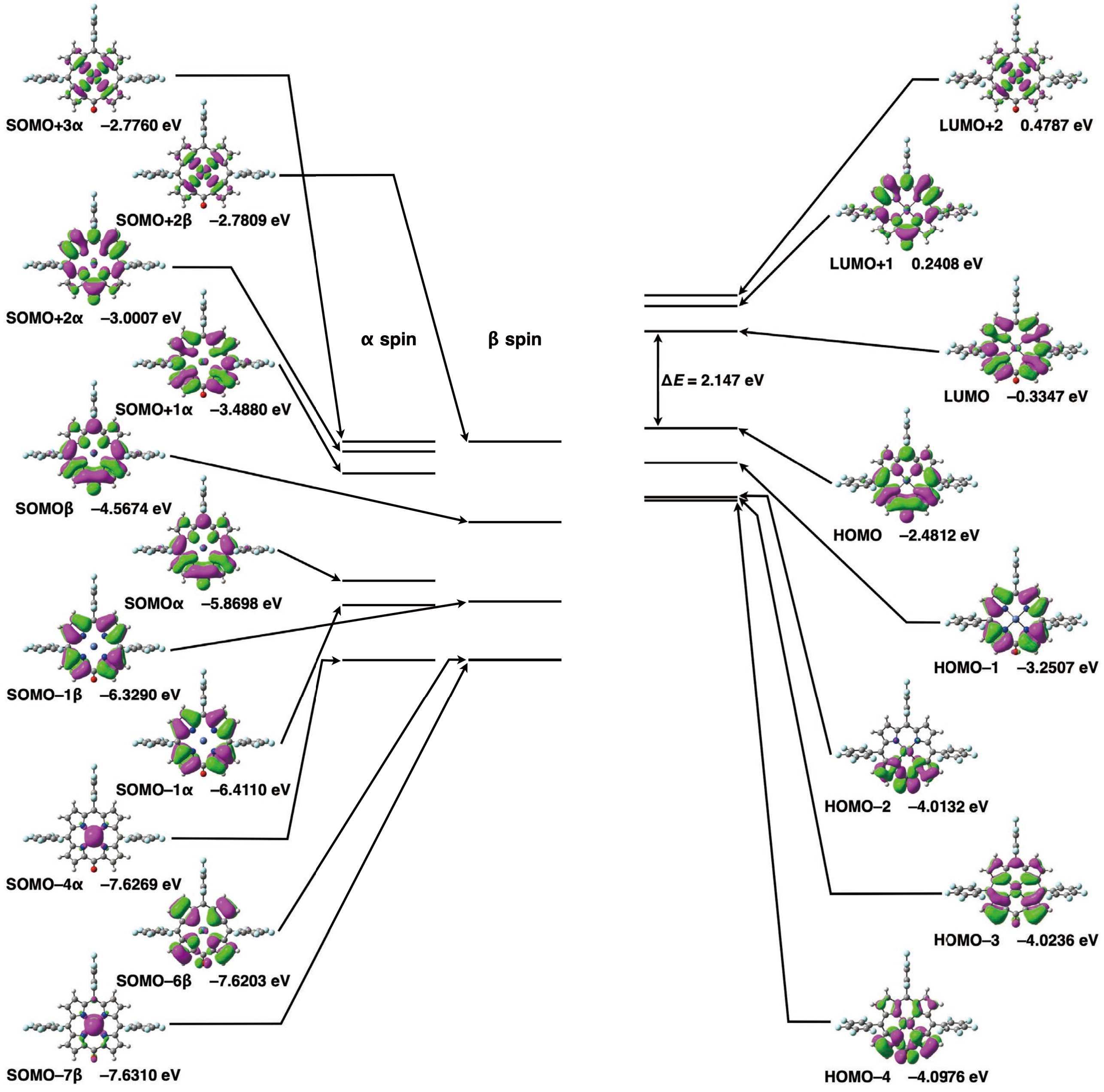

Fig. S54 Molecular orbitals (HOMO/LUMO) of **2ni** radical as the one-electron-oxidized state of **2ni**⁻ (left) at estimated at UB3LYP/6-31+G(d,p) for C, H, N, O, and F and UB3LYP/LanL2DZ for Ni and **2ni**⁻ (right) (Fig. S48 right) estimated at B3LYP/6-31+G(d,p) for C, H, N, O, and F and B3LYP/LanL2DZ for Ni.

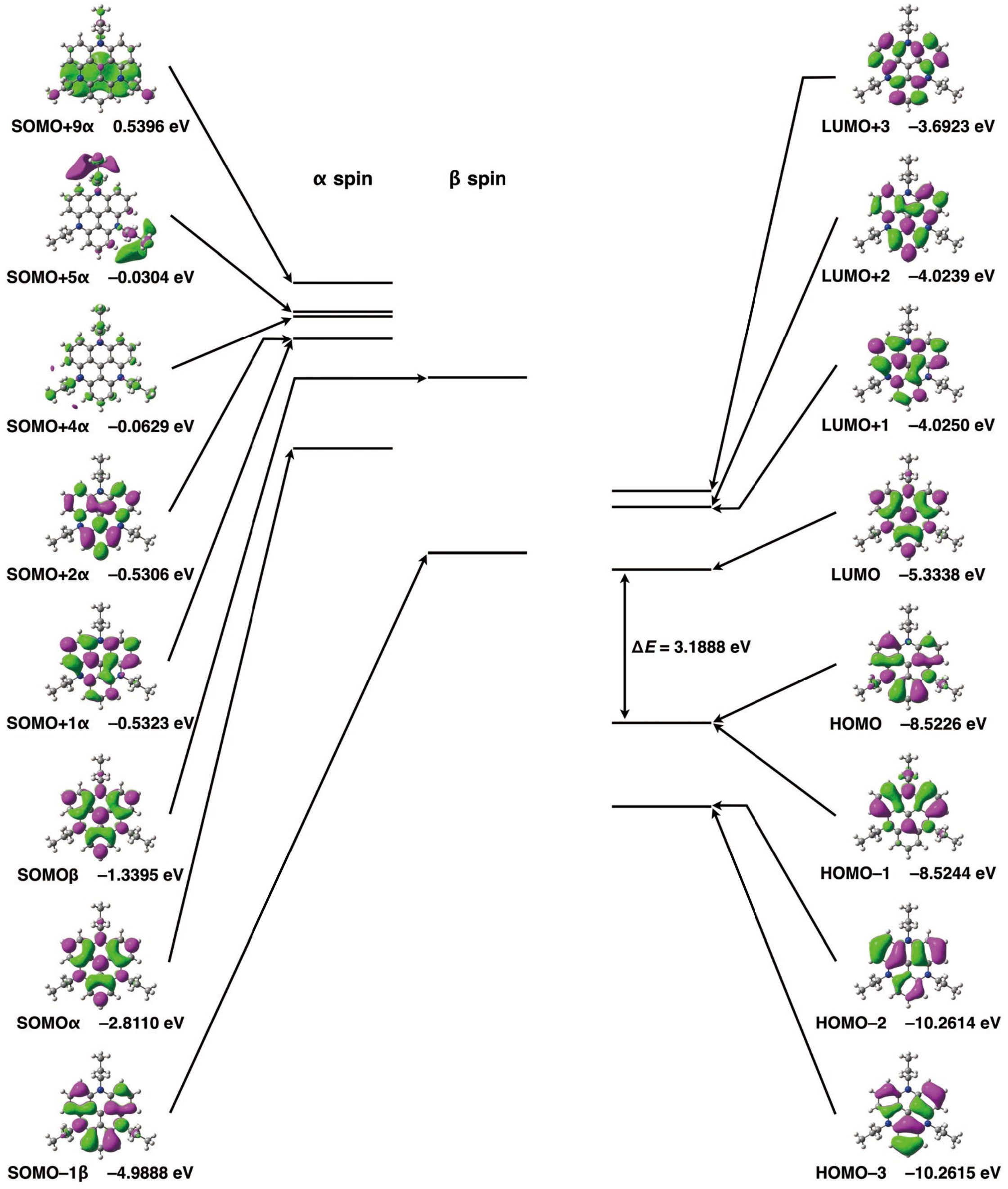

Fig. S55 Molecular orbitals (HOMO/LUMO) of TATA radical (left) estimated at UB3LYP/6-31+G(d,p) and $TATA^+$ (right) estimated at B3LYP/6-31+G(d,p).

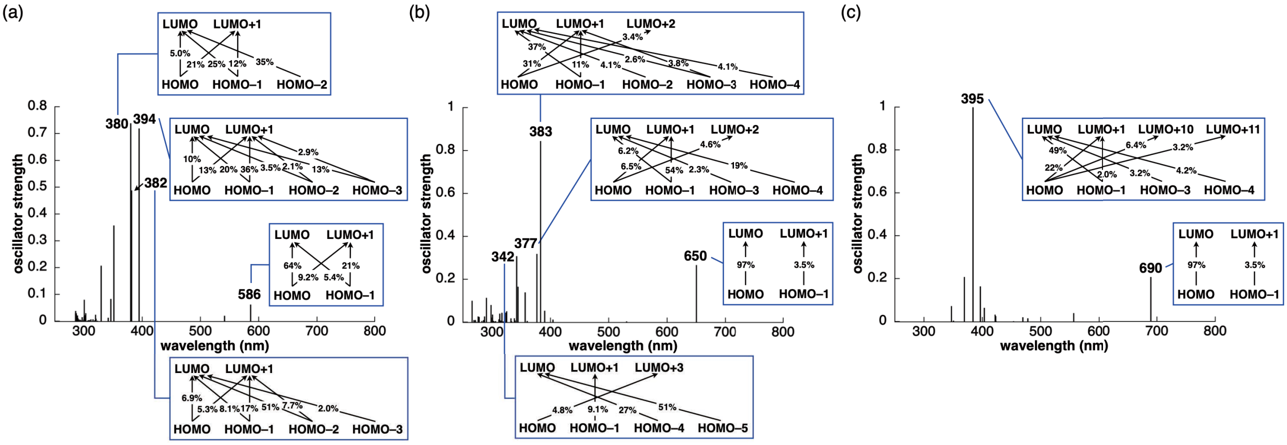

Fig. S56 TD-DFT-based UV/vis absorption stick spectra of (a) **2-h-b**, (b) **2-o-a**, and (c) **2⁻-a** with the transitions correlated with molecular orbitals estimated at B3LYP/6-31+G(d,p). These theoretical results were consistent with the observed spectra (Fig. S8).

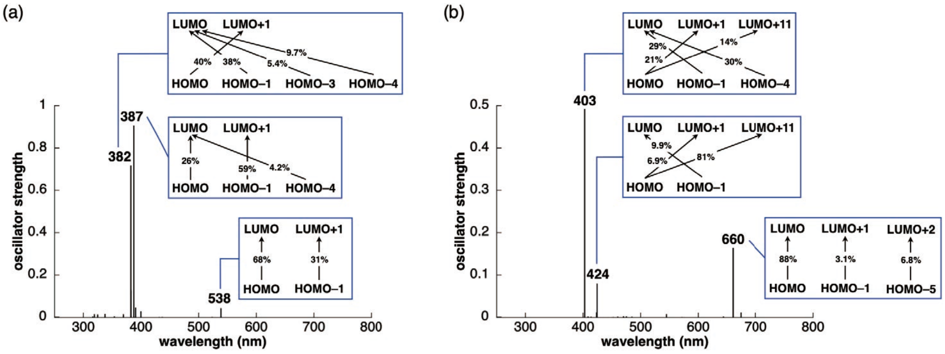

Fig. S57 TD-DFT-based UV/vis absorption stick spectra of (a) **2ni** and (b) **2ni⁻** with the transitions correlated with molecular orbitals estimated at B3LYP/6-31+G(d,p) for C, H, N, O, and F and B3LYP/LanL2DZ for Ni. These theoretical results were consistent with the observed spectra (Fig. S8).

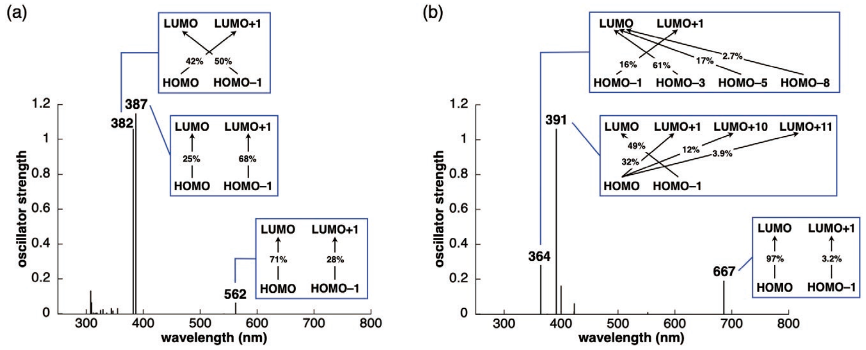

Fig. S58 TD-DFT-based UV/vis absorption stick spectra of (a) **2zn** and (b) **2zn⁻** with the transitions correlated with molecular orbitals estimated at B3LYP/6-31+G(d,p) for C, H, N, O, and F and B3LYP/LanL2DZ for Zn. These theoretical results were consistent with the observed spectra (Fig. S8).

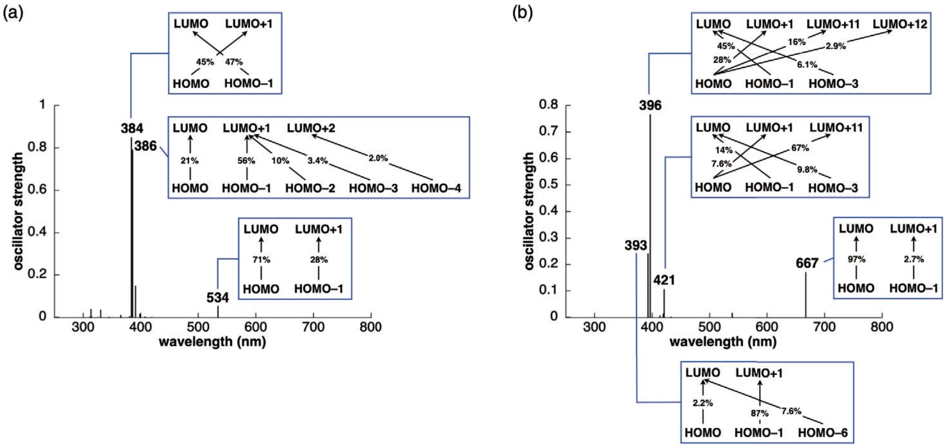

Fig. S59 TD-DFT-based UV/vis absorption stick spectra of (a) **2pd** and (b) **2pd**⁻ with the transitions correlated with molecular orbitals estimated at B3LYP/6-31+G(d,p) for C, H, N, O, and F and B3LYP/LanL2DZ for Pd. These theoretical results were consistent with the observed spectra (Fig. S8).

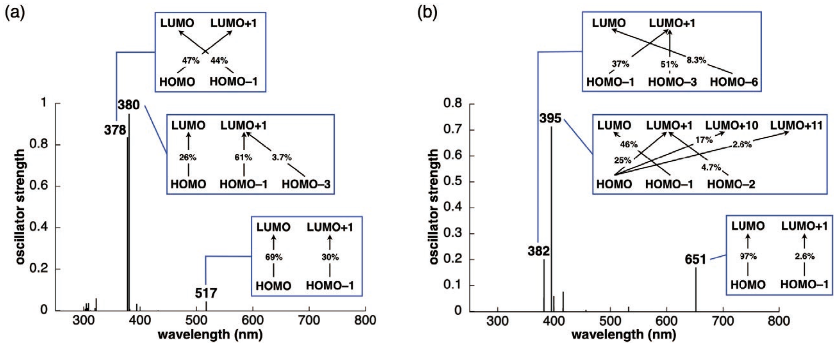

Fig. S60 TD-DFT-based UV/vis absorption stick spectra of (a) **2pt** and (b) **2pt**⁻ with the transitions correlated with molecular orbitals estimated at B3LYP/6-31+G(d,p) for C, H, N, O, and F and B3LYP/LanL2DZ for Pt. These theoretical results were consistent with the observed spectra (Fig. S8).

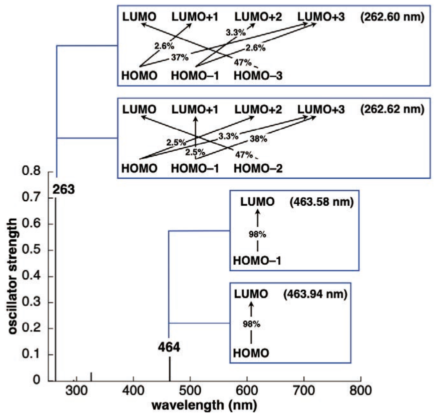

Fig. S61 TD-DFT-based UV/vis absorption stick spectra of TATA^{+} with the transitions correlated with molecular orbitals estimated at B3LYP/6-31+G(d,p).

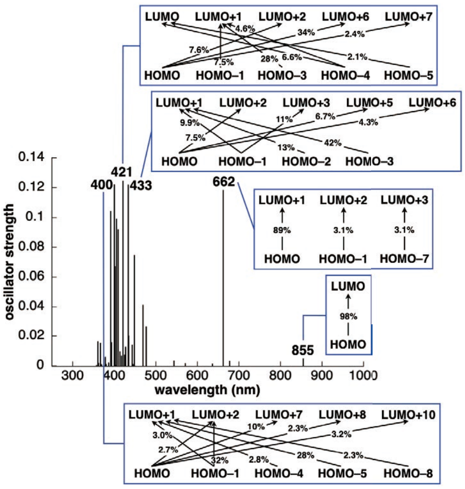

Fig. S62 TD-DFT-based UV/vis absorption stick spectra of **2ni**⁻-TATA⁺ with the transitions correlated with molecular orbitals estimated at B3LYP-GD3BJ/6-31+G(d,p) for C, H, N, O, and F and B3LYP-GD3BJ/LanL2DZ for Ni. The estimated excitation at 855 nm includes the transition from HOMO, localized at the negative charged core of **2ni**⁻, to LUMO, localized at the positive charged core of TATA⁺ (Fig. S52). This result suggests the charge transfer from **2ni**⁻ to TATA⁺, which was not observed by UV/vis absorption spectrum (Fig. S93).

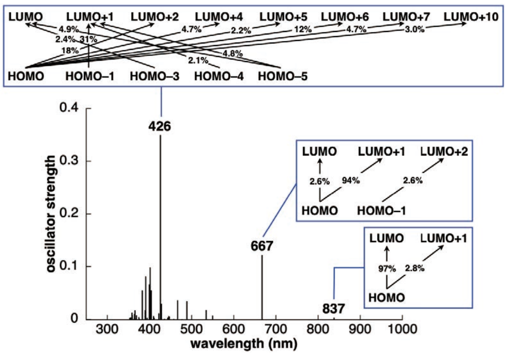

Fig. S63 TD-DFT-based UV/vis absorption stick spectra of **2pd**⁻-TATA⁺ with the transitions correlated with molecular orbitals estimated at B3LYP-GD3BJ/6-31+G(d,p) for C, H, N, O, and F and B3LYP-GD3BJ/LanL2DZ for Pd. The estimated excitation at 837 nm includes the major transition from HOMO, localized at the negative charged core of **2pd**⁻, to LUMO, localized at the positive charged core of TATA⁺ (Fig. S53). This result suggests the charge transfer from **2pd**⁻ to TATA⁺, which was not observed by UV/vis absorption spectrum (Fig. S94).

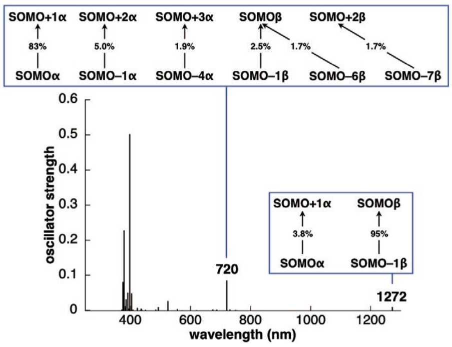

Fig. S64 TD-DFT-based UV/vis absorption stick spectra of **2ni** radical with the transitions correlated with molecular orbitals estimated at UB3LYP/6-31+G(d,p) for C, H, N, O, and F and UB3LYP/LanL2DZ for Ni. In spectroelectrochemical analysis of **2ni**⁻-TBA⁺, the broad peak at 795 nm in the oxidation states can be assigned as a component of **2ni** radical (Fig. S105a): the theoretically estimated excitation at 720 nm includes the major transition from SOMOα to SOMO+1α, both of which are localized at the core π-system (Fig. S54).

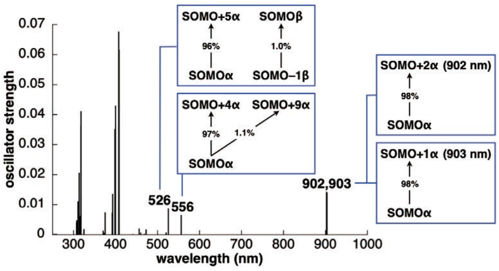

Fig. S65 TD-DFT-based UV/vis absorption stick spectra of TATA radical with the transitions correlated with molecular orbitals estimated at UB3LYP/6-31+G(d,p). In spectroelectrochemical analysis of $TATA^+$-Cl^- (Fig. S106), the broad peak at 960 nm in the reduction states can be assigned as a component of TATA radical: the theoretically estimated excitations at 902 and 903 nm include the respective major transitions from SOMOα and SOMOα to SOMO+2α and SOMO+1α, respectively, all of which are localized at the core π-system (Fig. S55).

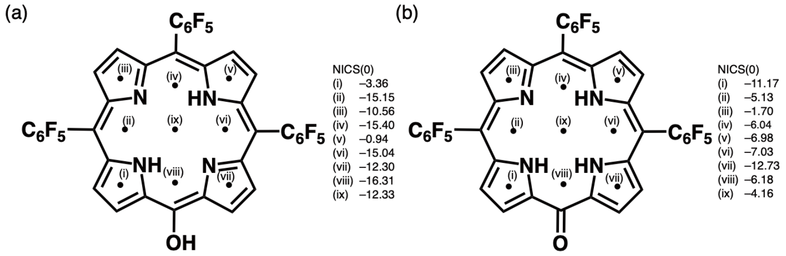

Fig. S66 NICS values (ppm) of (a) **2-h-b** and (b) **2-o-a** calculated at B3LYP/6-31+G(d,p).

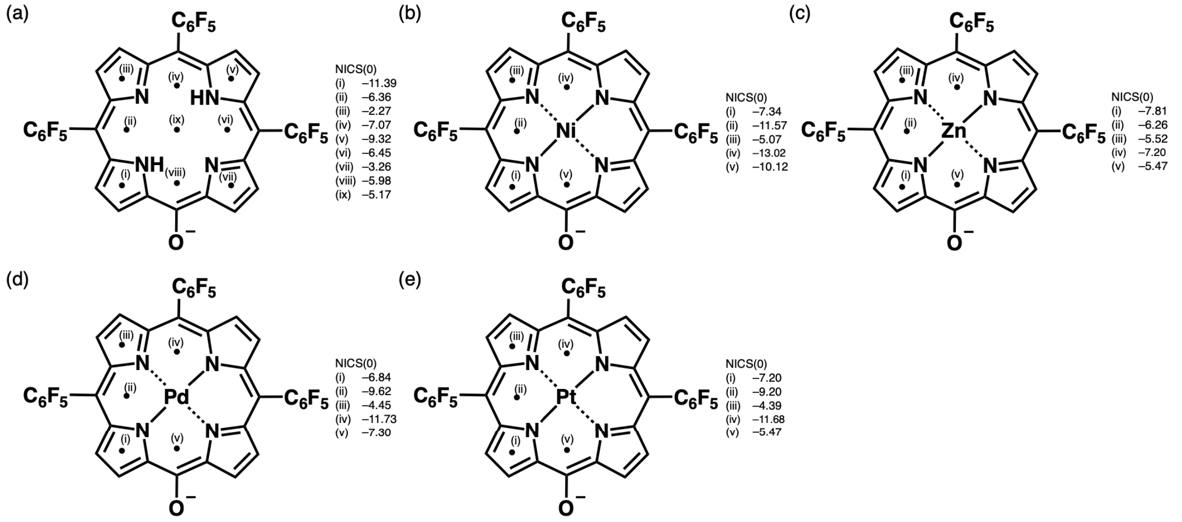

Fig. S67 NICS values (ppm) of (a) $\mathbf{2}^-$, (b) $\mathbf{2ni}^-$, (c) $\mathbf{2zn}^-$, (d) $\mathbf{2pd}^-$, and (e) $\mathbf{2pt}^-$ calculated at B3LYP/6-31+G(d,p) for C, H, N, O, and F and B3LYP/LanL2DZ for Ni, Zn, Pd, and Pt.

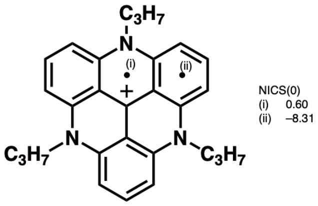

Fig. S68 NICS values (ppm) of $TATA^+$ calculated at B3LYP/6-31+G(d,p).

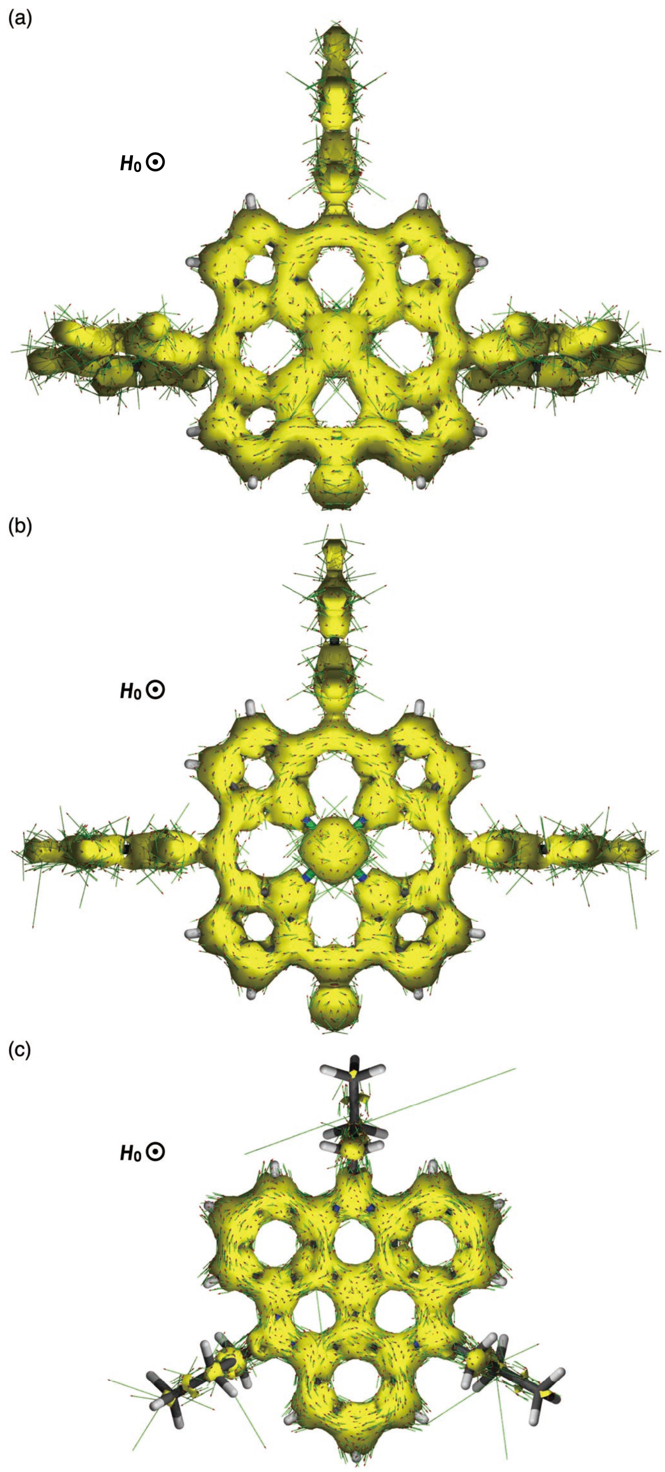

Fig. S69 Anisotropy of the current induced density (ACID)[S14] of (a) **2ni**$^{-}$, (b) **2pd**$^{-}$, and (c) TATA^{+} (top and side views) at isosurface value of δ = 0.05 based on the optimized structure at B3LYP/6-31+G(d,p) for C, H, N, O, and F and B3LYP/LanL2DZ for Ni and Pd. Current density vectors are plotted on to the ACID isosurface based on the vector of the magnetic field (H_0) which is orthogonal with respect to the molecule. The plots for **2ni**$^{-}$ and **2pd**$^{-}$ suggest that the porphyrin cores of the deprotonated anions are aromatic, and the results are in good agreement with NICS values (Fig. S67). In the plot for TATA^{+}, clockwise ring currents of the phenyl rings suggest their aromaticity, while the current of the N-containing six-membered rings show non-aromatic property, and the results are also in good agreement with NICS value (Fig. S68). These theoretical results were consistent with ^{1}H NMR spectra of **2ni**$^{-}$-TATA^{+} (Fig. S87) and **2pd**$^{-}$-TATA^{+} (Fig. S90).

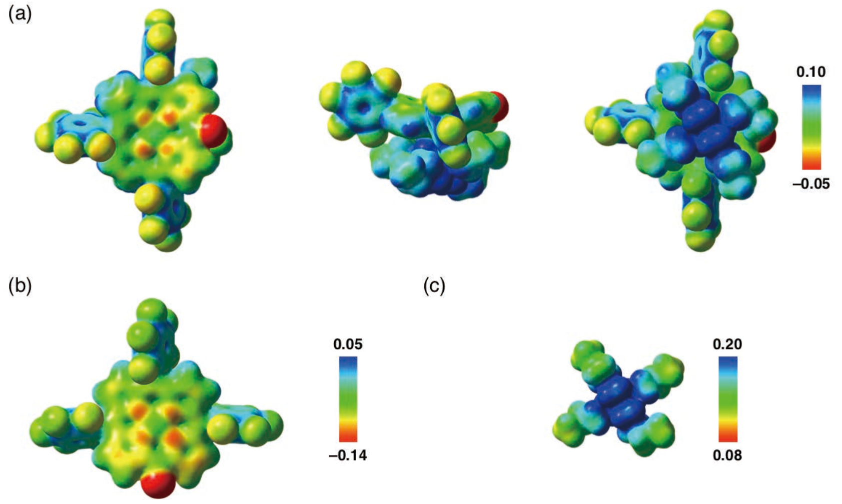

Fig. S70 Electron density diagrams (top, side, and bottom views, δ = 0.01) of (a) **2ni**$^{-}$-TBA^{+}, (b) **2ni**$^{-}$, and (c) TBA^{+} in the single-crystal X-ray structure[S5b] calculated at B3LYP/6-31+G(d,p) for C, H, N, O, and F and B3LYP/LanL2DZ for Ni.

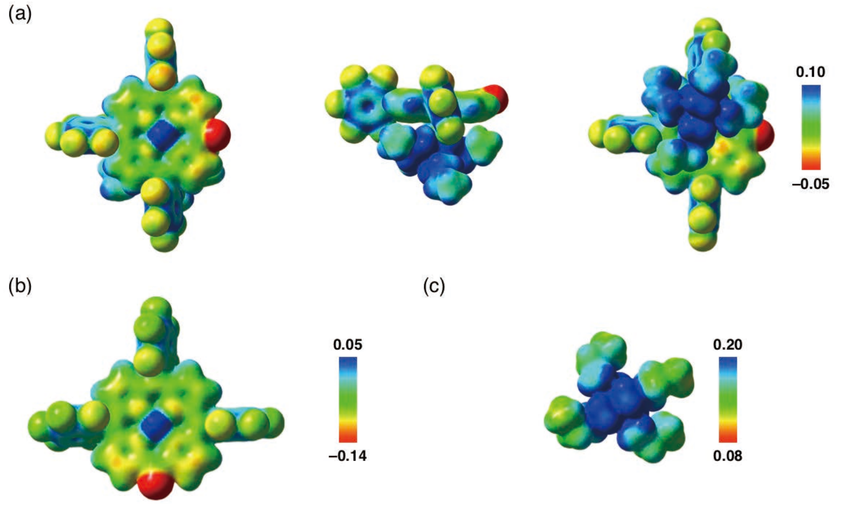

Fig. S71 Electron density diagrams (top, side, and bottom views, δ = 0.01) of (a) **2zn**$^{-}$-TBA^{+}, (b) **2zn**$^{-}$, and (c) TBA^{+} in the single-crystal X-ray structure (Fig. S20) calculated at B3LYP/6-31+G(d,p) for C, H, N, O, and F and B3LYP/LanL2DZ for Zn.

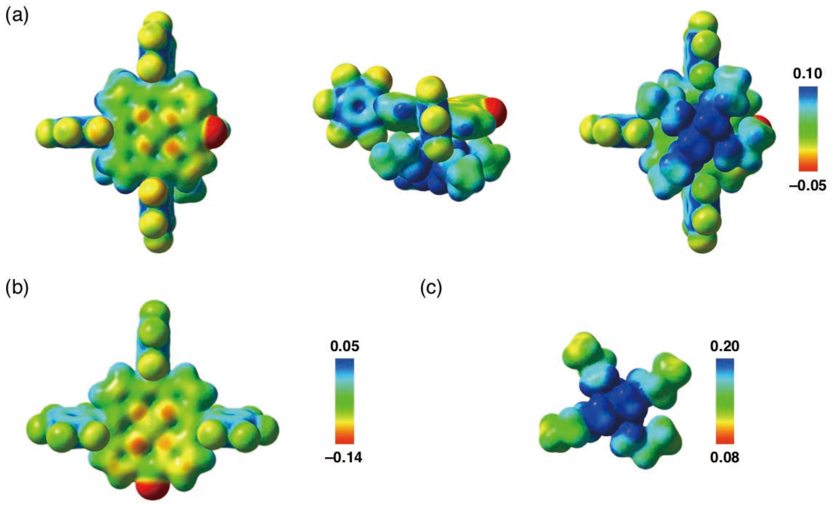

Fig. S72 Electron density diagrams (top, side, and bottom views, δ = 0.01) of (a) **2pd**$^-$-TBA$^+$, (b) **2pd**$^-$, and (c) TBA$^+$ in the single-crystal X-ray structure (Fig. S22) calculated at B3LYP/6-31+G(d,p) for C, H, N, O, and F and B3LYP/LanL2DZ for Pd.

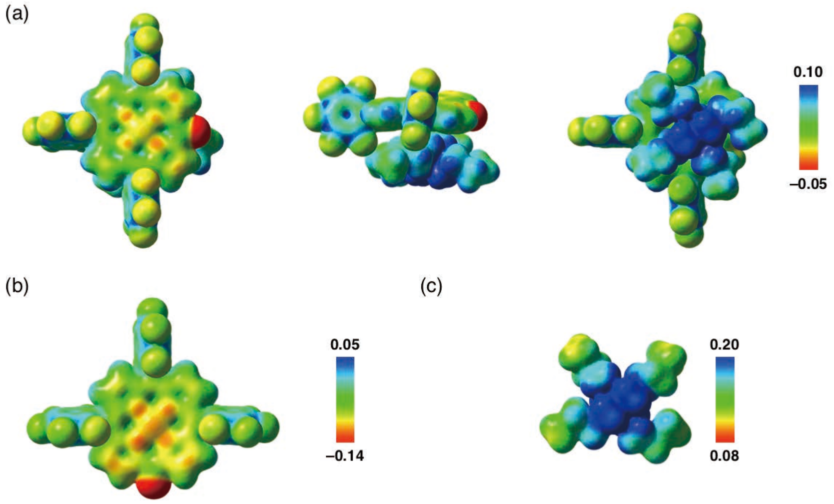

Fig. S73 Electron density diagrams (top, side, and bottom views, δ = 0.01) of (a) **2pt**$^-$-TBA$^+$, (b) **2pt**$^-$, and (c) TBA$^+$ in the single-crystal X-ray structure (Fig. S24) calculated at B3LYP/6-31+G(d,p) for C, H, N, O, and F and B3LYP/LanL2DZ for Pt.

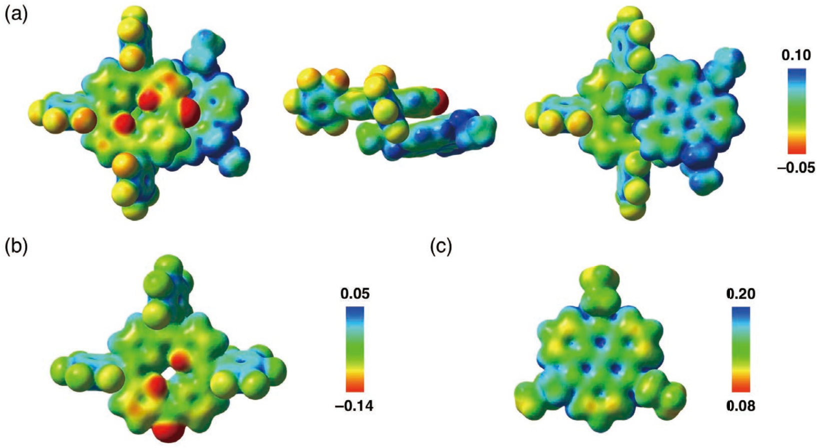

Fig. S74 Electron density diagrams (top, side, and bottom views, δ = 0.01) of (a) **2**$^-$-TATA$^+$, (b) **2**$^-$, and (c) TATA$^+$ in the single-crystal X-ray structure (Fig. S26) calculated at B3LYP/6-31+G(d,p).

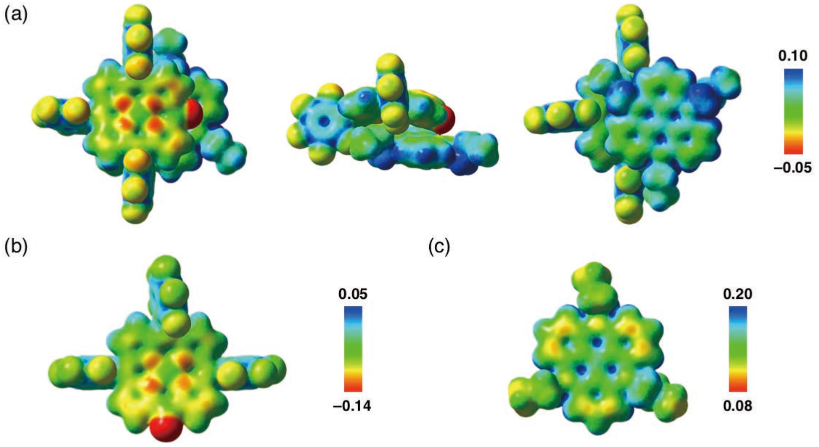

Fig. S75 Electron density diagrams (top, side, and bottom views, δ = 0.01) of (a) **2ni**$^{-}$-TATA^{+}, (b) **2ni**$^{-}$, and (c) TATA^{+} in the single-crystal X-ray structure (Fig. S28) calculated at B3LYP/6-31+G(d,p) for C, H, N, O, and F and B3LYP/LanL2DZ for Ni.

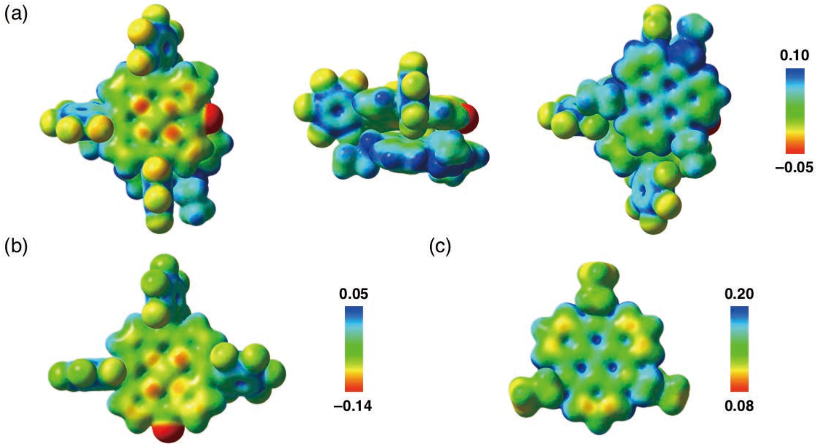

Fig. S76 Electron density diagrams (top, side, and bottom views, δ = 0.01) of (a) **2pd**$^{-}$-TATA^{+}, (b) **2pd**$^{-}$, and (c) TATA^{+} in the single-crystal X-ray structure (Fig. S30) calculated at B3LYP/6-31+G(d,p) for C, H, N, O, and F and B3LYP/LanL2DZ for Pd.

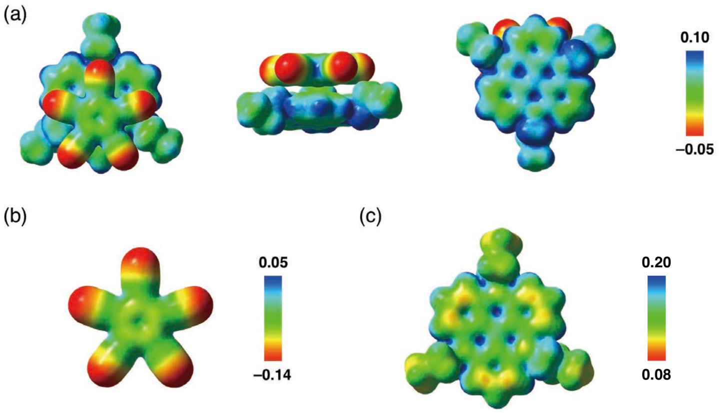

Fig. S77 Electron density diagrams (top, side, and bottom views, δ = 0.01) of (a) TATA^{+}-PCCp^{-}, (b) PCCp^{-}, and (c) TATA^{+} in the single-crystal X-ray structure[S12] calculated at B3LYP/6-31+G(d,p).

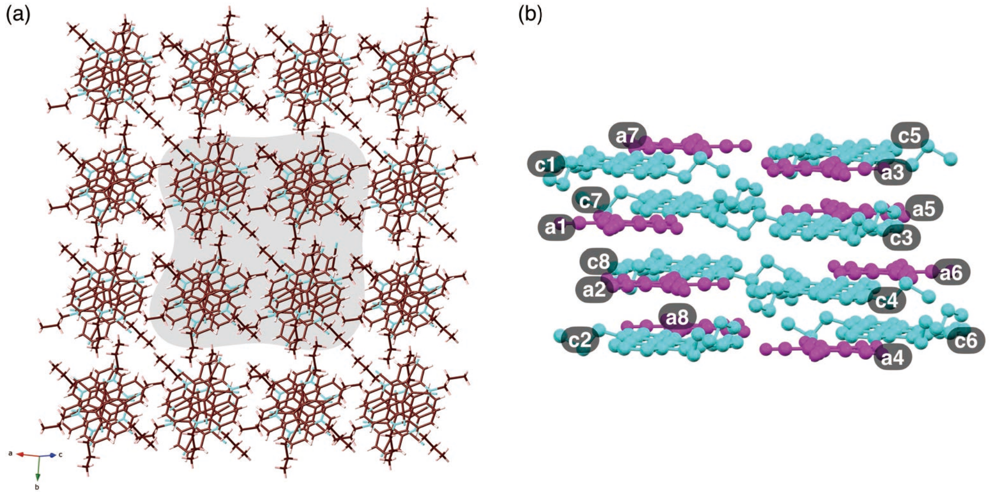

Fig. S78 Single-crystal X-ray structure of PCCp^{-}-TATA^{+}[S12] for the energy decomposition analysis (EDA) calculation (Table S4): (a) top view of charge-by-charge structure and (b) side view of shaded part in (a). The labels (a1–8 and c1–8) correspond to the fragments shown in Table S4. E_{tot} of stacking ion pairs of PCCp^{-}-TATA^{+} were –138.8 – –141.3 kcal/mol, which were larger than those of lateral positions (–34.4 – –63.2 kcal/mol). Importantly, E_{disp} for the stacking ion pairs were –95.03 – –95.54 kcal/mol, which were larger than those of lateral positions. Favorable contribution of E_{disp} for the stacking ion pairs indicated effective $^{i}\pi$–$^{i}\pi$ interaction. It should be noticed that energetically unfavored E_{es} and favored E_{disp} were observed in the stacking structures of PCCp^{-} (E_{es}/E_{disp} of 43.18/–43.92 and 43.21/–43.92 kcal/mol for two different structures a1/a2 and a5/a6, respectively) and TATA^{+} (E_{es}/E_{disp} of 35.12/–133.40 and 35.00/–133.40 kcal/mol for two different structures c3/c4 and c7/c8, respectively). The stacking structures of PCCp^{-} are less stable than those of TATA^{+}. Atom color code in (a): brown, pink, and cyan refer to carbon, hydrogen, and nitrogen, respectively. Color code in (b): magenta and cyan refer to anion and cation parts, respectively.

Table S4 Energies between each fragment in PCCp⁻-TATA⁺ (Fig. S78) estimated by EDA calculation[S15] based on an FMO2-MP2 using NOSeC-V-DZP with MCP.[S16–18]

fragments	total interaction energy (kcal/mol)	electrostatic interaction energy (E_{es}) (kcal/mol)	exchange repulsion interaction energy (E_{ex}) (kcal/mol)	charge-transfer interaction energy (E_{ct+mix}) (kcal/mol)	dispersion interaction energy (E_{disp}) (kcal/mol)
c8-c1	30.756	30.756	0.000	0.000	0.000
c5-c1	20.742	24.625	0.903	–0.313	–4.473
c5-c8	25.965	25.965	0.000	0.000	0.000
c4-c1	20.858	20.858	0.000	0.000	0.000
c4-c8	21.541	21.541	0.000	0.000	0.000
c4-c5	21.513	21.513	0.000	0.000	0.000
c7-c1	6.872	34.317	3.787	–2.314	–28.918
c7-c8	–84.166	35.005	24.617	–10.387	–133.400
c7-c5	8.778	31.317	2.388	–1.493	–23.435
c7-c4	18.554	18.554	0.000	0.000	0.000
c2-c1	24.247	24.247	0.000	0.000	0.000
c2-c8	20.533	29.801	0.276	–0.576	–8.968
c2-c5	17.614	17.614	0.000	0.000	0.000
c2-c4	8.787	31.329	2.390	–1.492	–23.439
c2-c7	21.532	21.532	0.000	0.000	0.000
c3-c1	20.105	25.387	0.373	–0.639	–5.016
c3-c8	22.984	22.984	0.000	0.000	0.000
c3-c5	20.453	29.747	0.276	–0.578	–8.992
c3-c4	–84.042	35.117	24.621	–10.385	–133.395
c3-c7	21.523	21.523	0.000	0.000	0.000
c3-c2	25.988	25.988	0.000	0.000	0.000
c6-c1	17.636	17.636	0.000	0.000	0.000
c6-c8	20.076	25.334	0.373	–0.638	–4.993
c6-c5	24.208	24.208	0.000	0.000	0.000
c6-c4	6.822	34.282	3.788	–2.315	–28.933
c6-c7	20.827	20.827	0.000	0.000	0.000
c6-c2	20.747	24.632	0.898	–0.312	–4.471
c6-c3	30.718	30.718	0.000	0.000	0.000
a7-c1	–63.153	–44.943	2.667	–2.551	–18.326
a7-c8	–34.050	–34.050	0.000	0.000	0.000
a7-c5	–55.531	–39.392	1.389	–2.003	–15.525
a7-c4	–16.791	–16.791	0.000	0.000	0.000
a7-c7	–141.336	–58.976	21.672	–8.490	–95.541
a7-c2	–18.536	–18.536	0.000	0.000	0.000
a7-c3	–19.934	–19.934	0.000	0.000	0.000
a7-c6	–18.119	–18.119	0.000	0.000	0.000
a2-c1	–32.155	–32.155	0.000	0.000	0.000
a2-c8	–54.377	–38.402	2.395	–2.460	–15.910
a2-c5	–20.318	–20.318	0.000	0.000	0.000
a2-c4	–55.113	–38.901	1.340	–2.003	–15.549
a2-c7	–28.293	–26.102	0.000	–0.289	–1.902
a2-c2	–139.245	–57.571	21.734	–8.187	–95.221
a2-c3	–43.151	–32.439	0.684	–0.859	–10.538
a2-c6	–23.828	–23.828	0.000	0.000	0.000
a2-a7	22.066	22.066	0.000	0.000	0.000
a3-c1	–33.760	–27.883	2.005	–1.026	–6.856
a3-c8	–20.922	–20.922	0.000	0.000	0.000
a3-c5	–52.093	–36.030	2.288	–2.432	–15.919
a3-c4	–33.595	–33.595	0.000	0.000	0.000
a3-c7	–20.992	–20.992	0.000	0.000	0.000
a3-c2	–22.077	–22.077	0.000	0.000	0.000
a3-c3	–140.879	–58.667	21.821	–8.593	–95.440
a3-c6	–24.765	–24.765	0.000	0.000	0.000
a3-a7	20.613	20.613	0.000	0.000	0.000
a3-a2	26.866	26.866	0.000	0.000	0.000
a6-c1	–19.754	–19.754	0.000	0.000	0.000
a6-c8	–34.362	–28.669	2.204	–1.084	–6.813
a6-c5	–32.064	–32.064	0.000	0.000	0.000
a6-c4	–59.992	–41.677	2.518	–2.463	–18.370
a6-c7	–24.213	–24.213	0.000	0.000	0.000
a6-c2	–22.836	–22.836	0.000	0.000	0.000
a6-c3	–61.996	–40.626	2.303	–1.587	–22.086
a6-c6	–139.150	–57.574	21.785	–8.315	–95.046
a6-a7	21.117	21.117	0.000	0.000	0.000
a6-a2	24.252	24.252	0.000	0.000	0.000
a6-a3	30.720	30.720	0.000	0.000	0.000
a1-c1	–138.826	–57.276	21.807	–8.322	–95.034

Table S4 (Continued)

fragments	total interaction energy (kcal/mol)	electrostatic interaction energy (E_{es}) (kcal/mol)	exchange repulsion interaction energy (E_{ex}) (kcal/mol)	charge-transfer interaction energy ($E_{ct + mix}$) (kcal/mol)	dispersion interaction energy (E_{disp}) (kcal/mol)
a1-c8	–61.219	–39.859	2.303	–1.571	–22.092
a1-c5	–22.632	–22.632	0.000	0.000	0.000
a1-c4	–24.522	–24.522	0.000	0.000	0.000
a1-c7	–58.734	–40.478	2.525	–2.440	–18.340
a1-c2	–32.519	–32.519	0.000	0.000	0.000
a1-c3	–34.749	–29.034	2.200	–1.094	–6.820
a1-c6	–19.748	–19.748	0.000	0.000	0.000
a1-a7	31.360	31.360	0.000	0.000	0.000
a1-a2	4.391	43.180	8.581	–3.456	–43.915
a1-a3	25.913	25.913	0.000	0.000	0.000
a1-a6	21.330	21.330	0.000	0.000	0.000
a8-c1	–24.926	–24.926	0.000	0.000	0.000
a8-c8	–140.619	–58.461	21.848	–8.564	–95.442
a8-c5	–22.044	–22.044	0.000	0.000	0.000
a8-c4	–21.352	–21.352	0.000	0.000	0.000
a8-c7	–33.281	–33.281	0.000	0.000	0.000
a8-c2	–53.368	–37.238	2.281	–2.462	–15.948
a8-c3	–21.187	–21.187	0.000	0.000	0.000
a8-c6	–34.119	–28.230	2.004	–1.037	–6.857
a8-a7	26.690	26.690	0.000	0.000	0.000
a8-a2	27.676	32.725	0.023	–0.337	–4.734
a8-a3	18.920	18.920	0.000	0.000	0.000
a8-a6	25.921	25.921	0.000	0.000	0.000
a8-a1	30.713	30.713	0.000	0.000	0.000
a5-c1	–23.955	–23.955	0.000	0.000	0.000
a5-c8	–43.031	–32.316	0.684	–0.857	–10.541
a5-c5	–138.818	–57.170	21.759	–8.180	–95.226
a5-c4	–28.893	–26.713	0.000	–0.290	–1.890
a5-c7	–54.553	–38.370	1.347	–1.996	–15.534
a5-c2	–20.560	–20.560	0.000	0.000	0.000
a5-c3	–55.665	–39.623	2.386	–2.494	–15.935
a5-c6	–32.572	–32.572	0.000	0.000	0.000
a5-a7	31.288	31.288	0.000	0.000	0.000
a5-a2	23.289	23.289	0.000	0.000	0.000
a5-a3	27.653	32.706	0.023	–0.338	–4.738
a5-a6	4.419	43.211	8.581	–3.456	–43.919
a5-a1	24.227	24.227	0.000	0.000	0.000
a5-a8	26.857	26.857	0.000	0.000	0.000
a4-c1	–17.961	–17.961	0.000	0.000	0.000
a4-c8	–19.625	–19.625	0.000	0.000	0.000
a4-c5	–18.268	–18.268	0.000	0.000	0.000
a4-c4	–140.975	–58.688	21.678	–8.458	–95.506
a4-c7	–16.562	–16.562	0.000	0.000	0.000
a4-c2	–55.029	–38.908	1.394	–1.994	–15.522
a4-c3	–33.720	–33.720	0.000	0.000	0.000
a4-c6	–61.770	–43.630	2.693	–2.522	–18.311
a4-a7	14.979	14.979	0.000	0.000	0.000
a4-a2	31.300	31.300	0.000	0.000	0.000
a4-a3	26.676	26.676	0.000	0.000	0.000
a4-a6	31.355	31.355	0.000	0.000	0.000
a4-a1	21.112	21.112	0.000	0.000	0.000
a4-a8	20.625	20.625	0.000	0.000	0.000
a4-a5	22.071	22.071	0.000	0.000	0.000

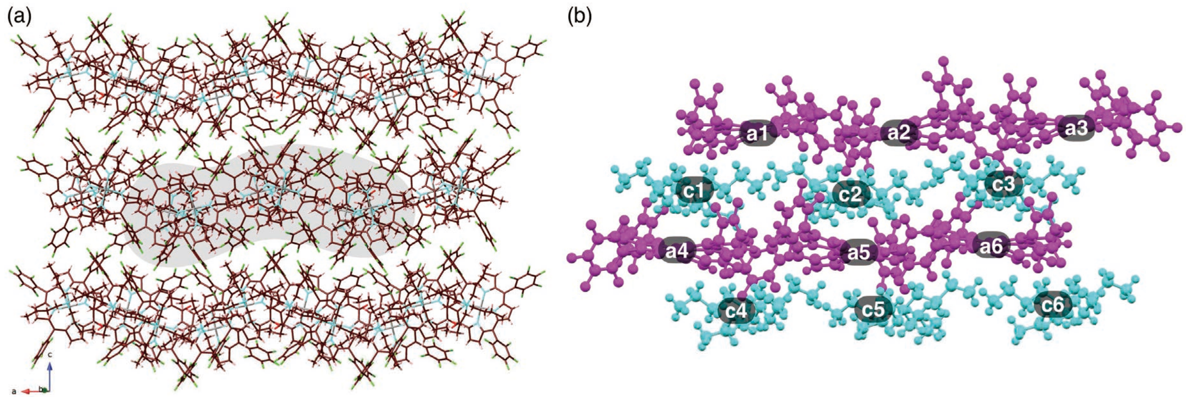

Fig. S79 Single-crystal X-ray structure of **2ni**$^-$-TBA$^+$[S5b] for the EDA calculation (Table S5): (a) top view of charge-by-charge structure and (b) side view of shaded part in (a). The labels (a1–6 and c1–6) correspond to the fragments shown in Table S5. E_{tot} of –169.0 and –129.1 kcal/mol were observed for charge-by-charge stacking ion pairs of a5-c2 and a5-c5, respectively, with the contributions of E_{es}/E_{disp} of –68.34/–111.1 and –68.08/–67.66 kcal/mol, respectively. E_{tot} of –83.34 and –23.77 kcal/mol were observed for a5-c4 and a5-c6 ion pairs, respectively, indicating the smaller attractive interaction energies compared to charge-by-charge stacking ion pairs (a5-c2 and a5-c5). On the other hand, identically charged ions c1-c2 was in energetically disfavored state, which is mainly contributed by the electrostatic repulsion. Atom color code in (a): brown, pink, cyan, red, green, and gray refer to carbon, hydrogen, nitrogen, oxygen, fluorine, and nickel, respectively. Color code in (b): magenta and cyan refer to anion and cation parts, respectively.

Table S5 Energies between each fragment in **2ni**$^{-}$-TBA^{+} (Fig. S79) estimated by EDA calculation[S15] based on an FMO2-MP2 using mixed basis sets including NOSeC-V-TZP with MCP for Ni and NOSeC-V-DZP with MCP for the other atoms.[S16–18]

fragments	total interaction energy (kcal/mol)	electrostatic interaction energy (E_{es}) (kcal/mol)	exchange repulsion interaction energy (E_{ex}) (kcal/mol)	charge-transfer interaction energy (E_{ct+mix}) (kcal/mol)	dispersion interaction energy (E_{disp}) (kcal/mol)
a4-a2	22.065	22.065	0.000	0.000	0.000
a6-a2	10.828	24.888	0.783	–0.536	–14.307
a6-a4	15.251	15.251	0.000	0.000	0.000
a1-a2	–28.917	20.130	10.464	–5.834	–53.677
a1-a4	30.520	36.652	0.003	–0.039	–6.095
a1-a6	15.233	15.233	0.000	0.000	0.000
a3-a2	–29.662	19.400	10.491	–5.855	–53.698
a3-a4	13.407	13.407	0.000	0.000	0.000
a3-a6	30.226	36.355	0.003	–0.036	–6.095
a3-a1	15.366	15.366	0.000	0.000	0.000
a5-a2	30.335	36.442	0.003	–0.042	–6.067
a5-a4	–29.948	19.170	10.365	–5.832	–53.652
a5-a6	–29.531	19.546	10.358	–5.830	–53.606
a5-a1	11.054	25.127	0.785	–0.532	–14.325
a5-a3	22.019	22.019	0.000	0.000	0.000
c1-a2	–23.508	–23.508	0.000	0.000	0.000
c1-a4	–169.357	–68.762	20.642	–10.156	–111.082
c1-a6	–14.100	–14.100	0.000	0.000	0.000
c1-a1	–127.824	–67.325	13.298	–6.307	–67.489
c1-a3	–13.348	–13.348	0.000	0.000	0.000
c1-a5	–45.464	–23.902	2.669	–1.845	–22.385
c3-a2	–82.284	–38.233	7.406	–4.157	–47.300
c3-a4	–15.590	–15.590	0.000	0.000	0.000
c3-a6	–169.298	–68.621	20.699	–10.374	–111.001
c3-a1	–17.137	–17.137	0.000	0.000	0.000
c3-a3	–127.868	–67.524	13.434	–6.320	–67.458
c3-a5	–29.803	–29.803	0.000	0.000	0.000
c3-c1	15.079	15.079	0.000	0.000	0.000
c5-a2	–25.650	–25.650	0.000	0.000	0.000
c5-a4	–23.869	–23.869	0.000	0.000	0.000
c5-a6	–83.724	–38.690	7.429	–4.759	–47.703
c5-a1	–19.306	–19.306	0.000	0.000	0.000
c5-a3	–20.960	–20.960	0.000	0.000	0.000
c5-a5	–129.072	–68.075	13.449	–6.784	–67.663
c5-c1	19.316	19.316	0.000	0.000	0.000
c5-c3	28.870	28.870	0.000	0.000	0.000
c2-a2	–127.911	–67.512	13.417	–6.352	–67.464
c2-a4	–30.136	–30.136	0.000	0.000	0.000
c2-a6	–45.776	–24.223	2.667	–1.848	–22.372
c2-a1	–83.524	–39.398	7.359	–4.170	–47.314
c2-a3	–23.549	–23.549	0.000	0.000	0.000
c2-a5	–168.999	–68.335	20.730	–10.306	–111.088
c2-c1	22.386	28.973	1.300	–0.591	–7.296
c2-c3	22.146	28.728	1.301	–0.587	–7.296
c2-c5	35.089	35.089	0.000	0.000	0.000
c4-a2	–20.859	–20.859	0.000	0.000	0.000
c4-a4	–129.630	–68.646	13.490	–6.800	–67.674
c4-a6	–16.956	–16.956	0.000	0.000	0.000
c4-a1	–25.677	–25.677	0.000	0.000	0.000
c4-a3	–13.725	–13.725	0.000	0.000	0.000
c4-a5	–83.335	–38.333	7.425	–4.732	–47.695
c4-c1	34.882	34.882	0.000	0.000	0.000
c4-c3	16.515	16.515	0.000	0.000	0.000
c4-c5	22.707	29.293	1.290	–0.588	–7.288
c4-c2	28.843	28.843	0.000	0.000	0.000
c6-a2	–19.171	–19.171	0.000	0.000	0.000
c6-a4	–13.453	–13.453	0.000	0.000	0.000
c6-a6	–128.748	–67.683	13.354	–6.745	–67.674
c6-a1	–12.897	–12.897	0.000	0.000	0.000
c6-a3	–25.912	–25.912	0.000	0.000	0.000
c6-a5	–23.770	–23.770	0.000	0.000	0.000
c6-c1	12.287	12.287	0.000	0.000	0.000
c6-c3	35.849	35.849	0.000	0.000	0.000
c6-c5	22.705	29.289	1.292	–0.589	–7.287
c6-c2	19.372	19.372	0.000	0.000	0.000

Table S5 (Continued)

fragments	total interaction energy (kcal/mol)	electrostatic interaction energy (E_{es}) (kcal/mol)	exchange repulsion interaction energy (E_{ex}) (kcal/mol)	charge-transfer interaction energy (E_{ct+mix}) (kcal/mol)	dispersion interaction energy (E_{disp}) (kcal/mol)
c6-c4	15.173	15.173	0.000	0.000	0.000

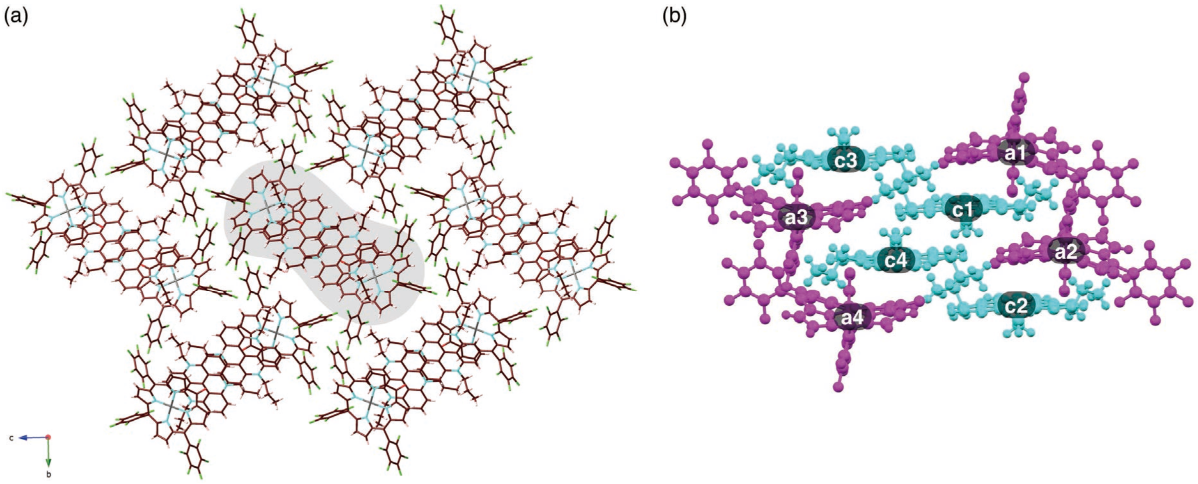

Fig. S80 Single-crystal X-ray structure of **2ni**$^{-}$-TATA^{+} for the EDA calculation (Table S6): (a) top view of charge-by-charge structure and (b) side view of shaded part in (a). The labels (a1–4 and c1–4) correspond to the fragments shown in Table S6. E_{tot} of the oppositely charged stacking ion pair a2-c2 was –196.2 kcal/mol, whose absolute value was larger than that of the ion pair located at the lateral positions (–63.42 kcal/mol for a2-c4). The main contributions in attractive forces in **2ni**$^{-}$-TATA^{+} were E_{es} and E_{disp} with the values of –69.72 and –141.9 kcal/mol, respectively, for the ion pair a2-c2. The E_{es} values were rather similar to the values observed in the charge-by-charge stacking ion pairs of **2ni**$^{-}$-TBA^{+} (Fig. S79). On the other hand, characteristic large E_{disp} contribution can be attributed to the overlap of π-planes. E_{disp} values (absolute values) of a2-c1 (–63.30 kcal/mol) and a2-c4 (–21.14 kcal/mol) are smaller than that of a2-c2 due to the less or no overlapping of core π-planes. Similarly, the stacking of TATA^{+} in **2ni**$^{-}$-TATA^{+} (c1-c4) is stabilized by the contribution of dispersion interaction. These results strongly support that dispersion interaction is a key for controlling the locations of identically charged species. Atom color code in (a): brown, pink, cyan, red, green, and gray refer to carbon, hydrogen, nitrogen, oxygen, fluorine, and nickel, respectively. Color code in (b): magenta and cyan refer to anion and cation parts, respectively.

Table S6 Energies between each fragment in **2ni**$^-$-TATA$^+$ (Fig. S80) estimated by EDA calculation[S15] based on an FMO2-MP2 using mixed basis sets including NOSeC-V-TZP with MCP for Ni and NOSeC-V-DZP with MCP for the other atoms.[S16–18]

fragments	total interaction energy (kcal/mol)	electrostatic interaction energy (E_{es}) (kcal/mol)	exchange repulsion interaction energy (E_{ex}) (kcal/mol)	charge-transfer interaction energy (E_{ct+mix}) (kcal/mol)	dispersion interaction energy (E_{disp}) (kcal/mol)
a2-a1	–2.562	34.854	3.746	–2.166	–38.995
a3-a1	33.586	33.586	0.000	0.000	0.000
a3-a2	22.760	22.760	0.000	0.000	0.000
a4-a1	25.937	25.937	0.000	0.000	0.000
a4-a2	33.581	33.581	0.000	0.000	0.000
a4-a3	–2.649	34.769	3.750	–2.167	–39.001
c1-a1	–192.461	–66.417	28.999	–13.342	–141.701
c1-a2	–111.808	–53.658	11.406	–6.254	–63.302
c1-a3	–63.177	–43.060	3.401	–2.387	–21.130
c1-a4	–35.983	–32.530	0.000	–0.578	–2.876
c2-a1	–27.529	–27.529	0.000	0.000	0.000
c2-a2	–196.168	–69.723	29.491	–14.044	–141.892
c2-a3	–22.086	–22.086	0.000	0.000	0.000
c2-a4	–64.848	–44.270	3.523	–2.850	–21.251
c2-c1	34.075	34.075	0.000	0.000	0.000
c3-a1	–65.097	–44.511	3.524	–2.856	–21.253
c3-a2	–22.136	–22.136	0.000	0.000	0.000
c3-a3	–195.919	–69.473	29.487	–14.035	–141.898
c3-a4	–27.478	–27.478	0.000	0.000	0.000
c3-c1	0.096	34.713	7.282	–4.118	–37.781
c3-c2	20.200	20.200	0.000	0.000	0.000
c4-a1	–36.139	–32.682	0.000	–0.577	–2.880
c4-a2	–63.417	–43.291	3.401	–2.392	–21.135
c4-a3	–111.700	–53.547	11.405	–6.254	–63.304
c4-a4	–192.210	–66.166	28.995	–13.332	–141.708
c4-c1	–47.122	33.269	18.101	–7.466	–91.026
c4-c2	0.106	34.725	7.281	–4.116	–37.784
c4-c3	34.068	34.068	0.000	0.000	0.000

Table S7 Energies between anion and cation for monomeric ion pairs extracted from the crystal structures **2ni**$^-$-TBA$^+$[S5b] and **2ni**$^-$-TATA$^+$ (Fig. S28) estimated by EDA calculation[S15] based on an FMO2-MP2 using mixed basis sets including NOSeC-V-TZP with MCP for Ni and NOSeC-V-DZP with MCP for the other atoms.[S16–18] The most stable monomeric ion pairs, c3-a6 and c2-a2 for **2ni**$^-$-TBA$^+$ and **2ni**$^-$-TATA$^+$, respectively, were selected according to the EDA calculations of packing structures (Fig. S79,80, Table S5,6). E_{tot} values and those energy contributions of monomeric ion pairs were similar to those of the packing structures, suggesting the negligible effect of the surrounding ions.

fragments	total interaction energy (kcal/mol)	electrostatic interaction energy (E_{es}) (kcal/mol)	exchange repulsion interaction energy (E_{ex}) (kcal/mol)	charge-transfer interaction energy (E_{ct+mix}) (kcal/mol)	dispersion interaction energy (E_{disp}) (kcal/mol)
**2ni**$^-$-TBA$^+$	–175.081	–74.176	20.558	–10.289	–111.175
**2ni**$^-$-TATA$^+$	–196.473	–70.294	29.058	–13.489	–141.749

Table S8 Energies between anion and cation for optimized monomeric ion pairs **2ni**$^-$-TBA$^+$ and **2ni**$^-$-TATA$^+$ (Fig. S44) estimated by EDA calculation[S15] based on an FMO2-MP2 using mixed basis sets including NOSeC-V-TZP with MCP for Ni and NOSeC-V-DZP with MCP for the other atoms.[S16–18] E_{tot} values (absolute values) of optimized monomeric ion pairs were larger than those of monomeric (Table S7) and packing ion pairs (Fig. S79,80, Table S5,6) in the crystal structures.

fragments	total interaction energy (kcal/mol)	electrostatic interaction energy (E_{es}) (kcal/mol)	exchange repulsion interaction energy (E_{ex}) (kcal/mol)	charge-transfer interaction energy (E_{ct+mix}) (kcal/mol)	dispersion interaction energy (E_{disp}) (kcal/mol)
**2ni**$^-$-TBA$^+$	–204.804	–90.129	52.871	–18.296	–149.250
**2ni**$^-$-TATA$^+$	–229.567	–88.573	62.514	–22.185	–181.323

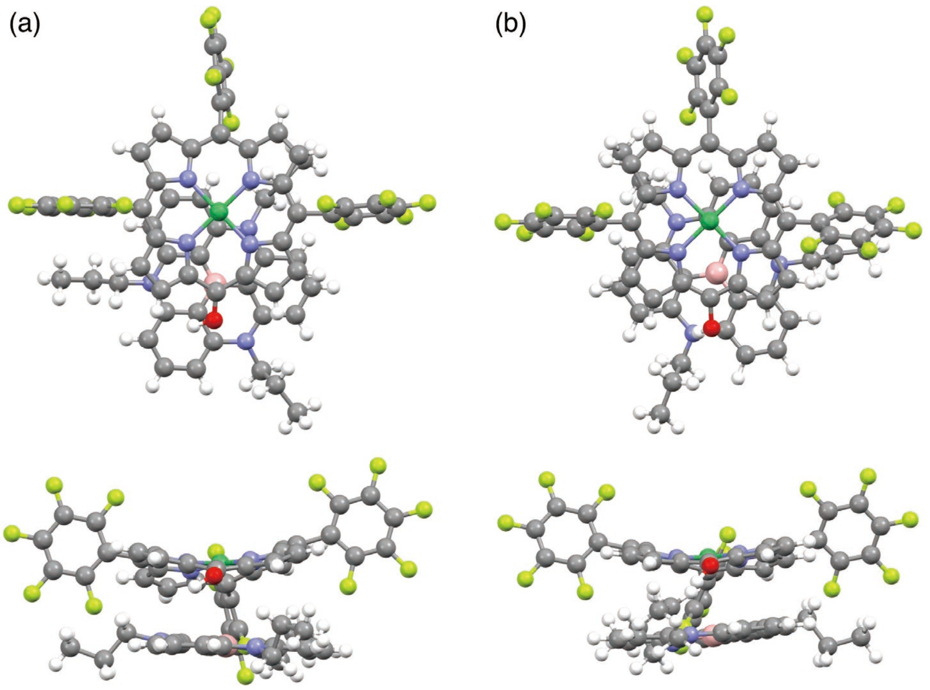

Fig. S81 2ni-TATAB as a model of electronically neutral species for **2ni**$^{–}$-TATA^{+} for EDA calculation:[S15] (a) unoptimized and (b) optimized structures. Model structure of **2ni**-TATAB was prepared based on the crystal structure of monomeric **2ni**$^{–}$-TATA^{+} (Fig. S28), wherein the oxygen of **2ni**$^{–}$ was protonated and the central carbon of TATA^{+} was substituted to boron. In (a), the position of the proton at oxygen was optimized using B3LYP/6-31G(d) with the fixed coordination for the other atoms, whereas, in (b), the structure was optimized using B3LYP/6-31G(d,p).

Table S9 Energies between **2ni** and TATAB as unoptimized and optimized structures (Fig. S80) estimated by EDA calculation[S15] based on an FMO2-MP2 using mixed basis sets including NOSeC-V-TZP with MCP for Ni and NOSeC-V-DZP with MCP for the other atoms.[S16–18] E_{tot} (absolute value) of **2ni**-TATAB was smaller than that of monomeric **2ni**$^{–}$-TATA^{+} (Table S7,8) and packing **2ni**$^{–}$-TATA^{+} (Fig. S80, Table S6). In particular, E_{es} (absolute value) of **2ni**-TATAB was smaller than that of **2ni**$^{–}$-TATA^{+}, whereas E_{disp} of **2ni**-TATAB was similar to **2ni**$^{–}$-TATA^{+}. The loss of charges induced a less stable structure with the decreased electrostatic interaction along with the similar dispersion interaction.

fragments	total interaction energy (kcal/mol)	electrostatic interaction energy (E_{es}) (kcal/mol)	exchange repulsion interaction energy (E_{ex}) (kcal/mol)	charge-transfer interaction energy (E_{ct+mix}) (kcal/mol)	dispersion interaction energy (E_{disp}) (kcal/mol)
**2ni**-TATAB (unoptimized)	–149.135	–24.975	38.969	–19.030	–144.099
**2ni**-TATAB (optimized)	–168.533	–29.679	59.887	–23.222	–175.519

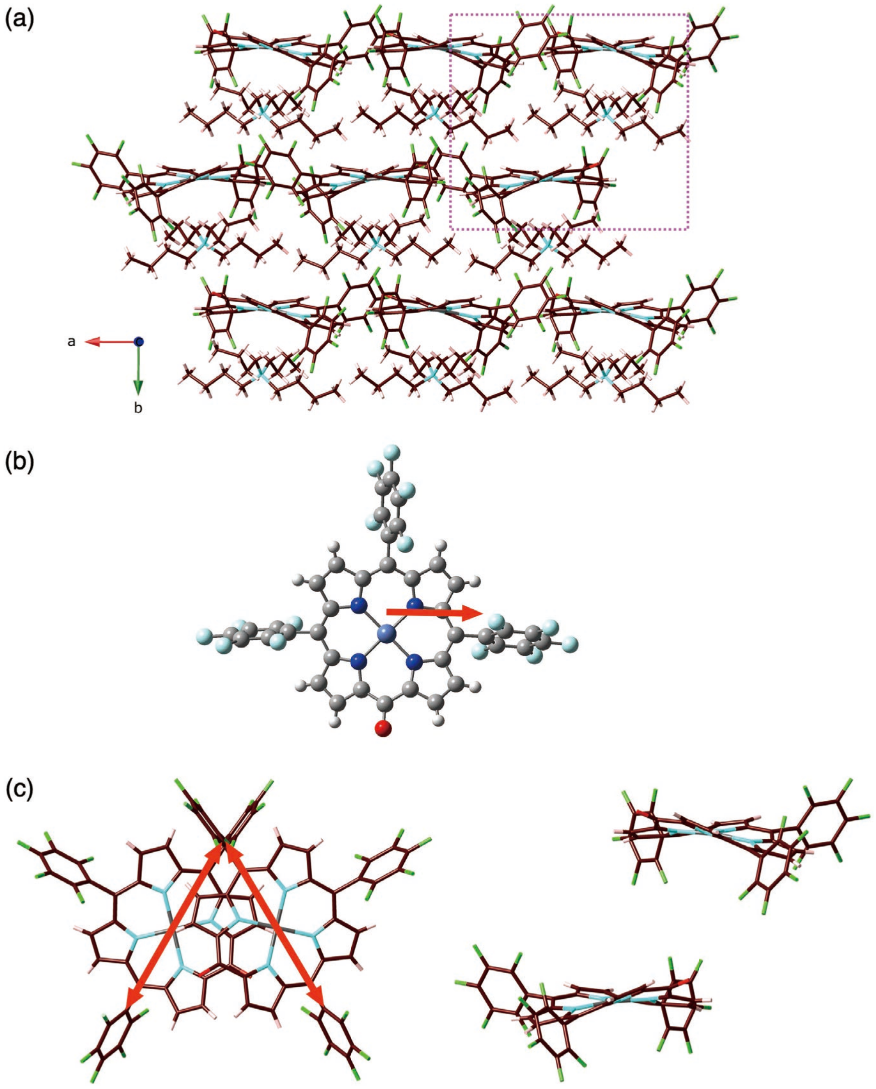

Fig. S82 Evaluation of the transition dipole moments for the solid-state **2ni**$^{-}$-TBA^{+}:[S5b] (a) packing structure of **2ni**$^{-}$-TBA^{+}, (b) ground-to-excited-state transition dipole moments of **2ni**$^{-}$ for 650 nm (HOMO–1-to-LUMO+1 and HOMO-to-LUMO transition with the strength of 4.62 D) estimated by TD-DFT calculation at B3LYP/6-31+G(d,p) of the crystal structure, and (c) oblique-arranged two **2ni**$^{-}$ extracted from the magenta boxed area in (a). In (c), red arrows indicate direction-extended transition dipole moments with a consideration of the symmetry of structures. Nearly orthogonally arranged transition dipoles provide an almost negligible effect on the UV/vis absorption spectrum (Fig. S96a).[S19]

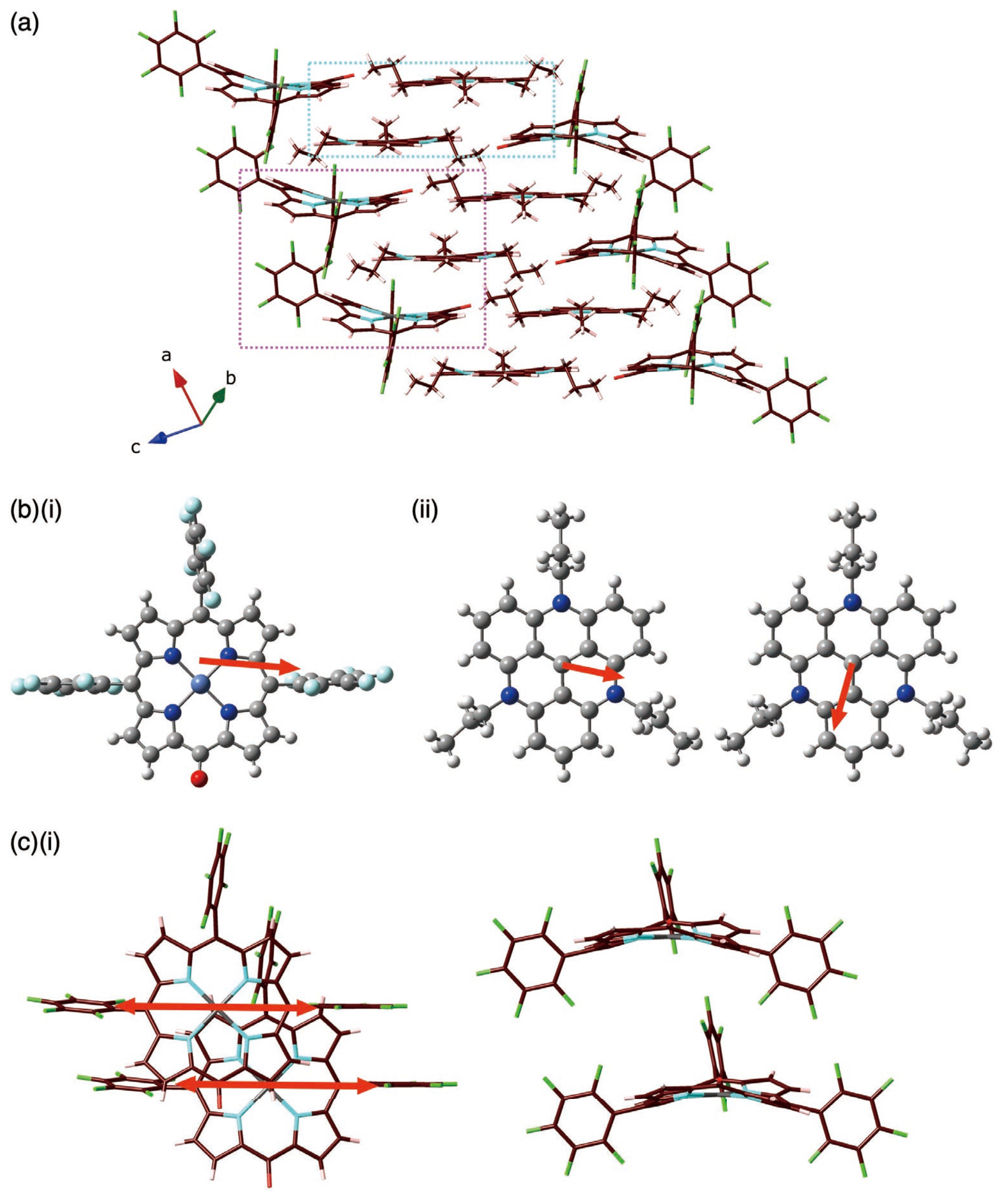

Fig. S83 Evaluation of the transition dipole moments for the solid-state **2ni**$^-$-TATA$^+$: (a) packing structure of **2ni**$^-$-TATA$^+$ (Fig. S28), (b) ground-to-excited-state transition dipole moments of (i) **2ni**$^-$ for 662 nm (HOMO–1-to-LUMO+1 and HOMO-to-LUMO transition with the strength of 4.80 D) and (ii) TATA$^+$ for 472 and 462 nm (state 1 and 2 for HOMO-to-LUMO and HOMO–1-to-LUMO transitions with the strengths of 2.79 and 3.10 D, respectively) estimated by TD-DFT calculation at B3LYP/6-31+G(d,p) of the crystal structure (Fig. S29), and (c)(i) side-by-side-arranged two **2ni**$^-$ and (ii) slipped-stacking dimeric structure of TATA$^+$ extracted from the cyan boxed area in (a). In (c)(i), red arrows indicate direction-extended transition dipole moments with a consideration of the symmetry of structures. The **2ni**$^-$ units, side-by-side-arranged in the packing structure, showed the parallel-arranged transition dipole moments with the offset angle of 75°. The distance of ~7 Å between two **2ni**$^-$ units is within the range of distances that induce the shift of absorption spectrum by exciton coupling.[S19] The parallel-arranged transition dipole moments showed blue-shifted absorption bands for **2ni**$^-$ in the solid-state absorption spectrum of **2ni**$^-$-TATA$^+$ (Fig. S96b). In (c)(ii), red arrows indicate representative transition dipole moments with a consideration of the symmetry of structures as TATA$^+$ has a centrosymmetric structure with six directions of symmetrical units. Therefore, the transition dipole moments of TATA$^+$ in state 1 can be superimposed on the slipped-stacking dimeric structure with the approximate offset angle of ~40°. In contrast, the transition dipole moments of TATA$^+$ in state 2 are canceled out in the dimeric structure with the anti-parallel arrangement. To sum up, the head-to-tail-arranged transition dipole moments of TATA$^+$ induced red-shifted absorption bands for TATA$^+$ in the solid-state absorption spectrum of **2ni**$^-$-TATA$^+$ (Fig. S96b).

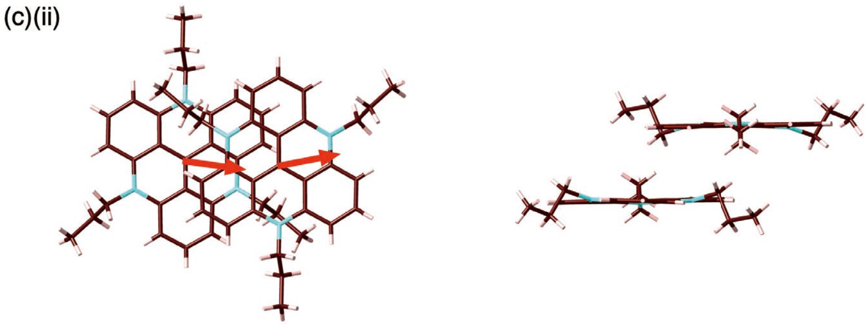

Fig. S83 (Continued)

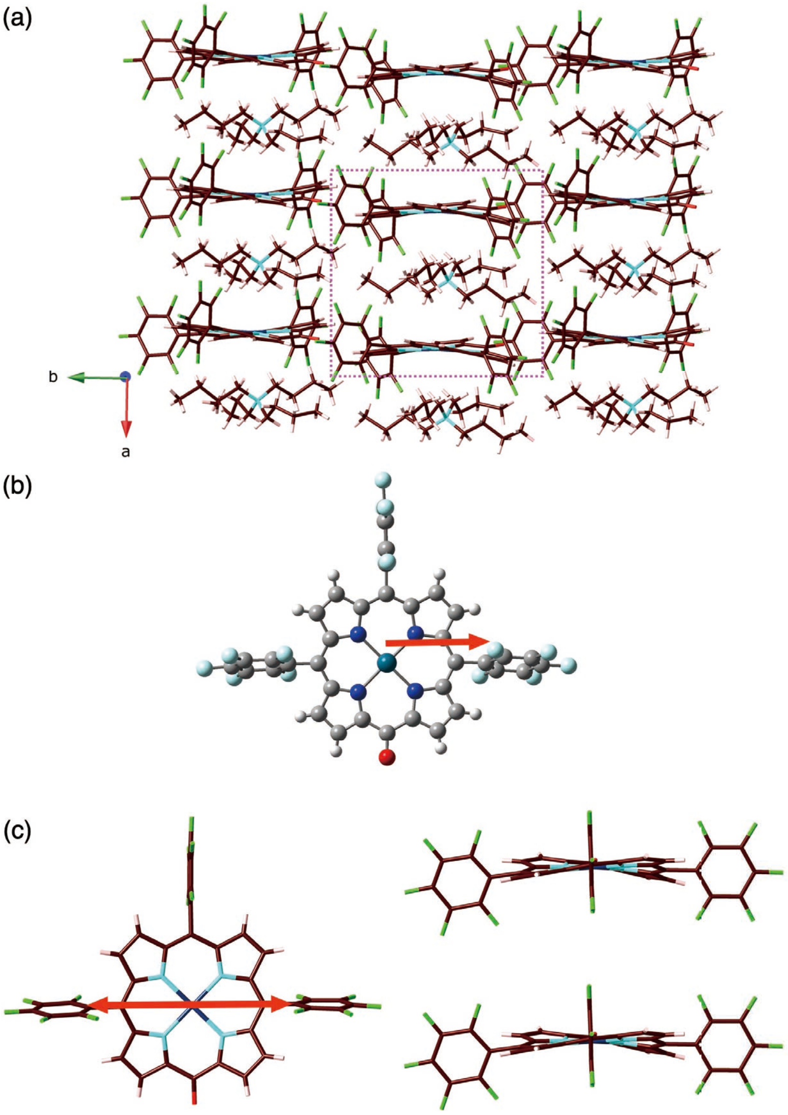

Fig. S84 Evaluation of the transition dipole moments for the solid-state **2pd**$^{-}$-TBA^{+}: (a) packing structure of **2pd**$^{-}$-TBA^{+} (Fig. S22), (b) ground-to-excited-state transition dipole moments of (i) **2pd**$^{-}$ for 654 nm (HOMO-to-LUMO transition with the strength of 5.01 D) estimated by TD-DFT calculation at B3LYP/6-31+G(d,p) of the crystal structure (Fig. S23), and (c) side-by-side-arranged two **2pd**$^{-}$ extracted from the magenta boxed area in (a). In (c), red arrows indicate direction-extended transition dipole moments with a consideration of the symmetry of structures. The **2pd**$^{-}$ units, with the side-by-side-arranged in the packing structure, showed the parallel-arranged transition dipole moments to the *a*-axis. The completely parallel-arranged transition dipole moments showed blue-shifted absorption bands for **2pd**$^{-}$ in the solid-state absorption spectrum of **2pd**$^{-}$-TBA^{+} (Fig. S96c).

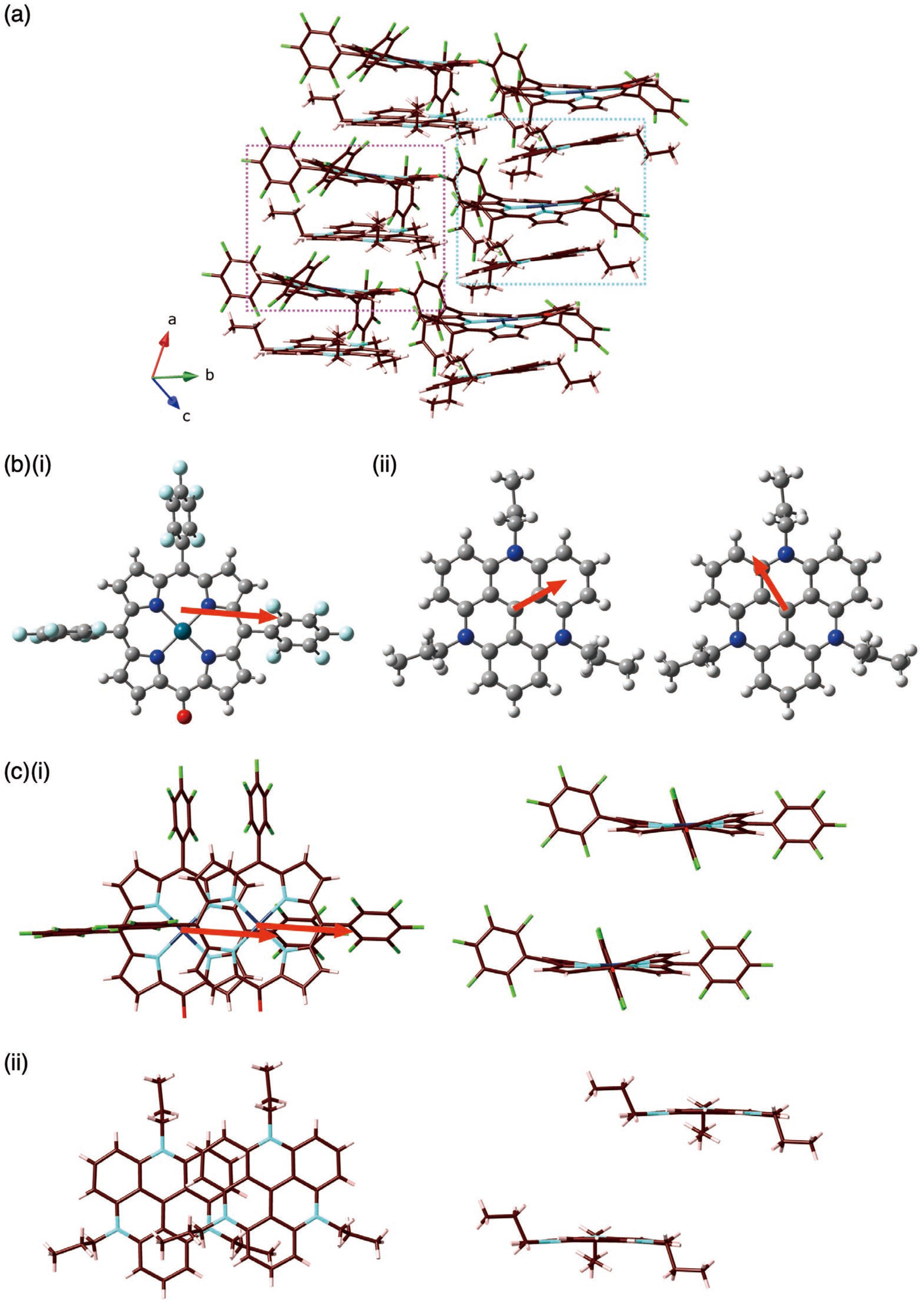

Fig. S85 Evaluation of the transition dipole moments for the solid-state **2pd**⁻-TATA⁺: (a) packing structure of **2pd**⁻-TATA⁺ (Fig. S30), (b) ground-to-excited-state transition dipole moments of (i) **2pd**⁻ for 678 nm (HOMO-to-LUMO transition with the strength of 5.15 D) and (ii) TATA⁺ for 455 and 448 nm (state 1 (left) and 2 (right) for HOMO-to-LUMO and HOMO–1-to-LUMO transitions with the strengths of 2.81 and 3.06 D, respectively) estimated by TD-DFT calculation at B3LYP/6-31+G(d,p) of the crystal structure (Fig. S31), and (c)(i) side-by-side-arranged two **2pd**⁻ and (ii) slipped-stacking dimeric structure of TATA⁺ extracted from the magenta and cyan boxed areas, respectively, in (a). In (c)(i), the **2pd**⁻ units, side-by-side-arranged in the packing structure, showed the head-to-tail-arranged transition dipole moments with the offset angle of 60°. The transition dipole moments, even with the offset angle that is larger than 54.7°, showed slightly red-shifted absorption bands for **2pd**⁻ in the solid-state absorption spectrum of **2pd**⁻-TATA⁺ (Fig. S96d). In (c)(ii), several combinations of the transition dipole moments of TATA⁺ are arranged in orthogonal orientations. In addition, parallel-arranged and head-to-tail-arranged orientations are seen in the small transition dipole moments, compared to those of **2pd**⁻, with larger distances that are not effective for exciton coupling, resulting in the small shifts for TATA⁺ in the solid-state absorption spectrum of **2pd**⁻-TATA⁺ (Fig. S96d).

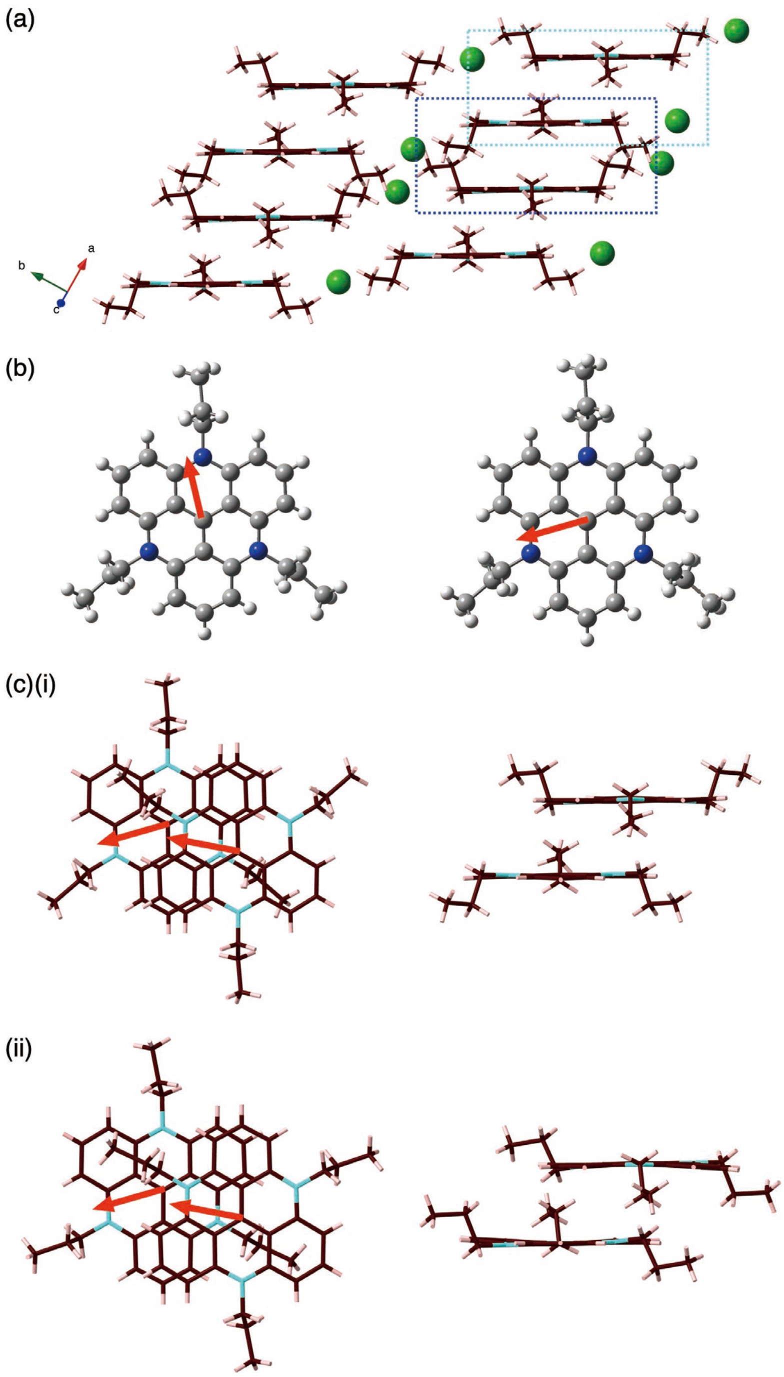

Fig. S86 Evaluation of the transition dipole moments for the solid-state TATA$^+$-Cl$^-$:[S4] (a) packing structure of TATA$^+$-Cl$^-$, (b) ground-to-excited-state transition dipole moments of TATA$^+$ for 456 and 450 nm (state 1 (left) and 2 (right) for HOMO-to-LUMO and HOMO–1-to-LUMO transitions, respectively) estimated by TD-DFT calculation at B3LYP/6-31+G(d,p) of the crystal structure, and (c) slipped-stacking structures of TATA$^+$ extracted from (i) the cyan boxed area and (ii) the blue boxed area in (a). In (c), red arrows indicate representative transition dipole moments with a consideration of the symmetry of structures as TATA$^+$ has a centrosymmetric structure with six directions of symmetrical units. Therefore, the transition dipole moments of TATA$^+$ in state 2 can be superimposed on the slipped-stacking dimeric structures with the approximate offset angles of ~50° (i) and ~40° (ii). In contrast, the transition dipole moments of TATA$^+$ in state 1 are canceled out in the dimeric structure with the anti-parallel arrangement. Similar to the discussion in Fig. S83, such head-to-tail-arranged transition dipole moments of TATA$^+$ induced red-shifted absorption bands for TATA$^+$ in the solid-state absorption spectrum of TATA$^+$-Cl$^-$ (Fig. S96e).

Cartesian Coordination of 2-h-a

–3246.3261893 hartree

C,1.18602339,-4.0558665067,0.0346791158

C,-1.2315336763,1.6356995826,-0.025831118

C,-3.4846276035,1.3641621439,-0.0937542239

C,-2.8626474224,0.0701147139,-0.0669863658

C,-2.8466559098,-2.4316564386,-0.0024788728

C,-2.6007527036,-4.6681747105,0.1380966389

C,-1.331257243,-3.9704525668,0.074875096
C,1.272496332,1.5469076266,0.0492473705
C,2.5603832728,2.2412588364,0.0772349571
C,2.8149526719,-2.4976079689,0.0128314062
C,3.4304171441,-3.7820247788,-0.0745177903
C,2.8014881036,0.0030562094,0.0496036515
H,4.4919354747,-3.9561325474,-0.1600734389
H,2.705259617,3.3115041021,0.084026553
H,-4.5496832539,1.5346029224,-0.1325652623
N,-1.497964177,-2.6175236862,-0.0067825694
N,1.4632311596,-2.7091399806,0.0739851104
N,-1.50327332,0.2912282196,-0.0337831694
N,1.4442434779,0.1969015159,0.039083244
C,-2.5000893567,2.3104020116,-0.0619047031
H,-2.6271873534,3.3820673266,-0.0573473261
C,-3.553121805,-3.703099465,0.085931165
H,-4.6248660177,-3.8344784963,0.1197085963
C,2.4359717667,-4.7320267208,-0.0628028953
C,3.510064093,1.2812135599,0.0814605823
H,4.5816104466,1.4137175932,0.1101682364
C,-0.0947451246,-4.6363666769,0.0883947515
C,3.4531140634,-1.2373290642,0.0253978716
C,-3.4965511801,-1.174097444,-0.0526056312
C,0.0372931606,2.2298099028,0.017877987
H,-2.7267676518,-5.737852764,0.2134934964
H,2.5820863002,-5.8001294357,-0.1540596003
C,-4.9917609047,-1.1646765857,-0.0786260624
C,-5.7443183741,-0.7469794289,1.0243699817
C,-5.7034372727,-1.583217106,-1.2084082369
C,-7.1371916133,-0.7397202737,1.009951708
C,-7.0957034486,-1.5908995387,-1.2445421946
C,-7.8147636589,-1.1657048251,-0.1298422157
C,0.0658891661,3.7260188894,0.0280239944
C,0.3777142617,4.4437863101,1.1876300786
C,-0.2172854786,4.4726017285,-1.1206194098
C,0.4141091018,5.8360616251,1.2091552349
C,-0.1946719034,5.8654785255,-1.1227758733
C,0.12452901,6.5489739758,0.0480559397
C,4.9473556236,-1.2569173678,0.0031262984
C,5.6892608713,-1.7021882334,1.1033347315
C,5.6720100647,-0.8396210109,-1.119300069
C,7.08158977,-1.7323559255,1.0955339216
C,7.0646297749,-0.8533419205,-1.1483059563
C,7.7715928201,-1.3035620913,-0.0358886739
F,-5.0448257502,-1.9937521911,-2.2994957082
F,-7.7440835461,-1.9957839171,-2.341973351
F,-9.1495736996,-1.1653263362,-0.1544888881
F,-7.8248443984,-0.333666002,2.0830347341
F,-5.1269307029,-0.3350329502,2.1400189706
F,0.6591715226,3.7906303539,2.3226523994
F,0.7169819388,6.4903989355,2.3358363092
F,0.1523160515,7.8838301935,0.0577307924
F,-0.4704986757,6.5475370294,-2.2399793524
F,-0.5264729211,3.8481298207,-2.2649683629
F,5.0275179618,-0.4075692067,-2.2107366138
F,7.7246400816,-0.4467552748,-2.2381854247
F,9.1062818817,-1.3248804307,-0.0542588836
F,5.059549289,-2.1184579477,2.210627146
F,7.7573999617,-2.1628123163,2.1666937152
O,-0.186468943,-5.9979037175,0.1312086848
H,0.6656002535,-6.3782412567,0.3779260915
H,0.771374847,-1.9679088916,0.1179730864
H,-0.8042656375,-0.4429676566,-0.0264494051

Cartesian Coordination of 2-h-b

–3246.327678 hartree

C,-1.1966368159,-4.0447446405,-0.0406172101
C,1.2323900924,1.6327011607,-0.0286756098
C,3.486361133,1.3579748634,-0.0508907255
C,2.8623787788,0.0636948557,-0.0284543205
C,2.8426527517,-2.4393798946,0.0273928319
C,2.593663524,-4.6752222312,0.1280440981
C,1.3224537246,-3.9840039623,0.0239689646
C,-1.2725032993,1.5493912623,0.0167816896
C,-2.5592310253,2.2459339508,0.0403245637
C,-2.8222666381,-2.4927323476,-0.0131304958
C,-3.4380699626,-3.7803689482,-0.0873366818
C,-2.8041861228,0.0079008804,0.0256997748
H,-4.5022077425,-3.9542419774,-0.1343992514
H,-2.7025610244,3.3163961429,0.0415839277
H,4.552169704,1.527004665,-0.0708194367
N,1.4973756668,-2.6298474559,-0.0273538511
N,-1.4690384542,-2.6992267816,0.0167354527
N,1.5022545032,0.2876135331,-0.0234071267
N,-1.4459543258,0.200421179,0.0113556573
C,2.5033365439,2.3051951969,-0.0457058934
H,2.6317943146,3.3767174579,-0.0466269771
C,3.5466829049,-3.7064426356,0.1291478755
H,4.615187494,-3.8399378832,0.2144464737
C,-2.4428065295,-4.7281450665,-0.1056387494
C,-3.5103885713,1.2871849313,0.0519374745
H,-4.5817282484,1.4212760659,0.0809638833
C,0.0766571731,-4.6378494028,-0.026910523
C,-3.45751769,-1.2315188856,0.0090303437
C,3.4949279337,-1.1790825208,0.0016696333
C,-0.0347382433,2.2298767837,-0.0077075411
H,2.7696230041,-5.7399281311,0.226736669
H,-2.5475577564,-5.8006932158,-0.1607811327
C,4.989909636,-1.1712656653,0.0155013922
C,5.7319603742,-1.6040233675,-1.0894686934
C,5.7138152141,-0.7397004557,1.1326836583
C,7.1245990626,-1.612207281,-1.0888068823
C,7.1066438849,-0.7320908628,1.1546577161
C,7.814175578,-1.172359096,0.0387607329
C,-0.0599815219,3.7261882102,-0.0093149789
C,0.2366281455,4.4626679555,-1.1610740666
C,-0.3816620879,4.4542556381,1.1411649879
C,0.2172199684,5.8555180097,-1.1749594064
C,-0.4152164362,5.8467282732,1.1508185454
C,-0.1122919809,6.5493621277,-0.0131340557
C,-4.9523735589,-1.2481188413,0.001913587
C,-5.6865422982,-0.8489176811,-1.1204782858
C,-5.683338903,-1.6744229499,1.1164845969
C,-7.0793052426,-0.8623206803,-1.1365238287
C,-7.0757784176,-1.7033282629,1.1220642698
C,-7.7759308249,-1.2933583533,-0.010096208
F,5.068139088,-0.3144950578,2.2269557776
F,7.7657870701,-0.3128081456,2.240317264
F,9.1490845232,-1.1722896254,0.0494293191
F,7.8014439366,-2.0314234086,-2.1635459678

F,5.1028614155,-2.0288244236,-2.1928587838
F,0.5563495551,3.8280867779,-2.2969938166
F,0.5062200842,6.5277614831,-2.2948211436
F,-0.1369994841,7.8843337155,-0.0147601765
F,-0.7277460757,6.5110385696,2.2689974755
F,-0.6758427132,3.8111405264,2.2786627641
F,-5.042999199,-2.0721893381,2.2240119546
F,-7.7420265371,-2.1147229803,2.2064994949
F,-9.1108388511,-1.3136937482,-0.0155506093
F,-5.0512299268,-0.4348901509,-2.224410386
F,-7.7491791558,-0.4729700039,-2.2268348527
O,0.0245181567,-5.9997192735,-0.0532113806
H,-0.7812658648,-1.9542089198,0.0442708778
H,0.8002235038,-0.443272217,-0.0223099729
H,0.9095250859,-6.3533264452,-0.2063547063

Cartesian Coordination of 2-h-c

–3246.3117381 hartree

C,1.256020828,-4.1936429402,-0.0135446926
C,-1.2611365606,1.6527734853,0.0276889965
C,-3.4653270383,1.2493093444,0.1317880974
C,-2.68540839,0.0115167924,0.0471556406
C,-2.6663754466,-2.5361178407,-0.024628865
C,-2.5272488715,-4.7967887391,-0.1078401971
C,-1.231870798,-4.1552038312,-0.0591641278
C,1.2104573665,1.7274419753,-0.0345231165
C,2.5162695621,2.3151871934,-0.1049599745
C,2.7454759843,-2.4962834308,0.0002738697
C,3.4610954031,-3.7333514662,0.0591513548
C,2.7274934225,0.0442976722,-0.0367163625
H,4.5347861049,-3.8206950731,0.1147441982
H,2.7155359099,3.3746120038,-0.1609082923
H,-4.5406941872,1.3178721019,0.2024047963
N,-1.3340672627,-2.7961774305,-0.0141902229
N,1.4124277608,-2.8222820574,-0.0392256253
N,-1.3486723913,0.3003725803,-0.006169195
N,1.3852739586,0.3594089734,0.0008196687
C,-2.5779255216,2.2706576739,0.1191178718
H,-2.7856863727,3.3291193756,0.1803338016
C,-3.4298465001,-3.7824569292,-0.0862482191
H,-4.5060816928,-3.8665954535,-0.1174378676
C,2.5526937897,-4.766357249,0.0499365502
C,3.4302035412,1.3031419725,-0.105271495
H,4.5034056684,1.3995673899,-0.1604390709
C,0.0013426884,-4.8283114597,-0.0506834129
C,3.3421442579,-1.2114049317,-0.0187829324
C,-3.2827232215,-1.2586526493,0.0232760149
C,-0.0330904363,2.3606260785,-0.0077733396
H,-2.7066631823,-5.8609316948,-0.15324394
H,2.7932303042,-5.8201671639,0.1014482276
C,-4.7854712745,-1.2730080516,0.0517835572
C,-5.4890831882,-1.5578746447,1.2251816584
C,-5.540897402,-1.0089366063,-1.0938798768
C,-6.8813448615,-1.5796173596,1.2649911983
C,-6.9340314396,-1.0230636228,-1.0795906863
C,-7.6057148524,-1.310292361,0.1061670944
C,-0.0558964503,3.8510506515,-0.0134414454
C,0.4453494116,4.600131529,1.0581830487
C,-0.5866802503,4.5709720717,-1.0911296876
C,0.4254792391,5.9927155527,1.0629594342
C,-0.6249297572,5.9629227618,-1.1053961432
C,-0.1145485354,6.6763893647,-0.0234427036
C,4.8407815814,-1.2046767807,-0.0205339645
C,5.5724157218,-0.9132835278,1.1356230721
C,5.5714309081,-1.4952539842,-1.1775325159
C,6.9656054232,-0.9047129681,1.1460130556
C,6.9643069755,-1.4987026141,-1.1910359525
C,7.6628677144,-1.2003103815,-0.0231541078
F,-4.926823972,-0.7291825252,-2.2509659724
F,-7.6277824672,-0.7665584427,-2.1945871065
F,-8.9411291571,-1.3273365611,0.1324798047
F,-7.5249436343,-1.8536095073,2.4056507794
F,-4.822775484,-1.8223044857,2.3568857695
F,0.9661724722,3.9790185747,2.1257160538
F,0.9126419863,6.6745588434,2.1056038087
F,-0.141353088,8.0111172056,-0.0286746987
F,-1.1365609525,6.6170206924,-2.1537724324
F,-1.0768916241,3.9204181509,-2.1541121933
F,4.9305053193,-1.7860233123,-2.3170111211
F,7.6322977022,-1.7804563853,-2.3147665736
F,8.9974474742,-1.1981070149,-0.0246332877
F,4.9337144563,-0.6269187981,2.2769223064
F,7.6347051687,-0.6213931495,2.2684648657
O,-0.0707794721,-6.1858463238,-0.0691709339
H,0.8111551443,-6.567645168,-0.161149161
H,0.5615517042,-2.2500712405,-0.0697701107
H,0.5391827829,-0.2192578539,0.0366560285

Cartesian Coordination of 2-h-d

–3246.3136484 hartree

C,-1.2655087806,-4.1785397935,-0.0145769795
C,1.2605518878,1.6466353656,-0.0329203141
C,3.465508999,1.2429863474,-0.1312846523
C,2.6853819225,0.0049143293,-0.0461347608
C,2.6647200363,-2.543101811,0.0220211521
C,2.5224776874,-4.8014793384,0.0976074749
C,1.2241676865,-4.1642201226,0.0332025633
C,-1.2115610802,1.7291230825,0.0273209283
C,-2.5162759239,2.320632315,0.0971077914
C,-2.7547719137,-2.4891653496,-0.0040721416
C,-3.4676987869,-3.7303242101,-0.0542281174
C,-2.7335278574,0.0499331775,0.0358040622
H,-4.5424089597,-3.8181807099,-0.0897390126
H,-2.7127774068,3.3807360522,0.1496028715
H,4.5409760434,1.3115730082,-0.200584682
N,1.3350156905,-2.8049794437,-0.0053168478
N,-1.4197661938,-2.8087228345,0.0168923819
N,1.3471796468,0.2948468217,0.0034349385
N,-1.3900779155,0.3620768542,-0.0048074941
C,2.5783567739,2.2639642735,-0.1232076907
H,2.7863802413,3.3222199702,-0.1867557146
C,3.4264987358,-3.7856512872,0.0914102923
H,4.5016320339,-3.8734420133,0.1420158468
C,-2.5566456104,-4.7603652458,-0.0596435528
C,-3.4326206161,1.3109615411,0.1016044066
H,-4.5056184745,1.4100489283,0.1565510844
C,-0.0189071837,-4.8240828979,0.0041828409
C,-3.3502640613,-1.2043153636,0.0218421039
C,3.2827771534,-1.2631392759,-0.0194070277
C,0.0338980456,2.3583605855,-0.0000024282

H,2.7487491069,-5.8596855827,0.1573470525
H,-2.7531718559,-5.8211585572,-0.0938879369
C,4.7853339089,-1.2789410775,-0.0374457691
C,5.4973526144,-1.5801941494,-1.2019157418
C,5.5336401622,-1.0000179369,1.1096867201
C,6.8898121875,-1.6031920598,-1.2321126187
C,6.9268742306,-1.0146985705,1.1048602844
C,7.6065625876,-1.3182270683,-0.0722644122
C,0.0610346274,3.8486660483,0.0036591026
C,-0.4383118139,4.5978644515,-1.0688234369
C,0.5933817434,4.5687791569,1.0804880103
C,-0.4151464341,5.9903985839,-1.0752221584
C,0.635254753,5.9606219637,1.0930763353
C,0.1265667231,6.6740869298,0.0103269571
C,-4.8489828577,-1.1958552986,0.0353785208
C,-5.5892903825,-0.918255908,-1.1183698312
C,-5.5697832888,-1.4731823737,1.2015567114
C,-6.9824865862,-0.9105279867,-1.1180136556
C,-6.9625145001,-1.4765984717,1.2259765917
C,-7.6702587874,-1.192522509,0.0600700925
F,4.9125270281,-0.7054866191,2.2591676338
F,7.6131425326,-0.7440543418,2.2210357568
F,8.9420238863,-1.3365325536,-0.08915666
F,7.5407360662,-1.8932587333,-2.364648129
F,4.8388657731,-1.8601825243,-2.3346606792
F,-0.9606732476,3.9768112074,-2.1356600118
F,-0.9005977455,6.6721890503,-2.1187157453
F,0.1567417879,8.0087701286,0.0139287521
F,1.1486363611,6.6146838085,2.140666677
F,1.0821937604,3.9182797801,2.1442522034
F,-4.919460197,-1.749682871,2.3390013518
F,-7.6217371904,-1.7447584808,2.358103851
F,-9.0048333169,-1.1902759945,0.0721983197
F,-4.9591786366,-0.6443154665,-2.2676044084
F,-7.6605260582,-0.640123772,-2.2383711679
O,-0.0965421036,-6.1802508034,-0.0023033456
H,-0.5740132836,-2.2323470626,0.0413762554
H,-0.5458024471,-0.2187263063,-0.0355236959
H,0.7920870732,-6.5548379851,-0.0563590985

Cartesian Coordination of 2-h-e

–3246.3168991 hartree

C,7.9225933275,-1.2318351521,-0.1372801854
C,7.2332670213,-1.7167135381,0.9717094803
F,9.2573468983,-1.243493887,-0.1535938784
F,7.9104225781,-2.1886216968,2.0240195871
C,7.214980677,-0.7332323129,-1.2282038204
F,7.8736917999,-0.2699058708,-2.2960323922
C,0.0027090006,-4.451454206,-0.0532859359
C,1.2816345372,-3.8493349112,-0.0671210588
C,2.5035720829,-4.6394707485,-0.0872484788
C,3.5279963005,-3.7488079357,-0.1145708183
C,2.914670097,-2.4309877543,-0.09613227
C,3.6070941379,-1.1984822694,-0.086575229
C,2.9676724555,0.0447671619,-0.0495776309
C,3.5453493849,1.3493997182,0.0255788673
C,1.2741371817,1.5445307403,0.0338296783
C,0.0023146679,2.1473738659,0.0772421286
C,-1.2673918632,1.5436056023,0.068895929
C,-2.5168531407,2.2593281159,0.085772251
C,-3.5398761659,1.3539440736,0.0679648773
C,-2.9654418172,0.0442860384,0.0320270523
C,-3.6075805853,-1.1944684796,-0.0121329111
C,-2.9146755918,-2.4304776318,-0.0508129498
C,-3.5310565176,-3.7349192675,-0.1945163499
C,-2.5089053965,-4.63132986,-0.2155836157
C,-1.2816967516,-3.8582796714,-0.0801333907
C,5.0985646682,-1.2064541469,-0.1018429653
C,5.8408585973,-1.6956177357,0.9796618429
C,0.0027066423,3.6455620173,0.1385777363
C,-0.2335130883,5.719013017,1.4067422678
C,-0.2282361647,4.3276868239,1.3375208752
C,-5.0982224318,-1.2072007795,-0.0300229442
C,-7.2222289279,-0.7110382705,-1.1333013063
C,-7.9232645147,-1.2529116104,-0.0588923388
C,-7.2270695176,-1.7725703745,1.029858071
C,-5.8349208277,-1.7421471153,1.0341417014
F,5.2137778256,-2.1597215014,2.0680655269
F,-0.459350401,6.3412681737,2.5687255144
F,-0.459628144,3.6407208933,2.4636270731
F,-9.2578470261,-1.2733549626,-0.0719611156
F,-7.897532427,-2.2874065741,2.0661978031
F,-5.20130251,-2.2424469323,2.1027170123
H,2.552230671,-5.7178043577,-0.0854292001
H,4.5868443535,-3.9614996516,-0.1477100465
H,-2.6043105742,3.3344775701,0.0998467385
H,-4.5981160537,1.5665583389,0.0788530321
H,-4.5869745999,-3.9395992667,-0.297803508
N,1.5545852898,-2.5196995377,-0.0772883942
N,1.5959463133,0.2104991456,-0.0463661278
N,-1.5923708168,0.2074356868,0.037015977
N,-1.5599818844,-2.530771606,0.0114121877
O,0.071905927,-5.8136220951,-0.0395765703
C,2.5231951456,2.2568434215,0.0772538475
C,5.8225078636,-0.727949731,-1.2002583411
C,0.2337620392,4.4235594798,-1.0006043293
C,0.2393427114,5.8160279878,-0.9559371713
C,0.0031827095,6.4646312158,0.254062132
C,-5.8296905602,-0.6975720895,-1.109712764
F,5.1766984235,-0.2462555856,-2.2709544099
F,0.4638298049,3.8310997582,-2.1790725898
F,0.4637070979,6.5306147191,-2.0637154243
F,0.0029057368,7.7981666914,0.3093553354
F,-5.1920020077,-0.1764366259,-2.1670289651
F,-7.8874072449,-0.2166945457,-2.183049574
H,4.6042048439,1.55792817,0.0533871918
H,2.6130273311,3.3293022268,0.1516690089
H,-2.6136690186,-5.7000158928,-0.3622472751
H,-1.0155127477,-0.6386692535,0.0518175521
H,1.0198911137,-0.6366205881,-0.0794964663
H,-0.8029999381,-6.1808816305,0.135450464

Cartesian Coordination of 2-h-f

–3246.3111442 hartree

C,7.9303412208,-1.139998905,-0.1708492614
C,7.2547239273,-1.6417320238,0.9388708109
F,9.2650624178,-1.1264773417,-0.1925688898
F,7.9443105542,-2.1051560918,1.9872333027
C,7.2085967115,-0.6531704634,-1.2579932884
F,7.8544463399,-0.1764110045,-2.3276025293

C,-0.0024910038,-4.4967742205,-0.0216566327
C,1.2678641984,-3.9062429995,-0.0272043962
C,2.4952149085,-4.6404103054,-0.0047494264
C,3.5292332151,-3.7417898443,-0.0593455467
C,2.9653308651,-2.4293208729,-0.0949590103
C,3.6145987135,-1.1795848331,-0.1039361497
C,2.9181876686,0.0411047571,-0.0714327447
C,3.5402329113,1.3588126751,0.0045419861
C,1.2773554474,1.4659526888,0.0299375508
C,0.0055053271,2.0746894317,0.0945917233
C,-1.2707695946,1.4677328882,0.0959572014
C,-2.5115094482,2.2549832445,0.1238766428
C,-3.5346378524,1.3727730184,0.0960568061
C,-2.9207838396,0.0504895829,0.0470536736
C,-3.6200603375,-1.1653954981,-0.0110663024
C,-2.9729086631,-2.4179417811,-0.0497665528
C,-3.5379584436,-3.7167771936,-0.2034881282
C,-2.505018092,-4.6224977049,-0.2188414972
C,-1.2776409191,-3.905568467,-0.0663374629
C,5.1049996349,-1.1696216309,-0.1251153889
C,5.8622453903,-1.6468394372,0.9515156911
C,0.0081079053,3.5760756831,0.1664576356
C,-0.2151358125,5.6405130155,1.4514607413
C,-0.2117134609,4.2497700056,1.3713058328
C,-5.1094581023,-1.1595316311,-0.0466587904
C,-7.2125652368,-0.6059705139,-1.1630742449
C,-7.9351628847,-1.151320709,-0.1046424301
C,-7.2604115122,-1.7029720313,0.9816811256
C,-5.8681328883,-1.6989287733,0.9997914853
F,5.2484695241,-2.1292166873,2.0414407563
F,-0.4292494193,6.2544026221,2.6210289022
F,-0.4335225902,3.5547624871,2.4952815696
F,-9.269693878,-1.1466593093,-0.1318026213
F,-7.9508156098,-2.2239586256,2.0020660721
F,-5.25496757,-2.232130997,2.0660438061
H,2.5522050305,-5.7166184595,0.0405017261
H,4.5854835282,-3.9638391207,-0.0673890494
H,-2.5781218177,3.3323535203,0.1488534593
H,-4.593603989,1.5860339387,0.1110791313
H,-4.5900490168,-3.927401099,-0.3215905597
N,1.5991326764,-2.5697244128,-0.0878695717
N,1.5563643036,0.1384783302,-0.0574903547
N,-1.556563842,0.1406519225,0.057877482
N,-1.610310093,-2.5711434882,0.0305990168
O,0.0502991482,-5.8720614956,-0.0229295103
C,2.5226611462,2.2454530121,0.0696016734
C,5.8164377458,-0.6765209688,-1.2258645578
C,0.2283291489,4.362515847,-0.9680651191
C,0.2334752818,5.7547290418,-0.9138980852
C,0.0097757101,6.3950606124,0.3025350661
C,-5.8203409127,-0.6204103835,-1.1263085091
F,5.1579563929,-0.2076735724,-2.2935957143
F,0.447900713,3.7791677713,-2.1537480929
F,0.4469189604,6.4775544216,-2.0194374392
F,0.0102888284,7.7292203367,0.3677244324
F,-5.1624193104,-0.0985880308,-2.1695428166
F,-7.85778721,-0.0842283334,-2.211823819
H,4.6006711947,1.5644780504,0.0255976019
H,2.5956602871,3.3195353679,0.1525347855
H,-2.594430319,-5.6879215239,-0.3781086624
H,-0.7234001284,-6.2184871181,0.4393409156
H,-1.0432320293,-1.7177240799,0.1190947841
H,1.03571785,-1.70932429,-0.109003234

Cartesian Coordination of 2-o-a

–3246.3260612 hartree

C,-1.1903400298,-4.1072252671,-0.1336230688
C,1.2229089389,1.6661726959,-0.1264671121
C,3.4667935285,1.3755095891,-0.3422173287
C,2.8622078874,0.0816846406,-0.1281206905
C,2.9061435218,-2.4103744997,0.1737002533
C,2.5274120982,-4.5921801745,0.6669309576
C,1.3470392177,-4.0556560687,0.1608505676
C,-1.2616917321,1.5242543055,0.1335944082
C,-2.5523479949,2.1995901298,0.2425375432
C,-2.8100343856,-2.5407054609,-0.0890183285
C,-3.41185141,-3.7869429952,-0.3753408683
C,-2.7814792808,-0.0392937671,0.1648774079
H,-4.4649266071,-3.9400324754,-0.557366226
H,-2.70914103,3.2674010203,0.2748650891
H,4.5237509412,1.5334153033,-0.4945804959
N,1.5769486172,-2.7092036901,-0.1053101327
N,-1.4595748064,-2.7675782793,0.0378468617
N,1.4932274615,0.3166635651,-0.0462839975
N,-1.4083546398,0.1791489478,0.1153056655
C,2.4909719032,2.3228443117,-0.3319894353
H,2.6168132284,3.3874910785,-0.4563157928
C,3.4917279441,-3.5812711482,0.679746281
H,4.5031081606,-3.6539494172,1.0511896153
C,-2.408719763,-4.7483420544,-0.4005726538
C,-3.4915640536,1.2285636709,0.2706878077
H,-4.5619126274,1.3497925526,0.3472931193
C,0.0965265746,-4.7991374152,-0.011110627
C,-3.4282553091,-1.2648739067,0.0504419772
C,3.520353015,-1.1285202999,0.0019642255
C,-0.0339187651,2.2435511627,-0.0032846904
H,2.6242039197,-5.6115824299,1.0086551637
H,-2.4969599218,-5.8072130395,-0.5893450672
C,5.0108580414,-1.1174843513,0.0194039009
C,5.7502415158,-1.7595377912,-0.9831879727
C,5.7389889206,-0.4995318335,1.044079157
C,7.1418603996,-1.7835810129,-0.9759740666
C,7.1317258951,-0.5064397927,1.0691565979
C,7.8348082925,-1.1530428406,0.055628626
C,-0.0957377184,3.7364667259,-0.0450358256
C,0.1023418716,4.448407018,-1.2331674976
C,-0.3581674089,4.4900530953,1.1045808742
C,0.0472479714,5.8395920826,-1.2822554425
C,-0.4291564731,5.8806861772,1.0799784389
C,-0.2227459276,6.5577410122,-0.1199561189
C,-4.9237327855,-1.2826799687,0.0372131057
C,-5.6592071494,-0.7975989723,-1.0495320149
C,-5.6491847468,-1.815041345,1.1090260723
C,-7.0517577398,-0.8278021123,-1.069884018
C,-7.0409867678,-1.857604752,1.1102068697
C,-7.7440366653,-1.3612725821,0.0146098197
F,5.0990763211,0.120754093,2.0444878643
F,7.794498905,0.0928794412,2.0636228749
F,9.1685338672,-1.1663110522,0.0705546194
F,7.8154883042,-2.3962665546,-1.9543231443

F,5.1168197247,-2.3641278337,-1.9956642128
F,0.3645735083,3.790888077,-2.371700215
F,0.2450539845,6.48665905,-2.4360268961
F,-0.2815328278,7.8908427184,-0.1551307572
F,-0.6836256906,6.5679461078,2.1986662515
F,-0.5605162994,3.8707278588,2.2754006712
F,-5.00374105,-2.2952548157,2.1793112001
F,-7.7039085863,-2.3647893932,2.1545328216
F,-9.0780229037,-1.3951626034,0.0051206783
F,-5.0273676895,-0.2844309758,-2.1129856978
F,-7.7249010731,-0.3553059589,-2.1242318462
O,0.122919374,-6.0353839603,-0.0124505753
H,-0.8372630167,-2.0272905475,0.3327154047
H,0.7628252736,-0.3219091374,0.24046838
H,1.0469467476,-2.195552855,-0.797260355

Cartesian Coordination of 2-o-b

–3246.3246602 hartree

C,-1.2690248496,-4.0789650795,-0.2342268529
C,1.2586885584,1.6171602889,-0.1419737158
C,3.4882672223,1.2808495577,-0.4102636248
C,2.8760835938,0.0294600558,-0.1551531603
C,2.824460938,-2.4682825213,0.0876094444
C,2.5327252719,-4.6978406313,0.1966983722
C,1.2810464312,-3.9881965522,0.0734756032
C,-1.2374972645,1.6203676832,0.1237829605
C,-2.4883708426,2.2144432037,0.5015582671
C,-2.8604443109,-2.4813459768,-0.2337676475
C,-3.4658576864,-3.7130788285,-0.5688364602
C,-2.8684006668,0.0081925628,0.0818957426
H,-4.5125125566,-3.8412249362,-0.8012705506
H,-2.6050671255,3.2447218094,0.8024446254
H,4.541628625,1.4288835242,-0.5945987236
N,1.4436030163,-2.6554851114,0.0301010289
N,-1.515204313,-2.7400406496,-0.0600860912
N,1.5212530266,0.272693456,-0.0207377123
N,-1.4918320479,0.2586651511,-0.0821533708
C,2.5029348218,2.2485139827,-0.4086003779
H,2.629930713,3.3048167351,-0.5892332203
C,3.5020695215,-3.7449610322,0.211092412
H,4.5696288459,-3.8889524641,0.293068942
C,-2.4818083608,-4.6965695715,-0.5624022336
C,-3.4577454057,1.254867951,0.4740492806
H,-4.4941385707,1.374759634,0.7530232356
C,0.021628668,-4.7496525586,-0.0752951562
C,-3.4994508762,-1.2147080862,-0.0578337891
C,3.479526563,-1.240305527,-0.0399085454
C,-0.0063974593,2.2549090653,0.0169514126
H,2.6239550678,-5.7720466962,0.248053049
H,-2.5847132013,-5.7502906488,-0.7715785741
C,4.9756516721,-1.245309462,-0.0766364485
C,5.681027013,-1.7398286203,-1.1787830252
C,5.7285814782,-0.7468442494,0.9916533124
C,7.0733996297,-1.7460405036,-1.2184941643
C,7.1210955035,-0.7431866457,0.9739738831
C,7.7947921648,-1.2456032836,-0.1369565834
C,0.0018260512,3.7436226076,0.0967501129
C,-0.6089614128,4.5354308157,-0.8844228016
C,0.6253282278,4.4203530061,1.1538909627
C,-0.6058883857,5.9267985508,-0.822462335
C,0.6490494443,5.8102354282,1.2324663013
C,0.0285174804,6.5660300345,0.2399691576
C,-4.9896736347,-1.2243938037,-0.0105631405
C,-5.759773921,-0.5947009269,-0.9966514721
C,-5.6860672826,-1.8772330247,1.0156047137
C,-7.1521990826,-0.6088773948,-0.9690912726
C,-7.0771854059,-1.9077150411,1.0608142809
C,-7.8124807784,-1.2700417439,0.0636027628
F,5.1113768675,-0.2607346052,2.0763188945
F,7.8117068808,-0.2649187709,2.0140544188
F,9.1287838816,-1.2462275251,-0.1656145883
F,7.7189829737,-2.2215887104,-2.2881135054
F,5.020007732,-2.2202257101,-2.2384912202
F,-1.2212062147,3.9577815856,-1.9269718683
F,-1.1977508738,6.6498424554,-1.778836434
F,0.0405044133,7.8983701802,0.3082685527
F,1.2498760622,6.4218769221,2.2582911009
F,1.2201422014,3.7274365382,2.1331447696
F,-5.013544109,-2.4858848823,1.9992466688
F,-7.7098557718,-2.5319468971,2.0590892107
F,-9.1457227575,-1.2899104642,0.1001257563
F,-5.1614208157,0.042603785,-2.0119647875
F,-7.8555154888,-0.0000958148,-1.9293670379
O,0.0597745561,-5.9852906791,-0.1027280343
H,-0.7996632225,-2.115617722,0.2847810604
H,0.8947054279,-0.4701921173,0.2598297658
H,-0.9705198508,-0.2506221008,-0.7857985363

Cartesian Coordination of 2i-a

–3246.3112208 hartree

C,0.082836088,-3.7449082509,2.5046844497
C,-0.5432562561,1.6304838525,-0.510245232
C,-1.3954083722,1.1037702694,-2.5367256286
C,-1.1418420601,-0.0911244718,-1.8260053179
C,-1.2799437215,-2.5835252039,-1.528457295
C,-1.4689212074,-4.7411681592,-0.9076015874
C,-0.9762595106,-3.8908321152,0.1683154155
C,0.6317672764,1.8266323103,1.695329722
C,1.1917106977,2.5759178438,2.8128828498
C,1.3853976952,-2.1581073515,3.4000179094
C,1.2170285803,-3.155479763,4.362788977
C,1.6049597865,0.3688608874,2.9620110028
H,1.6486144087,-3.1441330972,5.3542312821
H,1.1113150272,3.6421459438,2.9667727795
H,-1.8109520971,1.161707539,-3.5307929842
N,-0.8526057293,-2.6232777567,-0.190608938
N,0.7009474686,-2.5301339556,2.2880029116
N,-0.6257291843,0.2653859855,-0.6067028853
N,0.9093200027,0.4563376021,1.8498055906
C,-1.0342387017,2.166123126,-1.7213114557
H,-1.0917238993,3.2167831099,-1.9608260427
C,-1.6390372561,-3.9257102257,-1.9756904747
H,-1.9839333057,-4.1914953563,-2.9642898608
C,0.4027159742,-4.1511023923,3.8006239704
C,1.8139372594,1.6642217296,3.6013148656
H,2.3565387956,1.8312524463,4.5226145477
C,-0.709882485,-4.4647937503,1.525096295
C,2.2055408093,-0.9037223138,3.5235449792
C,-1.4047499664,-1.4288821462,-2.2709372416
C,-0.0221206436,2.362397902,0.6086014325

H,-1.6454213011,-5.8023046781,-0.8173114378
H,0.0640333024,-5.0711835137,4.2527348172
C,-1.8997049631,-1.5469066218,-3.6791695456
C,-3.2332608446,-1.8538337515,-3.9664140218
C,-1.0416265297,-1.340674317,-4.7640524377
C,-3.6967243279,-1.9600049006,-5.2760156441
C,-1.4829349468,-1.4408572966,-6.0808527924
C,-2.816940147,-1.7522013908,-6.3358518575
C,-0.1885806856,3.8473918799,0.5402739852
C,0.899802916,4.701373816,0.33379576
C,-1.4500012888,4.4384294943,0.6704423197
C,0.7462549884,6.0844954894,0.267906378
C,-1.6267631699,5.8180491003,0.6085117322
C,-0.5221741716,6.6431909647,0.4064451033
C,3.6437827383,-1.0634493261,3.0062792137
C,4.72385401,-0.9036874054,3.8774228879
C,3.951419631,-1.3602323398,1.6761260188
C,6.0459932922,-1.0429202611,3.4637290935
C,5.2637531915,-1.505985303,1.2341665155
C,6.3164157823,-1.3468281774,2.1325958796
F,0.2454729874,-1.0408757931,-4.5485414315
F,-0.6375566243,-1.2436834147,-7.0968174756
F,-3.2500578721,-1.8509269624,-7.5933531029
F,-4.9773677089,-2.2539986108,-5.519237765
F,-4.1054694164,-2.0533448093,-2.9714710569
F,2.1316146948,4.198037982,0.1884809898
F,1.8053208755,6.8745443468,0.068223445
F,-0.6795309805,7.9659560506,0.3464891065
F,-2.8434337595,6.3530553131,0.7452288934
F,-2.5286603995,3.6710725954,0.8690919833
F,2.9716662099,-1.515222775,0.7727333508
F,5.5178043993,-1.7935178174,-0.0464679168
F,7.5780293063,-1.4828212664,1.7184692288
F,4.5030687014,-0.5982962295,5.1705716092
F,7.0500326216,-0.8835988655,4.3324619025
O,-1.1722919905,-5.5822852062,1.769001997
H,0.6065764722,-1.9859536628,1.4411728902
H,-0.3517867802,-0.3841311526,0.1238892923
H,2.3117524442,-0.7356303915,4.5977024156

Cartesian Coordination of 2i-b

–3246.2855023 hartree

C,7.0921279997,-1.7023215287,1.4025052558
C,6.080289479,-1.3268918925,2.2841378289
F,8.3484719105,-1.8087541188,1.8350505937
F,6.3690390606,-1.0777389913,3.5642006022
C,6.7891263975,-1.9664409951,0.0685748513
F,7.759149713,-2.3197059236,-0.7790490193
C,-0.3996940714,-4.4270487078,-1.874752695
C,0.8243493498,-3.8883354314,-1.2221274442
C,1.956075309,-4.6899438871,-0.8927732728
C,2.9044281506,-3.8598532592,-0.3555050714
C,2.3804355692,-2.5205913276,-0.3651598262
C,3.0239903072,-1.3486510553,0.0111611566
C,2.4794745078,-0.0315834717,-0.0450156049
C,3.2667119968,1.1693938201,0.1596347496
C,1.1182580801,1.6301978611,-0.3042268995
C,-0.0608327797,2.3837877491,-0.4975159102
C,-1.3486697829,1.8905298603,-0.7259032974
C,-2.559322287,2.6377503578,-0.8257651272
C,-3.5965888432,1.7457081827,-1.0011906459
C,-3.0353326694,0.4456870658,-1.0252315296
C,-3.7899220857,-0.8346295617,-1.1905081187
C,-3.0583548981,-2.0804870416,-1.6253711877
C,-3.7616512753,-3.1624709578,-2.2425737073
C,-2.8263560152,-4.1546656303,-2.4317829178
C,-1.6031424394,-3.6418868449,-1.9176317851
C,4.4347281366,-1.480806017,0.4883890244
C,4.7717248448,-1.2186310575,1.8212227661
C,0.0599626819,3.8754389056,-0.4307827752
C,0.3820924869,5.9239915669,0.8524970256
C,0.2642049383,4.5379816873,0.7834918088
C,-4.6178252346,-1.1193324973,0.0734151049
C,-6.7741635664,-1.6050930508,1.1114332233
C,-6.1598246419,-1.7414284762,2.3530485228
C,-4.7810798037,-1.5698413026,2.4646680896
C,-4.0308776819,-1.2679719641,1.3323228547
F,3.8209717819,-0.859461333,2.6913097906
F,0.5745226381,6.5301515431,2.0271668675
F,0.3501774252,3.837784507,1.9206970755
F,-6.8876139145,-2.032032228,3.4342014065
F,-4.1886070707,-1.6948546546,3.6568013336
F,-2.707339063,-1.1061720934,1.4742903196
H,1.9950300627,-5.7539271797,-1.0658115212
H,3.8804824836,-4.1335385834,0.015854349
H,-2.6280705556,3.7129859474,-0.7618582966
H,-4.6485069008,1.9675701059,-1.1092491418
H,-4.8149563721,-3.1737247064,-2.4937204906
N,1.0997711465,-2.612242288,-0.8967185073
N,1.1851217349,0.2633801007,-0.3289594506
N,-1.7075672221,0.5522152654,-0.8590593708
N,-1.7606148392,-2.3508906455,-1.4222148527
O,-0.3047500659,-5.5802892777,-2.3245981532
C,2.4144355798,2.2131531433,-0.0172793577
C,5.4729017802,-1.8533887489,-0.3727753305
C,-0.0274217822,4.660985609,-1.5843231664
C,0.0877419672,6.0484234829,-1.5388629593
C,0.292933761,6.6805068416,-0.3143552169
C,-5.9993727888,-1.2972828465,-0.0043725354
F,5.2177365931,-2.1056881929,-1.6624385436
F,-0.2229655939,4.0814562642,-2.7750446842
F,0.0039201349,6.7719095068,-2.6587021844
F,0.4033630837,8.0079073197,-0.2582120926
F,-6.6237106541,-1.1804593427,-1.1919880851
F,-8.0966739488,-1.7670850703,0.9953901445
H,4.323109325,1.2116884369,0.379958043
H,2.6449168251,3.2666343528,0.0379766543
H,-2.9505875502,-5.1355140847,-2.8667614159
H,-1.079109071,-0.2474336946,-0.8229773712
H,-4.5315734968,-0.6256051927,-1.9698083193
H,0.4358417231,-1.8481113288,-0.9870925599

Cartesian Coordination of 2i-c

–3246.2991655 hartree

C,6.563292703,-1.4631794736,1.7061757128
C,5.4072839377,-1.3521727741,2.4760535624
F,7.7542551487,-1.5571651548,2.2999775918
F,5.4925939703,-1.3411100619,3.8099227205
C,6.4735113542,-1.4765281568,0.3163534627
F,7.5828857845,-1.5764519617,-0.4226992307

C,-0.1557478049,-3.9948273212,-2.9802647356
C,0.964531276,-3.4961781067,-2.1172234732
C,2.1691500011,-4.2798783318,-1.8634433938
C,2.9336637041,-3.5165010437,-1.0471673115
C,2.2091181302,-2.2549150384,-0.8504414714
C,2.6960528406,-1.1484830928,-0.1897724373
C,2.0961322202,0.1733656677,-0.0878930534
C,2.8753665808,1.3582546048,0.2555304318
C,0.7430502229,1.8668283176,-0.2144740166
C,-0.4403048546,2.5804467678,-0.4253093779
C,-1.6911754199,1.9821440447,-0.7141554217
C,-2.9409676237,2.5619141569,-1.0217174925
C,-3.848850734,1.5207885643,-1.2094279607
C,-3.1587078972,0.3114612063,-1.0169237143
C,-3.7459612629,-1.080716842,-1.0660991366
C,-3.0664958622,-2.0678927712,-1.9862445585
C,-3.663860232,-2.870886482,-2.9600442975
C,-2.6641715723,-3.6974674585,-3.5046461753
C,-1.4717209638,-3.3822494972,-2.8560468357
C,4.0459007647,-1.2732394181,0.4513353428
C,4.170688011,-1.2541595154,1.8443990207
C,-0.4080772409,4.0695705775,-0.3448592397
C,-1.0789318389,6.1499780742,0.7422675483
C,-1.0975180255,4.7602926285,0.6589930121
C,-3.9762814506,-1.6495957654,0.343497591
C,-5.5466334393,-2.4158066456,2.0544892408
C,-4.4912155315,-2.7206895095,2.9094175993
C,-3.1807361101,-2.493620795,2.4946853657
C,-2.9446555463,-1.9660679756,1.2288529632
F,3.0759702168,-1.1447961907,2.6078483648
F,-1.7404061686,6.7788549406,1.7183354403
F,-1.7891161059,4.0846658525,1.585302427
F,-4.7325012305,-3.2262248328,4.1201707802
F,-2.1624361155,-2.7810918402,3.3099305798
F,-1.6600910037,-1.7619174397,0.8724209801
H,2.3638474632,-5.2643731564,-2.2624797732
H,3.8990500779,-3.7626627147,-0.6302516865
H,-3.1376784757,3.6203401972,-1.106327795
H,-4.8975526802,1.6033298142,-1.4572510367
H,-4.7128831964,-2.8552560184,-3.2223817454
N,0.9804341357,-2.3189899363,-1.5242980956
N,0.8249014806,0.4905327348,-0.349912118
N,-1.8724558421,0.6100651077,-0.7227880438
N,-1.7466691488,-2.3903676613,-1.9374100616
O,0.0625959015,-4.9369736107,-3.7419797989
C,2.0278971283,2.4179968226,0.1618791167
C,5.2244288607,-1.3789285927,-0.2936973273
C,0.3049548504,4.8367536233,-1.2736561555
C,0.3345632292,6.2277471138,-1.2099301741
C,-0.3602671361,6.8855516059,-0.1975468228
C,-5.2768564465,-1.8879070059,0.7943666747
F,5.1768197846,-1.3843724427,-1.6317249101
F,0.9738562657,4.2400596347,-2.2665780139
F,1.0185221453,6.9323174729,-2.1159232387
F,-0.3374912072,8.216838252,-0.1279402531
F,-6.3248829541,-1.6053671485,-0.0007011529
F,-6.8070087788,-2.6284767547,2.4446622951
H,3.9267612563,1.3808122753,0.5019061071
H,2.2521144033,3.4597502708,0.3407221288
H,-2.7686345589,-4.4494640864,-4.2724697373
H,-4.7451886397,-0.94746196,-1.4834591338
H,-1.0638850729,0.0072188866,-0.5447963755
H,-0.9903776776,-1.98105463,-1.392893277

Cartesian Coordination of 2i-d

–3246.275402 hartree

C,6.8684856359,-1.3114863606,1.3650553276
C,5.7841792912,-1.4672732986,2.2265277048
F,8.1116282979,-1.3665878591,1.8426735884
F,5.9913262652,-1.6715529518,3.5297243037
C,6.6530292551,-1.0977957965,0.0047532388
F,7.6920223133,-0.9463933359,-0.8205921618
C,-0.0850999048,-4.4498777587,-2.4782497068
C,1.0759064951,-3.805925129,-1.798048473
C,2.3503050391,-4.4104136591,-1.6117558627
C,3.1235328492,-3.5080519996,-0.9330225967
C,2.3459011384,-2.3099626278,-0.7178366977
C,2.8379314893,-1.1301634132,-0.1679857092
C,2.2339758466,0.1509901432,-0.0651024275
C,2.90064512,1.3402466419,0.3398236213
C,0.7850979154,1.8633257732,-0.2285603727
C,-0.4575413114,2.5538777755,-0.4529408395
C,-1.6295120936,1.9073825236,-0.764931354
C,-2.9514518737,2.4918687159,-0.9650508618
C,-3.7880196557,1.4464688035,-1.1661918286
C,-2.9587519996,0.2324233598,-1.1191817853
C,-3.5774419086,-1.1334959729,-1.2471796616
C,-2.8290243056,-2.2928964529,-1.8589925273
C,-3.526327712,-3.3187057161,-2.5777629636
C,-2.5786399687,-4.2571874529,-2.9283843737
C,-1.3496882708,-3.7732654906,-2.4059251647
C,4.2436994856,-1.1972427292,0.3575799443
C,4.4891355631,-1.4071595603,1.7170143487
C,-0.4454344087,4.0368868676,-0.2977192635
C,-1.13399815,6.0609121752,0.8799904655
C,-1.1411171287,4.6754090047,0.7352874304
C,-4.1600034411,-1.5074249303,0.1290639539
C,-6.110147215,-1.93296921,1.5407528848
C,-5.2816724372,-2.2405757473,2.6158268194
C,-3.8986593771,-2.1782629537,2.4601854933
C,-3.3553861509,-1.8125962388,1.2313030095
F,3.4640656825,-1.5573626792,2.5636810518
F,-1.8072218823,6.6401884541,1.8777743236
F,-1.8357434942,3.955046107,1.6243798834
F,-5.8097937594,-2.588870574,3.7922996381
F,-3.0991064077,-2.4633382326,3.4946184671
F,-2.0215656916,-1.7539851874,1.1327872143
H,2.5983112454,-5.4003485885,-1.9617584179
H,4.1503021637,-3.6315251288,-0.6236959425
H,-3.1903007751,3.5454181864,-0.9522372334
H,-4.8560229583,1.4645701781,-1.3404029395
H,-4.5884542566,-3.3325372369,-2.7898705704
N,1.090863931,-2.5650250979,-1.2701086653
N,0.9484979349,0.5163806632,-0.4035862574
N,-1.6996785449,0.5086088099,-0.8858487529
N,-1.5239674391,-2.5603718729,-1.7504542573
O,0.1248286464,-5.5381021637,-3.0341540452
C,2.0126382737,2.3937065667,0.2286385774
C,5.349111568,-1.0412923021,-0.4825147661
C,0.2703414694,4.8513779594,-1.1831088048

C,0.2931358451,6.2374239187,-1.0564940546
C,-0.4140388505,6.8434646719,-0.0198199434
C,-5.5413138483,-1.5690412347,0.323097519
F,5.1677435399,-0.8342134518,-1.7922028692
F,0.9546427213,4.2975595739,-2.1923185335
F,0.9804548673,6.9862911551,-1.9231644392
F,-0.4015478018,8.1699784743,0.1099731294
F,-6.3753137967,-1.264262213,-0.6907613355
F,-7.4397139866,-1.9820588156,1.6828838566
H,3.9314270839,1.3951586932,0.6534104891
H,2.2015651445,3.4315704994,0.4561739368
H,-2.7045725532,-5.1776552482,-3.4800267447
H,-4.4548524733,-0.9884856297,-1.8844678438
H,0.1205155043,-0.0402946639,-0.6517169081
H,0.1576552108,-2.0890427292,-1.2481352647

Cartesian Coordination of 2i-e

–3246.2714433 hartree
C,7.3424579366,-1.582363008,1.4081790937
C,6.3329469219,-1.1273337592,2.2540759946
F,8.5966835318,-1.6696448695,1.8520748269
F,6.6205293482,-0.7830167499,3.5127551751
C,7.0386706807,-1.944525643,0.0979166133
F,8.0069670863,-2.3724957297,-0.7169317672
C,-0.2273558967,-4.1204111015,-1.9415461416
C,0.9830645277,-3.6121592709,-1.2317328377
C,2.0653368879,-4.5560031296,-0.8956278599
C,3.0719473289,-3.8153927446,-0.3848748854
C,2.6045339574,-2.4285901578,-0.4173682884
C,3.2841381551,-1.2989384239,-0.020733559
C,2.6968690419,0.0125569305,-0.0741033198
C,3.3213200762,1.2673569102,0.1401952893
C,1.1295126246,1.5984498599,-0.3688499858
C,-0.1062875798,2.2535706305,-0.5417078227
C,-1.3967699419,1.7385672907,-0.7124028199
C,-2.5924359045,2.5272858962,-0.6897417286
C,-3.6732000741,1.6815828079,-0.7890895846
C,-3.157229266,0.369061344,-0.9029756158
C,-3.934879272,-0.8946296316,-1.0966010341
C,-3.1495076591,-2.1148846575,-1.5029165983
C,-3.7436188199,-3.2761719348,-2.0757151038
C,-2.695974815,-4.1408686478,-2.3165618809
C,-1.5167802579,-3.4789589021,-1.8707103925
C,4.6872979487,-1.3993716015,0.468451374
C,5.0273283112,-1.0426868936,1.7793509139
C,-0.0288383766,3.7541577504,-0.494991524
C,0.0380240065,5.8413092025,0.7606041665
C,-0.0338012812,4.4507986728,0.7159645132
C,-4.8135016915,-1.1772960965,0.1285759514
C,-7.0052279523,-1.6834495625,1.0782070649
C,-6.4429401053,-1.8006164867,2.3459663936
C,-5.0724655477,-1.6089431578,2.5151748776
C,-4.2789167538,-1.3075731153,1.4129264092
F,4.0778038854,-0.6086421991,2.6192078528
F,0.0302606941,6.4831149608,1.9317693883
F,-0.1086610632,3.7797742415,1.8715274519
F,-7.2130114475,-2.0913020233,3.3974499097
F,-4.5300467447,-1.7143353148,3.7328571397
F,-2.9651387899,-1.1253294533,1.6082981615
H,2.0068298343,-5.6214574404,-1.0581604064
H,4.027034694,-4.1519494857,-0.0082699859
H,-2.6171437328,3.6013299409,-0.5897866202
H,-4.7213461729,1.9433597606,-0.7935407066
H,-4.797638424,-3.4258478373,-2.2730803885
N,1.305096433,-2.3691558352,-0.939292655
N,1.3853385492,0.2401589892,-0.3719538004
N,-1.8144687593,0.4102744401,-0.8485636259
N,-1.816998466,-2.2237904881,-1.3671317013
O,-0.0509384663,-5.1875496903,-2.5542846181
C,2.3650598228,2.2399707937,-0.0535954178
C,5.7256552234,-1.8477721902,-0.3572708921
C,0.0503058309,4.5031703368,-1.6717205819
C,0.1234906344,5.8942391473,-1.6512174224
C,0.1164517779,6.563504266,-0.4288957603
C,-6.1877253781,-1.3748081045,-0.006883
F,5.4758744388,-2.1922804641,-1.6257204029
F,0.0580983102,3.8836898168,-2.8575696793
F,0.200116375,6.5854711554,-2.7911675108
F,0.1854532123,7.8942122192,-0.3963847283
F,-6.7624083178,-1.2754613463,-1.2203236231
F,-8.3192891821,-1.864628529,0.907509748
H,4.3597284276,1.4139369105,0.3947247779
H,2.5034240076,3.3064287781,0.0322430609
H,-2.7195717283,-5.1328721506,-2.7432496719
H,-4.6397027542,-0.6795288291,-1.9098702766
H,0.8183787954,-0.5704687724,-0.6641293171
H,-1.3296016957,-0.5126036237,-0.9787746052

Cartesian Coordination of 2i-f

–3246.2981805 hartree
C,1.1244510028,-3.5744847354,-1.7163309793
C,-1.5737801951,1.6915369563,-0.7976819136
C,-3.7714393444,1.5168107765,-1.2890033985
C,-3.1381576272,0.2205140022,-1.0536355811
C,-3.1413720585,-2.2337136399,-1.7320462555
C,-2.5467979564,-4.0560603216,-2.9113183033
C,-1.4054598329,-3.5387327793,-2.2956459555
C,0.8829431607,1.580999089,-0.1658114252
C,1.9640041186,2.2414943198,0.5564657479
C,2.6008655188,-2.2708636296,-0.6425916066
C,3.2052369064,-3.5339752325,-0.8421068194
C,2.4575538569,0.1102818451,0.0686925934
H,4.2025289903,-3.8043116617,-0.528929506
H,1.9586545912,3.2591037933,0.9191957087
H,-4.8119338006,1.6771418934,-1.5395460082
N,-1.8035742728,-2.4261254039,-1.5723468962
N,1.3290736792,-2.3373051348,-1.1835039847
N,-1.8621023372,0.3146616299,-0.7672232827
N,1.1891367089,0.3131654124,-0.4511693031
C,-2.789036067,2.4363050669,-1.139478563
H,-2.8620627855,3.506705721,-1.2628882676
C,-3.6326828967,-3.2406183005,-2.5601458198
H,-4.6631357865,-3.3519378969,-2.8678904302
C,2.2906699112,-4.3347944301,-1.5105383492
C,2.9446478685,1.3130429185,0.7124619689
H,3.8897790348,1.4294535731,1.2227072634
C,-0.0767072834,-4.125214385,-2.4024733435
C,3.130535024,-1.1068198149,-0.0324891618
C,-3.8977237979,-1.0891644168,-1.1118640443
C,-0.3570687469,2.2603148436,-0.5052678677

H,-2.5448192368,-4.9319781395,-3.5420973366
H,2.3989676677,-5.3591249609,-1.8308574756
C,-0.2774227973,3.7557491794,-0.4885542165
C,-0.9554902282,4.5310003126,0.4584651055
C,0.5201427902,4.436835801,-1.4155847053
C,-0.8586160619,5.9208206363,0.4775822401
C,0.629710436,5.8243833173,-1.4174945878
C,-0.0635370421,6.568472894,-0.4648404747
C,4.508465935,-1.211572037,0.5327247315
C,4.7820390428,-2.0132189327,1.6466789966
C,5.581793088,-0.5137664299,-0.0324192646
C,6.0638967169,-2.1174209004,2.1806615425
C,6.8716880089,-0.6048826866,0.4847156012
C,7.1119609055,-1.4100889179,1.5956984233
F,-1.7183136403,3.9442524729,1.3899868347
F,-1.5184881985,6.6331066872,1.396462138
F,0.0334350727,7.8986790354,-0.456450819
F,1.3891880536,6.4447183869,-2.3252592997
F,1.1967407804,3.7484734677,-2.3427424491
F,5.3879209197,0.2621402318,-1.1053834387
F,7.8770873718,0.0698728302,-0.0803987623
F,8.3431517439,-1.5030908251,2.0991978185
F,3.7958510428,-2.6990453315,2.2373425544
F,6.2920312058,-2.8847811934,3.2503796558
O,0.0893966131,-5.1623255857,-3.0508266225
H,0.771398258,-1.4789782778,-1.220817587
H,-1.2540021633,-1.7682203533,-1.0302014138
C,-4.5003890631,-1.4084107038,0.2633113417
C,-3.7398299304,-1.8298614176,1.3572737062
C,-5.874117326,-1.2663402551,0.4725836957
C,-4.3164261637,-2.1126388684,2.5929203557
C,-6.4772227357,-1.5406486625,1.6970628941
C,-5.6913094572,-1.967035215,2.7637910973
H,-4.7577478202,-0.9172812573,-1.7634195388
F,-2.4126072903,-1.9796828275,1.2426768372
F,-3.5575285982,-2.518674989,3.615738916
F,-6.2520005908,-2.234142249,3.9452434214
F,-7.7973676457,-1.3945229301,1.8524483773
F,-6.6649972465,-0.8470483638,-0.5341565088

Cartesian Coordination of 2i-g

–3246.3143714 hartree
C,1.3594476956,1.442460077,-0.4254586436
C,-1.3827187977,-3.9155352093,0.6321851871
C,-3.4385037433,-3.782723868,-0.2850260368
C,-2.8816826316,-2.4890398209,-0.23333498
C,-2.8027328592,-0.0216982111,-0.6524629204
C,-2.442415956,2.1921985843,-0.8485206935
C,-1.2324715596,1.4595403118,-0.5414679164
C,1.1879884578,-3.9040727399,0.8537784288
C,2.3854666085,-4.7256336658,0.8764795508
C,2.9070202482,-0.1741335357,-0.3236695022
C,3.5733469977,1.0513074426,-0.5515500778
C,2.8017418477,-2.6192468931,0.2069648878
H,4.6408320391,1.1695104709,-0.6605426455
H,2.4340919064,-5.7658454487,1.1712262742
H,-4.4101036652,-4.0282018687,-0.6867117558
N,-1.4361034757,0.1517295399,-0.4092810262
N,1.5610450138,0.1045810705,-0.260133371
N,-1.6266026722,-2.6187052463,0.3331042975
N,1.4196911794,-2.6697460303,0.4592365287
C,-2.5027533877,-4.667658229,0.2515814059
H,-2.6032857346,-5.7374491223,0.3678407964
C,-3.4274615124,1.2583071881,-0.9305144716
H,-4.4756585475,1.4189034065,-1.1381284931
C,2.6112186359,2.0535097148,-0.6009131871
C,3.3987962581,-3.9180056324,0.4616781167
H,4.4438929629,-4.1692826467,0.3518384198
C,0.0728765136,2.1591711045,-0.4634921885
C,3.4796747763,-1.4705181405,-0.1490743709
C,-3.4506537594,-1.2505366083,-0.6321936279
C,-0.1518661839,-4.4338551922,1.3231295764
H,-2.4983564466,3.2611751057,-0.9878324012
H,2.7508129718,3.1121565883,-0.7557173934
C,-4.882834659,-1.2980781029,-1.0573745032
C,-5.2580113262,-1.0489444923,-2.3825623087
C,-5.9044326793,-1.6019119977,-0.1502821346
C,-6.5886803834,-1.098652659,-2.7913310838
C,-7.2409476169,-1.6542716728,-0.5372240618
C,-7.5826534756,-1.4020755725,-1.8640228146
C,4.9601986298,-1.5653262065,-0.3534392258
C,5.8622077417,-1.0809395341,0.5981404208
C,5.4945356293,-2.143298612,-1.5085347073
C,7.2402248594,-1.1653366414,0.4142239705
C,6.8691539585,-2.2398972861,-1.7129458553
C,7.7436611473,-1.7483187858,-0.7466897004
F,-5.6128816344,-1.8386759662,1.1347795092
F,-8.1945256956,-1.9397181485,0.3549604453
F,-8.8597052339,-1.4512872156,-2.245515367
F,-6.9153507555,-0.8634434486,-4.0656866613
F,-4.3296439339,-0.7650068641,-3.302824222
F,4.6800132476,-2.623965555,-2.4558924484
F,7.3513391304,-2.7986960648,-2.8273016186
F,9.0618815471,-1.8368907259,-0.931634332
F,5.4073692301,-0.5197270074,1.7255299646
F,8.0789175313,-0.6973638903,1.3437420731
O,0.090116832,3.3905657879,-0.4992671723
H,0.84548298,-0.6001394077,-0.1156211024
H,-0.9899099062,-1.8383759798,0.4499594476
H,-0.1413857368,-5.5066645278,1.1171920876
C,-0.2551238983,-4.3273925092,2.8520370977
C,-0.2581856965,-5.4829118489,3.6365602648
C,-0.3356526833,-3.1079977805,3.5295910212
C,-0.3445748451,-5.4427785334,5.0256099127
C,-0.4246769159,-3.0376260264,4.9173009852
C,-0.4290660635,-4.210913227,5.6686497567
F,-0.1699955572,-6.691615671,3.0490670059
F,-0.3449227135,-6.5734854414,5.7390427324
F,-0.5133547165,-4.1531400419,6.9989619708
F,-0.5042348236,-1.8536391384,5.5311096921
F,-0.3320791546,-1.9536429009,2.8458438542

Cartesian Coordination of 2i-h

–3246.2879882 hartree
C,7.498423374,-1.5250842309,-0.5671279741
C,6.9482307133,-0.8117721725,0.495012277
F,8.8247995527,-1.6289686118,-0.6879993231
F,7.7496218612,-0.2314242453,1.3959421708
C,6.6627880729,-2.1280189222,-1.5041946234
F,7.1901115223,-2.8102314569,-2.5273921628

C,-0.1017253146,-4.379168659,1.5512888586
C,1.1612575624,-3.7475147712,1.2074608274
C,2.440314401,-4.3312837014,1.4679274807
C,3.3503127671,-3.4410220305,0.9688147182
C,2.5963763457,-2.3297083179,0.4257864757
C,3.2052483697,-1.1978619083,-0.1730511954
C,2.6575948084,0.0008413295,-0.6366447851
C,3.4429739119,1.0548468009,-1.2400592304
C,1.2964362977,1.6233893926,-1.0867549218
C,0.0581025287,2.4814262268,-1.1951484885
C,-1.2595326942,1.776858796,-1.0786705381
C,-2.5265036905,2.2847658198,-1.4339218667
C,-3.4443324769,1.2889766298,-1.1715135406
C,-2.7475979999,0.1628724396,-0.6406585749
C,-3.3520193431,-1.0254734057,-0.2031425662
C,-2.7954979288,-2.1818403023,0.3619986868
C,-3.5485045039,-3.2832628024,0.8841487982
C,-2.6678600489,-4.210574307,1.3922845718
C,-1.3700217326,-3.6973914945,1.1798105729
C,4.7011163432,-1.3033044942,-0.3140276465
C,5.563550425,-0.7084131708,0.6089678805
C,0.1368142569,3.6664917977,-0.2261702643
C,0.0597418884,4.5935669624,2.0282592841
C,-0.047103772,3.5183891992,1.1509721457
C,-4.8483991046,-1.0574127278,-0.3444685605
C,-6.841862176,-1.7796994476,-1.5446264211
C,-7.6480692255,-1.1386882647,-0.6057512719
C,-7.067503247,-0.4563782971,0.4620112953
C,-5.6800841789,-0.4221410963,0.5812143424
F,5.0659716237,-0.0159296236,1.6419284503
F,-0.1329058071,4.4201100599,3.3387235244
F,-0.3468022665,2.3143712672,1.6571153373
F,-8.9741904987,-1.176987949,-0.7293910459
F,-7.8400236209,0.1571200545,1.3617032176
F,-5.1459935663,0.2387583974,1.6148441887
H,2.609698548,-5.2798883785,1.9556367509
H,4.4265237278,-3.5309036361,0.9853208521
H,-2.7154718637,3.2681625401,-1.8392852842
H,-4.5097261206,1.3317311217,-1.3360152701
H,-4.6260351244,-3.3423082828,0.8853426738
N,1.2507423898,-2.5478427014,0.5875021199
N,1.318663605,0.4019625633,-0.5670045018
N,-1.3989872563,0.5227343684,-0.6112375471
N,-1.4565932601,-2.4925167828,0.5742704192
O,-0.1877158059,-5.46636617,2.1286816195
C,2.5846533667,2.0809039435,-1.521377458
C,5.2812103238,-2.0133133339,-1.3671191072
C,0.4511920172,4.9446712478,-0.6891176315
C,0.569909689,6.037235647,0.1662227997
C,0.3717724446,5.8587046001,1.5328072987
C,-5.45662896,-1.7340466942,-1.4048921558
F,4.5049135724,-2.6039829734,-2.2848939551
F,0.6628193861,5.1458165485,-2.003685049
F,0.8723330247,7.24663703,-0.3160082206
F,0.4789284601,6.8952933179,2.3658116207
F,-4.703702206,-2.3571002162,-2.3184188597
F,-7.3981052652,-2.4306307894,-2.5688230129
H,4.5044203579,1.021388349,-1.4354618465
H,2.8042996512,3.036691497,-1.9785620054
H,-2.873385482,-5.1566727242,1.8681555604
H,-0.5584494565,-2.0325719318,0.3302899882
H,0.0691113857,2.9281596729,-2.1963110553
H,-0.5309965789,0.017754405,-0.3504168454

Cartesian Coordination of 2i-i

–3246.304707 hartree

C,1.3150219264,-2.2345452122,-2.2578298692
C,-1.2885582169,1.4793110636,1.530871275
C,-3.3796304064,0.7746480493,2.0199484015
C,-2.9120475123,0.0635456739,0.9005007515
C,-2.9164284601,-1.604861763,-0.9423854391
C,-2.5115774294,-2.9184266743,-2.7225033161
C,-1.3150364444,-2.2345427472,-2.2578284366
C,1.288553562,1.4793047527,1.5308734227
C,2.3640468978,1.6547284951,2.4108253852
C,2.9164158605,-1.6048693259,-0.94238687
C,3.5125377749,-2.5340681449,-1.8852141268
C,2.9120390356,0.0635355487,0.9005014546
H,4.5506121954,-2.8339513289,-1.9130557764
H,2.3886686147,2.3569725252,3.2321574741
H,-4.3443589824,0.6471191488,2.4882089744
N,-1.5536152974,-1.4520608004,-1.2184093787
N,1.5536028082,-1.4520653494,-1.2184096217
N,-1.6354057146,0.5218826384,0.6358508463
N,1.6353998772,0.5218789103,0.6358496716
C,-2.3640535017,1.6547430884,2.4108192578
H,-2.3886745245,2.356990241,3.2321487019
C,-3.5125524603,-2.5340618402,-1.8852102211
H,-4.5506272854,-2.8339438037,-1.9130502189
C,2.5115614467,-2.9184300062,-2.7225069867
C,3.3796217251,0.7746316323,2.0199531329
H,4.344348714,0.6470973366,2.4882155026
C,-0.0000078196,-2.4869548997,-2.9090789386
C,3.544357035,-0.9284317531,0.0936426638
C,-3.5443678629,-0.9284211499,0.093643146
C,-0.0000004264,2.2694444793,1.5084107647
H,-2.5482825487,-3.5974502986,-3.5612199774
H,2.5482648381,-3.5974522701,-3.5612248352
C,-4.9736397612,-1.2314205024,0.3932154696
C,-5.3689134055,-2.4866999051,0.870264365
C,-5.9774481306,-0.2726531868,0.2118118338
C,-6.7007911926,-2.7793961798,1.1543315109
C,-7.3146570374,-0.5453095272,0.4872234214
C,-7.6764138298,-1.8043907145,0.9610689721
C,4.9736278588,-1.231435554,0.3932149284
C,5.9774387825,-0.2726700953,0.2118151936
C,5.3688982098,-2.4867175695,0.8702598415
C,7.3146468497,-0.5453306802,0.4872266723
C,6.7007751013,-2.7794180772,1.154326769
C,7.6764002656,-1.8044143754,0.961068133
F,-5.6681833603,0.9433728945,-0.2558334561
F,-8.2506659018,0.3891910562,0.2937585642
F,-8.954807658,-2.0749413081,1.2286394219
F,-7.0449348692,-3.9855866801,1.6157112883
F,-4.4589859726,-3.4444323625,1.0802809313
F,4.458968441,-3.4444485288,1.0802728507
F,7.0449155423,-3.985610958,1.6157027129
F,8.9547932708,-2.0749690367,1.2286383936
F,5.6681775218,0.9433582651,-0.2558264422
F,8.250658166,0.3891682048,0.2937654856

O,-0.0000090218,-3.0393927228,-4.0090909183
H,-1.1070359678,0.0872513635,-0.1261956157
H,-0.0000001294,2.8285947495,2.4452579713
C,0.0000040287,3.3414442107,0.4069153556
C,-0.0000014884,4.6947457322,0.7531113339
C,0.0000142406,3.047750223,-0.9577478642
C,0.0000026192,5.7096874239,-0.2002160714
C,0.0000186407,4.0386358361,-1.9348813206
C,0.0000128099,5.378415987,-1.552423317
F,-0.0000110659,5.0549712309,2.0497956743
F,-0.0000027048,6.9923073308,0.1749363461
F,0.0000172595,6.3385371534,-2.4781533727
F,0.0000287541,3.7139569777,-3.2299735319
F,0.00002093,1.7654904673,-1.3726613338
H,1.1070287867,0.0872496419,-0.1261970106

Cartesian Coordination of 2i-j

–3246.3058581 hartree
C,1.3232286338,-2.6854957082,-2.0233602776
C,-1.2972547094,1.4300996446,1.3473396684
C,-3.4516128454,0.9737785118,1.8365322083
C,-2.9077968514,0.0727644011,0.8367577034
C,-2.9316146198,-1.79111476,-0.8060822174
C,-2.4891457745,-3.2758461694,-2.4488489399
C,-1.2883047566,-2.6575246113,-2.0632162536
C,1.3156720312,1.4660928023,1.3177027773
C,2.486592027,1.9397839925,2.0456268042
C,2.9579053514,-1.801528218,-0.7665671907
C,3.5177300034,-2.8557526527,-1.5156079723
C,2.9339148245,0.1145828518,0.8165004943
H,4.5463445656,-3.1806433315,-1.4691403542
H,2.5175321729,2.8034001229,2.697372575
H,-4.4528688123,0.9473015433,2.2422031514
N,-1.5796670484,-1.7674724586,-1.0662355159
N,1.6205347877,-1.7304524019,-1.0913016178
N,-1.5659068446,0.3982049314,0.581210959
N,1.5756222807,0.3975883784,0.6002889597
C,-2.4435543114,1.8306161562,2.1544698225
H,-2.462953675,2.6519601597,2.8591403629
C,-3.5101477309,-2.747189954,-1.6644187799
H,-4.5583802572,-3.0039374988,-1.7000818881
C,2.5034214558,-3.3938757688,-2.3022989155
C,3.5002365128,1.0875978301,1.7327935598
H,4.5242484828,1.1196237383,2.0766698156
C,0.0217655373,-3.0397096044,-2.6276327495
C,3.5833779335,-0.9168864642,0.1698318535
C,-3.5574154777,-0.9425814677,0.1647235526
C,-0.0010305315,2.2121576712,1.3620163125
H,-2.5539836301,-4.0382077091,-3.2095154866
H,2.5587539238,-4.2186904362,-2.9955949419
C,-5.0062875758,-1.1819600511,0.4284919298
C,-5.448593007,-2.3735015912,1.0155758503
C,-5.9815363392,-0.2362236237,0.0919034093
C,-6.7967982359,-2.6169313639,1.2631976242
C,-7.3356829467,-0.4598161924,0.3301809806
C,-7.7434192956,-1.6548172032,0.917821059
C,5.0338553355,-1.1427744167,0.444869603
C,6.0099623137,-0.8826994903,-0.5229071986
C,5.4725782333,-1.6266006289,1.6818381106
C,7.3644404318,-1.0921195429,-0.2766177202
C,6.822333036,-1.8429307845,1.9498425644
C,7.7705713874,-1.574139159,0.9658004429
F,-5.6302789216,0.9191306609,-0.4857134141
F,-8.2434624763,0.4615208115,-0.0067516925
F,-9.037642616,-1.8771801864,1.1509681457
F,-7.1856533596,-3.7609999311,1.8340405132
F,-4.5639151261,-3.3138698842,1.3685849414
F,4.587968495,-1.9013881632,2.6482584484
F,7.2101030651,-2.3083100595,3.1413642902
F,9.0658325645,-1.7759881968,1.2126370008
F,5.6527388338,-0.4099165193,-1.7234175389
F,8.2741262431,-0.8270834373,-1.2191359722
O,0.0298043383,-3.7876343499,-3.6075288992
H,0.003298646,2.7498680128,2.3135118852
C,-0.0293726389,3.314492359,0.2936799333
C,-0.0695237649,4.6582842353,0.674400361
C,-0.0191750438,3.0499511462,-1.0799306583
C,-0.0978697167,5.6984943203,-0.2502626663
C,-0.0472236231,4.0726885958,-2.0255639323
C,-0.0867793547,5.4014825804,-1.6100695181
F,-0.0814538502,4.9846061109,1.9815755275
F,-0.1354144626,6.971328351,0.1592085757
F,-0.1133209348,6.3849526971,-2.5121449743
F,-0.0358310768,3.7849719377,-3.3310705704
F,0.0198052424,1.7911521038,-1.531407271
H,-1.0147323768,-1.0503701311,-0.6022215553
H,1.0672689294,-0.9692875369,-0.6863010943

Cartesian Coordination of 2⁻-a

–3246.0118661 hartree
C,1.2156262321,-4.1178732813,0.056697105
C,-1.2374685522,1.6435371101,-0.0325426734
C,-3.4890734114,1.3259529238,-0.0709802952
C,-2.8505883277,0.0459871853,-0.034536616
C,-2.8453380215,-2.4795691807,-0.004081825
C,-2.6121355769,-4.7210866926,0.0224800066
C,-1.3424495747,-4.0440810858,0.0292103053
C,1.2711597208,1.5609106768,-0.007205745
C,2.5667769519,2.2432670175,0.0220337226
C,2.8114899605,-2.526826405,0.0602822762
C,3.4506663972,-3.794030433,0.0828850251
C,2.7950562772,-0.0022398381,0.0364396335
H,4.519625769,-3.9520720528,0.0993586163
H,2.7260223259,3.3131570602,0.0269096037
H,-4.5578806408,1.4832860354,-0.095942335
N,-1.4845358812,-2.6979790517,0.0136188002
N,1.4586099872,-2.7660080419,0.0444882045
N,-1.4877541916,0.2948193329,-0.011311443
N,1.4326758708,0.2134621597,0.0048356674
C,-2.51580754,2.2955202118,-0.0728453138
H,-2.665126006,3.3649623991,-0.1017481987
C,-3.5631697752,-3.7367648651,0.0014473202
H,-4.6379835046,-3.8616567561,-0.009044996
C,2.460980926,-4.770788188,0.0804904503
C,3.5112999534,1.2714412861,0.0465218069
H,4.5850288732,1.4003522579,0.0719591008
C,-0.0828537063,-4.7945179377,0.0494222714
C,3.4276722421,-1.2423889131,0.0581023816
C,-3.4635144103,-1.2095931441,-0.0277582251
C,0.0331506857,2.2464420025,-0.028243034

H,-2.7398140935,-5.7938244738,0.0323286976
H,2.5754788415,-5.844230159,0.0944361722
C,-4.964342876,-1.195254774,-0.0500974594
C,-5.718125631,-1.1770360702,1.1281052782
C,-5.6835463018,-1.2137260831,-1.2497375959
C,-7.1120591102,-1.1770814954,1.1220126788
C,-7.0770460891,-1.2140156673,-1.2840345551
C,-7.7940487619,-1.1967431317,-0.0911700716
C,0.0582886513,3.7404597317,-0.0488836842
C,-0.2116985524,4.505352701,1.0942079764
C,0.3523953318,4.4625636637,-1.213656948
C,-0.1919813659,5.8989497441,1.0882531317
C,0.3849320883,5.8555659301,-1.2465186569
C,0.1098996301,6.5775607507,-0.0888576927
C,4.9265340591,-1.2582837344,0.083033226
C,5.6451939868,-1.2660481954,1.2838858628
C,5.6839088295,-1.2845506074,-1.093504402
C,7.0384081211,-1.2895585601,1.3210431023
C,7.0776188263,-1.3084709462,-1.0850454542
C,7.7574377375,-1.3115139998,0.1294916391
F,-5.0350779518,-1.2287431566,-2.4259999127
F,-7.7348509379,-1.22948647,-2.4575250961
F,-9.1376056019,-1.1954222671,-0.1108966336
F,-7.8035783765,-1.1571810904,2.2758449727
F,-5.1040484008,-1.1560973557,2.3224827446
F,-0.4949382767,3.8990013435,2.2599861532
F,-0.4524268408,6.5949978817,2.2105542935
F,0.1349773907,7.9213398354,-0.1076134149
F,0.6701557192,6.5098958722,-2.3876576759
F,0.6109728303,3.8136666706,-2.3618410839
F,5.072752243,-1.2825674629,-2.2896913467
F,7.7714304675,-1.3279070843,-2.2377390546
F,9.1008666832,-1.3332782303,0.1517417069
F,4.9954751761,-1.2450725016,2.4595604702
F,7.6944699854,-1.2904154248,2.4958425213
O,-0.101556366,-6.0433468646,0.0616016027
H,0.7408736654,-2.049281523,0.0255327386
H,-0.7657867829,-0.4175675877,0.0081371724

Cartesian Coordination of 2^{-}-b

–3245.9995931 hartree

C,-1.2415176701,-4.1994027611,-0.0004843824
C,1.2096256804,1.728003471,-0.0083840004
C,3.439128589,1.3075682064,-0.0520149392
C,2.7404583893,0.0388764897,-0.0350067882
C,2.7685775236,-2.5196646049,-0.0423150855
C,2.5736227771,-4.7913934233,-0.0467723136
C,1.2915751581,-4.2267588219,-0.0298051599
C,-1.2734161461,1.6452536355,0.0162755672
C,-2.5869250718,2.2549350656,0.0661537189
C,-2.6817273429,-2.5734078443,0.0271157543
C,-3.4427340429,-3.8124355369,0.0297029412
C,-2.6972928451,-0.0065107777,0.0421281953
H,-4.520540844,-3.9027928148,0.0425507058
H,-2.8082368923,3.3137491138,0.0913932077
H,4.5138412461,1.4132981685,-0.0740712258
N,1.4335258027,-2.8562629436,-0.0270190713
N,-1.3342243193,-2.8534902858,0.0080018824
N,1.3912871574,0.3565805148,-0.0067467954
N,-1.3558598622,0.2943614073,0.0054959133
C,2.5224054209,2.3177208396,-0.0391316391
H,2.7240818607,3.3793422841,-0.0512722752
C,3.4897936897,-3.7433429813,-0.0544832525
H,4.5665824097,-3.8288498133,-0.0674689683
C,-2.5320413782,-4.8336932397,0.011998077
C,-3.4776037934,1.2202366552,0.0792683045
H,-4.5557781706,1.2952357377,0.1142867624
C,0.018601633,-4.9495798017,-0.0183269134
C,-3.2745776009,-1.2911993019,0.0456365157
C,3.347599784,-1.2099863469,-0.0479097118
C,-0.0303113183,2.3534604846,0.0013431126
H,2.768844894,-5.853385779,-0.0524394299
H,-2.7029197231,-5.9007068447,0.0080667597
C,4.8477403666,-1.2000140927,-0.0720877701
C,5.567143844,-1.2068138553,-1.2727269514
C,5.6050698788,-1.2044838139,1.1049790331
C,6.9605578162,-1.2119992561,-1.3093575013
C,6.9989940749,-1.2097647524,1.0973008328
C,7.6792003532,-1.2147106007,-0.1171854069
C,-0.0598086916,3.8444761888,0.0007751791
C,-0.4370952382,4.5769937636,-1.1335948692
C,0.2718370554,4.5975412134,1.1353934092
C,-0.4855152081,5.9694550499,-1.1455989494
C,0.2374200559,5.9908540126,1.1483680764
C,-0.1457686785,6.6803983899,0.0020078981
C,-4.781977175,-1.3025273382,0.0740337709
C,-5.5399002517,-1.2981365831,-1.1005548661
C,-5.4958883092,-1.3242506577,1.2756911196
C,-6.9336950251,-1.3132617179,-1.0894537846
C,-6.8891322601,-1.3392960673,1.3156584527
C,-7.6113326738,-1.3342141617,0.1259873643
F,4.9932546856,-1.1987823763,2.3006791968
F,7.6922694346,-1.2091885677,2.2499778231
F,9.0222639171,-1.2182100437,-0.1388222739
F,7.6168703081,-1.2131092506,-2.4835293755
F,4.9175287133,-1.2027003854,-2.4484661291
F,-0.7531968733,3.9392008812,-2.2728237851
F,-0.8453885008,6.6343430212,-2.2586846071
F,-0.1840776875,8.0237428184,0.0014922487
F,0.5615544081,6.6750158595,2.2610525241
F,0.6316788477,3.9804281039,2.2744956262
F,-4.8430091067,-1.3284813463,2.4502255639
F,-7.5423047004,-1.3575052231,2.4923959009
F,-8.9555582385,-1.3478027605,0.1509709444
F,-4.9308264323,-1.2775181729,-2.2980302616
F,-7.6297830624,-1.3068489673,-2.2413908603
O,0.022379138,-6.1966090089,-0.0237891825
H,0.5851279171,-2.2828010406,-0.0154512025
H,0.552752304,-0.2319294131,-0.0024106541

Cartesian Coordination of 2^{-}-c

–3245.9953872 hartree

C,-1.3061702319,-3.8874974987,0.2136863031
C,1.2772441668,1.5441150221,-0.0852928658
C,3.5524436048,1.3307566682,-0.0739239466
C,2.9739398853,0.0332022702,-0.0024362343
C,2.9349578277,-2.4550120762,0.1344033447
C,2.5285909101,-4.6607233031,0.255747358
C,1.3059578945,-3.8875770595,0.2134592392
C,-1.2771742242,1.5441923524,-0.0850848144

C,-2.5284809227,2.2498457259,-0.1238285942
C,-2.9350954178,-2.4548323724,0.1349068097
C,-3.5567666857,-3.7588762888,0.206590471
C,-2.9739485467,0.0333841274,-0.0019296385
H,-4.6195537343,-3.9646782596,0.2178712958
H,-2.6287500546,3.323381929,-0.1828593115
H,4.6129597111,1.5397216381,-0.0856263897
N,1.5590587321,-2.5680721175,0.1408960234
N,-1.5592025748,-2.5679779611,0.1411405134
N,1.5962469711,0.2077864946,-0.0117647656
N,-1.5962464541,0.2078810347,-0.011545377
C,2.5285871778,2.2496871696,-0.1243293777
H,2.6289123918,3.3232133545,-0.1834464622
C,3.5565610705,-3.7590963659,0.2059455713
H,4.6193374216,-3.9649646988,0.2170216727
C,-2.5288432676,-4.6605665015,0.2562165026
C,-3.5523848661,1.330978077,-0.0732512692
H,-4.6128901798,1.5400107331,-0.0847211675
C,-0.0001240165,-4.5723433578,0.2513763773
C,-3.5997278747,-1.2166367121,0.0668225441
C,3.5996542808,-1.2168580767,0.0661964407
C,0.0000501889,2.1441043434,-0.1181484098
H,2.5709303105,-5.7384246798,0.3149876915
H,-2.571239081,-5.7382640387,0.3154866699
C,5.0969029238,-1.2223525197,0.0662089801
C,5.8332041837,-1.2981328472,-1.1210853927
C,5.8336585806,-1.1674955496,1.2543621507
C,7.2268723416,-1.3187421983,-1.1341057875
C,7.2273397625,-1.1865557032,1.2690351376
C,7.9263904555,-1.2637660211,0.0679439396
C,0.0000888787,3.6401136704,-0.2004484316
C,-0.0002665385,4.3148356692,-1.4277938692
C,0.0004825569,4.4453616057,0.9455125304
C,-0.0002378194,5.7058499046,-1.5191923051
C,0.0005262948,5.8380051359,0.8838116377
C,0.0001625587,6.4705458823,-0.3561137612
C,-5.0969767891,-1.2220374186,0.0671310655
C,-5.8335171716,-1.297793033,-1.1200163973
C,-5.8334945185,-1.1671132052,1.2554288341
C,-7.2271892508,-1.3183157891,-1.1327614892
C,-7.2271738682,-1.1860859791,1.2703770772
C,-7.9264665617,-1.2632743275,0.0694253338
F,5.2025342918,-1.0899467652,2.4373706159
F,7.9023932028,-1.1299683642,2.4313530223
F,9.2699186908,-1.2814807796,0.0686481051
F,7.9014961176,-1.3899956123,-2.295869523
F,5.2016162536,-1.3508235444,-2.3052132483
F,-0.0006422687,3.6214453505,-2.578521101
F,-0.000582725,6.3164696565,-2.7183097066
F,0.0001972697,7.8121301399,-0.4298909727
F,0.0009059553,6.576451761,2.0087285451
F,0.0008249155,3.8823419069,2.1653326083
F,-5.2021320569,-1.0895835107,2.4383117203
F,-7.9019945741,-1.1294356505,2.4328271055
F,-9.2699957418,-1.2809051555,0.0703960106
F,-5.2021661911,-1.3505433406,-2.3042677098
F,-7.9020465039,-1.3895479495,-2.2943908647
O,-0.0001557381,-5.8198531449,0.3201670358
H,-1.0111950089,-0.6308465775,0.0345605109
H,1.0111516799,-0.6309032681,0.0344713894

Cartesian Coordination of 2⁻-d

–3245.9991568 hartree

C,-1.2953978798,-3.9698470373,-0.0160487501
C,1.2758078761,1.4713975136,0.0062193215
C,3.544792167,1.3563297569,0.0095736046
C,2.9219118004,0.0356413919,0.0257986816
C,2.9697548234,-2.4561568884,0.0254772693
C,2.5162287918,-4.6722894954,0.0491217117
C,1.295441834,-3.9698309216,0.0175272261
C,-1.2758233209,1.471381843,-0.0060058474
C,-2.5261400194,2.2497760354,0.0005945959
C,-2.96972547,-2.4561935423,-0.0248046233
C,-3.5522009757,-3.7459297354,-0.0558846419
C,-2.9219099716,0.035604794,-0.0254931556
H,-4.6126833611,-3.9528927703,-0.0826115632
H,-2.6075343283,3.3281443569,0.0136289159
H,4.6064106827,1.5654708798,0.00377674
N,1.6043485636,-2.6238982386,0.0033517851
N,-1.6043244649,-2.6239157061,-0.00220527
N,1.5509890901,0.1458080482,0.0204793498
N,-1.5509888821,0.1457870134,-0.0200659422
C,2.526115647,2.2498044304,-0.0005843918
H,2.6074971297,3.3281715444,-0.0137976648
C,3.5522568491,-3.7458825826,0.0564943959
H,4.6127508656,-3.9528292502,0.0828866726
C,-2.5161629203,-4.6723223123,-0.0481140962
C,-3.5448060362,1.3562891378,-0.0095458133
H,-4.6064272953,1.5654202881,-0.0039005938
C,0.0000222646,-4.6514779516,0.0010502391
C,-3.6033698716,-1.1793933995,-0.0279529737
C,3.6033850593,-1.1793490407,0.0283164955
C,-0.0000112724,2.084522045,0.0000698479
H,2.578710559,-5.7497662675,0.0648540109
H,-2.5786259901,-5.7498013487,-0.0637672325
C,5.0965415301,-1.1736023056,0.0360610539
C,5.8434072581,-1.4693912943,-1.1119836703
C,5.831552063,-0.8848854882,1.1930581229
C,7.2367657713,-1.4743094615,-1.1181503128
C,7.2253121455,-0.8797923734,1.2129390536
C,7.9311885758,-1.1772144152,0.0511819948
C,-0.0000200305,3.5873998588,-0.0000613543
C,-0.0026173392,4.3292577498,-1.1874073516
C,0.0025683284,4.3294640676,1.1871558337
C,-0.0025372638,5.7231778755,-1.2029190898
C,0.0024718688,5.7233869346,1.2024248793
C,-0.0000368027,6.4236734048,-0.0003079692
C,-5.0965248451,-1.1736631563,-0.0360203663
C,-5.8312878125,-0.8851413437,-1.1932235425
C,-5.8436363878,-1.4692718937,1.1119108655
C,-7.2250435344,-0.8800642549,-1.2134069219
C,-7.2369962221,-1.4742025551,1.1177763505
C,-7.9311687264,-1.1773038165,-0.0517543442
F,5.1976316214,-0.6076289915,2.344925336
F,7.8944771634,-0.5982393531,2.3459170426
F,9.2750220869,-1.1761101636,0.0573648068
F,7.9174747439,-1.7540455244,-2.2446068937
F,5.2195906167,-1.7467464884,-2.2690144149
F,-0.0044631316,3.7006738475,-2.3756509031
F,-0.0045473668,6.3987877892,-2.3675417609

F,-0.0000447642,7.7682727391,-0.0004247119
F,0.0044739399,6.3991995508,2.3669298033
F,0.0044216211,3.7010870135,2.3755085825
F,-5.2200680042,-1.7464307436,2.2691223086
F,-7.9179463439,-1.7537614832,2.2441309807
F,-9.2750009499,-1.1762134634,-0.0582284752
F,-5.1971206383,-0.6080676759,-2.3449991971
F,-7.8939659078,-0.5987027716,-2.3465759099
O,0.0000372521,-5.9029811382,0.0004576156
H,-1.0189879611,-1.7836214585,0.0103155361
H,1.0189975018,-1.7836118078,-0.0090072805

Cartesian Coordination of 2⁻-e

–3245.9660485 hartree
C,-1.2609486603,-4.024168538,-0.004468265
C,1.2063140691,1.6049863041,-0.0108205795
C,3.459398799,1.2945668595,-0.0480043074
C,2.8182305483,0.0074926495,-0.0453832674
C,2.8409579404,-2.5056271646,-0.0603374682
C,2.5399844931,-4.7383217226,-0.068759484
C,1.2830320651,-4.0058745079,-0.0445660607
C,-1.3046555639,1.6054635198,0.0285820557
C,-2.5977170035,2.2909643656,0.0528285432
C,-2.7957041305,-2.4979731521,0.0284709361
C,-3.5002265039,-3.7772317473,0.0326004774
C,-2.8053560678,0.0359713913,0.043134147
H,-4.5713750931,-3.9338349005,0.0488834493
H,-2.765930377,3.3603042565,0.0616190798
H,4.5285429517,1.4508708397,-0.0638894882
N,1.499731337,-2.6610151149,-0.0400320886
N,-1.4587174193,-2.6858609812,0.006087905
N,1.4537710826,0.2466718681,-0.0225421786
N,-1.4571239413,0.2670295995,0.0233106338
C,2.4888341229,2.2579312139,-0.0272102078
H,2.6362359145,3.3283294706,-0.0233561911
C,3.5251389117,-3.7934651526,-0.078747979
H,4.5947583102,-3.9604405536,-0.0966624041
C,-2.5326841859,-4.7414852381,0.0118341274
C,-3.5359520301,1.3069537695,0.0619058065
H,-4.6109698385,1.4310768216,0.0794832851
C,0.0190872524,-4.6294674206,-0.0282389729
C,-3.4251125415,-1.223004466,0.0456804655
C,3.4655876887,-1.2307886432,-0.0627529858
C,-0.0393314789,2.2453992196,0.0124309374
H,2.6460450117,-5.8142083817,-0.0768018836
H,-2.7129973253,-5.8123997522,0.0089093813
C,4.9635040865,-1.2068168054,-0.0861667132
C,5.6842149491,-1.2022869395,-1.286197273
C,5.7215785662,-1.2163972365,1.0905888555
C,7.0777198683,-1.2037047464,-1.3225313555
C,7.115535923,-1.2179845045,1.0830830763
C,7.7963287752,-1.2126811235,-0.1307350208
C,-0.0257256643,3.7428996333,0.0209004697
C,-0.0486541728,4.4905129786,-1.1628890207
C,-0.0106084398,4.4767042827,1.2134217061
C,-0.0551094853,5.8844918188,-1.169319489
C,-0.0165861398,5.8705167608,1.2362546004
C,-0.0396708628,6.5773126702,0.0375964806
C,-4.9272194087,-1.2336371137,0.069325549
C,-5.6848932491,-1.2409452985,-1.1069448
C,-5.6474360347,-1.2533642027,1.2687559849
C,-7.0787443486,-1.261271761,-1.0996729611
C,-7.0408258968,-1.2738474156,1.3052162287
C,-7.7598080976,-1.2783379648,0.1137521947
F,5.1109038412,-1.2212950896,2.2871509035
F,7.8094029821,-1.2252210804,2.2365624149
F,9.1405379797,-1.2138485526,-0.1518701811
F,7.7349830612,-1.197131674,-2.4972575247
F,5.0362315086,-1.1931098421,-2.4629498576
F,-0.0634449414,3.8686113507,-2.3536995432
F,-0.0764117445,6.5671159004,-2.3294243771
F,-0.0453651636,7.9216786249,0.0454929126
F,-0.0006202764,6.5396039449,2.4043039646
F,0.0125934812,3.8410120795,2.3967925127
F,-4.9996996382,-1.251236122,2.4460849665
F,-7.6979572602,-1.2904357091,2.4804537938
F,-9.1045051916,-1.2983346551,0.1348508199
F,-5.0745956296,-1.2263429495,-2.3040205825
F,-7.7725995783,-1.2656613228,-2.2537204493
O,0.0842148408,-6.0041334291,-0.0372381048
H,0.7313673129,-0.4800375159,-0.0154063391
H,-0.8119252892,-6.3611227014,-0.0255812607

Cartesian Coordination of 2⁻-f

–3245.9655645 hartree
C,-1.2642597814,-4.0200777698,-0.0311705183
C,1.2016875441,1.6066368927,-0.0173914833
C,3.4549306291,1.2954136025,-0.0007467903
C,2.813671407,0.007306857,0.0098257275
C,2.8390313623,-2.5074542734,0.0334654175
C,2.5411110786,-4.736822044,0.1102691985
C,1.2792188781,-4.0149949223,0.0205574978
C,-1.3095402124,1.608347576,-0.0055242339
C,-2.6035273307,2.2904758102,-0.0188610626
C,-2.795398658,-2.4989845603,-0.0221663728
C,-3.4974568135,-3.7843039648,-0.074140565
C,-2.8062532939,0.0352652259,-0.0061121752
H,-4.5687742981,-3.9385002386,-0.1046658147
H,-2.7749172192,3.3591730604,-0.0308984794
H,4.5240033728,1.4520209504,0.0021343307
N,1.500848546,-2.6714307444,-0.0223072542
N,-1.4569067344,-2.681299368,0.006278158
N,1.4487782373,0.2473714444,-0.0051380852
N,-1.4588086572,0.2695067071,0.0021127288
C,2.4851724231,2.2583560243,-0.0131299999
H,2.6334891265,3.3285210434,-0.0168446515
C,3.5245597973,-3.7866426023,0.1180058736
H,4.5918276109,-3.9529235004,0.1889801108
C,-2.5287563178,-4.7439629532,-0.082417807
C,-3.5396146869,1.3036172569,-0.016613422
H,-4.614991841,1.4251317498,-0.0240573404
C,0.0073073555,-4.6323099419,-0.0195592951
C,-3.4240621128,-1.2265252966,-0.015820548
C,3.4633221037,-1.2289313121,0.0309279068
C,-0.0429962401,2.248663594,-0.0173837075
H,2.7039524391,-5.8072722595,0.1884013674
H,-2.6498772387,-5.8178388628,-0.1203043511
C,4.9596764462,-1.2046324856,0.0625649162
C,5.7257618137,-1.4489682403,-1.0843469351
C,5.6754924909,-0.9653985939,1.2424010506

C,7.1194643113,-1.4544457231,-1.0677257726
C,7.0687301292,-0.9622711736,1.2863093476
C,7.7941052263,-1.2100208374,0.1247845246
C,-0.0294855871,3.745131315,-0.0323313925
C,0.1829301088,4.4753881022,-1.2086813234
C,-0.2506283723,4.498278871,1.1279277501
C,0.1801243395,5.8689354759,-1.2375035134
C,-0.2624161351,5.892123464,1.1267387997
C,-0.0456700074,6.580654139,-0.0630992018
C,-4.9266620028,-1.2376042231,-0.0309570718
C,-5.6545821117,-1.2294109346,-1.2256028121
C,-5.6757821183,-1.2732969061,1.1500172579
C,-7.048108684,-1.2511031711,-1.2529360397
C,-7.0695747917,-1.2952703282,1.1517730369
C,-7.7588612759,-1.2846212352,-0.0570421437
F,5.0224403699,-0.7326905723,2.3935996503
F,7.7195887294,-0.7296926388,2.4414657853
F,9.1381739752,-1.2111983634,0.1541787504
F,7.8194969667,-1.6882709779,-2.1934739841
F,5.122508223,-1.6788982833,-2.2624606354
F,0.3958135832,3.8358700058,-2.371427798
F,0.3871689385,6.5334877013,-2.3898833005
F,-0.0520998524,7.9250530446,-0.0777428381
F,-0.4746779292,6.5790269385,2.2648136924
F,-0.4525147576,3.8815892037,2.3042810629
F,-5.056907477,-1.284773836,2.3426289986
F,-7.7554861558,-1.3269281691,2.3101306751
F,-9.1037389664,-1.3054841772,-0.0695074002
F,-5.0146644754,-1.1985289981,-2.4068654838
F,-7.7132870834,-1.2405555593,-2.4237679102
O,-0.0473265468,-6.0069304789,-0.0403121372
H,0.7233605941,-0.4762811805,-0.0064009399
H,0.8464416092,-6.3582683548,-0.1316680249

Cartesian Coordination of 2^{-}-g
–3245.9633866 hartree
C,-1.2137689516,-4.0120370944,-0.0496464656
C,1.2706055282,1.5692485696,0.008141845
C,3.5190285357,1.3332409387,0.0272600419
C,2.8110836217,0.0453600792,0.0241892282
C,2.8071688094,-2.4860668863,0.0255289593
C,2.5858310242,-4.7411786399,0.0356198016
C,1.3062810453,-4.0508804397,-0.0000149698
C,-1.2610799362,1.5732805406,-0.0148481592
C,-2.5338863306,2.3105541526,-0.0077211639
C,-2.8226749349,-2.4276590894,-0.0515406162
C,-3.4559766591,-3.7127074708,-0.0885278742
C,-2.8395619127,0.0801094847,-0.0414876756
H,-4.523803441,-3.8750651326,-0.1166895989
H,-2.6532089368,3.386067739,0.0087979503
H,4.5909147541,1.4851681959,0.0351075374
N,1.4703265785,-2.7048293878,-0.0041376367
N,-1.4649737239,-2.6496585181,-0.0298074486
N,1.4646435197,0.2368575768,0.0121933968
N,-1.4936375777,0.2328910594,-0.0348112858
C,2.5545059147,2.2890135953,0.0171866081
H,2.6978646662,3.3621060383,0.0154127622
C,3.5303676199,-3.7538256619,0.0503546903
H,4.6041862965,-3.8861591659,0.0799038156
C,-2.4748231961,-4.6755520432,-0.0864956163
C,-3.5201119573,1.3783529994,-0.0219427331
H,-4.5887815446,1.550229988,-0.017234843
C,0.0332313008,-4.6536858267,-0.032091738
C,-3.4763630182,-1.1748189449,-0.0509891506
C,3.4370494388,-1.211253564,0.0332678752
C,-0.0002625108,2.2002341425,-0.0008792172
H,2.7815197775,-5.8085942661,0.0567772807
H,-2.598024188,-5.7479782656,-0.1089922184
C,4.9387246036,-1.214239668,0.0533810915
C,5.6937397224,-1.2368129216,-1.1245870571
C,5.662588305,-1.1970945341,1.2508235669
C,7.0877318837,-1.2393623037,-1.1209677535
C,7.0562341073,-1.1993114199,1.2837248548
C,7.7721220545,-1.2206602484,0.090579421
C,0.0046295724,3.7020094069,0.0056439344
C,0.0119948083,4.4471727627,-1.1790736369
C,0.0125548057,4.4375917235,1.1963494899
C,0.0240335838,5.8411188326,-1.1884209323
C,0.0243508366,5.8313963594,1.2167109699
C,0.030356564,6.5364568388,0.0169738862
C,-4.9716537751,-1.2004875603,-0.0603934547
C,-5.709722438,-0.953923662,-1.225368668
C,-5.7178046627,-1.4624269582,1.0962281114
C,-7.1035164667,-0.9613275754,-1.2462493039
C,-7.1113447585,-1.4798480526,1.1024262827
C,-7.8077206151,-1.2264333721,-0.0756742639
F,5.0180946477,-1.1793292347,2.4298245788
F,7.7166385271,-1.1824594467,2.4571240002
F,9.1169925696,-1.2235377619,0.1082588267
F,7.7786144214,-1.2603998276,-2.2766392338
F,5.0802649525,-1.2567526795,-2.320009058
F,0.0067487112,3.8232798482,-2.3692964649
F,0.0296776065,6.5216296247,-2.3505388767
F,0.0414847972,7.881516104,0.0226521869
F,0.0295937872,6.5027385884,2.3841839542
F,0.0064537765,3.8042079194,2.3816936199
F,-5.0937181238,-1.7017756178,2.2621240856
F,-7.7909867222,-1.7315118021,2.2371057405
F,-9.1522352387,-1.2385103719,-0.0826271508
F,-5.0782116797,-0.7075639526,-2.3854738658
F,-7.7752781493,-0.7227263785,-2.3883074604
O,-0.0601334744,-6.0304752107,-0.0450199911
H,0.829819473,-6.4003724006,-0.0864268755
H,-0.7552056248,-1.9096987507,-0.0158509358

Cartesian Coordination of 2^{-}-h
–3245.9612987 hartree
C,-1.2078456723,-4.0215055863,0.0459376736
C,1.2729919224,1.5726712345,-0.0124079036
C,3.5205495831,1.3337313965,-0.0038483072
C,2.8108070825,0.0468073044,-0.0117651643
C,2.8063201825,-2.4842498936,-0.0227273272
C,2.5849773269,-4.7411774119,-0.0903321332
C,1.3101326028,-4.0462677569,-0.0270021056
C,-1.2582562773,1.5737193881,-0.0115886288
C,-2.5309313543,2.3102772584,-0.0262221318
C,-2.817867477,-2.4282468366,0.054422554
C,-3.4538658297,-3.7065301721,0.1271308542
C,-2.8360521982,0.0804061724,0.022889745
H,-4.5201365092,-3.867237085,0.1909677979

H,-2.6505327137,3.3856282641,-0.0496009103
H,4.5926547997,1.4840014055,0.0028016798
N,1.4687358937,-2.6995987677,0.0139526802
N,-1.4608852258,-2.6585078352,0.0106688706
N,1.4661000145,0.2393292875,-0.0159773991
N,-1.4900207429,0.2333367739,0.0145897629
C,2.5572538125,2.291079038,-0.0045896101
H,2.7017970514,3.3639990993,0.0005278893
C,3.5290486103,-3.7576535303,-0.0823934063
H,4.6031851602,-3.8862794498,-0.120427208
C,-2.4744423438,-4.6737342441,0.124466615
C,-3.5171055409,1.3779266184,-0.0069582956
H,-4.5857475406,1.5498157936,-0.0142448967
C,0.0465640624,-4.6573595344,-0.0085967393
C,-3.4719174682,-1.1745080766,0.046440868
C,3.436169273,-1.2123866894,-0.0199103712
C,0.0025422908,2.2020992638,-0.0182889448
H,2.7281493921,-5.8119716776,-0.1339940848
H,-2.6450329932,-5.7399352863,0.2043843947
C,4.9381164301,-1.2152938486,-0.0341418454
C,5.6669201575,-1.1770731456,-1.2279480556
C,5.6877724422,-1.2581920198,1.1465510405
C,7.0606258668,-1.1796087077,-1.254924286
C,7.0817327818,-1.2612001619,1.1488182718
C,7.7713093221,-1.2219442185,-0.0592551732
C,0.0059880749,3.7039232065,-0.0288848031
C,0.0191536436,4.4359609763,-1.2216589938
C,0.0068888871,4.4523440058,1.1537659732
C,0.0299119981,5.829702728,-1.2459321804
C,0.0179035155,5.8463061285,1.1591614281
C,0.029556112,6.5381722486,-0.0481972093
C,-4.9666030726,-1.2010304462,0.0666041243
C,-5.7208145393,-1.5041365634,-1.0749784455
C,-5.6978246205,-0.9133221111,1.2268742243
C,-7.1142780725,-1.5219587916,-1.0716645408
C,-7.0914667488,-0.9196417587,1.2566774446
C,-7.8033105973,-1.2263481996,0.1009331488
F,5.0689149439,-1.2968043796,2.3386833562
F,7.7677039644,-1.3014853772,2.3069052913
F,9.1163271146,-1.2247356516,-0.0711290417
F,7.7261055087,-1.1426137476,-2.4250571988
F,5.0272945258,-1.1386631766,-2.4091631794
F,0.0193013089,3.7990695069,-2.4051489167
F,0.0402841225,6.4976901456,-2.415268157
F,0.0396162196,7.8831965325,-0.0576947227
F,0.0171659127,6.5301223623,2.3193025932
F,-0.004163387,3.8317571383,2.3456248136
F,-5.0594926816,-0.6285970266,2.3742162733
F,-7.7558592977,-0.6417308425,2.3940139097
F,-9.1476864189,-1.2384429011,0.1170195874
F,-5.1045864551,-1.7839144441,-2.2363949475
F,-7.800859682,-1.8137745599,-2.1925436956
O,0.0752511643,-6.0385763117,-0.0251989272
H,-0.7450285493,-1.923965639,-0.0141937879
H,-0.7913910684,-6.3766804139,-0.2797721903

Cartesian Coordination of 2ni

–3414.4908501 hartree
C,1.2165101347,-3.5455013215,-1.4893331557
C,-1.2160188001,1.7162103852,0.0008346145
C,-3.4018786633,1.3671082008,0.3921999301
C,-2.7310005784,0.1311919179,0.0670676954
C,-2.7367100539,-2.2053187056,-0.6460297602
C,-2.4906647255,-4.1803849707,-1.6967605274
C,-1.235993891,-3.5098369538,-1.5422070209
C,1.2236071055,1.7199150746,-0.0468845992
C,2.4921841416,2.3622853184,0.2040582226
C,2.7227411135,-2.2275745709,-0.6091768328
C,3.3952556417,-3.4402905196,-0.9782885066
C,2.7391607809,0.1355909419,0.0112936089
H,4.4472500434,-3.6389530512,-0.8378111001
H,2.6338267316,3.4246970706,0.3362947978
H,-4.4510397421,1.4615477231,0.6287620019
N,-1.3921849184,-2.2966200389,-0.9077750761
N,1.385024586,-2.3067279409,-0.9087375598
N,-1.3853310357,0.3610973712,-0.1512287022
N,1.3886365527,0.3621639292,-0.1782839706
C,-2.4746619545,2.3519797774,0.3097631778
H,-2.611525169,3.4109357921,0.4711782122
C,-3.4237731699,-3.3814220168,-1.1080369419
H,-4.4862504066,-3.5546912191,-1.0216553845
C,2.459963413,-4.2532524748,-1.5472333217
C,3.4214050642,1.378985214,0.2805491922
H,4.4777335136,1.4777870763,0.4812232726
C,-0.0120021063,-4.076868574,-1.8834080653
C,3.3770126674,-1.0881894401,-0.1193639305
C,-3.3799254851,-1.0823813091,-0.1085047783
C,0.002866381,2.3908329868,-0.0246791948
H,-2.6207124681,-5.143084146,-2.1678928853
H,2.6188423346,-5.2582128115,-1.9173096042
C,-4.8366288045,-1.1852447185,0.1999520699
C,-5.2814993505,-1.8947073776,1.320952937
C,-5.8138278372,-0.603210861,-0.6142564554
C,-6.6347411416,-2.0166247067,1.6280436116
C,-7.172682877,-0.7077956862,-0.3250326129
C,-7.5828588722,-1.4188183452,0.8006926784
C,0.0030379485,3.8810747581,0.0835937621
C,0.0096551585,4.5313981398,1.3214365855
C,-0.0031828974,4.6853233657,-1.0601782796
C,0.0093943049,5.920806182,1.4243625221
C,-0.0029244095,6.0767010402,-0.982931994
C,0.0032552267,6.6947029094,0.2655770291
C,4.8336020561,-1.1900668526,0.191852933
C,5.2729650929,-1.8083883947,1.3673669863
C,5.8148878687,-0.6940120824,-0.6723784433
C,6.6258920668,-1.9259557635,1.6781174017
C,7.173586324,-0.7956700136,-0.3810376974
C,7.5786709603,-1.4155070313,0.799166088
F,-5.4541025111,0.0807201272,-1.7088051858
F,-8.0830809459,-0.1376594781,-1.1217764817
F,-8.8822370908,-1.5265935087,1.0863460994
F,-7.0279005966,-2.6974070048,2.7096106554
F,-4.3950308398,-2.4790220799,2.1367173732
F,0.0156577991,3.8143899185,2.4528291621
F,0.0155169297,6.5141210666,2.6230251595
F,0.0033016869,8.0267737481,0.3521951518
F,-0.0088613341,6.8192407223,-2.0949587698
F,-0.0093827145,4.1214951186,-2.2744774498
F,5.4589215477,-0.0981225635,-1.8183150151
F,8.088572679,-0.3098868486,-1.2268639479

F,8.8777938664,-1.5200193335,1.0870678269
F,4.381505758,-2.3074519252,2.2328507325
F,7.0141377141,-2.5197497291,2.8114383241
Ni,-0.0019483809,-0.9659791708,-0.5372760404
O,-0.078955055,-5.2664481,-2.5410536918
H,0.8002806328,-5.5144552362,-2.8523248325

Cartesian Coordination of 2ni⁻

–3414.1711764 hartree
C,-1.2473084494,-3.4789102685,-1.6908508228
C,1.2339951539,1.7019025887,-0.0113989015
C,3.4193720444,1.3198148539,0.3862273051
C,2.7343404489,0.0930282178,0.0685017302
C,2.7122496411,-2.2630909858,-0.6373995333
C,2.4500745231,-4.2117507584,-1.7297557799
C,1.228651783,-3.4931361432,-1.6734659109
C,-1.2164820391,1.7159028765,-0.0284523082
C,-2.481580174,2.3499125571,0.2624232227
C,-2.7309417238,-2.2320597185,-0.6751182503
C,-3.4076033307,-3.4152813837,-1.1044778416
C,-2.7359706874,0.1241884173,0.0306556898
H,-4.4579577446,-3.6266571349,-0.957012417
H,-2.6286687534,3.4091818253,0.420487206
H,4.4715724312,1.4067776237,0.618927482
N,1.3793999088,-2.3092592782,-1.0014548197
N,-1.393806402,-2.2934236349,-1.0207342885
N,1.3898299417,0.3476484384,-0.165781258
N,-1.3855643619,0.3634955038,-0.1851014088
C,2.5020985159,2.3215114806,0.2969098924
H,2.6590196032,3.3790523351,0.457064548
C,3.3812114867,-3.454072796,-1.0572559773
H,4.4269329411,-3.677419655,-0.8952291277
C,-2.4759634279,-4.1835216219,-1.7641780832
C,-3.4113263785,1.3587127208,0.3390078009
H,-4.4655716261,1.4576567351,0.557217941
O,-0.0091687782,-5.0240446383,-2.962690474
C,-0.0088945731,-4.0375446206,-2.1956984482
C,-3.3619493496,-1.100555305,-0.1279849387
C,3.3484794309,-1.138768583,-0.0815977442
C,0.0126249344,2.3823142952,-0.0242157868
H,2.563564761,-5.172864837,-2.2095691004
H,-2.593771689,-5.1432688783,-2.2456840353
C,4.7958253339,-1.2653644675,0.272220367
C,5.8166374862,-0.8635265915,-0.5969190189
C,5.1996715262,-1.8134307067,1.4953688468
C,7.1664209624,-0.9914239589,-0.2722192211
C,6.5420573319,-1.9542718412,1.8437163639
C,7.5299247519,-1.5404870269,0.9542681367
C,0.0202042181,3.8697202091,0.1073010979
C,0.0325597256,4.7029773436,-1.0186321707
C,0.0154107883,4.5135914675,1.3513325183
C,0.0398033121,6.0939150175,-0.9220659383
C,0.0224304449,5.9019181946,1.4771766314
C,0.034703965,6.6955078285,0.3332713684
C,-4.8153341678,-1.210730521,0.2059922465
C,-5.8195706876,-0.7973092873,-0.6769176958
C,-5.2420272147,-1.7542603341,1.4233958823
C,-7.1750219038,-0.9099162378,-0.3707009913
C,-6.5905555759,-1.8798944287,1.7533576005
C,-7.5614353998,-1.4548895375,0.8505969836
F,4.2820689987,-2.2197916817,2.3873528802
F,6.8919051223,-2.4807994732,3.0309844557
F,8.8274021046,-1.6681179723,1.2787233693
F,8.1204934116,-0.5915085997,-1.132299916
F,5.5154184888,-0.3339266902,-1.7941831993
F,0.0378879072,4.1699643152,-2.2512135546
F,0.0516399444,6.8598070991,-2.0279850546
F,0.0415618681,8.0347724065,0.4407092309
F,0.0174669734,6.4825245852,2.6912479841
F,0.0035705063,3.7912699938,2.4844319042
F,-4.3413572002,-2.1710393326,2.3277409871
F,-6.9625193741,-2.4024914805,2.9356284446
F,-8.8645861831,-1.5678255257,1.1572818814
F,-5.4960523652,-0.2711302782,-1.8698674318
F,-8.112670118,-0.4991806062,-1.2436488306
Ni,-0.002472042,-0.9701571092,-0.5991667197

Cartesian Coordination of 2zn

–3310.7656464 hartree
C,-1.2827273994,-4.0561042041,-0.125532227
C,1.2476655676,1.5643685982,0.0364770231
C,3.4904885243,1.2812544302,0.0011237396
C,2.8130673961,-0.0001249211,-0.0288577292
C,2.8028884356,-2.5124858617,-0.1032714964
C,2.5080909652,-4.7519592574,-0.1363570118
C,1.2417426097,-4.0718034045,-0.1438697454
C,-1.2630901462,1.5715065518,0.0602112571
C,-2.5418757711,2.2547234637,0.0554841352
C,-2.835488621,-2.4987761504,-0.0481731815
C,-3.5098622637,-3.7747401288,-0.0661268704
C,-2.8345501506,0.0133224883,0.0279603419
H,-4.5794787722,-3.9222994483,-0.0350006888
H,-2.6824988349,3.3256412277,0.0540189498
H,4.56065694,1.426977433,-0.0158224683
N,1.4490152548,-2.712918811,-0.1218525902
N,-1.4805908854,-2.6982342205,-0.0815854551
N,1.4512565084,0.2096149834,-0.0080192173
N,-1.4722640138,0.217859798,0.0417006357
C,2.5301624935,2.2414361919,0.046313876
H,2.6755628652,3.3109089062,0.0875581347
C,3.4758487593,-3.7858456312,-0.1103674036
H,4.5448481253,-3.9375384963,-0.0846991022
C,-2.5446492627,-4.7407578125,-0.1180957747
C,-3.5065993931,1.2972484402,0.0355480539
H,-4.5762234486,1.4466988161,0.0144668633
C,-0.0261485171,-4.6909607801,-0.1610224215
C,-3.4733387965,-1.2337362594,-0.0040807314
C,3.4468787823,-1.2481203549,-0.0704483273
C,-0.0053061991,2.2084178209,0.0662709656
H,2.6866074979,-5.8207357557,-0.1351459201
H,-2.6720767624,-5.8127310293,-0.144522239
C,4.9424672873,-1.2564086646,-0.0759038933
C,5.6662348623,-1.5918341901,-1.2253399479
C,5.6815181812,-0.9348897344,1.0676325836
C,7.0589592147,-1.6096101523,-1.2432673956
C,7.0747260759,-0.939751824,1.0727610578
C,7.7651661538,-1.2802360417,-0.088302745
C,-0.0017841733,3.7037572543,0.1018829992
C,0.3330834539,4.4618617272,-1.0251846682
C,-0.3358751967,4.4083633711,1.2634445092

C,0.341250124,5.8547523856,-1.0038794905
C,-0.3411335179,5.8008137783,1.3080074341
C,0.0007184887,6.5258042935,0.168554479
C,-4.9690893657,-1.2342588047,0.0062369153
C,-5.7072632806,-1.5590571776,-1.136850383
C,-5.6925814591,-0.9141902588,1.1598795148
C,-7.1001641989,-1.568767543,-1.1387299742
C,-7.0856316655,-0.9120228166,1.1812058041
C,-7.7910885783,-1.2422701615,0.0262579867
F,5.0503533058,-0.6089836209,2.2035115901
F,7.7508869606,-0.6271867865,2.1835390325
F,9.1000931094,-1.2911117748,-0.0942778795
F,7.7200098915,-1.9338325812,-2.3598685708
F,5.0185581873,-1.9092199952,-2.3544539585
F,0.6609997952,3.8485256614,-2.17058653
F,0.6663503533,6.5496140195,-2.0996516942
F,0.0023396437,7.8605528835,0.1998710383
F,-0.6648135276,6.4435336445,2.4354649665
F,-0.6653947354,3.742354807,2.3780861449
F,-5.0460960612,-0.5973896252,2.2895340783
F,-7.7472749335,-0.6015264727,2.3012999594
F,-9.126108266,-1.245498585,0.0353884888
F,-5.0741839174,-1.8722017413,-2.2752473902
F,-7.7760279685,-1.881976314,-2.2495958154
Zn,-0.0143857912,-1.2381884759,-0.0441778757
O,-0.1052012075,-6.0504729436,-0.2047208181
H,0.7783268585,-6.4299302325,-0.2824990172

Cartesian Coordination of 2zn⁻

–3310.450939 hartree
C,-1.2839261113,-4.0843234506,-0.0002465265
C,1.2583892551,1.5735510354,0.0000875599
C,3.5055222396,1.2889579142,0.0000478115
C,2.8325748297,0.0088832508,-0.0000670498
C,2.8360603615,-2.5177125537,-0.0001922191
C,2.5366883576,-4.7575912414,-0.0004546187
C,1.2839036318,-4.0843342331,-0.0002840844
C,-1.2581361007,1.5736398063,0.0001146677
C,-2.5394187378,2.2534330897,0.0001769932
C,-2.8359898017,-2.5175938775,-0.0001457813
C,-3.5122821673,-3.7807847618,-0.0001751637
C,-2.8323861042,0.0090140437,0.0000328992
H,-4.58418723,-3.9276231256,-0.0001439436
H,-2.6865147316,3.3248786374,0.0002514582
H,4.5766740529,1.4395952954,0.0000630421
N,1.4728489728,-2.7297745396,-0.0001538125
N,-1.4727876926,-2.7297481775,-0.0001829323
N,1.4627730316,0.2201872294,-0.0000344657
N,-1.4625705633,0.2202821338,0.0000292406
C,2.5396935207,2.2533051372,0.0001281044
H,2.6868291657,3.3247448496,0.000212363
C,3.5122819152,-3.780948704,-0.0002932077
H,4.5841795856,-3.9278423358,-0.0003225116
C,-2.5367447221,-4.7574935238,-0.0002897672
C,-3.5052798216,1.2891216813,0.0001268217
H,-4.576425485,1.439802256,0.0001491142
C,-0.0000204652,-4.7968522128,-0.0002996002
C,-3.4577205446,-1.2391055216,-0.0000513385
C,3.4578577424,-1.239267509,-0.0001340243
C,0.0001414062,2.2136534678,0.0001492543
H,2.6533780389,-5.8315952395,-0.0006037842
H,-2.6535111893,-5.8314894775,-0.0003608
C,4.9574511531,-1.2406888493,-0.0001341808
C,5.6947711168,-1.2485099061,-1.189225227
C,5.6947621318,-1.2494143112,1.1889580643
C,7.0887839544,-1.2608993718,-1.2036174615
C,7.0887734882,-1.2618162886,1.2033514376
C,7.788572685,-1.2681229743,-0.0001334607
C,0.0001862119,3.710183595,0.0002642947
C,0.0000304595,4.4518154031,-1.1879202322
C,0.0003795558,4.4516310029,1.1885644738
C,0.0000636347,5.8459327071,-1.2029517973
C,0.0004201215,5.8457457805,1.2038112065
C,0.0002623428,6.5458417995,0.0004840598
C,-4.9573138242,-1.2404603235,-0.0000296869
C,-5.6946456184,-1.2487626943,-1.1891118349
C,-5.6946154658,-1.2486442365,1.1890711602
C,-7.0886581668,-1.2611023225,-1.2034864287
C,-7.0886280699,-1.2609808944,1.2034826629
C,-7.7884374428,-1.2677780874,0.00000753
F,5.063424951,-1.2420793861,2.3748113507
F,7.7635482002,-1.2659764021,2.3672383115
F,9.1321823779,-1.278534093,-0.000131473
F,7.7635660075,-1.2641920898,-2.3675032373
F,5.0634449195,-1.2403224901,-2.3750814236
F,-0.0001526246,3.8225034903,-2.3753674155
F,-0.0000881508,6.5215150227,-2.3668359487
F,0.0002968964,7.8896327762,0.0005898893
F,0.0006055618,6.5211486644,2.3677993982
F,0.0005280895,3.8221353234,2.3759146039
F,-5.0632689676,-1.2408560886,2.3749186145
F,-7.7633915211,-1.2646228863,2.3673780958
F,-9.1320474595,-1.278132211,0.0000247126
F,-5.063328678,-1.2410753794,-2.3749746907
F,-7.7634518442,-1.2648539275,-2.3673637935
Zn,-0.0000377511,-1.2605067556,-0.0000823488
O,-0.0000236435,-6.0469209386,-0.0004182938

Cartesian Coordination of 2pd

–3371.9147926 hartree
C,-1.2682699399,-4.0388462597,-0.1887881967
C,1.2336489771,1.5524768569,0.0247955595
C,3.4725486975,1.2672284603,-0.0139498629
C,2.7976869291,-0.0127755633,-0.0033238572
C,2.7885221777,-2.4984696916,-0.0373113291
C,2.4911573996,-4.7344919061,-0.0671856648
C,1.2274728878,-4.0550706827,-0.1222456186
C,-1.2492183548,1.5570716053,0.0470314289
C,-2.5270588187,2.2346552589,0.0905166404
C,-2.8225713644,-2.4839777472,-0.1159292797
C,-3.493717399,-3.7582635371,-0.1817006466
C,-2.8191293963,-0.0015879134,-0.0015892606
H,-4.5643469953,-3.8989926618,-0.1835841933
H,-2.6619794008,3.3045498633,0.1448610128
H,4.5430209242,1.4057085483,-0.0432906128
N,1.4350674849,-2.6962128416,-0.0951023018
N,-1.4673304238,-2.6826775516,-0.1188303417
N,1.4364973843,0.1976365284,0.0132514946
N,-1.4571993916,0.2039427059,-0.0044407008
C,2.5143353031,2.2266512456,0.0032673716

H,2.6532538848,3.2973507465,-0.0086779359
C,3.4565643067,-3.7722320611,-0.0140414704
H,4.524240337,-3.9186866054,0.0504433152
C,-2.5297914848,-4.7206056868,-0.2331176661
C,-3.489126161,1.2788540242,0.0566304165
H,-4.5596506323,1.4200943825,0.0651762018
C,-0.0251661624,-4.6812647404,-0.1938192467
C,-3.4670681872,-1.2345609218,-0.0554117624
C,3.4403309253,-1.2489664984,-0.0105042715
C,-0.0061358678,2.2035550642,0.0507372222
H,2.6596623077,-5.8041027677,-0.0452234855
H,-2.6494855896,-5.7920314443,-0.2888854378
C,4.9345980166,-1.2560106378,0.0103866871
C,5.6789811352,-1.6233993912,-1.1160421846
C,5.6517805924,-0.899221413,1.1576419103
C,7.0718009955,-1.6389497356,-1.1079782688
C,7.0447044552,-0.901565996,1.1877943739
C,7.7562882621,-1.2745886613,0.049637776
C,-0.0028181509,3.6979535466,0.084543239
C,-0.3544212217,4.4546388414,-1.0384211544
C,0.3526126151,4.4022316331,1.2399397926
C,-0.3576136542,5.8476083347,-1.018751687
C,0.3627752139,5.7947470641,1.2821258562
C,0.0043450931,6.5189513616,0.1471816819
C,-4.9623949721,-1.2341895452,-0.0488240576
C,-5.6968169382,-1.484660505,-1.2123945226
C,-5.6865398176,-0.9891111294,1.1222077535
C,-7.0898948071,-1.493694739,-1.2173782726
C,-7.0796605039,-0.9882425325,1.1408921477
C,-7.7825389801,-1.2430901182,-0.0346830756
F,4.9996456216,-0.5411766119,2.2709755651
F,7.7002275048,-0.5552064375,2.3004987755
F,9.090837551,-1.2831975171,0.0680032133
F,7.753446237,-1.994369901,-2.2022047945
F,5.0523276606,-1.9747024673,-2.2466235484
F,-0.7034750106,3.8403208466,-2.1763506721
F,-0.6987039897,6.541933648,-2.109604401
F,0.0075971344,7.8534557925,0.1765762118
F,0.7074281798,6.438030758,2.4026974677
F,0.6988678862,3.7364156897,2.349095326
F,-5.0406253475,-0.7439287318,2.2695358924
F,-7.7437000596,-0.7499720135,2.2768713497
F,-9.1173313459,-1.2465576258,-0.0280143855
F,-5.060293263,-1.7264159695,-2.3655283238
F,-7.7637084028,-1.7350002816,-2.3469293112
Pd,-0.0138319995,-1.2416330619,-0.0528852584
O,-0.1026380589,-6.0396077476,-0.2595948865
H,0.7780315219,-6.4145290342,-0.379849816

Cartesian Coordination of 2pd^{-}

–3371.5975289 hartree
C,-1.2648168137,-4.064564466,-0.0004653267
C,1.2455510128,1.5614528514,-0.0002240686
C,3.4891784011,1.2769543719,-0.0001661493
C,2.8179216887,-0.0010048886,-0.0003435703
C,2.8167730675,-2.5000559253,-0.0003778368
C,2.5177885154,-4.7355845275,-0.0003677528
C,1.2648097225,-4.0646237193,-0.0004309983
C,-1.2452878311,1.5615110134,-0.0001333867
C,-2.5246631681,2.2391215907,0.0000923744
C,-2.8167036423,-2.4999229445,-0.000372416
C,-3.4910572618,-3.7630017849,-0.0002969889
C,-2.8177308653,-0.0008695711,-0.0001844935
H,-4.5632933142,-3.9033855169,-0.0001660975
H,-2.6649577167,3.3110125523,0.0002665341
H,4.5607302409,1.4210134398,-0.0000690503
N,1.4523097622,-2.7113561481,-0.000479052
N,-1.4522521343,-2.711290218,-0.0005728189
N,1.4499288685,0.2087608659,-0.0004579832
N,-1.4497297867,0.208831146,-0.0003296564
C,2.5249603303,2.2389987048,-0.0000919502
H,2.6653069546,3.310882423,0.0000727122
C,3.4910644448,-3.7631671242,-0.000205657
H,4.5632936141,-3.9036021978,-0.0000602434
C,-2.5178286338,-4.7354664521,-0.00042357
C,-3.4889268634,1.2771225966,0.0000680425
H,-4.5604718943,1.4212305816,0.0002160813
C,-0.0000199893,-4.7865030952,-0.000441929
C,-3.4493777193,-1.2381712371,-0.0002358058
C,3.4495096852,-1.2383356138,-0.0003457604
C,0.000146321,2.2088186334,-0.0001126563
H,2.6249675284,-5.8103098414,-0.0003517992
H,-2.6250631366,-5.8101861129,-0.0004053095
C,4.9478863816,-1.2418536037,-0.0001550189
C,5.6846654173,-1.2502589686,-1.1896302974
C,5.6843550771,-1.2506249106,1.189510835
C,7.0786979303,-1.2636359431,-1.2035308809
C,7.078382901,-1.264010457,1.2037722939
C,7.7781441222,-1.2709884911,0.0002106152
C,0.0001783257,3.7043866335,0.0001544122
C,0.000043708,4.4451410819,-1.1885061536
C,0.0003371289,4.4447136203,1.1890815627
C,0.0000720633,5.8392848151,-1.2030212222
C,0.0003726713,5.8388518666,1.2040970747
C,0.0002423831,6.5388770122,0.0006637908
C,-4.9477550294,-1.2416201422,-0.000025433
C,-5.6845465367,-1.2501489633,-1.1894930196
C,-5.6842121277,-1.2502046114,1.1896480248
C,-7.0785792099,-1.2634685679,-1.2033778563
C,-7.078240986,-1.2635226027,1.2039252385
C,-7.778014184,-1.2706284815,0.0003720082
F,5.0525474878,-1.2427066116,2.374501961
F,7.7530109072,-1.2683983763,2.3674665332
F,9.1215095494,-1.2823913818,0.0003844251
F,7.7536293245,-1.2676711328,-2.3670503982
F,5.0531692994,-1.2419888925,-2.3747864694
F,-0.0001142361,3.8154669531,-2.3751069775
F,-0.0000562113,6.5149017187,-2.3665387006
F,0.0002715227,7.8823847842,0.0009067036
F,0.000529868,6.5140513059,2.3678568262
F,0.0004656676,3.8146130286,2.3754562046
F,-5.052393256,-1.2421661772,2.3746333366
F,-7.7528567482,-1.2677306428,2.3676273821
F,-9.1213801544,-1.2819701234,0.0005600325
F,-5.0530610713,-1.2420502083,-2.3746550559
F,-7.75352331,-1.2676202975,-2.3668895889
O,-0.0000495283,-6.0375862366,-0.0003733332
Pd,0.0000707354,-1.2519343823,-0.0008576431

Cartesian Coordination of 2pt

–3364.3565272 hartree
C,-1.2687725812,-4.0397954415,0.0972705115
C,1.2335288713,1.5519942046,-0.0287366007
C,3.4714196109,1.2625176461,-0.0071209718
C,2.7973072912,-0.0159866095,-0.0255073864
C,2.7857418694,-2.4978701276,-0.0212577559
C,2.4866082174,-4.7330213288,-0.0129273996
C,1.2222107871,-4.0564346013,0.0389410173
C,-1.245794137,1.5587226087,-0.0408556107
C,-2.5226135899,2.2356548198,-0.064473871
C,-2.8231406487,-2.4809176568,0.0536816799
C,-3.4933543447,-3.7555334221,0.0930759458
C,-2.8179075088,-0.0006843873,-0.0137470333
H,-4.5640326738,-3.8946166993,0.0908177991
H,-2.6563413723,3.3064421732,-0.0970826702
H,4.5421460666,1.399092337,0.0158301047
N,1.4276407981,-2.6939807468,0.0249024662
N,-1.4638743852,-2.6791359946,0.0546180654
N,1.4330307868,0.1932609184,-0.0310575876
N,-1.4526339203,0.2011589344,-0.0140181076
C,2.5143549602,2.2224782487,-0.009007287
H,2.6537885507,3.2928464823,0.0113709358
C,3.451670433,-3.7712936158,-0.0508160817
H,4.5197750708,-3.9165434036,-0.1075799659
C,-2.5311637354,-4.7189068132,0.1256409141
C,-3.4849058796,1.2805055391,-0.0440821875
H,-4.5552486167,1.4221669394,-0.04435099
C,-0.0285836257,-4.6834362505,0.0944821785
C,-3.4660676424,-1.2328577434,0.0181356921
C,3.4378393138,-1.25158468,-0.0333032156
C,-0.004094892,2.2032168535,-0.040031867
H,2.6553909552,-5.8023014165,-0.0400028153
H,-2.6513883464,-5.7910471911,0.1594835933
C,4.9324538594,-1.2595703197,-0.0546652641
C,5.6485579762,-0.9120680426,-1.205140566
C,5.676470896,-1.6178712056,1.0746223151
C,7.0414986464,-0.9149355591,-1.2357895963
C,7.0693491354,-1.6334504886,1.0661829279
C,7.753324271,-1.2786647637,-0.0947303454
C,0.0001546987,3.6982791371,-0.051202536
C,0.3312577137,4.4193475901,-1.2032157606
C,-0.326290351,4.4371186354,1.0908351541
C,0.3417518977,5.8123855927,-1.2241284975
C,-0.3281565163,5.8302554418,1.0927053228
C,0.0090001954,6.5191147592,-0.0704710049
C,-4.9615083612,-1.231097944,0.0148222048
C,-5.6875291435,-0.9681364401,-1.1510723923
C,-5.6933199454,-1.4976583157,1.1763712414
C,-7.0806901438,-0.9655822566,-1.1666757747
C,-7.0864111921,-1.505116134,1.1841722307
C,-7.7812493255,-1.2366515641,0.0066778825
F,5.0495458604,-1.9606046555,2.2074694136
F,7.7513843212,-1.9802027001,2.1628686403
F,9.0878223716,-1.2875667403,-0.113509405
F,7.6967052455,-0.5775547564,-2.3513766786
F,4.9953303157,-0.562337409,-2.3203023027
F,0.6532315825,3.769917639,-2.3291017521
F,0.6626652696,6.4726777041,-2.3417767388
F,0.0130350046,7.8538517985,-0.0793197225
F,-0.6448659342,6.5079768481,2.2011224527
F,-0.6517494008,3.8050133576,2.2258048408
F,-5.0543703226,-1.7571237072,2.3241344502
F,-7.758105397,-1.7621640717,2.3114497052
F,-9.1160065596,-1.2385841772,0.0028618475
F,-5.043528026,-0.7066763121,-2.2957135195
F,-7.7469278816,-0.7100362215,-2.2975430213
O,-0.108028693,-6.0427341462,0.1370498362
H,0.7730150807,-6.4219911308,0.2386986928
Pt,-0.0143972406,-1.2424450874,0.0098981307

Cartesian Coordination of 2pt⁻

–3364.0371422 hartree
C,-1.2605803186,-3.8458065334,-0.0002468628
C,1.2431542052,1.7768891359,0.0001538069
C,3.4853906517,1.4905085951,0.0001078923
C,2.8158098722,0.2133544871,0.0000015544
C,2.813964085,-2.2803489388,-0.0002094647
C,2.5139378995,-4.5142146675,-0.0003870657
C,1.2606055015,-3.845798795,-0.0003029946
C,-1.2431639421,1.7768814519,0.0001437064
C,-2.5216932123,2.4521664142,0.0001978357
C,-2.8139486882,-2.2803663743,-0.0001258893
C,-3.4867589534,-3.5433661228,-0.0001929114
C,-2.8158098991,0.2133370616,0.0000347882
H,-4.5588230455,-3.6831618376,-0.0001879775
H,-2.6615458555,3.5239294971,0.000282232
H,4.5568446453,1.6336871429,0.000108182
N,1.4452715703,-2.489370375,-0.0002062954
N,-1.4452548097,-2.4893793443,-0.0001456071
N,1.4454997704,0.4206473582,0.000044249
N,-1.4455010829,0.4206384316,0.0000367396
C,2.5216793255,2.4521820374,0.0001980464
H,2.6615252607,3.5239459984,0.0002820849
C,3.4867822543,-3.5433444563,-0.0003308466
H,4.5588472094,-3.6831335257,-0.0003643642
C,-2.5139085713,-4.5142302618,-0.0002695544
C,-3.4853985989,1.4904869662,0.0001216462
H,-4.5568534724,1.6336589231,0.0001324394
C,0.0000148474,-4.5686647986,-0.0003204236
C,-3.4474720108,-1.0226488636,-0.0000421011
C,3.4474796727,-1.0226274627,-0.0001141206
C,-0.0000068331,2.4235443629,0.0002024742
H,2.6200561757,-5.5889476332,-0.0004720789
H,-2.6200200804,-5.588963893,-0.0003392898
C,4.945684186,-1.0263866575,-0.0001392258
C,5.6820545369,-1.0345163446,-1.1898252819
C,5.6820931871,-1.0348531782,1.1895208492
C,7.0760922584,-1.0475060625,-1.2038673463
C,7.0761311791,-1.0478468662,1.2035138979
C,7.7756714881,-1.0546333137,-0.0001891885
C,-0.0000114711,3.9192098368,0.0003219791
C,-0.0001428099,4.6593427531,-1.1885724186
C,0.0001152688,4.6591522476,1.1893352308
C,-0.0001496527,6.0534900795,-1.2031234045
C,0.00011346,6.0532971687,1.2041087672
C,-0.0000202655,6.7531418492,0.0005487788
C,-4.9456765142,-1.026417283,-0.0000318078
C,-5.6820741187,-1.0346825122,-1.1897002717
C,-5.6820581292,-1.0347572637,1.1896458837
C,-7.0761119417,-1.0476828268,-1.203708838

C,-7.0760958633,-1.0477574427,1.203672435
C,-7.7756636267,-1.0546812891,-0.000013655
F,5.0503083681,-1.0267226507,2.3743912414
F,7.7508907698,-1.0518802419,2.3670693998
F,9.1190004111,-1.0655656706,-0.0002126826
F,7.7508138334,-1.0512128393,-2.3674460698
F,5.0502314559,-1.0260595639,-2.3746735756
F,-0.0002630144,4.0292302131,-2.3747712277
F,-0.0002760588,6.7288599964,-2.3666478774
F,-0.0000244117,8.0965587901,0.0006572594
F,0.0002357221,6.7284813848,2.3677409315
F,0.000239463,4.0288500378,2.3754333745
F,-5.050246299,-1.0264981275,2.3745014547
F,-7.7508285301,-1.0516687099,2.3672440295
F,-9.1189924859,-1.0656218654,-0.00000495
F,-5.0502781206,-1.0263465415,-2.3745633875
F,-7.7508603788,-1.0515199636,-2.3672714942
O,0.0000186742,-5.8205904111,-0.0004218405
Pt,0.0000038576,-1.0332907116,-0.0000707993

Cartesian Coordination of 2ni⁻-TBA⁺

–4100.5891076 hartree

C,1.3537173987,-2.6502832847,-2.6436196318
C,2.5919580981,-3.3423376683,-2.6066811337
C,3.4549510083,-2.572247776,-1.8548998621
C,2.7364421256,-1.3931123823,-1.4869716699
C,3.3101659893,-0.2192501051,-0.9711293648
C,2.6334101757,0.9831334672,-0.8762364788
C,3.2409292923,2.2692584569,-0.6475533683
C,2.2359667902,3.1916327191,-0.649813823
C,0.9973318845,2.4648630741,-0.8163348965
C,-0.2706671931,3.0027405941,-0.5748133556
C,-1.4076854306,2.2010172909,-0.4525372005
C,-2.6707930514,2.6348795674,0.1031181851
C,-3.4932091079,1.5498248311,0.1267625739
C,-2.7741148483,0.4599821424,-0.4873965237
C,-3.3319633649,-0.740505327,-0.8865853911
C,-2.6722439142,-1.6658788888,-1.7185090336
C,-3.3015011682,-2.7367068851,-2.4220129984
C,-2.3218397183,-3.3405380864,-3.1834374005
C,-1.1103513132,-2.658878873,-2.9045024906
C,0.1798338515,-3.1665953508,-3.3251089146
C,4.7470442774,-0.2590569277,-0.5782439184
C,5.1181832955,-0.4146805602,0.7582674392
C,6.4466860499,-0.4868269382,1.1640657695
C,7.4562014385,-0.3961667536,0.2085736836
C,7.1241686213,-0.2340257928,-1.1353343318
C,5.7834840878,-0.168884831,-1.5117291628
C,-0.3776672977,4.4631581738,-0.3112566622
C,-0.5579259994,4.9865536209,0.9730924786
C,-0.6448738033,6.3539089715,1.2190878618
C,-0.54030369,7.2493151808,0.1574194296
C,-0.3537719764,6.76696102,-1.1360404135
C,-0.2777634907,5.3928730121,-1.3530784209
C,-4.7384597183,-1.0490949195,-0.5069411462
C,-5.8334764159,-0.3763581988,-1.0593609207
C,-7.1464024188,-0.6725907652,-0.6963776914
C,-7.3944430811,-1.6753212545,0.2383047346
C,-6.3277774532,-2.375067999,0.7980190614
C,-5.0290513867,-2.0572146436,0.4164558605
C,-0.9609374588,-1.911017492,3.3008031403
C,-2.0746352652,-0.9365758394,2.9281807662
C,-3.2607811542,-1.0653118975,3.8924007818
C,-4.3724939536,-0.0623247244,3.5692419348
C,-0.4125607072,-2.7545825105,1.0109467206
C,-1.0178058114,-4.1341639391,1.2604006927
C,-1.2357750297,-4.891188117,-0.0613848263
C,0.0541810933,-5.4408147847,-0.6763653257
C,1.2629577315,-2.8728107069,2.868973484
C,2.3496484538,-3.3001106286,1.8860465344
C,3.6123965657,-3.7630312413,2.6244297828
C,4.7177796503,-4.1831035802,1.6512145654
C,0.6477286272,-0.6839130284,1.8332545119
C,1.177977181,0.1892879236,2.9646651826
C,1.5013445352,1.5918821675,2.4385846633
C,1.9950647696,2.5307267551,3.5392322857
F,4.1668509134,-0.5185629158,1.7127540335
F,6.7582656299,-0.6491232408,2.4595984523
F,8.7395337663,-0.4625174592,0.5804935521
F,8.0954442332,-0.1405254414,-2.0526144467
F,5.5010218548,-0.0066962234,-2.8110462029
F,-0.6464379075,4.1577808643,2.0329518173
F,-0.8177051547,6.8141141028,2.4681440791
F,-0.6185844964,8.5669265125,0.379697381
F,-0.2550090041,7.6272358116,-2.1591822034
F,-0.1044995268,4.9668601838,-2.6136451884
F,-5.6403157502,0.5804885934,-1.9788127797
F,-8.1712415284,-0.0050449835,-1.242826409
F,-8.649922335,-1.9650067183,0.5981518555
F,-6.5571444374,-3.3358267951,1.7066735366
F,-4.0239497572,-2.7509163347,0.9934156845
H,2.7706132784,-4.2871420791,-3.0982401227
H,4.4924993388,-2.7702439785,-1.626312057
H,4.3008195186,2.4411580883,-0.5225247984
H,2.3164185599,4.2597248978,-0.5089488367
H,-2.8916224978,3.6360478908,0.4424147732
H,-4.512139045,1.5048472323,0.4821364808
H,-4.3517971997,-2.9871601246,-2.3702935616
H,-2.3972493052,-4.1912093078,-3.8443055567
H,-1.3617768809,-2.9121993808,3.4609065611
H,-0.4690770303,-1.6051535152,4.2258808279
H,-2.4251671696,-1.1223452536,1.9110433749
H,-1.7048682876,0.0934449922,2.9482953451
H,-3.6597386599,-2.0863007794,3.8415929352
H,-2.9172440613,-0.9133144826,4.9245437238
H,0.4135536051,-2.7916480892,0.3040607234
H,-1.1569471273,-2.0883487882,0.5808545813
H,-1.9801581628,-4.0259956632,1.7680257963
H,-0.3727824227,-4.7393171234,1.9081428339
H,-1.925574219,-5.7199226263,0.1350632367
H,-1.7441661328,-4.2372211011,-0.7744952755
H,0.8007636297,-3.7412915773,3.3411000346
H,1.6813911345,-2.2545017743,3.6634930719
H,1.9865800763,-4.1102963707,1.2467179023
H,2.6181099428,-2.4761084143,1.2210131188
H,3.3674320237,-4.6004306592,3.291551741
H,3.9772388883,-2.9486207026,3.2631166803
H,1.4191315108,-0.8578041521,1.0858187582
H,-0.1803677504,-0.1971915537,1.3197291285
H,2.0857379145,-0.2420949559,3.3993709589

H,0.4421639623,0.2789422047,3.7715093916
H,2.2602088186,1.5131581269,1.6577924736
H,0.6107213982,2.0146342628,1.9654269625
H,-5.2093646918,-0.1625870631,4.2669316045
H,-4.0035788434,0.9674679066,3.626160974
H,-4.7578882802,-0.2173936741,2.5562915494
H,-0.1456984037,-5.9116266903,-1.6427949161
H,0.7917729744,-4.6556260334,-0.8644781333
H,0.5144537999,-6.1896699467,-0.0196699528
H,5.6159268775,-4.503367994,2.1877387697
H,4.3903915069,-5.0103111007,1.0122102943
H,4.9942277938,-3.3514544797,0.9947354406
H,2.224331104,3.5187106033,3.1286853974
H,1.2379925636,2.6653136191,4.320616196
H,2.905407999,2.1447904595,4.0130979328
N,1.4274879305,-1.4789731464,-1.9386448693
N,1.2590969817,1.1304586162,-1.0071529767
N,-1.4787914653,0.8700695383,-0.7912522413
N,-1.3255549175,-1.6239162942,-2.0255645257
N,0.1333482666,-2.0572651491,2.2564697575
Ni,-0.0322765367,-0.2635679101,-1.4677260764
O,0.2757854218,-4.1006417274,-4.1426763603

Cartesian Coordination of 2ni^{-}-TATA^{+}

–4663.907739 hartree

C,0.3613297225,-3.1205654403,1.1924806808
C,-0.9714310692,-2.5784494536,1.2553985253
C,-2.1018217902,-3.4006922517,1.5068180502
C,-3.1793952234,-2.558080472,1.6421839235
C,-2.6898627792,-1.2313062464,1.4352274574
C,-3.4888673523,-0.081421014,1.4019613741
C,-3.0254825805,1.1606124983,1.0157669592
C,-3.8705262798,2.2941909,0.7409391438
C,-3.079501231,3.2531575438,0.1832499745
C,-1.7344170318,2.7274337235,0.1657510697
C,-0.616183905,3.4626330159,-0.2299108133
C,0.6822623434,3.0546165239,0.0617228018
C,1.84105238,3.9124259673,-0.0467362619
C,2.8826079552,3.2398904343,0.512388809
C,2.3907549209,1.9364847444,0.8907201855
C,3.1719871251,0.9101967981,1.3859793991
C,2.7169442453,-0.4156321671,1.513974314
C,3.5554416895,-1.5373760083,1.8017837501
C,2.7749763484,-2.6626754635,1.6625487714
C,1.4712748012,-2.213911966,1.3277339118
C,-4.936655358,-0.2254059112,1.7295033398
C,-5.4334502201,0.1416453039,2.9835399113
C,-6.780961979,0.0122758249,3.3148101525
C,-7.6730099513,-0.5003334565,2.3759133535
C,-7.2105835121,-0.877138904,1.1171049628
C,-5.8597795043,-0.7347141843,0.8137100456
C,-0.8414863459,4.7898825906,-0.8638832669
C,-0.7141001719,5.9918367066,-0.1590447927
C,-0.9426554634,7.2308910253,-0.7541161928
C,-1.3220113019,7.2918482603,-2.093241134
C,-1.4663431423,6.1151236052,-2.8245181532
C,-1.224734829,4.8928938756,-2.204201578
C,4.5856817687,1.1902088144,1.7655672338
C,4.9906399373,1.1117212906,3.1051494137
C,6.307648107,1.3313879191,3.5015483135
C,7.2725225788,1.6467826812,2.5471993023
C,6.90592179,1.7417401529,1.2079832395
C,5.5824764928,1.5149836721,0.8410828807
C,0.6713201541,-2.872517847,-1.9743264001
C,-0.6726677159,-3.3045378746,-2.0149963786
C,-0.9836441023,-4.6718786627,-1.7608333063
C,-2.3321352015,-5.0517589808,-1.6846994014
C,-3.3245544125,-4.0971703712,-1.8889435908
C,-3.0434034075,-2.770959488,-2.2026586337
C,-1.7070984801,-2.3601443099,-2.2826794926
C,0.9762543179,-1.5053344721,-2.1548675736
C,-0.0639023753,-0.5854010357,-2.4831959574
C,0.2492979615,0.7672710022,-2.6552601388
C,1.5701845474,1.1797027013,-2.5067274484
C,2.6051946577,0.303031237,-2.1986774265
C,2.3201384268,-1.0552159789,-2.0052057635
C,1.7011598532,-3.800655555,-1.7126401699
C,3.0442445086,-3.3399389141,-1.5845875102
C,4.0624119748,-4.2759548848,-1.3535484842
C,3.7283658064,-5.6177098303,-1.1954365733
C,2.4194883406,-6.0830325716,-1.2622435267
C,1.3807292131,-5.17880276,-1.5274980454
C,-0.2707762669,-6.9920301572,-1.3716484005
C,-0.4623162775,-7.3181071286,0.1127768004
C,-0.7227183859,-8.8105971082,0.32589879
C,4.6613084442,-1.500414633,-1.3916407203
C,5.5595216116,-1.4606024921,-2.6302918816
C,6.9767887923,-1.0034065132,-2.2729053614
C,-2.3707266453,-0.2069115633,-3.2775397016
C,-3.1568495627,0.6700974631,-2.3062351442
C,-4.2522955254,1.4591986998,-3.0231701324
F,-4.6023577667,0.6327120592,3.9146179824
F,-7.224769399,0.3741571917,4.5267269751
F,-8.9699616548,-0.6305022607,2.6809543314
F,-8.0676127503,-1.3690202302,0.2083092715
F,-5.4554925473,-1.1037434707,-0.4182507804
F,-0.3702952742,5.9744470027,1.138136893
F,-0.8102523236,8.3633844587,-0.0484417999
F,-1.5475107764,8.4761357941,-2.6751476239
F,-1.8303501008,6.1692049976,-4.1151640218
F,-1.3689714043,3.7770559021,-2.9490226703
F,4.0939986634,0.8251886163,4.0591682776
F,6.6535192068,1.2473758729,4.793392625
F,8.5416588491,1.8602948038,2.9149804963
F,7.8277351567,2.0400078709,0.2783200812
F,5.2886335338,1.6096592198,-0.4752701934
H,-2.0579183003,-4.474975718,1.597322742
H,-4.2065838689,-2.8195645748,1.8524087932
H,-4.9344284803,2.3348004486,0.9260041518
H,-3.3663281434,4.2368577836,-0.1591850555
H,1.8403197818,4.9147742939,-0.4493973665
H,3.895749657,3.5895897766,0.6429621423
H,4.6044036651,-1.4852226049,2.0566496846
H,3.0426267171,-3.7012044298,1.7861709857
H,-2.6227474845,-6.0585672376,-1.4257334896
H,-4.3629565866,-4.3959729731,-1.78518145
H,-3.8556230165,-2.0696556324,-2.321681425
H,-0.5142875758,1.505787491,-2.8431917699
H,1.8000943199,2.2346479533,-2.609421945
H,3.6043559255,0.6949411747,-2.0931994505

H,5.0988902839,-3.981460695,-1.2881856288
H,4.5230129713,-6.3303381398,-0.9962552465
H,2.2285119429,-7.1274771163,-1.0697908946
H,-1.1648860661,-7.2317911529,-1.9493944655
H,0.5254686284,-7.5980201752,-1.80634644
H,-1.2918794431,-6.7218611902,0.505421804
H,0.418552901,-6.9852872683,0.6686031219
H,-1.620982996,-9.14360962,-0.2082760841
H,-0.8662242427,-9.0338737088,1.3872368041
H,0.1181021404,-9.418284371,-0.0299966206
H,5.0830243129,-2.1400329592,-0.6161429847
H,4.5762009226,-0.5159183939,-0.9410840497
H,5.1188000065,-0.7848228893,-3.3722373788
H,5.5855255691,-2.4543664239,-3.0923462318
H,6.972258071,0.0008124497,-1.8367158018
H,7.6181363016,-0.9796204115,-3.1588777487
H,7.439537108,-1.6815120638,-1.5458728659
H,-1.852652179,0.4071611839,-4.0187331392
H,-3.0412482151,-0.8656851807,-3.8348923085
H,-3.5883375949,0.0465129678,-1.5211022195
H,-2.4717888329,1.3548486289,-1.8071955251
H,-3.8315860479,2.1180620986,-3.7916976463
H,-4.7953394537,2.085657314,-2.3103463405
H,-4.9764137197,0.7937559777,-3.5084816634
N,-1.3240100785,-1.2530588434,1.2062156144
N,-1.711280285,1.4522908148,0.6726010937
N,1.0342269173,1.8440861222,0.6090374857
N,1.4302176082,-0.8413975085,1.2408658403
N,0.05570376,-5.5789202737,-1.6085400951
N,-1.3572251909,-1.0659636691,-2.6390088952
N,3.3016310641,-1.9799604703,-1.6722489847
Ni,-0.1413337225,0.2973550328,0.9206685256
O,0.5433551392,-4.3596344869,1.1002016385

Cartesian Coordination of 2pd⁻-TBA⁺

–4058.0146375 hartree

C,7.2897249236,12.7004874221,-0.7766434209
C,6.2000447668,12.076242751,-0.1732694117
F,7.112931413,13.4406182174,-1.877027771
F,4.9704330662,12.2194364906,-0.6928320562
C,8.5632080366,12.5505928149,-0.2310857181
F,9.6102555899,13.1495813258,-0.8133598753
C,7.9795909004,11.4283849235,7.607534556
C,7.819848415,11.5125042493,6.1644512439
C,7.581944832,12.7388062058,5.4860733629
C,7.5039221555,12.4455888012,4.1398238633
C,7.7145037785,11.0362856022,4.0087284477
C,7.8357057193,10.3352364695,2.7911021485
C,8.0959169728,8.9821447892,2.6508912589
C,8.3181382222,8.2926286803,1.4008993746
C,8.3027155889,6.8215493767,3.1223486586
C,8.2160746037,5.5979313908,3.7987066838
C,7.9650253916,5.455841938,5.1694282368
C,7.7365688333,4.1937758302,5.8432530504
C,7.5048615611,4.4721703454,7.1564342103
C,7.6195327371,5.903401407,7.3203487495
C,7.5379547468,6.591237295,8.5218444163
C,7.7249402673,7.9799535699,8.6953386778
C,7.8140475315,8.6549192254,9.9531907537
C,7.9947440773,9.9938410909,9.6796340678
C,7.9951263786,10.1324639582,8.2657044407
C,7.6625130852,11.1382745182,1.5445616153
C,6.402990216,11.3080345511,0.9684264803
C,8.3198786331,4.3560581586,2.9810483582
C,7.2623505155,2.481900116,1.8188122124
C,7.186309403,3.6357661075,2.5934987539
C,7.2213400659,5.812615402,9.7528760045
C,7.8182785171,4.1913709188,11.481198133
C,6.579925479,4.3642882266,12.0949082562
C,5.6602345946,5.2598200509,11.5530245543
C,5.9918537552,5.9663293217,10.4019632053
C,3.0608066279,7.9910125332,6.2697849641
C,3.9147409212,6.7988283411,6.6897590886
C,3.0580034656,5.7642267109,7.4335765983
C,3.8280420637,4.4704297367,7.7110043787
C,4.7368518807,8.6720662239,4.5460144241
C,4.0047070559,8.0333371047,3.3718846567
C,5.0026791447,7.3728875851,2.4146099326
C,4.3175475386,6.7054572124,1.2221555284
C,2.8261106711,10.1644761171,5.133879257
C,3.4272455315,11.3619065763,4.4049253126
C,2.326019827,12.3307080299,3.9528798459
C,2.9016800079,13.513571298,3.1680038815
C,4.7131258813,9.8041451221,6.7454044148
C,3.9740820882,10.1912438411,8.0295672142
C,4.6543205417,11.3492587565,8.7807175037
C,4.6102075623,12.685338386,8.0339623475
F,5.3232603542,10.716206618,1.5256937983
F,6.1496622345,1.8189672747,1.4666882081
F,5.9619936999,4.0562390752,2.969577913
F,6.2739654439,3.6740615474,13.199590561
F,4.4624967273,5.4254400756,12.1349660863
F,5.0679778443,6.8135377477,9.9029019843
H,7.5097079728,13.696692607,5.9795436327
H,7.351893859,13.1348473045,3.3212107266
H,7.7507621503,3.2238090072,5.3679897816
H,7.2958909302,3.7680794859,7.9483507616
H,7.766380315,8.1802864474,10.9228941639
H,2.5106086257,8.3929342941,7.1210184743
H,4.3615902236,6.3215737846,5.8119425052
H,2.1628123032,5.5342634794,6.8394178046
H,4.1523631498,3.9974139427,6.7779659949
H,5.3288034656,9.5292924022,4.2281688643
H,3.4235154732,8.783012432,2.8230899147
H,5.7085124263,8.1263775138,2.0590155614
H,3.6300815586,5.9156429404,1.5466162115
H,2.2277610651,10.4921037995,5.9848068985
H,4.1294129711,11.9012979131,5.0468073963
H,1.7769658769,12.6985383016,4.8297057115
H,3.6194552507,14.0752104145,3.7758959437
H,5.5129136283,9.0971031835,6.9556290083
H,2.3263080145,7.6880631019,5.5224351735
H,4.7404474312,7.1119432363,7.3346105347
H,2.7055674134,6.1972418318,8.3779075762
H,3.20857536,3.7537727546,8.2590173954
H,4.725402129,4.6671516474,8.303441975
H,5.435115079,7.9715935284,5.0005355025
H,3.2992556295,7.2703697716,3.7199103457
H,5.5896814047,6.6308092532,2.9633378721
H,5.0585227891,6.2476858627,0.5597114906

H,3.7450639752,7.4317480161,0.6331489421
H,2.1662012371,9.6072023222,4.4667019937
H,3.9888006854,11.0353511808,3.5263720642
H,1.5972891383,11.7951550068,3.3296317278
H,2.1118515648,14.2033324453,2.8556338479
H,3.4227736591,13.1686677459,2.2681270183
H,5.1810667802,10.6607999507,6.2669092265
H,2.9434479407,10.4954615362,7.8140691914
H,3.9156723535,9.3190380695,8.6894089313
H,5.6894635092,11.0869380663,9.007342752
H,4.1470848215,11.4550194381,9.7467847225
H,5.1764649386,12.6519844353,7.0982406502
H,5.0656055357,13.4737117682,8.6384227166
H,3.5776769722,12.9789518292,7.8033613897
N,7.8829467201,10.4836312541,5.2643260934
N,8.1193010316,8.0571395624,3.6818359065
N,7.8848662423,6.4726733406,6.0837595696
N,7.8486162301,8.9034485331,7.6764426194
N,3.8322618912,9.1584091684,5.672191513
O,8.0228516472,12.4739779669,8.2885864122
Pd,7.9632556677,8.4770295328,5.6786572355
C,8.4566815234,6.9674215618,1.6891081342
C,8.7337194997,11.7789855992,0.9175365396
C,9.5538607877,3.8611015294,2.547715852
C,9.6609305459,2.71141864,1.7674427528
C,8.5085672885,2.0190131229,1.4020831028
C,8.1241755993,4.9135260968,10.3288414203
F,9.9704225303,11.6591093312,1.4197412317
F,10.6850354471,4.5003290956,2.8830295274
F,10.8600352042,2.2649469954,1.3682750955
F,8.5981399155,0.9119932239,0.6549514283
F,9.3344099512,4.7309494693,9.7793138775
F,8.706651784,3.3359787998,12.0050690017
H,8.3631047791,8.7658344392,0.4301840012
H,8.6313326531,6.156604846,0.9969090302
H,8.0991015666,10.8205783841,10.3664838385

Cartesian Coordination of 2pd⁻-TATA⁺

–4621.3334537 hartree

C,-0.4796513125,4.9877098,-0.3049318451
F,-1.798741005,4.7737092339,-0.4070944449
F,-0.4498294306,5.9666396049,-2.4604487452
F,-0.4804594012,4.0321602901,1.8347347976
C,4.8305096421,-0.49032348,6.3641630574
C,3.6375476446,-1.2373838364,3.8035936206
C,2.7191484431,-2.0496135201,4.4834960835
C,2.5460703972,-3.3988374901,4.1829493014
C,3.2950021394,-3.9799974908,3.1620700676
C,4.2067119119,-3.202559722,2.4534460419
C,4.3609391095,-1.8586329437,2.7811558406
C,8.6504220872,-0.6456978689,3.3522165902
C,8.4500385989,-2.1575552655,3.212971234
F,1.9611102496,-1.5290565386,5.4598245317
F,1.65953715,-4.1408933938,4.8609920914
F,3.1382965944,-5.2753661525,2.8635680444
F,4.9370574329,-3.7520064574,1.4698245532
F,5.2634346811,-1.1535929587,2.0627760485
H,5.6479662683,-0.23297255,8.3856338243
H,4.8115343088,-1.559925931,6.2137639569
H,2.5755258359,-0.1394180109,1.5715131226
H,8.5085334215,-2.6563595436,4.1877987785
H,7.5664556614,-0.511201421,5.2385659132
H,7.4739931109,-2.3889695293,2.7750771109
C,0.2053373104,5.5978877737,-1.3531798024
C,1.576403121,5.8208799796,-1.2458277149
C,2.2426181196,5.429135344,-0.0888075812
F,2.2421765153,6.4033566729,-2.2548247388
H,9.215908404,-2.5981430372,2.5679461994
C,8.8515242954,7.335584104,6.7467918725
C,8.8592197013,5.9758476551,6.4153760373
C,9.5509265994,3.6825879019,6.863703356
C,10.2889388247,2.7459931113,7.6013187486
C,10.1641199167,1.3925085005,7.3058244987
C,9.335692278,0.9154483929,6.2958191492
C,10.500186874,5.5319087481,8.1905320903
C,9.808140468,5.601594444,9.5555200881
C,10.7342536344,6.1802360922,10.6274378899
H,9.3597796173,7.7114757256,7.6220303354
H,10.9098129791,3.0398660158,8.4337081768
H,10.7311774796,0.6782867507,7.8949367311
H,9.2947155792,-0.1467971796,6.1082196842
H,10.8736165518,6.5128111298,7.8921067105
H,9.4765706692,4.5983264455,9.8385649094
H,11.6373685121,5.5701393999,10.7461485463
H,9.6464630769,-0.4313250244,3.7562950285
H,11.3774976328,4.8852790519,8.2297228983
H,8.901521934,6.2096223368,9.4622826337
H,10.2318799063,6.2196917768,11.5983357005
H,11.0515277088,7.1988084461,10.3747026969
N,9.6161497139,5.0453022127,7.1223288903
C,5.6127687645,6.2334252141,8.0117531212
C,5.8653786315,5.0391150643,8.651622855
C,5.3905407194,3.9969509304,7.7928798978
C,5.4306254865,2.613912478,8.0757014066
C,5.1025445909,1.594290867,7.1962816577
C,5.2530755412,0.1837020885,7.4699748318
C,4.3824313219,0.4977218634,5.4030382279
C,3.8069620257,0.2006310255,4.1594458529
C,3.2936458973,1.1561127139,3.2697107725
C,2.6866929903,0.8499836837,1.9901314709
C,2.2799165307,2.0302786514,1.444053278
C,2.6168585068,3.0747219096,2.3831972472
C,2.3219275588,4.4182175808,2.2211425837
C,2.6524678602,5.4558236808,3.115237118
C,2.286129962,6.8313107972,2.9630859737
C,2.8621536024,7.5170447558,4.010950367
C,3.5719053974,6.5626560943,4.7895908296
C,4.4070105575,6.9375907063,5.9173451963
C,4.9618577628,5.9180424695,6.7882951983
C,5.7322830077,2.2165754622,9.4841628813
C,7.0066439217,2.2710300685,10.0449184268
C,7.2633281704,1.9187854043,11.3667294543
C,6.2165241313,1.4897331218,12.1771371821
C,4.9266866346,1.422737111,11.6544406792
C,4.7004036484,1.7871161475,10.3288375413
C,1.5894757332,4.8161144735,0.98253836
C,0.2142761899,4.6086071823,0.8428131232
C,8.1462998106,8.2290011431,5.9429123502
C,8.5971639466,1.8271835338,5.5247369743
C,7.1747303489,2.3156602851,3.6141536133

C,6.4867016014,1.8965114112,2.4719284814
C,5.8818297325,2.847244978,1.654927212
C,5.9265601049,4.2095766348,1.9221187602
C,6.6009119756,4.6625379419,3.0635285876
C,7.3734338232,6.4696031944,4.4924026449
C,7.4413726325,7.8317265345,4.8160175504
C,8.0826521518,5.5326903043,5.3039299491
C,8.710405634,3.2205466671,5.8079172052
C,7.2597017168,3.711085157,3.8992437066
C,8.0070033783,4.1545177075,5.0100718292
C,7.587443554,-0.0254284858,4.2625623063
C,5.8835191188,6.9652711592,2.5909337872
C,6.6947439784,7.5155487011,1.4169906124
C,5.8589319389,8.4934842488,0.5860377083
F,8.0495506971,2.6694190935,9.2958340785
F,8.5129010211,1.9867717204,11.8584603729
F,6.4477852014,1.1432287455,13.449448358
F,3.9150418634,1.0126727756,12.4321144633
F,3.442126246,1.722451452,9.8714859001
F,3.5727958508,5.6506927416,-0.0302717499
H,5.815462514,7.240969676,8.341975151
H,6.3291786704,4.8942742588,9.6165356001
H,1.7806284918,2.180690484,0.4975901578
H,1.6688395901,7.2306540948,2.1709482077
H,2.8247609383,8.5725244865,4.2363928942
H,8.1370059848,9.2795147564,6.2140339057
H,6.3986650082,0.8550049729,2.2112411054
H,5.3359251039,2.5067180156,0.7814742678
H,5.4153937509,4.8860905693,1.2574640506
H,6.9128578923,8.5775925804,4.2447482186
H,6.5919381608,-0.1647232212,3.8496685611
H,5.5368535936,7.7655944786,3.2401684297
H,7.594883075,8.0118740926,1.7991727517
H,4.9717582003,8.0002844575,0.1751817818
H,8.6043717957,-0.1660356998,2.368039136
H,4.976635045,6.4724707563,2.2483200387
H,7.0376968202,6.6843744166,0.7898593372
H,6.438755649,8.8954013222,-0.2502539512
H,5.5181363769,9.3380367858,1.195732132
N,3.2464414284,2.5072023536,3.4790584853
N,4.5923123419,1.7502494219,5.9171866504
N,4.8503172101,4.5590503988,6.6527715599
N,3.4239362097,5.3133299563,4.2526482478
N,7.7778052174,1.4152656379,4.486130082
N,6.6392392809,6.0045012957,3.411999283
O,4.6300943594,8.1477010652,6.1449010737
Pd,4.0353048561,3.5319918023,5.0708399963

Cartesian Coordination of 2ni-TATAB

–5990.0057217 hartree

C,-0.539636553,2.9741972857,1.3264597985
C,0.7845364376,2.5583028873,1.2588280808
C,1.8986232815,3.4443583424,1.4237597765
C,3.0054842536,2.6594906781,1.520607074
C,2.5656967993,1.2982520701,1.383402195
C,3.411575992,0.1910410921,1.4110682426
C,2.9708930556,-1.087829685,1.1070577801
C,3.8539584648,-2.200939305,0.8689234296
C,3.0969033548,-3.1974689539,0.3406229944
C,1.7395432686,-2.7156342488,0.3058977767
C,0.6545543543,-3.4859522355,-0.1001306689
C,-0.6568296079,-3.105807319,0.1518097314
C,-1.791652503,-3.9817744226,-0.0061849223
C,-2.8564129052,-3.3459665395,0.5426054338
C,-2.3973022745,-2.0448202551,0.9634484184
C,-3.2258202401,-1.0469424874,1.4545148772
C,-2.7952344844,0.2722096144,1.6152219469
C,-3.6805281372,1.3803526594,1.8734761677
C,-2.9445001365,2.5171731948,1.738580158
C,-1.6050567446,2.0972677982,1.4545808798
C,4.8565965749,0.3879863947,1.707049213
C,5.4177995343,-0.1194334835,2.882911644
C,6.7687024423,0.0331738614,3.182674932
C,7.5960701905,0.7165924338,2.2953332032
C,7.0680504603,1.2346790434,1.1156450389
C,5.7160585184,1.0614312641,0.8355145455
C,0.9364163638,-4.7900721419,-0.7572763733
C,0.7003101336,-6.0154738376,-0.1278299109
C,0.9868108894,-7.2297016527,-0.7463399735
C,1.5434167317,-7.2372797453,-2.0228403331
C,1.8028406344,-6.0333734038,-2.6730028211
C,1.4980650346,-4.8346446631,-2.0365482953
C,-4.6554466527,-1.3470313275,1.7429511671
C,-5.1509836304,-1.245779037,3.0479966161
C,-6.4937695156,-1.4551064205,3.3504297747
C,-7.3865707841,-1.783011294,2.3329384481
C,-6.9254216058,-1.9001344061,1.0250005643
C,-5.5783602447,-1.6899231286,0.7516032311
C,0.7406410544,3.3077203998,-2.0177264912
C,0.9885244301,4.6668656442,-1.7391063338
C,2.3227805303,5.1067457712,-1.6969841375
C,3.3364521943,4.1801892197,-1.9582946817
C,3.1007625584,2.8417174755,-2.2649623844
C,1.7705030562,2.3831763642,-2.2988111892
C,-0.9324204947,1.3670688418,-2.133572702
C,0.1440267993,0.5088855603,-2.4525927895
C,-0.1233411965,-0.861433097,-2.6133200331
C,-1.4399176562,-1.3002776899,-2.4693001583
C,-2.5116249906,-0.4623720263,-2.1612942887
C,-2.258344718,0.9081754102,-1.9735525392
C,-1.7663365367,3.7456778053,-1.6463808312
C,-3.0704747879,3.2201683074,-1.5168458512
C,-4.1201349005,4.11985673,-1.2443996902
C,-3.8142006497,5.4664490408,-1.0560679518
C,-2.5191707038,5.9850490277,-1.127098625
C,-1.4664917171,5.1086330597,-1.4397159441
C,0.1606145199,6.9655587833,-1.3923921178
C,0.4106395138,7.4310353346,0.045336088
C,0.6838583149,8.9328712123,0.1290176735
C,-4.6068991448,1.3095055721,-1.3578111889
C,-5.5128071805,1.2311361502,-2.5881040083
C,-6.8987506618,0.6895120883,-2.2302360589
C,2.4659719406,0.2054748454,-3.2179644497
C,3.2683611856,-0.6151797825,-2.2105834667
C,4.2795634627,-1.5380968144,-2.8888775725
F,4.6495047179,-0.7804725914,3.757250406
F,7.2735758513,-0.4639693647,4.3163443483
F,8.892133706,0.8734073223,2.5733895483
F,7.8614884629,1.8852135994,0.2574923387
F,5.2482040213,1.5663794113,-0.3178402107

F,0.1842024289,-6.0455470741,1.1075318627
F,0.7461032573,-8.3853604575,-0.1185644953
F,1.8269785612,-8.395518481,-2.6221354893
F,2.3368218743,-6.0365002356,-3.8986534329
F,1.7650243966,-3.6936975636,-2.6951486089
F,-4.3253435243,-0.9235609812,4.0502511537
F,-6.9303377207,-1.3477413379,4.6090578288
F,-8.676533104,-1.9824467788,2.6098134799
F,-7.7755413724,-2.1961292902,0.0347728008
F,-5.1901505348,-1.8073528994,-0.5329806978
H,1.83857115,4.5186919045,1.5036695321
H,4.0260206589,2.9714326538,1.6765404702
H,4.9190676552,-2.1942105104,1.0431603423
H,3.4124648216,-4.179539824,0.0229055237
H,-1.7607895731,-4.9680504753,-0.4424732778
H,-3.8660965865,-3.7122507042,0.6403563761
H,-4.7371767513,1.2939586366,2.075010225
H,-3.2549537216,3.5476557678,1.806739915
H,2.5979499076,6.1224684709,-1.4454786363
H,4.3670183068,4.5196022148,-1.9063736193
H,3.9434371147,2.1846319268,-2.4252818746
H,0.6492143485,-1.5885083648,-2.8131534748
H,-1.6411436721,-2.3609114767,-2.5837957135
H,-3.4960381897,-0.8957111438,-2.0678246674
H,-5.1532363615,3.807278643,-1.1808656514
H,-4.6279713068,6.1508009134,-0.8331587734
H,-2.3681471269,7.035640411,-0.918902264
H,1.0211982636,7.1972953704,-2.0231080435
H,-0.6800498563,7.5089000737,-1.828091358
H,1.2627074659,6.871996222,0.4521275311
H,-0.4602347168,7.1723876626,0.6598531901
H,1.5650320682,9.208324043,-0.4608073435
H,0.8611700991,9.2477556441,1.1610117169
H,-0.1646707545,9.5098344176,-0.2547520067
H,-5.0612032258,1.9292739357,-0.5832434623
H,-4.4938481654,0.3251290138,-0.912413184
H,-5.0359350029,0.5910780284,-3.338891019
H,-5.5960517017,2.2272211446,-3.0376780116
H,-6.8355283194,-0.3143679978,-1.7992543828
H,-7.5440186258,0.6337551224,-3.1115537744
H,-7.3967011306,1.3344814101,-1.4965825787
H,1.9719290842,-0.4551314125,-3.935423888
H,3.1266626121,0.8488641255,-3.8047609243
H,3.7751735874,0.0618479686,-1.519291381
H,2.573520265,-1.2041089581,-1.6115854804
H,3.7783415372,-2.2724348997,-3.5278092133
H,4.8563562994,-2.0912267107,-2.142406829
H,4.9858932638,-0.9761276027,-3.5107384814
N,1.1977082201,1.2428954069,1.2104188917
N,1.6674178464,-1.4288080184,0.7912545443
N,-1.0432248211,-1.9105587701,0.7218081564
N,-1.5132640247,0.7207822941,1.3857698722
N,-0.1125414977,5.530115485,-1.4922417546
N,1.4343644558,1.0541253238,-2.6113936257
N,-3.2698482285,1.8335913762,-1.6357176292
Ni,0.0734502402,-0.3402222184,1.0084719274
B,-0.6570563673,2.8110930402,-1.9578826814
O,-0.833407793,4.3041956277,1.4012186939
H,-0.3062398548,4.7842262407,0.7448945473

5. Solution-state behaviors of ion pairs

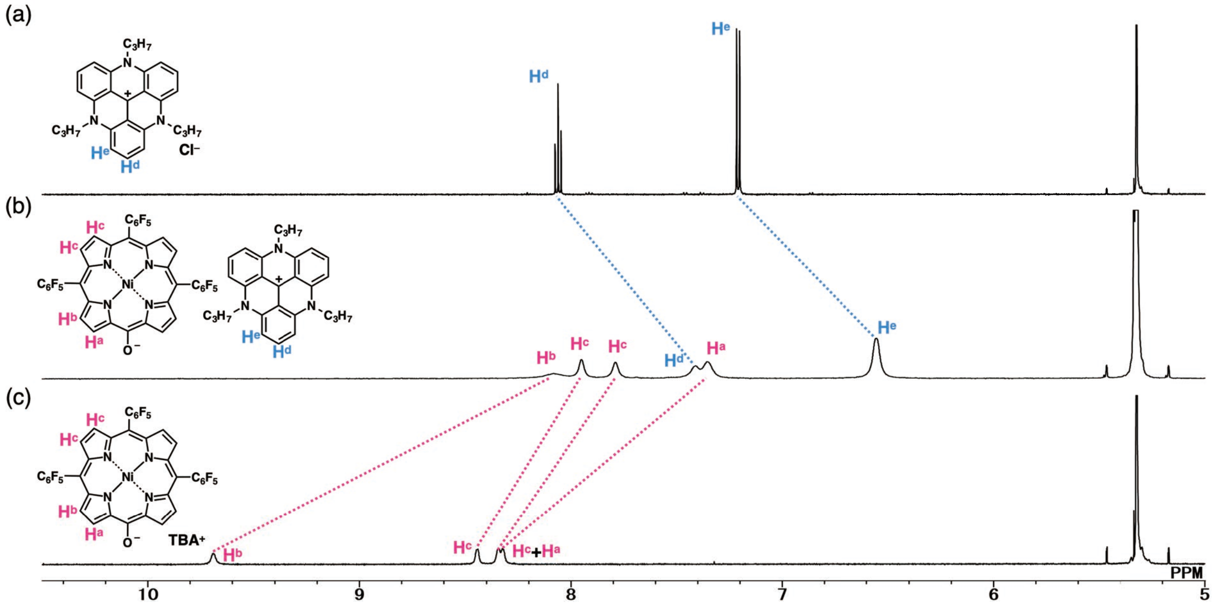

Fig. S87 ^{1}H NMR spectra of (a) TATA$^+$-Cl$^-$,[S4] (b) **2ni**$^-$-TATA$^+$, and (c) **2ni**$^-$-TBA$^+$[S5b] in CD_2Cl_2 at 20 °C (1.0 × 10^{-3} M). According to the optimized structure of **2ni**$^-$-TATA$^+$ (Fig. S44b), π-electronic ion pair exists as a stacking structure in solution state. Considering of the aromatic property of **2ni**$^-$ and TATA$^+$ in NICS calculations and ACID plots (Fig. S67–69), the upfield shifts of **2ni**$^-$ suggested the shielding effect of stacked π-systems and those of TATA$^+$ signals indicated the shielding effect of stacked π-systems and that from electron-rich anionic **2ni**$^-$.

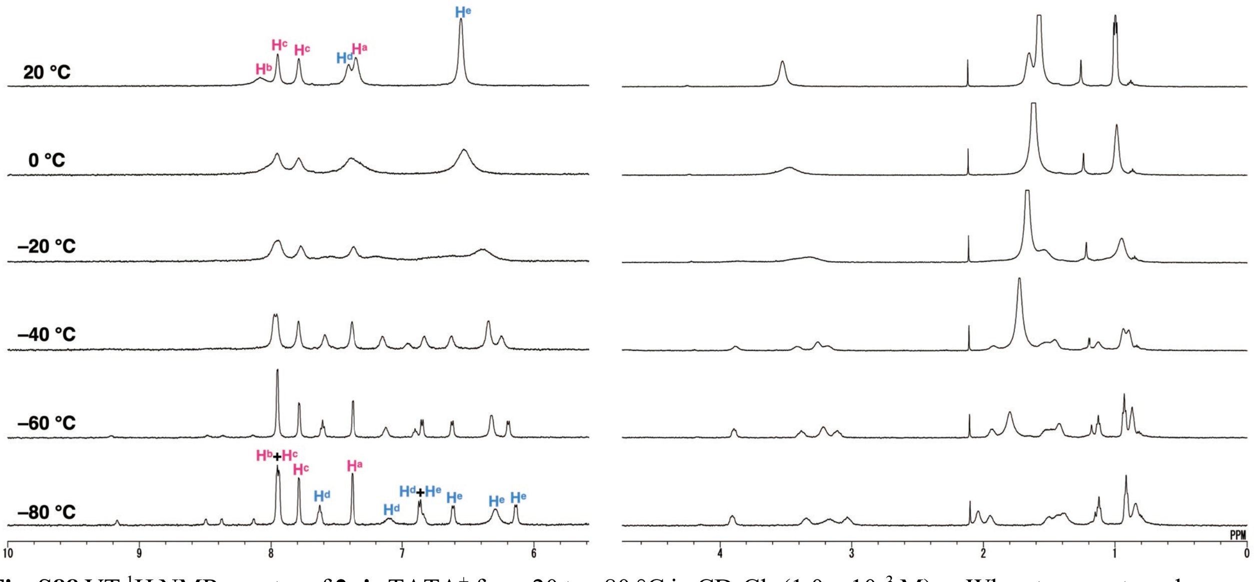

Fig. S88 VT ^{1}H NMR spectra of **2ni**$^-$-TATA$^+$ from 20 to –80 °C in CD_2Cl_2 (1.0 × 10^{-3} M). When temperature decreased to –80 °C, one signal of **2ni**$^-$ shifted upfield from 8.08 to 7.94 ppm (H^b), two signals of **2ni**$^-$ were almost no difference of chemical shift (H^c), and one signal of **2ni**$^-$ shifted downfield from 7.35 to 7.38 ppm (H^a), suggesting the tightly bound ion pair at low temperature, although a small amount of **2ni** was observed probably due to the basic property of the anionic oxygen site of **2ni**$^-$. The upfield shift of H^b indicated that H^b was located inside the current ring of TATA$^+$, whereas downfield shifts of H^a upon cooling suggested the location outside the current ring of TATA$^+$. Furthermore, signals of TATA$^+$ appeared at 7.63 (1H), 7.10 (1H), 6.87 (1H), 6.84 (1H), 6.61 (1H), 6.29 (2H), and 6.14 (1H) ppm at –80 °C (the signal of 1H would be overlapped with other signals). Split signals of TATA$^+$ suggested the slow rotation of TATA$^+$ at –80 °C due to tight ion-pairing. ^{1}H–^{1}H COSY of **2ni**$^-$-TATA$^+$ in CD_2Cl_2 at –80 °C (1.0 × 10^{-3} M) showed the correlation between signals (ppm) at 8.08–7.79, 7.94–7.38, 7.63–6.87/6.62, and 6.84–6.14, suggesting that the corresponding protons were located at the vicinal positions (Fig. S89a). In contrast to **2pd**$^-$-TATA$^+$ (Fig. S92b), the NOE correlation between 7.94 ppm of **2ni**$^-$ and 2.04 ppm of TATA$^+$ was observed (Fig. S89b), suggesting the more tightly bound stacking ion pair.

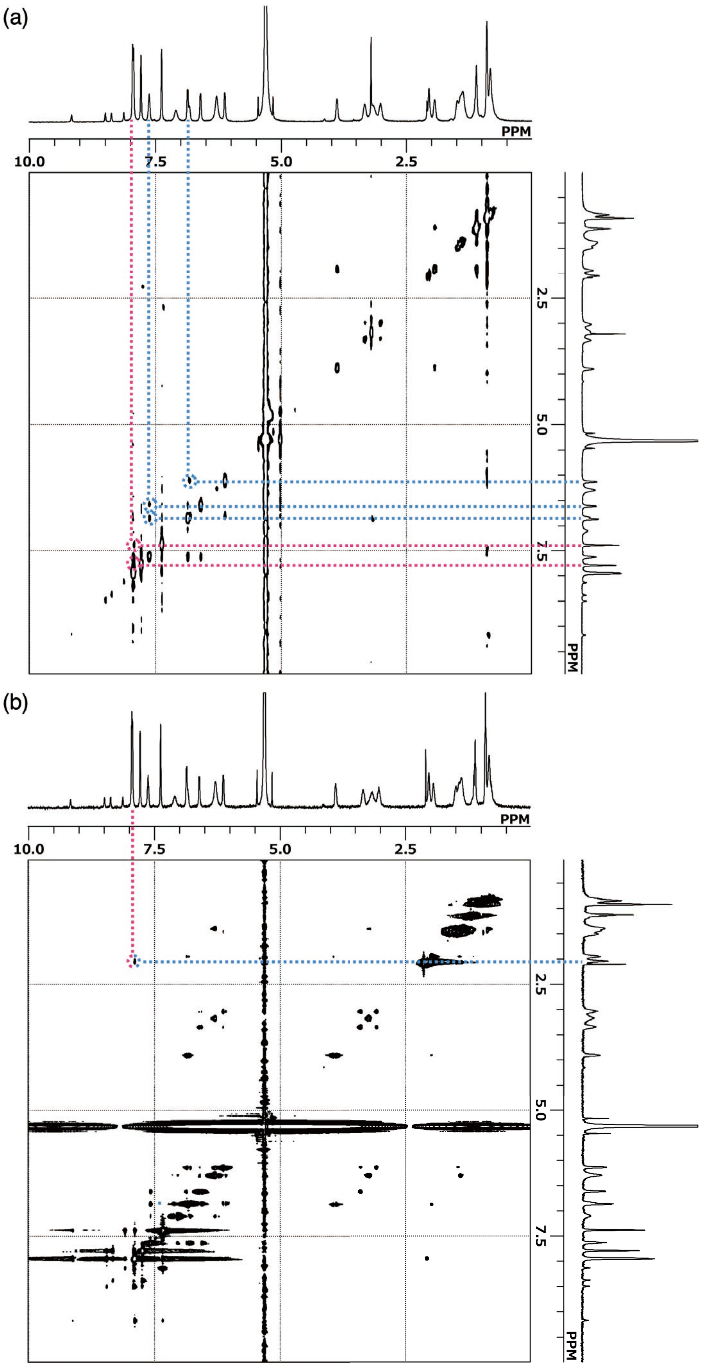

Fig. S89 (a) ^{1}H–^{1}H COSY and (b) ^{1}H–^{1}H NOESY spectra of **2ni**$^{-}$-TATA^{+} at –80 °C in CD_2Cl_2 (1.0×10^{-3} M).

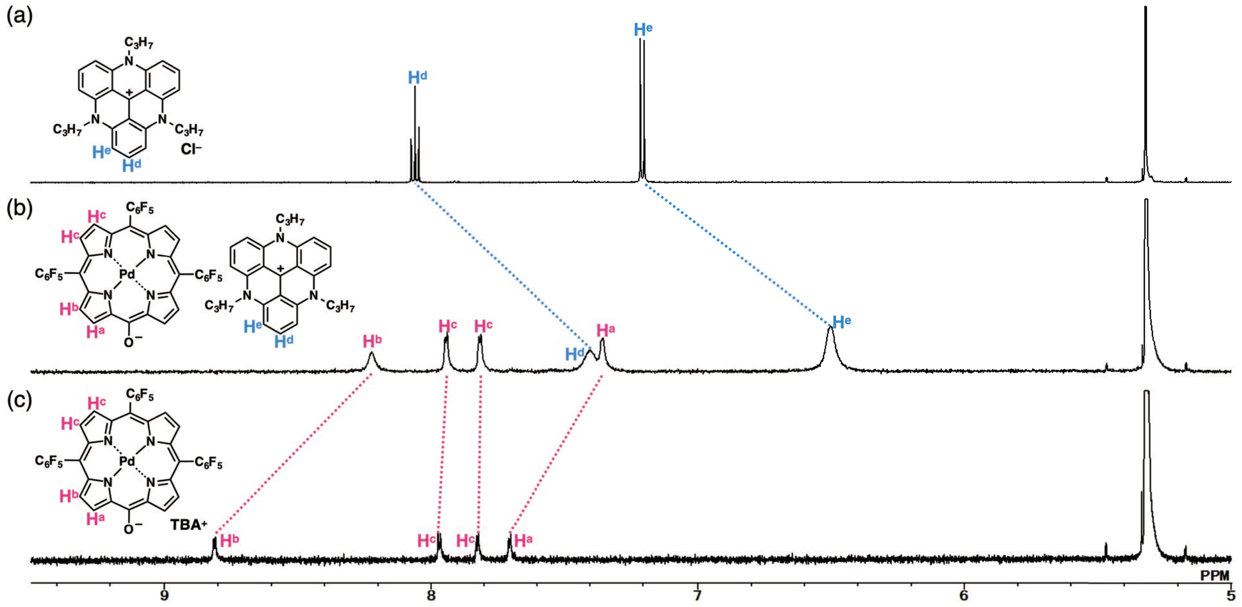

Fig. S90 ^{1}H NMR spectra of (a) TATA$^+$-Cl$^-$,[S4] (b) **2pd**$^-$-TATA$^+$, and (c) **2pd**$^-$-TBA$^+$ in CD_2Cl_2 at 20 °C (1.0×10^{-3} M). According to the optimized structure of **2pd**$^-$-TATA$^+$ (Fig. S44d), π-electronic ion pair exists as a stacking structure in solution state. As suggested by the aromatic properties of **2pd**$^-$ and TATA$^+$ in NICS calculations and ACID plots (Fig. S67–69), the upfield shifts of **2pd**$^-$ suggested the shielding effect of stacked π-systems and those of TATA$^+$ signals indicated the shielding effect of stacked π-systems and that from electron-rich anionic **2pd**$^-$.

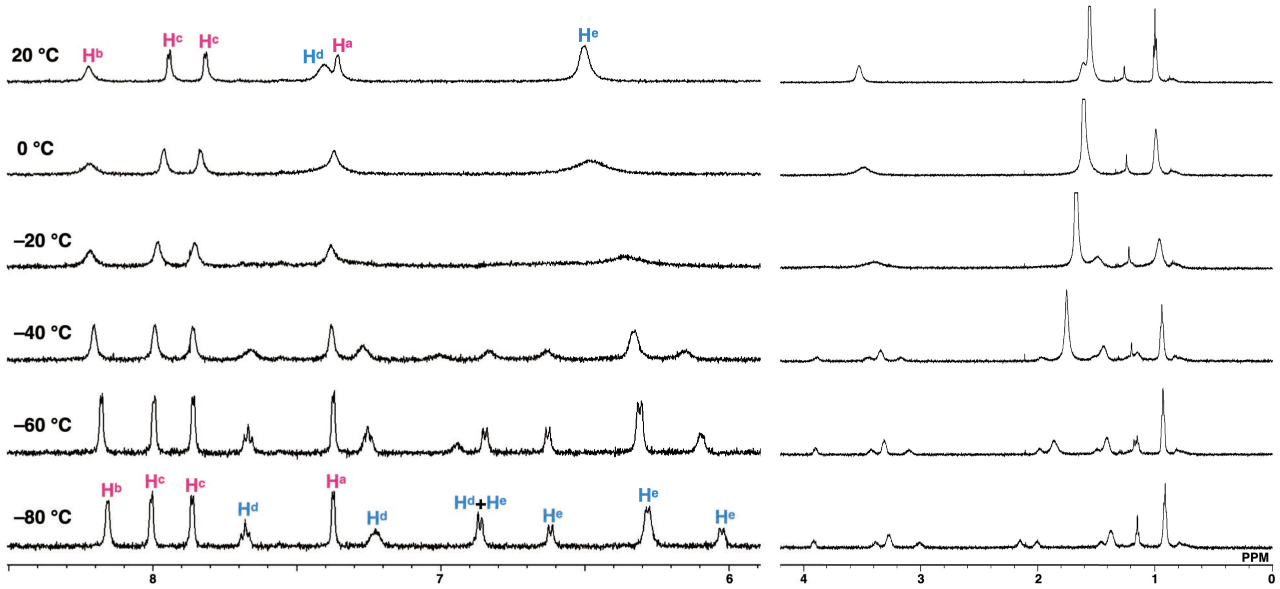

Fig. S91 VT ^{1}H NMR spectra of **2pd**$^{-}$-TATA^{+} from 20 to –80 °C in CD_2Cl_2 (1.0 × 10^{-3} M). When temperature decreased to –80 °C, one signal of **2pd**$^{-}$ shifted upfield from 8.22 to 8.16 ppm (H^{b}) and three signals of **2pd**$^{-}$ shifted downfield from 7.97 and 7.82 ppm to 8.00 and 7.86 ppm (two signals of H^{c}), respectively, and from 7.36 to 7.37 ppm (H^{a}), suggesting the tightly bound ion pair at low temperature. According to the optimized structure of **2pd**$^{-}$-TATA^{+} (Fig. S44d), π-electronic ion pair exists as a stacking structure in solution state. The upfield shift of H^{b} indicated that H^{b} is located inside the current ring of TATA^{+}, whereas downfield shifts of H^{c} upon cooling suggested the outside location of the current ring of TATA^{+}. Slight downfield shift of H^{a} is probably derived from the contributions of the shielding effect from current ring and the deshielding effect from electron-poor cationic state of TATA^{+}. Furthermore, signals of TATA^{+} appeared at 7.68 (1H), 7.23 (1H), 6.86 (2H), 6.62 (1H), 6.28 (3H), and 6.02 (1H) ppm at –80 °C. Split signals of TATA^{+} suggested the slow rotation of TATA^{+} at –80 °C due to tight ion-pairing. ^{1}H–^{1}H COSY of **2pd**$^{-}$-TATA^{+} in CD_2Cl_2 at –80 °C (1.0 × 10^{-3} M) showed the correlation between signals (ppm) at 8.16–7.36, 8.00–7.86, 7.68–6.86/6.62, 7.23–6.28, and 6.86–6.02, suggesting that the corresponding protons were located at the vicinal positions (Fig. S92a).

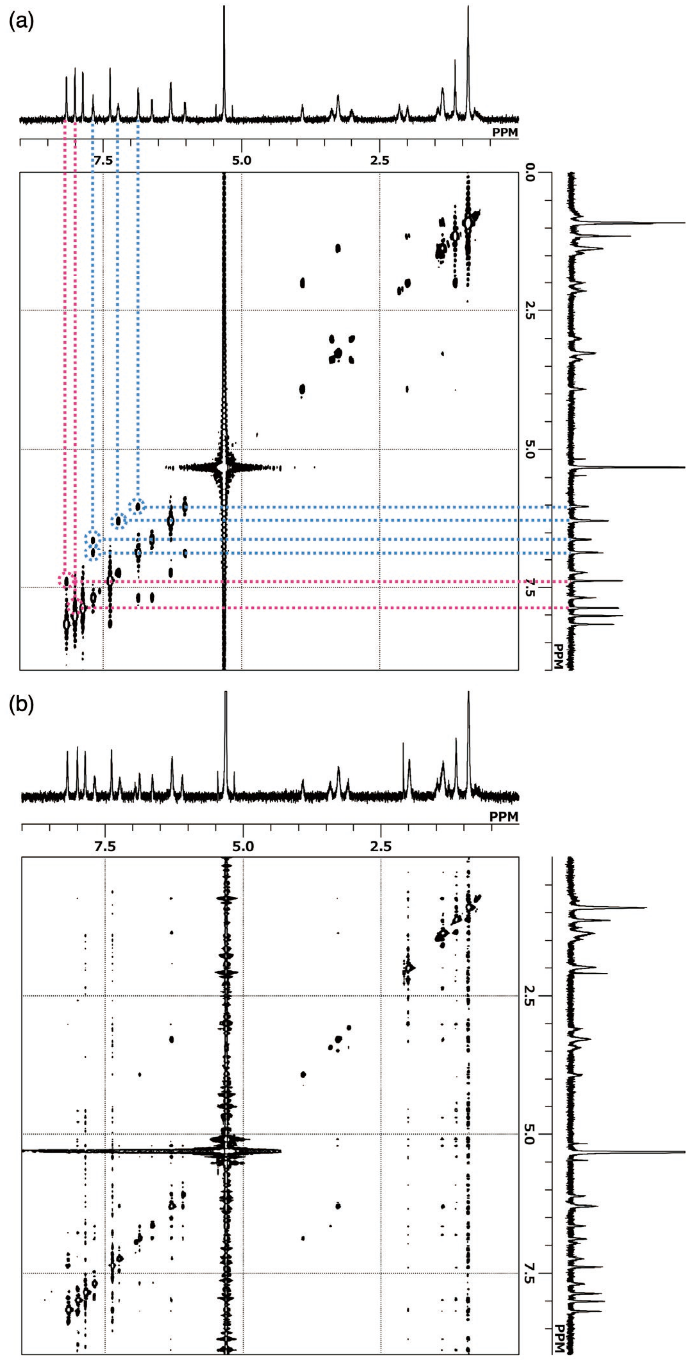

Fig. S92 (a) ^{1}H–^{1}H COSY and (b) ^{1}H–^{1}H NOESY spectra of **2pd**$^{-}$-TATA^{+} at –80 °C in CD_2Cl_2 (1.0 × 10^{-3} M).

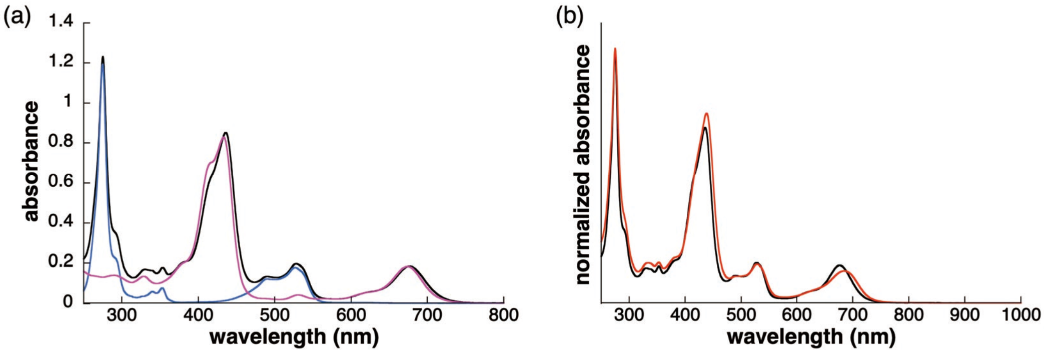

Fig. S93 UV/vis absorption spectra of (a) **2ni**$^-$-TATA$^+$ (black), TATA$^+$-Cl$^-$ (cyan), and **2ni**$^-$-TBA$^+$ (magenta) with the concentrations of 1×10^{-5} M and (b) **2ni**$^-$-TATA$^+$ with concentrations of 1×10^{-5} M (black) and 1×10^{-3} M (red) in CH_2Cl_2 at 25 °C. Independent absorption bands for each π-electronic ion suggest very weak exciton coupling between π-electronic anions and cations. The charge-transfer transition estimated by DFT calculation (Fig. S62) was not observed.

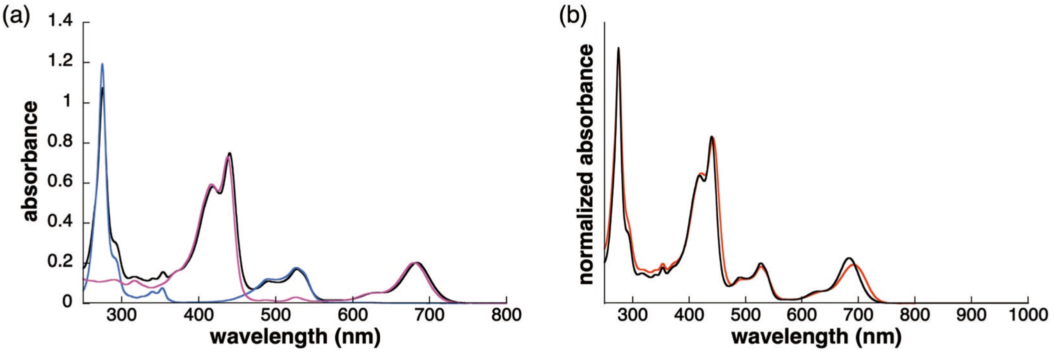

Fig. S94 UV/vis absorption spectra of (a) **2pd**$^-$-TATA$^+$ (black), TATA$^+$-Cl$^-$ (cyan), and **2pd**$^-$-TBA$^+$ (magenta) with the concentrations of 1×10^{-5} M and (b) **2pd**$^-$-TATA$^+$ with concentrations of 1×10^{-5} M (black) and 1×10^{-3} M (red) in CH_2Cl_2 at 25 °C. Independent absorption bands for each π-electronic ion suggest very weak exciton coupling between π-electronic anions and cations. The charge-transfer transition estimated by DFT calculation (Fig. S63) was not observed.

6. Solid-state properties of ion pairs

Method for steady-state absorption measurements of single crystals. An inverted optical microscope (IX71, Olympus) was used for steady-state absorption measurements of single crystals. The halogen lamp placed above the sample stage was employed as the incident light, and focused on the sample microcrystal with the objective lens (×40, NA 0.40). The transmitted light was collected with the objective lens (×60, NA 0.70), and then passed through a confocal pinhole (1 μm in diameter) to select the monitoring position which was the same in the femtosecond pump-probe measurement. The intensity was detected with another CCD camera (DU970P, Andor) coupled with another polychrometer (250is, Chromex). The absorption spectra were calculated by using reference light which was transmitted through only coverslip.

Method for femtosecond transient absorption measurements of single crystals. Transient absorption measurements on the sub-picosecond to nanosecond time scale were conducted by homemade pump-probe systems. For the solution sample, an amplified femtosecond laser, Spirit One 1040-8 (Spectra-Physics, 1040 nm, the pulse width: ~270 fs), was split into two beams with a ratio of 1:9. The stronger beam was directed to a noncollinear optical parametric amplifier (NOPA), Spirit-NOPA-3H (Spectra-Physics) to generate the 680-nm femtosecond laser pulse for the pump beam. The pump beam was chopped prior to the sample at 500 Hz for signal differencing. The other weaker beam was focused to a deuterated water placed in a 10-mm quartz cuvette to generate the white light continuum for the probe beam. Both pump and probe beams were focused to the sample solution placed in the 2-mm quartz cuvette. The polarization between the pump and probe pulses was set at magic angle. The transmitted probe beam was detected with multichannel detection system, PK120-C-RK (Unisoku), composed of a CMOS linear image sensor and a polychromator. The obtained spectra were calibrated for group velocity dispersion using the data obtained by the optical Kerr signal of $CHCl_3$ between the pump pulse and the white-light continuum. The instrumental response function was ~80 fs. The sample solution was stirred with a stirrer during the experiments and the measurements were performed under nitrogen atmosphere at r.t.

Femtosecond transient absorption measurements of crystals were carried out using 100-fs pulses (800 nm) from a Ti:Sapphire chirped pulse amplified system (Tsunami and Spitfire-ACE, Spectra-Physics) operating at 1 kHz repetition rate under the microscope.[S20,21] The output beam of the amplified system was divided into two using a beam splitter (80% – 20%). The stronger beam was guided into optical parametric oscillator and amplifier (OPA-800, Spectra-Physics), where the excitation wavelength was tuned to 650 nm and the pulse duration was ca. 150 fs in fwhm. These excitation pulses were chopped at a 500 Hz repetition rate with an optical chopper (S2000, Thorlabs) and were circularly polarized by Berek compensator (Newport). After passing through the concave and convex lenses to change the beam diameter at sample position, the excitation pulses were guided into an inverted optical microscope (IX71, Olympus), and focused at the sample with an objective lens (×60, NA 0.70). The excitation intensity was set to be ca. 0.1 mJ/cm^2 with a neutral density filter to avoid the photodecomposition. A small portion of the weaker fundamental beam was focused into a CaF_2 window (3-mm thickness) to generate the white-light continuum in the wavelength range of 380 to 860 nm, which was used as probe light. The probe pulses were guided into the microscope co-linearly with the pump pulses, and focused into the sample with the same ×60 objective lens. The transmitted light of the probe was collected with an objective lens (×40, NA 0.40), and then the intensity was detected with a CCD camera (BU-54DUV, Bitran) coupled with a polychrometer (250is, Chromex). The time delay between pump and probe pulses was carefully varied from –10 ps to 600 ps by a computer controlled translation stage (STM-150, SIGMA Koki). The temporal cross correlation of the pump and probe pulses at the sample position was 350 fs in fwhm. The chirping of the monitoring white light continuum was corrected for transient absorption spectra. The diameter of the pump beams was about 1.2 mm in fwhm at the focusing point, which was larger than that of the probe. The smooth and flat area of single microcrystal was selected.

Electrochemical analysis. Cyclic voltammograms (CVs) were measured under Ar atmosphere in CH_3CN solutions containing the sample and TBA^+-PF_6^- (0.1 M) as a supporting electrolyte using an ALS/CH Instruments 720 electrochemical analyzer with a glassy-carbon disk working electrode (3-mm diameter), an Ag/$AgNO_3$ (0.010 M) reference electrode, and a Pt counter electrode. Spectroelectrochemical analyses were conducted using an ALS/CH Instruments 720 electrochemical analyzer and a JASCO V-670 spectrometer with a thin layer quartz glass cell (path length: 1.0 mm), a Pt mesh working electrode, an Ag/$AgNO_3$ (0.010 M) reference electrode, and a Pt counter electrode. The supporting electrolyte was dried in vacuo at 100 °C for 1 day prior to use.

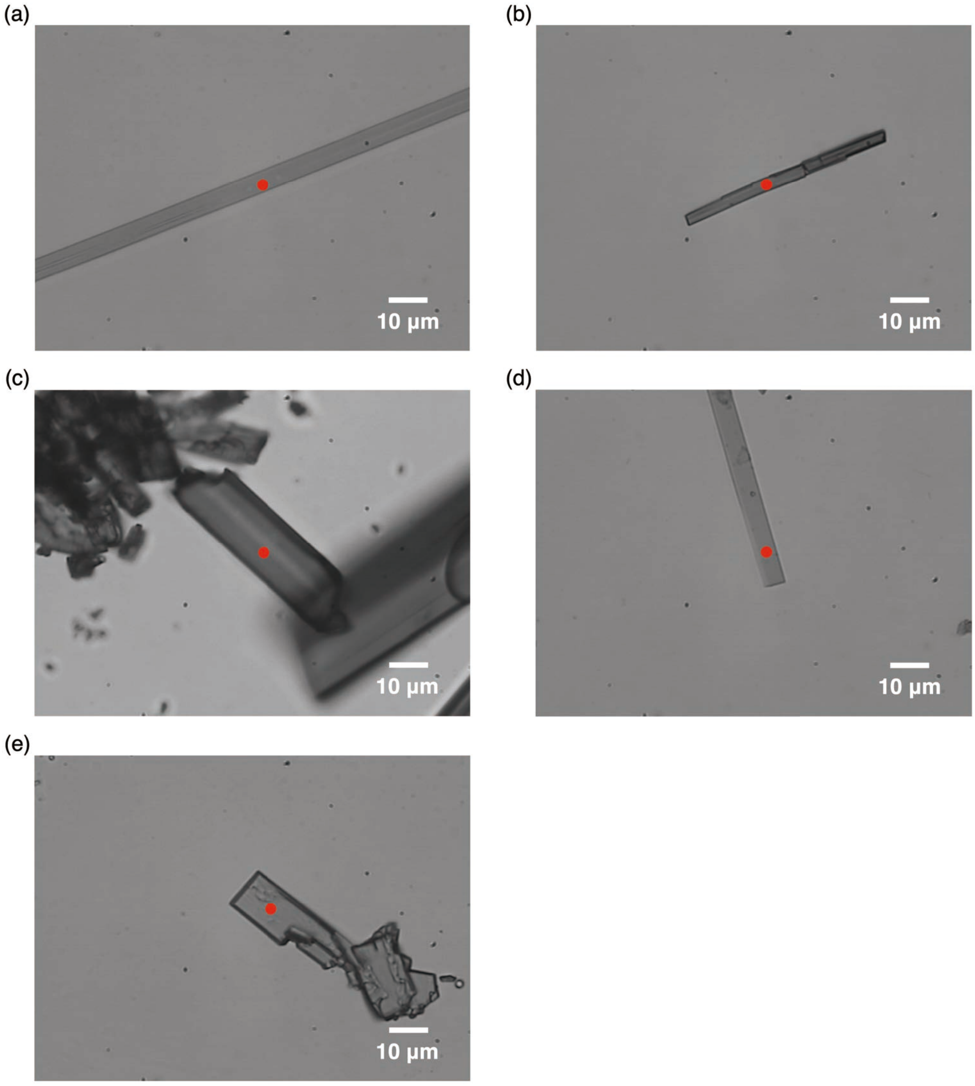

Fig. S95 Optical microscopic images of single crystals of (a) **2ni**$^{–}$-TBA^{+}, (b) **2ni**$^{–}$-TATA^{+}, (c) **2pd**$^{–}$-TBA^{+}, (d) **2pd**$^{–}$-TATA^{+}, and (e) TATA^{+}-Cl$^{–}$. Red spots indicate the position where the UV/vis absorption measurements were conducted (Fig. S96).

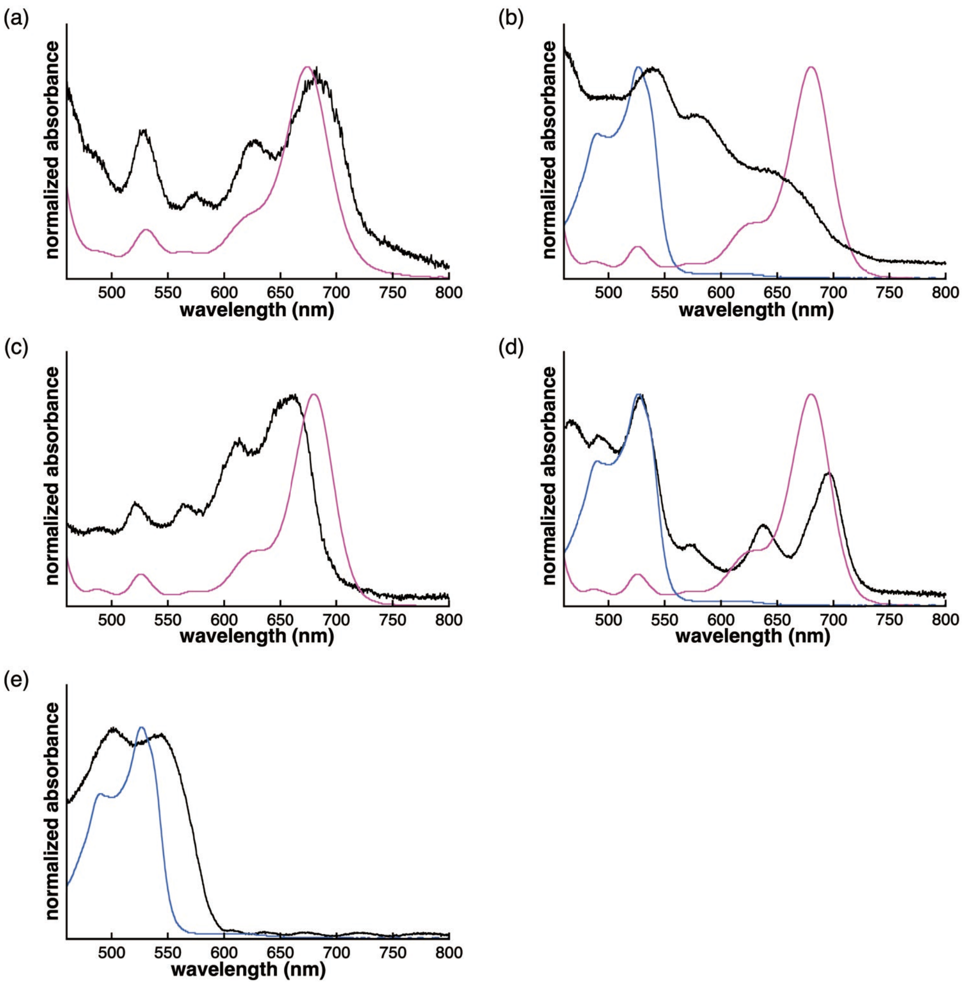

Fig. S96 UV/vis absorption spectra of (a) **2ni**$^-$-TBA$^+$ in the form of a single crystal (black) and in CH_2Cl_2 (1×10^{-5} M) (magenta) (Fig. S93a), (b) **2ni**$^-$-TATA$^+$ in the form of a single crystal (black) and **2ni**$^-$-TBA$^+$ (magenta) and TATA$^+$-Cl$^-$ (cyan) in CH_2Cl_2 (1×10^{-5} M) (Fig. S93a), (c) **2pd**$^-$-TBA$^+$ in the form of a single crystal (black) and in CH_2Cl_2 (1×10^{-5} M) (magenta) (Fig. S94a), (d) **2pd**$^-$-TATA$^+$ in the form of single crystals (black) and **2pd**$^-$-TBA$^+$ (magenta) and TATA$^+$-Cl$^-$ (cyan) in CH_2Cl_2 (1×10^{-5} M) (Fig. S94a), and (e) TATA$^+$-Cl$^-$ in the form of a single crystal (black) and in CH_2Cl_2 (1×10^{-5} M) (cyan) (Fig. S93a,94a). It should be emphasized that the UV/vis absorption spectrum of the single crystal of **2ni**$^-$-TATA$^+$, with the contribution of charge-segregated assembly (Fig. S28,29), showed significantly broad bands with the λ_{max} at 539 and 580 nm with a shoulder at 650 nm. The shoulder at 650 nm was blue-shifted (~600 cm^{-1}) from the band at 677 nm of the monomeric **2ni**$^-$, whereas the bands at 539 and 580 nm were red-shifted (~1900 and ~1700 cm^{-1}) from those at 490 and 527 nm of monomeric TATA$^+$. Therefore, the broad absorption bands of the solid-state **2ni**$^-$-TATA$^+$, with the shifts from monomeric states, can originate from the exciton coupling of proximally located identically charged π-electronic systems. The **2ni**$^-$ units, with the side-by-side-arrangement in the solid-state **2ni**$^-$-TATA$^+$, showed the parallel-arranged transition dipole moments (Fig. S83), inducing the blue-shifted absorption band. On the other hand, TATA$^+$ formed slipped-stacking dimeric structures with head-to-tail-arranged transition dipole moments of TATA$^+$ (Fig. S83), resulting in the red-shifted absorption bands. Focusing on other ion pairs, the crystal-state absorption band of **2ni**$^-$-TBA$^+$, in the form of charge-by-charge assembly,[S5b] was identical to that of monomeric **2ni**$^-$ due to the arrangement of **2ni**$^-$ (Fig. S82). In **2pd**$^-$-TBA$^+$, the band at 662 nm was blue-shifted (~400 cm^{-1}) from the band at 680 nm of the monomeric **2pd**$^-$ because of the parallel arrangement of **2pd**$^-$ in the charge-by-charge assembly (Fig. S84). The crystal-state absorption spectra of **2pd**$^-$-TATA$^+$ showed red-shifted absorption bands for **2pd**$^-$ and almost no shift for TATA$^+$ because of their arrangements (Fig. S85). Furthermore, the crystal-state absorption spectrum of TATA$^+$-Cl$^-$, with the contribution of charge-segregated assembly,[S4] indicated that the bands at 501 and 544 nm were red-shifted (~400 and ~600 cm^{-1}) from those at 490 and 527 nm of monomeric TATA$^+$. TATA$^+$ formed slipped-stacking dimeric structures with head-to-tail-arranged transition dipole moments of TATA$^+$ (Fig. S86), resulting in the red-shifted absorption bands.

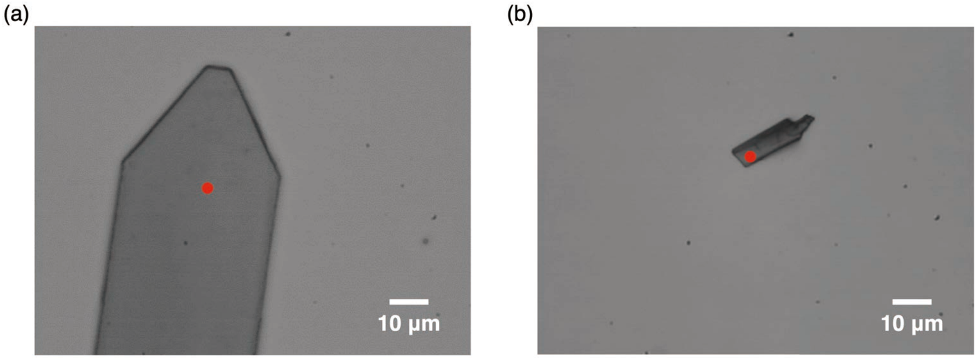

Fig. S97 Optical microscopic images of single crystals of (a) **2ni**⁻-TBA⁺ and (b) **2ni**⁻-TATA⁺. Red spots indicate the position where the transient absorption measurements were conducted (Fig. S98,99).

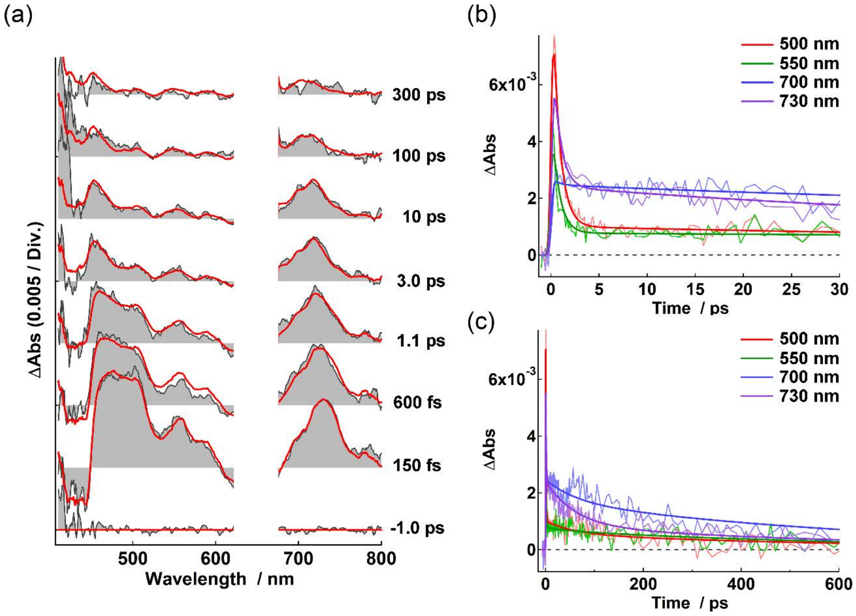

Fig. S98 (a) Transient absorption spectra of a single crystal of **2ni**⁻-TBA⁺ excited at 650 nm (<100 μJ/cm^2), wherein the red lines indicate the fitting lines by global analyses, and (b,c) transient absorption dynamics of a single crystal of **2ni**⁻-TBA⁺ at different probe wavelengths, wherein thin and thick lines indicate the experimental data and fitting data by the global analyses, respectively.

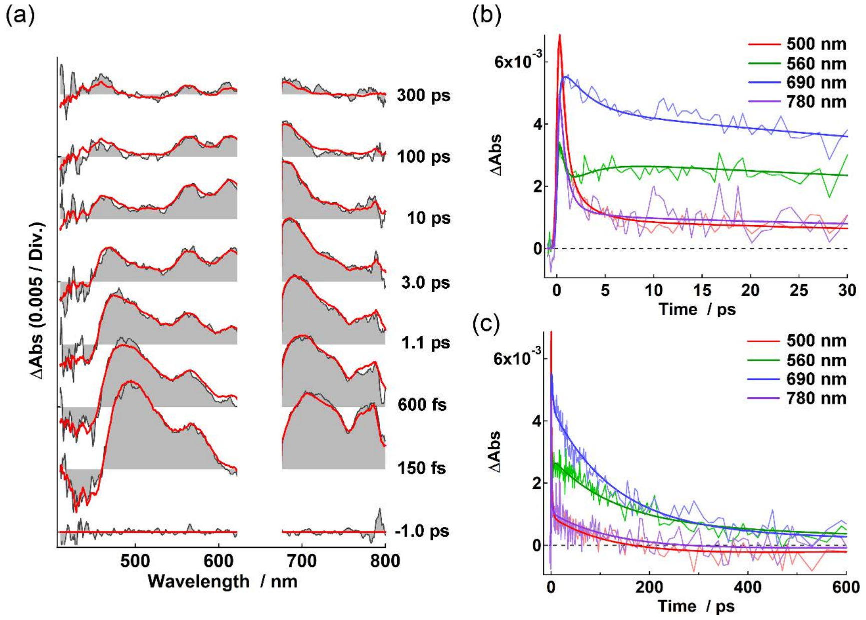

Fig. S99 (a) Transient absorption spectra of a single crystal of **2ni**⁻-TATA⁺ excited at 650 nm (<100 μJ/cm²), wherein the red lines indicate the fitting lines by global analyses, and (b,c) transient absorption dynamics of **2ni**⁻-TATA⁺ at different probe wavelengths, wherein thin and thick lines indicate the experimental data and fitting data by the global analyses, respectively.

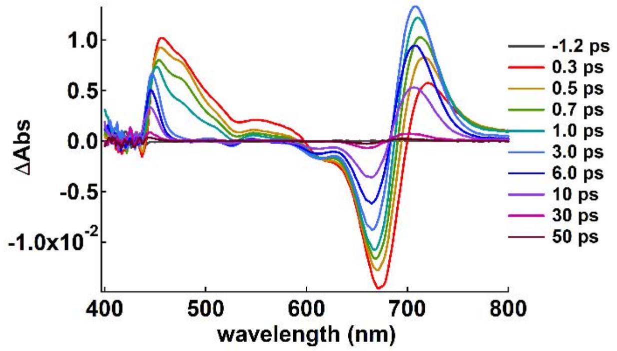

Fig. S100 Transient absorption spectra of **2ni**⁻-TBA⁺ in CH_2Cl_2 (1.8×10^{-4} M) excited at 680 nm (~500 μJ/cm²) with delay time spanning from –1.2 ps to 50 ps.

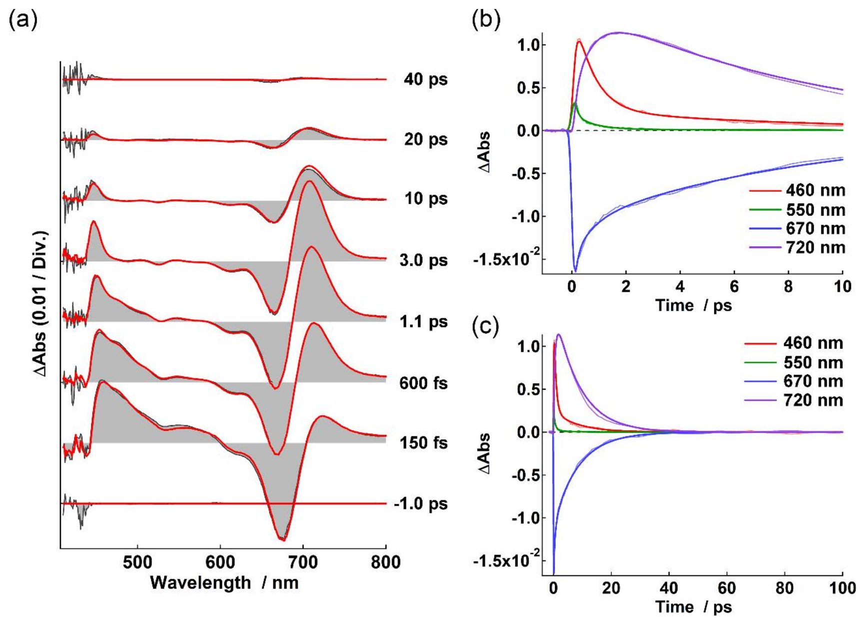

Fig. S101 (a) Transient absorption spectra of **2ni**$^{-}$-TBA^{+} in CH_2Cl_2 (1.8×10^{-4} M) excited at 680 nm (~500 μJ/cm^2), wherein the red lines indicate the fitting lines by global analyses, and (b,c) transient absorption dynamics of **2ni**$^{-}$-TBA^{+} in CH_2Cl_2 at different probe wavelengths, wherein thin and thick lines indicate the experimental data and fitting data by the global analyses, respectively.

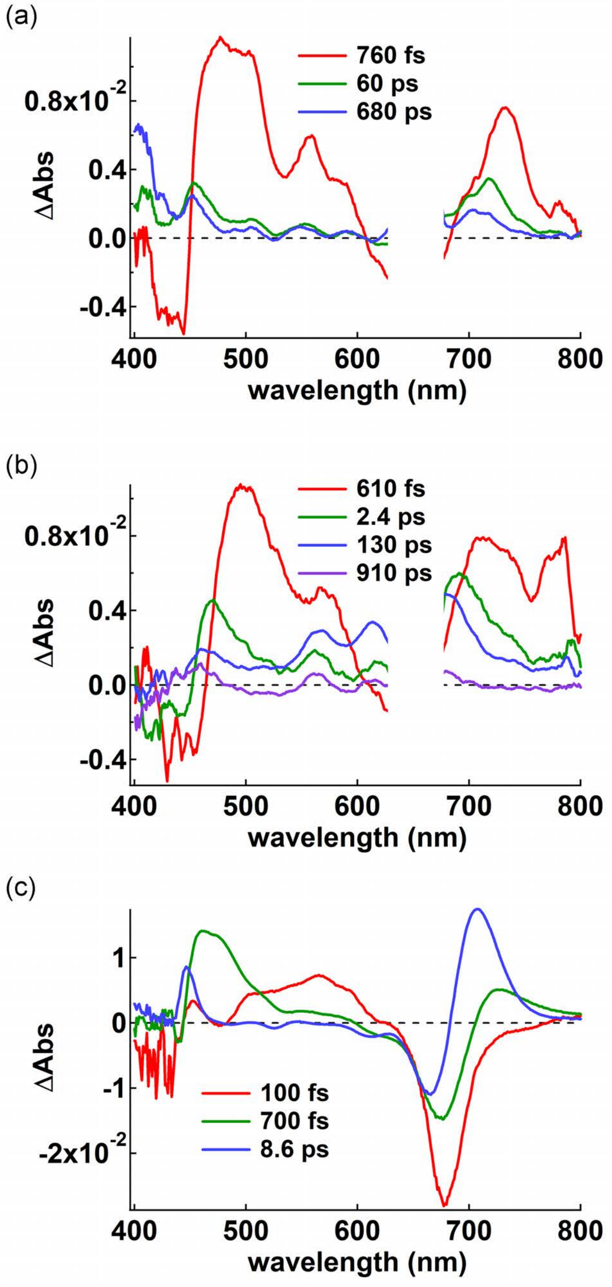

Fig. S102 Evolution-associated difference absorption spectra (EADS) of (a) **2ni**⁻-TBA⁺ and (b) **2ni**⁻-TATA⁺ as single crystals excited at 650 nm and (c) **2ni**⁻-TBA⁺ in CH_2Cl_2 excited at 680 nm.

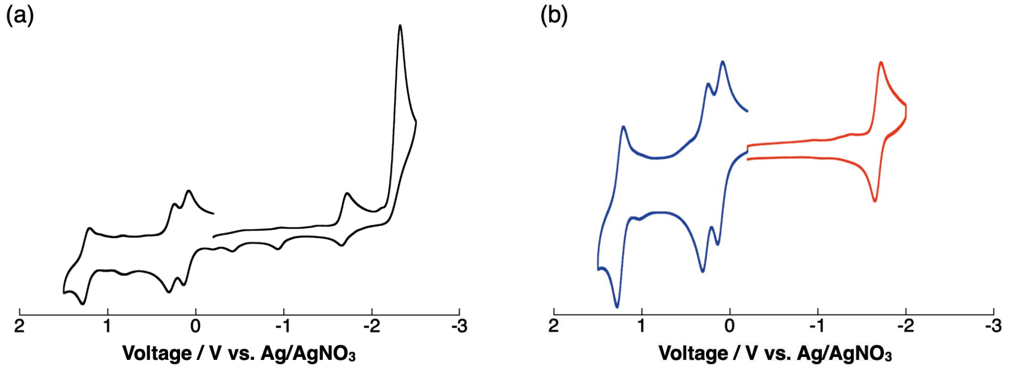

Fig. S103 CVs of **2ni**$^{-}$-TBA^{+} in CH_3CN (5.8×10^{-4} M) containing TBA^{+}-PF_6^{-} (0.1 M) as electrolyte under Ar atmosphere: (a) CV at a scan rate of 100 mV/s and (b) anodic scans from –0.2 to 1.5 V at a scan rate of 100 mV/s (blue) and cathodic scans from –0.2 to –2.0 V at a scan rate of 100 mV/s (red). The reversible waves in the anodic and cathodic scans confirmed that the first reduction at –1.68 V as well as oxidation at +0.11, +0.28, and +1.25 V are reversible processes.

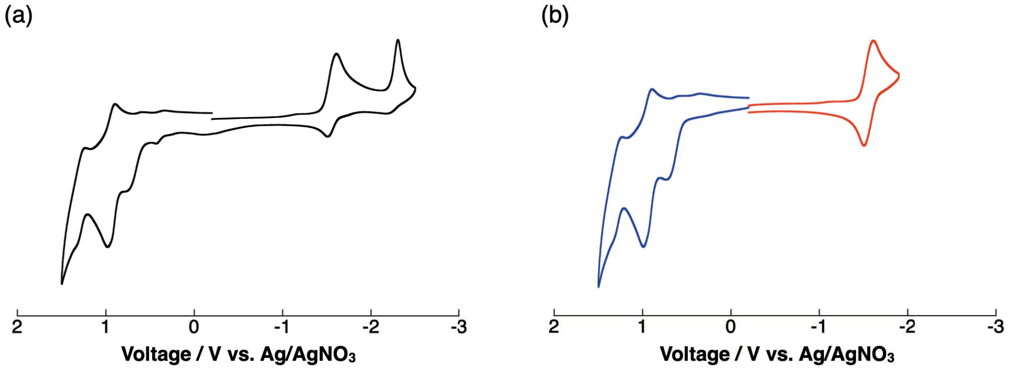

Fig. S104 CVs of TATA^{+}-Cl^{-} in CH_3CN (1.0×10^{-3} M) containing TBA^{+}-PF_6^{-} (0.1 M) as electrolyte under Ar atmosphere: (a) CV at a scan rate of 100 mV/s and (b) anodic scans from –0.2 to 1.5 V at a scan rate of 100 mV/s (blue) and cathodic scans from –0.2 to –1.9 V at a scan rate of 100 mV/s (red). The reversible wave in the cathodic scan confirmed that the first reduction at –1.56 V is a reversible process.

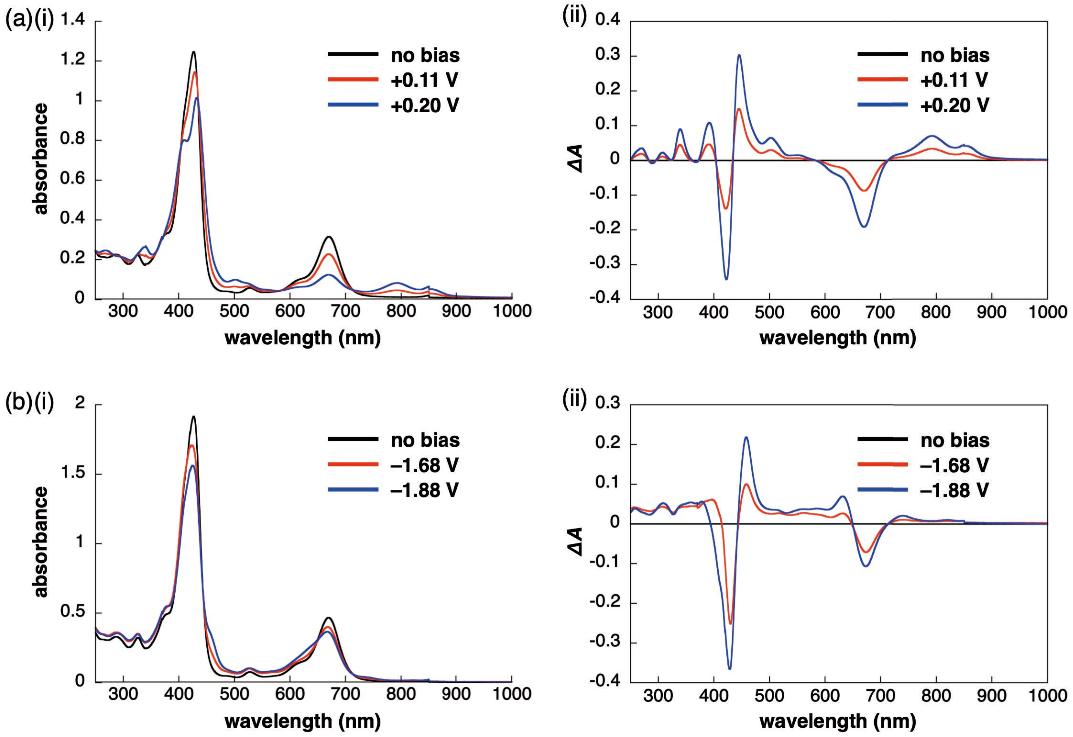

Fig. S105 Spectroelectrochemical analysis of **2ni**$^{-}$-TBA^{+} in (a) the oxidation states at +0.11 V (red) and +0.20 V (blue) and (b) the reduction states at –1.68 V (red) and –1.88 V (blue) in CH_3CN (1.2×10^{-4} M in (a) and 1.8×10^{-4} M in (b)) containing of 0.1 M TBA^{+}-PF_6^{-} as an electrolyte under Ar atmosphere: (i) UV/vis absorption spectral changes and (ii) difference spectra between no bias and applied bias. At +0.11 and +0.20 V, the molar ratios of **2ni**$^{-}$-TBA^{+} and the corresponding oxidized species [**2ni**$^{-}$-TBA^{+}]$^{+}$ are supposed to be 50:50 and 3:97, respectively. At –1.68 and –1.88 V, the molar ratios of **2ni**$^{-}$-TBA^{+} and the corresponding reduced species [**2ni**$^{-}$-TBA^{+}]$^{-}$ are supposed to be 50:50 and 1:200, respectively. The broad peak at 795 nm in the oxidation states can be assigned as a component of **2ni** radical: the theoretically estimated excitation at 720 nm includes the major transition from SOMOα to SOMO+1α, both of which are localized at the core π-system (Fig. S54,64).

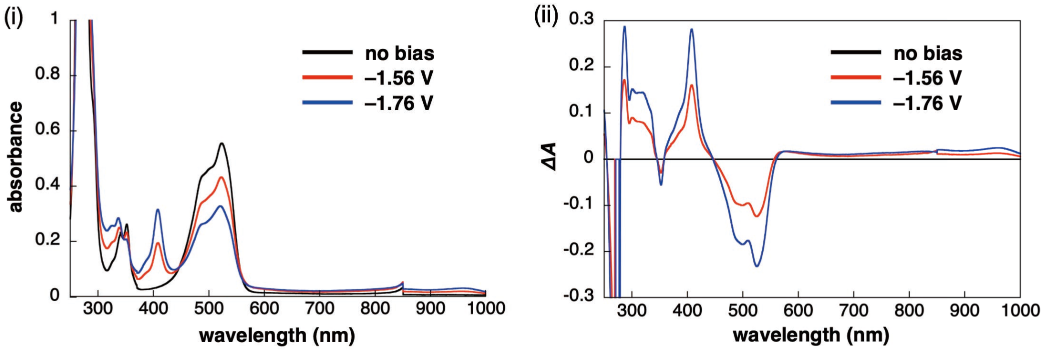

Fig. S106 Spectroelectrochemical analysis of TATA^{+}-Cl^{-} in the reduction states at –1.56 V (red) and –1.76 V (blue) in CH_3CN (3.0×10^{-4} M) containing of 0.1 M TBA^{+}-PF_6^{-} as an electrolyte under Ar atmosphere: (i) UV/vis absorption spectral changes and (ii) difference spectra between no bias and applied bias. At –1.56 and –1.76 V, the molar ratios of TATA^{+}-Cl^{-} and the corresponding reduced species [TATA^{+}-Cl^{-}]$^{-}$ are supposed to be 50:50 and 1:200, respectively. The broad peak at 960 nm in the reduction states can be assigned as a component of TATA radical: the theoretically estimated excitations at 902 and 903 nm include the respective major transitions from SOMOα and SOMOα to SOMO+2α and SOMO+1α, respectively, all of which are localized at the core π-system (Fig. S55,65).

References

[S1] J. R. Frost, S. M. Huber, S. Breitenlechner, C. Bannwarth and T. Bach, *Angew. Chem. Int. Ed.*, 2015, **54**, 691–695.

[S2] L. J. Esdaile, M. O. Senge and D. P. Arnold, *Chem. Commum.*, 2006, 4192–4194.

[S3] (a) B. W. Laursen and F. C. Krebs, *Angew. Chem. Int. Ed.*, 2000, **39**, 3432–3434; (b) B. W. Laursen and F. C. Krebs, *Chem. Eur. J.*, 2001, **7**, 1773–1783.

[S4] Y. Haketa, S. Sasaki, N. Ohta, H. Masunaga, H. Ogawa, N. Mizuno, F. Araoka, H. Takezoe and H. Maeda, *Angew. Chem. Int. Ed.*, 2010, **49**, 10079–10083.

[S5] (a) C. Stähler, D. Shimizu, K. Yoshida, K. Furukawa, R. Herges and A. Osuka, *Chem. Eur. J.*, 2017, **23**, 7217–7220; (b) Y. Sasano, N. Yasuda and H. Maeda, *Dalton Trans.*, 2017, **46**, 8924–8928.

[S6] A. Z. Muresan, P. Thamyongkit, J. R. Diers, D. Holten, J. S. Lindsey and D. F. Bocian, *J. Org. Chem.*, 2008, **73**, 6947–6959.

[S7] (a) N. Yasuda, H. Murayama, Y. Fukuyama, J. E. Kim, S. Kimura, K. Toriumi, Y. Tanaka, Y. Moritomo, Y. Kuroiwa, K. Kato, H. Tanaka and M. Takata, *J. Synchrotron Rad.*, 2009, **16**, 352–357; (b) N. Yasuda, Y. Fukuyama, K. Toriumi, S. Kimura and M. Takata, *AIP Conf. Proc.*, 2010, **1234**, 147–150.

[S8] G. M. Sheldrick, *Acta Crystallogr. Sect. A*, 2008, **64**, 112–122.

[S9] (a) *Yadokari-XG*, *Software for Crystal Structure Analyses*, K. Wakita, 2001; (b) C. Kabuto, S. Akine, T. Nemoto and E. Kwon, *J. Cryst. Soc. Jpn.*, 2009, **51**, 218–224.

[S10] *CrystalExplorer17*, M. J. Turner, J. J. McKinnon, S. K. Wolff, D. J. Grimwood, P. R. Spackman, D. Jayatilaka and M. A. Spackman, University of Western Australia, 2017.

[S11] (a) J. J. McKinnon, M. A. Spackman and A. S. Mitchell, *Acta Crystallogr. Sect. B*, 2004, **60**, 627–668; (b) S. L. Tan, M. M. Jotani and E. R. T. Tiekink, *Acta Crystallogr. Sect. E*, 2019, **75**, 308–318.

[S12] Y. Bando, Y. Haketa, T. Sakurai, W. Matsuda, S. Seki, H. Takaya and H. Maeda, *Chem. Eur. J.*, 2016, **22**, 7843–7850.

[S13] *Gaussian 09*, revision D.01, M. J. Frisch, G. W. Trucks, H. B. Schlegel, G. E. Scuseria, M. A. Robb, J. R. Cheeseman, G. Scalmani, V. Barone, B. Mennucci, G. A. Petersson, H. Nakatsuji, M. Caricato, X. Li, H. P. Hratchian, A. F. Izmaylov, J. Bloino, G. Zheng, J. L. Sonnenberg, M. Hada, M. Ehara, K. Toyota, R. Fukuda, J. Hasegawa, M. Ishida, T. Nakajima, Y. Honda, O. Kitao, H. Nakai, T. Vreven, J. A. Montgomery, Jr., J. E. Peralta, F. Ogliaro, M. Bearpark, J. J. Heyd, E. Brothers, K. N. Kudin, V. N. Staroverov, T. Keith, R. Kobayashi, J. Normand, K. Raghavachari, A. Rendell, J. C. Burant, S. S. Iyengar, J. Tomasi, M. Cossi, N. Rega, J. M. Millam, M. Klene, J. E. Knox, J. B. Cross, V. Bakken, C. Adamo, J. Jaramillo, R. Gomperts, R. E. Stratmann, O. Yazyev, A. J. Austin, R. Cammi, C. Pomelli, J. W. Ochterski, R. L. Martin, K. Morokuma, V. G. Zakrzewski, G. A. Voth, P. Salvador, J. J. Dannenberg, S. Dapprich, A. D. Daniels, Ö. Farkas, J. B. Foresman, J. V. Ortiz, J. Cioslowski and D. J. Fox, Gaussian, Inc., Wallingford CT, 2013.

[S14] (a) R. Herges and D. Geuenich, *J. Phys. Chem. A*, 2001, **105**, 3214–3220; (b) D. Geuenich, K. Hess, F. Köhler and R. Herges, *Chem. Rev.*, 2005, **105**, 3758–3772.

[S15] M. J. S. Phipps, T. Fox, C. S. Tautermann and C.-K. Skylaris, *Chem. Soc. Rev.*, 2015, **44**, 3177–3211.

[S16] Articles for *GAMESS*: (a) M. W. Schmidt, K. K., Baldridge, J. A. Boatz, S. T. Elbert, M. S. Gordon, J. H. Jensen, S. Koseki, N. Matsunaga, K. A. Nguyen, S. J. Su, T. L. Windus, M. Dupuis and J. A. Montgomery, *J. Comput. Chem.*, 1993, **14**, 1347−1363; (b) M. S. Gordon and M. W. Schmidt, in *Theory and Applications of Computational Chemistry: the first forty years*, ed. C. E. Dykstra, G. Frenking, K. S. Kim and G. E. Scuseria, Elsevier, 2005, pp.1167–1189.

[S17] Report for FMO: K. Kitaura, E. Ikeo, T. Asada, T. Nakano and M. Uebayasi, *Chem. Phys. Lett.*, 1999, **313**, 701–706.

[S18] Report for pair interaction energy decomposition analysis (PIEDA): D. G. Fedorov and K. Kitaura, *J. Comput. Chem.*, 2007, **28**, 222–237.

[S19] M. Kasha, H. R. Rawls and M. Ashraf El-Bayoumi, *Pure Appl. Chem.*, 1965, **11**, 371–392.

[S20] Y. Ishibashi, Y. Inoue and T. Asahi, *Photochem. Photobiol. Sci.*, 2016, **15**, 1304–1309.

[S21] Y. Ishibashi, M. Murakami, K. Araki, T. Mutai and T. Asahi, *J. Phys. Chem. C*, 2019, **123**, 11224–11232.
